# Accuracy of Resting Metabolic Rate Prediction Equations in Athletes: A Systematic Review with Meta-analysis

**DOI:** 10.1007/s40279-023-01896-z

**Published:** 2023-08-26

**Authors:** Jack Eoin Rua O’Neill, Clare A. Corish, Katy Horner

**Affiliations:** 1 Institute for Sport and Health and School of Public Health, Physiotherapy and Sport Science, University College Dublin, Belfield, Dublin 4, Ireland; 2 School of Public Health, Physiotherapy and Sport Science, University College Dublin, Dublin 4, Ireland

## Abstract

**Background:**

Resting metabolic rate (RMR) prediction equations are often used to calculate RMR in athletes; however, their accuracy and precision can vary greatly.

**Objective:**

The aim of this systematic review and meta-analysis was to determine which RMR prediction equations are (i) most accurate (average predicted values closest to measured values) and (ii) most precise (number of individuals within 10% of measured value).

**Data Sources:**

A systematic search of PubMed, CINAHL, SPORTDiscus, Embase, and Web of Science up to November 2021 was conducted.

**Eligibility Criteria:**

Randomised controlled trials, cross-sectional observational studies, case studies or any other study wherein RMR, measured by indirect calorimetry, was compared with RMR predicted via prediction equations in adult athletes were included.

**Analysis:**

A narrative synthesis and random-effects meta-analysis (where possible) was conducted. To explore heterogeneity and factors influencing accuracy, subgroup analysis was conducted based on sex, body composition measurement method, athlete characteristics (athlete status, energy availability, body weight), and RMR measurement characteristics (adherence to best practice guidelines, test preparation and prior physical activity).

**Results:**

Twenty-nine studies (mixed sports/disciplines *n* = 8, endurance *n* = 5, recreational exercisers *n* = 5, rugby *n* = 3, other *n* = 8), with a total of 1430 participants (822 F, 608 M) and 100 different RMR prediction equations were included. Eleven equations satisfied criteria for meta-analysis for accuracy. Effect sizes for accuracy ranged from 0.04 to − 1.49. Predicted RMR values did not differ significantly from measured values for five equations (Cunningham (1980), Harris-Benedict (1918), Cunningham (1991), De Lorenzo, Ten-Haaf), whereas all others significantly underestimated or overestimated RMR (*p* < 0.05) (Mifflin-St. Jeor, Owen, FAO/WHO/UNU, Nelson, Koehler). Of the five equations, large heterogeneity was observed for all (*p* < 0.05, *I*^2^ range: 80–93%) except the Ten-Haaf (*p* = 0.48, *I*^2^ = 0%). Significant differences between subgroups were observed for some but not all equations for sex, athlete status, fasting status prior to RMR testing, and RMR measurement methodology. Nine equations satisfied criteria for meta-analysis for precision. Of the nine equations, the Ten-Haaf was found to be the most precise, predicting 80.2% of participants to be within ± 10% of measured values with all others ranging from 40.7 to 63.7%.

**Conclusion:**

Many RMR prediction equations have been used in athletes, which can differ widely in accuracy and precision. While no single equation is guaranteed to be superior, the Ten-Haaf (age, weight, height) equation appears to be the most accurate and precise in most situations. Some equations are documented as consistently underperforming and should be avoided. Choosing a prediction equation based on a population of similar characteristics (physical characteristics, sex, sport, athlete status) is preferable. Caution is warranted when interpreting RMR ratio of measured to predicted values as a proxy of energy availability from a single measurement.

**PROSPERO Registration:**

CRD42020218212.

**Supplementary Information:**

The online version contains supplementary material available at 10.1007/s40279-023-01896-z.

## Key Points

**Table:** 

In most cases in this systematic review, athlete-population–derived resting metabolic rate (RMR) prediction equations were observed to be among the top performing equations, demonstrating greatest accuracy and precision. The potential for bias when assessing the performance of an equation within the same cohort from which it was derived may inflate the reported efficacy of the equation. To mitigate this risk, validation of locally derived equations within a separate cohort(s) is recommended. Until externally validated, caution is warranted before using such equations in practice.
Where possible, when choosing a RMR prediction equation, ensure that the subject characteristics of the athlete of interest are similar to the subject characteristics of the population in which the RMR equation was derived (height, weight, age, sex, fat-free mass [FFM]).
When using RMR ratio as a proxy indicator of low energy availability, use of an arbitrary or commonly used equation to detect suppression of RMR is not advised unless a specific equation has been shown to accurately predict an individual’s RMR. A more suitable use may be in longitudinal monitoring, and interpretation of directly measured RMR and body composition (i.e. RMR relative to body weight and/or FFM).

## Introduction

Accurate determination of total daily energy expenditure (TDEE) is essential for athletic performance and health [1]. Knowledge of TDEE is fundamental for calculating appropriate energy intake for athletes. In the general population, resting metabolic rate (RMR) is typically the largest component, contributing 60–75% of total daily energy expenditure [2]. In athletes, the contribution of RMR to TDEE on training days can vary widely. However, it remains a key contributor to overall TDEE. Therefore, accurate calculation of RMR is essential for determining an athlete’s overall energy requirements.

Indirect calorimetry (IC) is often referred to as the gold standard of RMR measurement. However, it is time-consuming and requires trained personnel to operate specialised equipment. To overcome this, RMR prediction equations are frequently used as an alternative method. In addition, the ratio of predicted versus measured RMR value is increasingly being used as a proxy indicator of low energy availability (LEA), whereby a ratio of 0.9 indicates energy deficiency [3–6]. However, such a method is dependent on the accuracy of the prediction equation in the first place. This emphasises the importance of establishing accurate RMR prediction equations for use in athletes.

Although population-specific equations are encouraged when possible, the American College of Sports Medicine [1] recommends the Harris-Benedict [7] and Cunningham [8] equations as giving reasonable estimates of RMR in athletes, with an activity factor (PAL) applied to estimate TDEE. These are among the most widely used equations. However, the Harris-Benedict equation was formulated over 100 years ago in a non-athletic population [7], and the Cunningham equation was similarly formulated in a population that omitted participants that were deemed ‘athletic’ [8]. These equations have regularly been shown to lack consistency when predicting RMR in both male and female athletes [9–13]. For example, the Harris-Benedict equation was found to under-estimate RMR by approximately 500 kcal on average in a group of elite male rowers and canoeists [14]. Given these athletes were highly active (PAL of 2+), calculated total daily energy requirements could be inaccurate by > 1000 kcal/day for some. Although other factors could potentially contribute to the initial difference between measured and predicted RMR (such as LEA and supressed RMR) [3–6, 15], this study highlights the errors that RMR prediction equations can have in predicting athlete energy requirements.

Other commonly used RMR prediction equations include, but are not limited to, the Owen [16], Mifflin-St. Jeor [17], Nelson [18], De Lorenzo [19], Henry [20], Ten Haaf and Weijs [21], Jagim [22], Tinsley [23], Wang [24], Watson [25], and FAO/WHO/UNU [26] equations. Multiple studies have assessed the accuracy of these RMR prediction equations in various athletic populations [3, 9, 12, 14, 23, 27–30]. However, the results of these studies have shown significant variability between cohorts. The performance of a prediction equation is likely highly dependent on homogeneity between the athlete physical characteristics and the characteristics of the population the equation was derived in, meaning any application of the prediction equation to groups differing physically from the original group may be inappropriate.

Several studies have examined the accuracy of different RMR prediction equations in athlete groups such as in collegiate athletes, recreational athletes, and female rugby players [9, 12, 21]; however, these studies have not been systematically reviewed and meta-analysed. The aim of this systematic review and meta-analysis was to determine which RMR prediction equations are (i) most accurate (average predicted values closest to measured values) and (ii) most precise (number of individuals within 10% of measured value) for predicting RMR in male and female athletes. It will also explore differences between subgroups based on key characteristics such as sex, athlete training status, and body composition to name a few.

## Methods

This systematic review and meta-analysis adhered to Preferred Reporting Items for Systematic Reviews and Meta-Analyses diagnostic test accuracy guidelines [31] and was prospectively registered in PROSPERO (CRD42020218212).

### Inclusion Criteria

The inclusion criteria for the review were: randomised controlled trials (RCTs), cross-sectional observational studies, case studies or any other study wherein RMR, measured by IC, was compared with RMR predicted via prediction equations. Studies had to report the following outcomes (or have them extrapolatable): (i) accuracy of a prediction equation (i.e., predicted energy expenditure expressed as a percentage of the measured energy expenditure) and/or (ii) precision of a prediction equation (i.e., the percentage/number of participants predicted correctly within 10% of their measured values). An accurate RMR prediction equation has previously been defined as predicting within 10% of measured values in studies and reviews in athletes [9, 27, 28, 32, 33], and in the general population [34–36]. This has been justified as consistent with IC measurement errors of ≤ 5% [36, 37]. Studies had to include male and/or female competitive and/or recreational athletes who were > 18 years old. The study had to be a full-text article, in English.

### Exclusion Criteria

The study could not be a review article, a commentary or an animal study, in children only, in pregnant/lactating women, in hospitalised patients, in individuals with physical disabilities/conditions and/or the presence of disease, include medications or known stimulant or drug use, or in older adults (≥ 65 years).

### Search Strategy

A systematic search of the literature was initially conducted on 10 December 2020 and updated on 12 November 2021 (by JERO). Search terms included keywords and subject headings in the following areas: athletes, sports, exercise, resting metabolic rate, and resting metabolic rate prediction equations. The search strategy was applied across electronic bibliographic and grey literature databases; MEDLINE via PubMed; EMBASE via Ovid; CINAHL and SportDiscus via Ebsco; and Web of Science (see Supplementary Document 1 in the electronic supplementary material [ESM]).

### Study Selection

Other potentially relevant studies were identified by hand-searching reference lists of included articles and reviews (JERO). All articles were uploaded for deduplication and title and abstract screening via Covidence (Veritas Health Innovation Ltd, Melbourne, Australia) (JERO). After deduplication, articles were screened based on title and abstract for eligibility independently by two authors (JERO, KH). Where discrepancies between reviewers were noted, eligibility was agreed on by discussion (JERO, KH). For the title and abstract screening stage, a kappa value of 0.77 was observed demonstrating substantial agreement between the reviewers, according to the guidelines by Landis and Koch [38]. Full-text articles were then independently reviewed by two authors for inclusion in this systematic review (JERO, KH). Where any discrepancies between reviewers were noted, eligibility was agreed on by discussion (JERO, KH). At the full-text screening stage, a higher kappa value was observed, 0.93, indicating almost perfect agreement [38]. For any potentially eligible articles with missing/unclear information, or where data were not possible to extract, corresponding authors were contacted, and data/information requested (JERO). If no response was received to this or a follow-up data/information request, the article was excluded. This resulted in the exclusion of two articles from 11 requested.

### Data Extraction

Pre-determined variables were independently gathered from each included study (JERO, KH). Variables included study characteristics (study title, study design, year of publication, authors, journal, funding sources), athlete characteristics (number of participants, nationality, sex, age, exercise training [hours and number of sessions per week], sport participated in, training and performance calibre, body weight [kg], height [m], body fat [%], fat-free mass [kg]), RMR prediction equation(s) used (name of equation, year equation was formulated, equation formula, performance of equation [accuracy and/or precision]), RMR measured by IC (name and brand of equipment used, position of participant, test duration, definition of steady state, measured RMR, respiratory exchange ratio), study limitations, and any other additional noteworthy points of information from the authors (such as conclusions, new hypotheses and/or recommendations for future research). Differentiation between levels of training and performance was determined using the athlete classification framework by McKay et al. [39] (see Supplementary Document 2 in the ESM). The outcomes extracted were mean (SD) predicted and measured RMR in kcal/24 h or converted to kcal/24 h where necessary and/or the precision of the prediction equation as the number/percentage of individuals predicted within 10% of measured values.

### Meta-analysis Data Synthesis

Meta-analysis for accuracy was subsequently conducted comparing measured versus predicted RMR values where a prediction equation was compared against IC in at least three separate studies. RMR predicted by prediction equation, RMR measured via IC, SD, and sample sizes were used to calculate the effect size (ES) and 95% confidence intervals (CIs) for each equation using Revman software (version 5.4.1) [40, 41]. A negative ES represents an underestimation of the predicted RMR value relative to the measured value via IC, and a positive value represents an overestimation. Interpretation of ES was as follows: < 0.20 as trivial, 0.20–0.39 as small, 0.40–0.80 as moderate and > 0.80 as large [42]. A random effects model was employed for all analyses based on the assumption that heterogeneity would exist between included studies due to the variability in athlete characteristics and study design [43]. To determine heterogeneity, the *I*^2^ statistic was used. Depending on the magnitude and direction of effect, *I*^2^ values from 0 to 40% are likely to lack importance, values from 30 to 60% may represent moderate heterogeneity, values from 50 to 90% may represent substantial heterogeneity, and values from 75 to 100% may represent considerable heterogeneity [44].

Meta-analysis for precision was conducted by pooling the number of participants whose RMR was predicted by RMR prediction equation within ± 10% of RMR measured via IC for each included equation. For an equation to be included in this meta-analysis, a minimum of three separate studies had to report precision values (either ratio or %) for the equation. Once pooled, the weighted mean (%) was calculated for each included equation.

### Subgroup and Sensitivity Analysis

For inclusion in the subgroup analysis, a subgroup had to contain at least three separate comparisons from three separate studies. Subgroup analysis was performed for the following categories which satisfied criteria: sex (females, males), body composition measurement method (DXA, BIA, Bodpod), energy availability (EA) status (non-LEA, LEA), training and performance calibre (Tier 1: recreational, Tier 3: highly trained/national level, Tier 4: elite/international level) as described by McKay et al. [39], and for heavy and light males and females. Participants of a single included study were classified as heavy if their mean body weight was greater or equal to the mean body weight of all studies included in the review (≥ 78.9 kg males, ≥ 62.7 kg females). Participants of a study were classified as light if their mean body weight was less than the average body weight of all included studies (< 78.9 kg males, < 62.7 kg females). In addition, a subgroup analysis was performed for best practice RMR measurement guidelines [45] such as the inclusion of prior-day physical activity abstinence (studies that imposed ≥ 24 h vigorous physical activity abstinence before testing versus those that did not impose such restrictions), adequate analysis to determine RMR (studies that discarded the first 5 min of testing and that utilised a validated RMR method to extract RMR versus those that did not), subject preparation protocols (studies that imposed a pre-test acclimation/rest period immediately prior to testing versus those that did not), the combination of physical activity abstinence and subject preparation protocols (studies that imposed ≥ 24 h vigorous physical activity abstinence and a pre-test acclimation/rest period immediately prior to testing versus those that did not), and subject fasting status (studies that imposed a ≥ 7 h fast, a ≥ 4 h abstinence from caffeine/stimulants, and a ≥ 2.5 h abstinence from nicotine before testing versus studies that imposed two out of three of these criteria versus studies that imposed one out of three of these criteria versus studies that imposed none of these criteria).

For sensitivity analysis, the impact of each study on the combined effect was assessed by omitting one study at a time. Funnel plots were generated to investigate any differences in study effects and publication bias (see Supplementary Document 3 in the ESM). All analyses were completed using Revman software (Revman version 5.3.5; The Cochrane Collaboration) and forest plots produced using GraphPad Prism version 8.0 for Mac (GraphPad Software, San Diego, CA, USA).

### Risk of Bias/Quality Assessment

According to best practice guidelines, risk-of-bias tools should be used in their original unmodified form, and authors should avoid developing their own critical assessment tool to assess risk of bias/study quality [46]. The existing risk of bias tools [47–50] are not appropriate or validated for the study designs included in this systematic review. Therefore, whilst risk of bias and quality assessment of included studies was considered, following best practice in this context [46], no risk of bias or quality assessment was performed.

### Adherence to Best Practice Resting Metabolic Rate Measurement Guidelines

It is acknowledged that methodological differences in RMR measurement itself could have a significant impact on the accuracy of prediction equations. Therefore, during the review process, it was decided to quantify how many criteria for best practice RMR measurement [45] were fulfilled by each study. These guidelines consist of 10 criteria which include physical activity abstinence, fasting adherence, caffeine/stimulant and nicotine abstinence, pre-test acclimation/rest periods, body position, room/environmental conditions, and appropriate test analysis to extract a value for RMR. These data were extracted in a separate table (see Supplementary Document 4 in the ESM) and completed independently by two authors (JERO and KH), with any discrepancies subsequently discussed and resolved.

### Locally Derived Equations

In this review, locally derived equations are defined as those formulated within the population of a particular study. These equations emerge from linear or multiple linear regression analyses that encompass all or a sub-sample of participants involved in the same study.

## Results

### Description of Included Studies

**Fig. 1:**
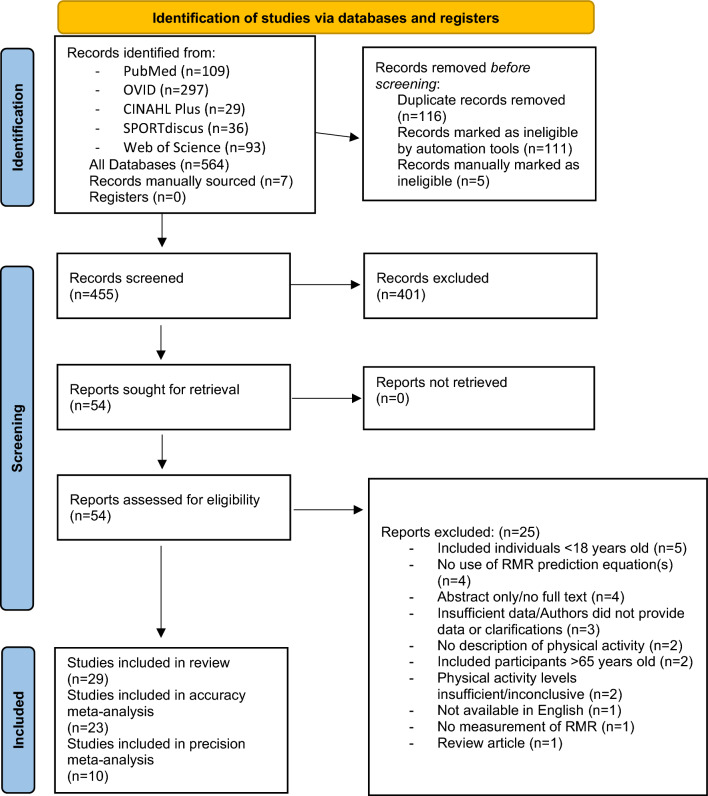
PRISMA flowchart. *RMR* resting metabolic rate

A total of 29 studies were deemed eligible and included in the systematic review (Fig. 1).

An overview of the study characteristics, methods and outcomes of all included studies is provided in Supplementary Document 5 in the ESM. Study publication dates ranged from 1993 to 2021.

#### Athlete Characteristics

Across all included studies, there was a total of 1430 participants (mean [SD]: age 24.2 [7.0] years, height 1.72 [0.10] m, weight 69.3 [15.8] kg). Of these participants, 822 were female (age 24.3 [6.9] years, height 1.67 [0.07] m, weight 62.7 [11.4] kg) and 608 were male (age 24.1 [7.1] years, height 1.78 [0.08] m, weight 78.9 [15.5] kg).

#### Athlete Status

Using the athlete classification framework by McKay et al. [39], the number of athletes per tier (including means and standard deviations for age, height, and weight) included across all studies is shown in Table 1.

**Table 1:** Athlete characteristics and status according to the athlete classification framework by McKay et al. [39] across all included studies

Athlete level	Males (*n*)	Females (*n*)	Total (*n*)	Age (years) mean (SD)	Height (m) mean (SD)	Weight (kg) mean (SD)	References
Tier 1: Recreational	77	409	486	24.2 (7.2)	1.71 (0.10)	69.25 (16.1)	[29, 30, 51, 57, 58]
Tier 2: Trained/developmental	104	37	141	22.9 (4.4)	1.78 (0.08)	70.30 (9.7)	[19, 21]
Tier 3: Highly trained/national level	392	310	702	23.4 (6.1)	1.72 (0.09)	71.10 (15.9)	[3, 4, 10, 12, 14, 22, 23, 25, 27, 32, 33, 52, 53, 56, 60–62]
Tier 4: Elite/international level	35	66	101	24.9 (5.6)	1.69 (0.07)	7.46 (11.4)	[9, 28, 29, 54, 55, 59]

Data are mean (SD)

As described by McKay et al. [39]: Tier 1 = athletes who meet World Health Organization minimum activity guidelines: adults aged 18–64 years old completing at least 150–300 min moderate-intensity activity or 75–150 min of vigorous-intensity activity a week, plus muscle-strengthening activities 2 or more days a week. Tier 2 = athletes who regularly train ~ 3 times per week, identify with a specific sport, and represent/compete in that sport at a local level. Tier 3 = athletes who compete at the national level. Tier 4 = athletes who compete at an international level

#### Sport Type

Athletes of individual studies were classified as endurance (*n* = 5 studies), team sport (*n* = 5 studies), recreational exercisers (*n* = 5 studies), combat (*n* = 2 studies), weightlifting (*n* = 1 study), bodybuilders (*n* = 1 study), and dancers (*n* = 1 study). Eight studies included a variety of athletes from multiple different sporting backgrounds (such as basketball, baseball, track and field, dancing, archery, diving, gymnastics, American football, waterpolo, volleyball, fencing, etc.). Further details on sports included are reported in Supplementary Document 6 in the ESM.

### Equations Included

One hundred different prediction equations from forty-six separate original articles were investigated across all included studies. Studies ranged from comparing the accuracy of 30 different equations [51] to examining the accuracy of a single equation [4, 10, 22, 52–56]. The top five most included equations were the Cunningham (1980) (lean body mass [LBM]) (*n* = 21 studies), the Harris-Benedict (1918) (age, weight, height) (*n* = 21 studies), the Mifflin St. Jeor (1990) (age, weight, height) (*n* = 11 studies), the De Lorenzo (1999) (age, weight, height) (*n* = 8 studies), and the FAO/WHO/UNU (1985) (age, weight) equations and the FAO/WHO/UNU (1985) (age, weight, height) equations (both *n* = 7 studies). Most equations (*n* = 43) were only used in single studies. An overview of all equations that have been developed in athletes, and results regarding mean bias and precision is provided in Table 2. For a complete list of all prediction equations that have been studied in athletes in chronological order, see Supplementary Document 7 in the ESM.

**Table 2:** List of equations developed in athletes and mean bias and precision results reported from studies in athletes

Equation name (year) (components), country	RMR equation	Population equation based on		Bias > 10% (% bias; precision [P] where reported)	Bias < 10% (% bias; precision [P] % where reported)	Bias < 5% (precision [P] % where reported)
		Number (male [M], female [F]), age	Other characteristics described			
Equations developed in athlete populations						
De Lorenzo (1999) (weight, height), Italy [19]	*Males* (*kcal/24* *h*): (9 × wt [kg]) + (11.7 × ht [cm]) − 857	*n* = 51 M, 22.3 ± 3.5 y	Athletes exercising ≥ 3 h/d, 6 d/wk in water polo, judo or karate. Wt, 78.0 ± 11.5 kg; ht, 178.4 ± 7.1 cm; body fat 12.4 ± 4.1%	*Overestimation* Balci (2021)—Female, members of the Turkish National Olympic Team, 17%, (P 42%) [32] *Underestimation* Jagim (2019)—Males, NCAA Div III mixed sports, 12%, (P NR%) [12] Tinsley (2019) – Male, bodybuilders, 13%, (P NR%) [23]	*Overestimation* Jagim (2019)—Females, NCAA Div III mixed sports, 7%, (P NR%) [12] Tinsley (2019)—Female, bodybuilders, 7%, (P NR%) [23] Ten-Haaf (2014)—Females, mix of athletics, endurance, and team sports, 7%, (P 59%) [21] Nichols (2020)—< 25 BMI Females, recreational exercisers, 7%, (P 52%) [51] Nichols (2020)—> 25 BMI Females, recreational exercisers, 6%, (P 57%) [51] Nichols (2020)—< 25 BMI Males, recreational exercisers, 8%, (P 46%) [51]	Balci (2021)—Males, members of the Turkish National Olympic Team (P 40%) [32] Marra (2021)—Males, mix of endurance, combat, and water polo (P 75%) [33] Ten-Haaf (2014)—Males, mix of athletics, endurance, and team sports, (P 77%) [21] Wong (2012)—Males, mix of combat, team, and skilled sports, (P NR%) [61] Nichols (2020)—> 25 BMI Males, recreational exercisers, (P 57%) [51]
Taguchi (2011) (weight), Japan [76]	*Females* (*kcal/24 h*): 26.9 × FFM (kg) + 36	*n* = 93 F; 20.3 ± 1.2 y	*n* = 93 Collegiate female athletes competing in Japan in inter-collegiate games from various sports including track and field, swimming, lacrosse, basketball, judo, rhythmic gymnastics, rowing, cheerleading, badminton, weightlifting. Wt, 57.0 ± 9.2 kg, ht, 162.8 ± 6.4 cm, body fat 20.0 ± 3.9%		*Underestimation* Watson (2019)—Females, NCAA Div II mixed sports, 7%, (P NR%) [25]	
Wong (2012) (weight), Malaysia [61]	*Unisex* (*kcal/24 h*): 669 + (13 × wt [kg]) + (192 × sex [M = 1, F = 0])	*n* = 92 M, *n* = 33 F; 18–31 y	‘Elite’ National Malaysian athletes from 15 sports (combat, racquet, team, skilled, other) training ≥ 6 h/d for at least 1 y. M: wt, 66.1 ± 8.5 kg; ht, 170.6 ± 6.5 cm; body fat 13.7 ± 4.0%, F: wt, 55.4 ± 5.7 kg; ht, 160.7 ± 4.8 cm; body fat, 21.7 ± 4.0%			Wong (2012)—Males and females, mix of combat, team, and skilled sports, (P NR%) [61] Marra (2021)—Males, mix of endurance, combat, and water polo, (P 75%) [33]
Ten-Haaf (2014) (age, weight, height), the Netherlands [21]	*Unisex* (*kcal/24 h*): (11.936 × wt [kg]) + (587.728 × ht [m]) − (8.129 × age [y]) + (191.027 × sex [M = 1, F = 0]) + 29.279	*n* = 53 M, *n* = 37 F; 18–35 y	Dutch recreationally active adults (training 9.1 ± 5.0 h/wk, frequency, 5.0 ± 1.8 times/wk) from ~ 16 sports including athletics, cycling, gymnastics, fitness, rowing/canoeing, team sports, other). All: wt, 52.8–100.3 kg; ht, 161–205 cm, body fat, 1.5–32.2%. F: wt, 52.8–78.3 kg; ht, 161–184 cm, body fat, 11.2–32.2%. M: wt, 62.1–100.3 kg; ht, 163–205 cm, body fat, 1.5–26.3%		*Underestimation* Tinsley (2019)—Male, bodybuilders, 6%, (P NR%) [23]	O’Neill (2022)—Female, rugby, (P 86%) [9] Marra (2021)—Males, mix of endurance, combat, and water polo, (P 75%) [33] Tinsley (2019)—Female, bodybuilders, (P NR%) [23] Ten-Haaf (2014)—Males and females, mix of athletics, endurance, and team sports, (Males: P 85%, Females: P 76%) [21] Van Hooren (2022)—Male and female, professional cyclists (P NR%) [75]** Freire (2023)—Male and female, Brazillian National and Olympic team, (Males: P 72%, Females: P 66%) [74]**
Ten-Haaf (2014) (FFM), the Netherlands [21]	*Unisex* (*kcal/24 h*): (22.771 × FFM [kg]) + 484.264		FFM by air displacement plethysmography (BodPod)			O’Neill (2022)—Female, rugby, (P 86%) [9] Ten-Haaf (2014)—Males and females, mix of athletics, endurance, and team sports, (Males: P 83%, Females: P 72%) [21] Tinsley (2019)—Male and female, bodybuilders, (P NR%) [23]
Koehler DXA-predicted (2016) (brain mass, skeletal muscle mass, adipose tissue mass, bone mass, residual mass), USA, Canada [58]	*Unisex *(*kcal/24 h*): (240 × brain mass [kg]) + (13 × skeletal muscle mass [kg]) + (2.3 × bone mass [kg]) + (4.5 × adipose tissue mass [kg]) + (43 × residual mass [kg])	*n* = 79 F, 18–35 y	All exercising (≥ 2 h/wk), primarily (72%) aerobic (running, cycling, cardio training, swimming, triathlon, aerobics), others in team sports and resistance exercise. *N* = 42 with amenorrhoea (wt, 58.4 ± 1.0 kg; ht, 168.0 ± 1.0 cm; body fat, 21.5 ± 0.9%; *N* = 37 with ovulatory menstrual cycles (wt, 58.2 ± 0.8 kg, ht, 164.0 ± 1.0 cm, body fat, 24.1 ± 0.8%). Modelling of organ tissues based on DXA	*Overestimation* Staal (2018)—Female, ballet, 13%, (P 65%) [3] Strock A (2020)—Females, recreational exercisers, amenorrhoeic group: 11%, (P NR%) [30]	*Overestimation* Staal (2018)—Female, ballet, 7%, (P 45%) [3] Strock A (2020)—Females, Recreational exercisers, subclinical menstrual dysfunction: 6% (P NR%) [30] Koehler (2016)—Female recreational exercisers, amenorrhoeic group, 9%, (P NR%) [58]	Strock A (2020)—Females, recreational exercisers, ovulatory group, (P NR%) [30] Strock B (2020)—Female, recreational exercisers, (P NR%) [57] Koehler (2016)—Female recreational exercisers, ovulatory group, (P NR%) [58]
Joseph (2017) (weight), India [28]	*Males* (*kcal/24 h*): − 164.065 + (0.039 × LBM [kg])	*n* = 30 M; 18–28 y	Indian international competitive weightlifters training ≥ 4 h/d ≥ 5 d/wk, wt, 56.6–127.6 kg; ht 157.5–182.0 cm, body fat 8.4–30.3%. LBM determined by DXA			Joseph (2017)—Male, Indian weightlifters, (P NR%) [28]
Jagim (2019) (weight), USA [22]	*Males* (*kcal/24 h*): 19.46 × wt (kg) + 775.33 *Females* (*kcal/24 h*): 21.10 × wt (kg) + 288.6	*n* = 68 M, *n* = 48 F; M: 20.1 ± 1.5 y, F: 19.4 ± 1.3 y	NCAA Division III athletes. M: football, track (distance), baseball; F: track (sprint, distance, throw), swimming, soccer, tennis) M: wt, 93.7 ± 16.3 kg; ht, 181.8 ± 5.9 cm; body fat, 16.3 ± 8.6% F: wt, 63.4 ± 12.7 kg, ht, 166.5 ± 6.0 cm; body fat, 21.5 ± 6.3%	*Overestimation* O’Neill (2022)—Female, rugby, 10%, (P 44%) [9]		Jagim (2019)—Females and males, NCAA Div III, (P NR%) [22]
Tinsley (2019) (FFM), USA [23]	*Unisex *(*kcal/24 h*): 25.9 × FFM (kg) + 284	*n* = 17 M, *n* = 10 F, 25.9 ± 6.0 y	Subjects who self-identified as bodybuilders, training 5.7 ± 0.9 d/wk. All: wt, 82.9 ± 17.0 kg; ht, 175.6 ± 9.2 cm; body fat, 15.0 ± 4.4%. M: wt, 94.0 ± 9.7 kg; ht, 180.4 ± 7.2 cm; body fat, 12.5 ± 2.7%. F: wt, 63.8 ± 5.7 kg; ht, 167.5 ± 5.7 cm; body fat, 19.2 ± 3.4%. FFM determined by DXA. Note 26% reported anabolic steroid use			Tinsley (2019)—Male and female, bodybuilders, (P NR%) [23] *Leave one out cross-validation performed in same study
Tinsley (2019) (weight), USA [23]	*Unisex* (*kcal/24 h*): 24.8 × wt (kg) + 10					Tinsley (2019)—Male and female, bodybuilders, (P NR%) [23] *Leave one out cross-validation performed in same study
Watson (2019) (age, weight, height), USA [25]	*Females* (*kcal/24 h*): 88.1 + (2.53 × ht [cm]) + (8.42 × wt [kg]) + (19.46 × age [y])	*n* = 66 F, 19.7 ± 1.1 y	NCAA Division II athletes (ice hockey, volleyball, basketball, softball, golf, field hockey, gymnastics, tennis, other). Wt, 67.3 ± 8.9 kg; ht, 169 ± 9 cm; body fat, 26.2 ± 4.9%			O’Neill (2022)—Female, rugby, (P 67%) [9] Watson (2019)—Cross-validated (paired t-test only, bias not reported) in subsample in same study (*n* = 22) [25]
Watson (2019) (age, FFM, height), USA [25]	*Females* (*kcal/24 h*): 120.81 + (4.88 × ht [cm]) + (8.24 × FFM [kg]) + (5.71 × age [y])		FFM determined by 3-site skinfolds		*Underestimation* O’Neill (2022)—Female, rugby, 8%, (P 53%)	Watson (2019)—Cross-validated (paired t-test only, bias not reported) in subsample in same study (*n* = 22) [25]
MacKenzie-Shalders (2020) (LBM, FM), Australia [60]	*Males* (*kcal/24 h*): 25.49 × LBM (kg) + 7.62 × FM (kg) + 162.93	*n* = 18 M, 20.2 ± 1.7 y	Developing elite rugby union athletes. Wt, 101.2 ± 14.5 kg; ht, 184.0 ± 8.4 cm, LBM, 81.3 ± 8.0 kg. LBM, FM by DXA			Mackenzie-Shalders (2020)—Male, elite rugby, (P NR%) [60]
MacKenzie-Shalders (2020) (LBM), Australia [60]	*Males* (*kcal/24 h*): 29.71 × LBM (kg) – 24.562					Mackenzie-Shalders (2020)—Male, elite rugby, 6%, (P NR%) [60]
MacKenzie-Shalders (2020) (weight), Australia [60]	*Males* (*kcal/24 h*): 15.95 × wt (kg) + 775.32					Mackenzie-Shalders (2020)—Male, elite rugby, 6%, (P NR%) [60]
O’Neill (2022) (age, weight, height), Ireland [9]	*Females* (*kcal/24 h*): 1501 − (6.858 × age [y]) − (2.946 × ht [cm]) + (11.21 × wt [kg])	*n* = 36 F, 18–35 y	Elite and sub-elite rugby players. Wt, 59.4–99.9 kg; ht, 157.0–182.2 cm; FFM, 43–63 kg; body fat, 15–41%			O’Neill (2022)—Female, Rugby, (P 83%) [9]
O’Neill (2022) (FFM), Ireland [9]	*Females* (*kcal/24 h*): 18.91 × FFM (kg) + 649.6		FFM by air displacement plethysmography			O’Neill (2022)—Female, Rugby, (P 83%) [9]
Marra (2021) (age, weight), Italy [33]	*Males* (*kcal/24 h*): (17.2 × wt [kg]) − (5.95 × age [y]) + 748	*n* = 75 M, 26.9 ± 9.1 y	Elite athletes (previously competed at regional/national level), training ≥ 24 h/wk swimming, cycling, running, karate, water polo, ballet, boxing. Wt, 71.3 ± 10.9 kg; Ht, 177 ± 7 cm			Marra (2021)—Males, Mix of endurance, combat, and water polo, (P 82%) [33] *Cross-validated in same study, similar population (*n* = 51)
Marra (2021) (weight, BIA-derived phase angle), Italy [33]	*Males *(*kcal/24 h*): (16.3 × wt [kg]) + (95.4 × PhA [degrees]) − 93					Marra (2021)—Males, Mix of endurance, combat, and water polo, (P 92%) [33] *Cross-validated in same study, similar population (*n* = 51)
Equations since review search completed						
Freire (2022) (sex, age, weight, height), Brazil [74]	*Unisex* (*kcal/24 h*): 729.50 + (175.84 × sex [M = 0, F = 1]) − (7.23 × age [y]) + (15.87 × wt [kg]) + (1.08 × ht [cm])	*n* = 34 M, *n* = 37 F; 24.5–26.4 y	‘High level’ athletes (majority world championship and/or Olympic) from 21 sports including endurance, team, racquet, combat, skilled sports, weightlifting and wrestling; wt, 72.9–82.2 kg; ht, 173.6–178.7 cm			Freire (2023)—Male and females, Brazillian National and Olympic Team, (P 61%) *Cross-validated in same study, similar population (*n* = 31) [74]
Freire (2022) (sex, age, weight, height), Brazil [74]	*Unisex* (*kcal/24 h*): − 2688.12 + (521.08 × sex [M = 0, F = 1]) + (42.86 × age [y]) + (18.98 × wt [kg]) + (16.76 × ht [cm]) + (85.47 × Meso) + (140.54 × Endo) − (8.24 × (wt [kg] × sex [M = 0, F = 1])) + (1.53 × (wt [kg] × Endo)) − (0.65 (wt [kg] × age [y]))					Freire (2023)—Male and females, Brazillian National and Olympic Team, (P 61%) *Cross-validated in same study, similar population (*n* = 31) [74]
Van Hooren (2023) (sex, weight), Netherlands [75]	*Unisex* (*MJ/24 h*): 0.963 − (0.186 × sex [M = 0, F = 1]) + (0.106 × wt [kg]) *Males only* (*MJ/24 h*): 0.767 + (0.106 × wt [kg])	*n* = 21 M, *n* = 4 F; 27.0 ± 4.0 y	Professional cyclists from UCI World Tour Team, Wt, 66.8 ± 7.5 kg; Ht, 180.6 ± 6.8 cm			Van Hooren (2023)—Male professional cyclists, (P NR%) [75]**

*BMI* body mass index, *BIA* bioelectrical impedance, *Endo* endomorphy, *F* female, *FFM* fat-free mass, *FM* fat mass, *ht* height, *LBM* lean body mass, *Meso* mesomorph, *M* male, *N/a* not applicable, *NR* not reported, *P* precision, *Pha* phase angle, *RMR* resting metabolic rate, *wt* weight

**Study published since search completed, not included in narrative synthesis of systematic review or meta-analysis

### Narrative Synthesis

Despite the athlete populations of studies included in the meta-analysis being heterogenous, five similar athlete demographic groups were identified for the purpose of grouping studies for a narrative synthesis (recreational athletes, endurance, rugby, mixed sports, and other).

#### Recreational Athletes

Five studies classified participants as recreational athletes (resistance, aerobic, and concurrent) [29, 30, 51, 57, 58]. In the original study in which the Koehler DXA (2016) (sum of organelle masses) equation was developed, it was the only equation examined and found to over-predict RMR by 4% in female recreational athletes [58]. For two other studies, the Koehler DXA (2016) (sum of organelle masses) was found to be most accurate (overestimate 7% [30], accurate within 1% [57]) compared with three other equations in females [30, 57]. Elsewhere in female athletes, Mackay et al. [29] found the Mifflin St. Jeor (1990) (age, weight, height) to be the most accurate out of three equations examined; however, RMR was overestimated by ~ 15%. In recreationally active males and females of varying BMI from Trinidad and Tobago, 30 equations were studied with several equations found to be the most accurate (range: within 1–2%) [51]. The most accurate equation for females with a BMI < 25 kg/m^2^ was Johnstone (2006) (fat-free mass [FFM], fat mass [FM], age) and for BMI $\ge$ 25 kg/m^2^ was Müller (MJ/d) (2004) (FFM, FM); and the most accurate equation for males with a BMI < 25 kg/m^2^ was Owen (1988) (weight) and for BMI $\ge$ 25 kg/m^2^ was Livingston and Kohlstadt (2005) (age, weight) [51].

#### Endurance

Five studies included endurance athletes. One study examined the accuracy of the Harris-Benedict equation only, finding it underestimated RMR by ~ 6% in male runners, tri- and bi-athletes [56]. Two studies examined the Cunningham (1980) (LBM) only, finding it overestimated RMR by ~ 10% in male cyclists [4] and underestimated RMR by ~ 16% in female elite endurance athletes (sport/s unspecified) [53]. Sjodin et al. [59] examined the accuracy of two equations—FAO/WHO/UNU (1985) (age, weight, height) and Westerterp (1995) (FFM, FM)—and found the Westerterp equation to be more accurate, but still to underestimate RMR by 12% in eight male and female cross-country skiers. Devrim-Lanpir et al. assessed both accuracy and precision of nine equations in 30 male and female triathletes and ultra-marathoners, finding most under-estimated RMR and the Mifflin-St. Jeor equation to be most accurate (underestimating by ~ 5%) [27]. However, this equation was only able to predict ~ 50% to within 10% of measured values [27].

#### Rugby

Three studies included rugby athletes [9, 10, 60]. In a study of six male rugby league athletes, only one equation (Cunningham [1980] [LBM]) was used [10]. It was found to overestimate RMR by 17%, with only one athlete estimated to within 10% of measured RMR [10]. In the two other studies, locally developed equations were reported to be the most accurate (to < 1%) in rugby league males compared with three previously published equations [60], and in rugby union females compared with seven previously published equations [9], respectively. The Ten-Haaf (2014) (FFM) equation was found to be most precise (predicting 31 out of 36 [82%] within 10%) in the latter study [9].

#### Mixed Sports

Eight studies included populations that were not specific to one sport and contained mixed disciplines (see Supplementary Document 6 in the ESM) [12, 19, 21, 22, 25, 32, 33, 61]. One study examined the accuracy of a locally developed equation finding it to be accurate to within < 2% in athletes from a range of NCAA sports [22]. All other studies compared measured values to a number of other equations ranging from five to twelve equations [12, 19, 21, 25, 32, 33, 61], with five of these studies also developing locally derived equations [19, 21, 25, 33, 61]. In all cases, those locally derived were found to be the most accurate (within < 1%) [19, 21, 25, 33, 61]. In two of the studies wherein precision was also reported, locally developed equations were also found to be the most precise [21, 33]. In the two other studies, the Mifflin St. Jeor (1990) (age, weight, height) equation was found to be the most accurate (< 1%) and precise (59%, 29/49) in male and female members of the Turkish Olympic National team [32], and the Cunningham (1980) (LBM) was found to be the most accurate both in male and female NCAA Division III athletes from mixed disciplines (within ~ 4%) [12].

#### Other

Eight studies included athletes that did not fit a specific grouping: ballet dancers (*n* = 1) [3], bodybuilders (*n* = 1) [23], karate (*n* = 1) [62], rowers and canoeists (*n* = 1) [14], soccer (*n* = 2) [52, 55], taekwondo (*n* = 1) [54], and weightlifters (*n* = 1) [28]. Three studies examined the accuracy of one equation only; the levels of recommended assumption of nutrients for the Italian population (LARN) (1996) equation underestimated RMR by ~ 13% in female soccer players [52], the Cunningham (1980) (LBM) equation, in contrast, overestimated RMR by ~ 6% in female soccer players [55], and the Cunningham (1980) (LBM) equation was accurate to < 1% in a single athlete case study of a male taekwondo athlete [54]. Two other studies found the Cunningham (1980) (LBM) equation to be the most accurate in male and female rowers and canoeists (~ 15% under-estimation in males, ~ 10% over-estimation in females) [14] and female karate (~ 8% under-estimation) [62], compared with two and three other equations respectively. In male and female ballet dancers, the Koehler DXA (2016) (sum of organelle masses) was found to be the most accurate (within ~ 10%) out of three equations, with the Harris-Benedict (1918) (age, weight, height) most precise (26/40; 65%) [3]. In male and female bodybuilders, a locally developed equation was found to be the most accurate (within < 1%) when compared with 11 other equations [23]. In males from the Indian national weightlifting team, the FAO/WHO/UNU (1985) (age, weight, height) was reported to be the most accurate and precise out of eight equations but its performance was still very poor (accuracy: underestimating by ~ 18%, precision: 11/30 [37%]) [28].

### Meta-analysis

Six articles could not be synthesised by meta-analysis for the following reasons: means and/or SDs for predicted or measured RMR were not provided (*n* = 3) [19, 51, 56], RMR prediction equation used by less than three separate studies (*n* = 2) [22, 52] and study sample size of *n* = 1 [54].

#### Meta-analysis Accuracy—Description of Included Studies

Twenty-three studies were included in the meta-analysis for prediction equation accuracy, involving 1058 participants (mean [SD]: age 23.4 [5.8] years, height 1.72 [0.09] m, weight 66.4 [13.5] kg). Of these participants, 671 were female (age 23.4 [5.2] years, height 1.67 [0.07] m, weight 61.0 [8.6] kg) and 387 were male (age 23.9 [6.7] years, height 1.78 [0.09] m, weight 76.6 [14.4] kg). A total of 1206 comparisons were made between predicted RMR by equation and measured RMR via IC.

#### Meta-analysis Accuracy—Results

**Fig. 2:**
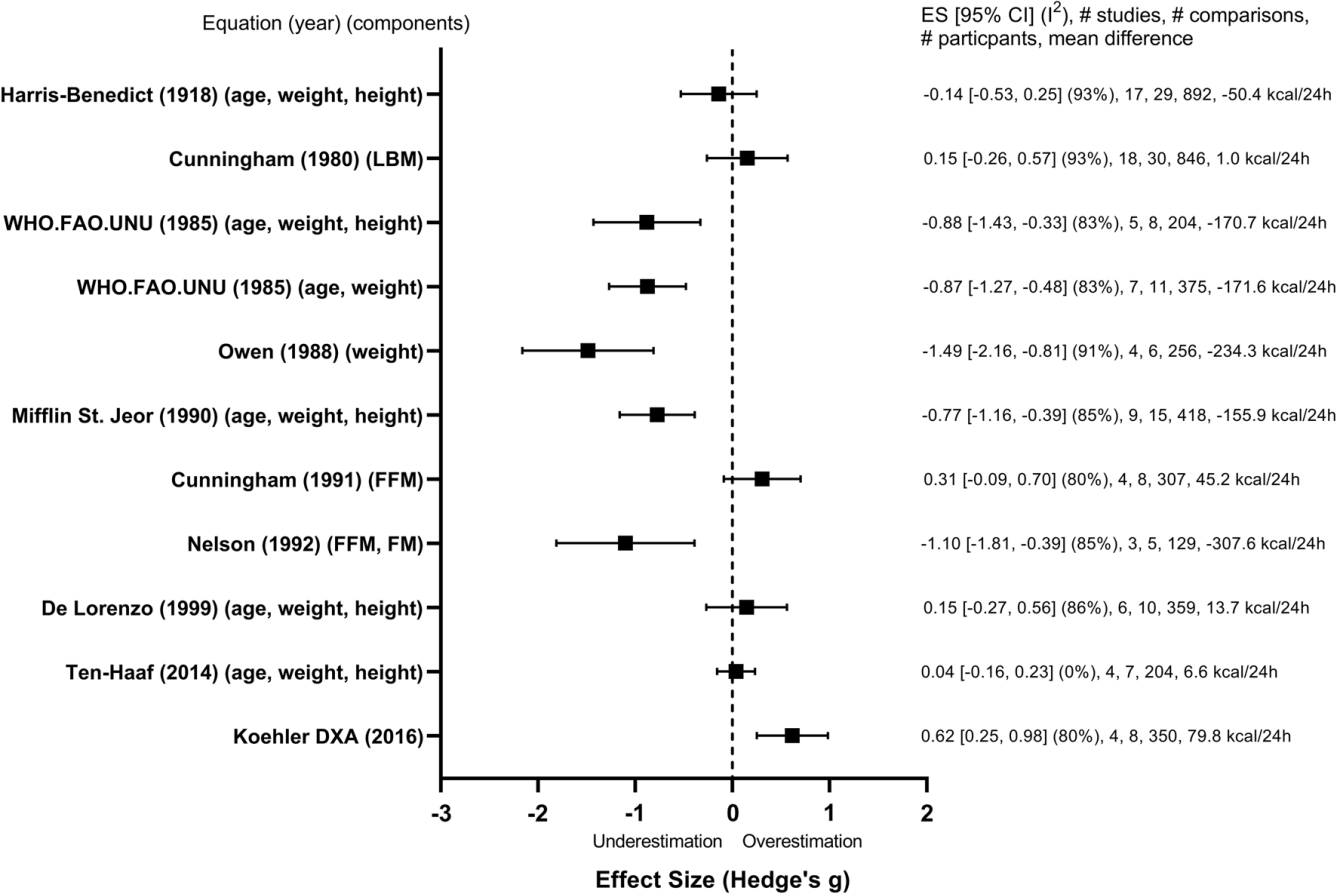
Forest plot containing all equations analysed in meta-analysis listed in chronological order. *CI* confidence interval, *ES* effect size, *FFM* fat free mass, *FM* fat mass, *LBM* lean body mass

A forest plot of results for individual equations across all studies is shown in Fig. 2. Tabulated results of the meta-analysis evaluating accuracy are shown in Supplementary Document 8 (see ESM).

Examining the equations individually, the accuracy of predicted compared with measured values did not differ significantly for five equations—the Cunningham (1980) (LBM), the Harris-Benedict (1918) (age, weight, height), the Cunningham (1991) (FFM), the De Lorenzo (1999) (age, weight, height), (the Ten-Haaf (2014) (age, weight, height)—whereas all others significantly underestimated or overestimated RMR (*p* < 0.05) (Fig. 2). The Ten-Haaf (2014) (age, weight, height) showed the smallest effect size (0.04), indicating the greatest accuracy.

#### Meta-analysis Accuracy—Heterogeneity Summary

Significantly large heterogeneity was observed for most equations included in the meta-analysis (range: *I*^2^: 80–93%) [7, 8, 16–19, 26, 58, 63]. For example, although the Cunningham (1980) (LBM) and the Harris-Benedict (1918) (age, weight, height) equations showed trivial ESs (ES = 0.15 [95% CI − 0.26 to 0.57], 18 studies, 30 comparisons, 846 participants and ES = − 0.14 [95% CI − 0.52 to 0.25], 17 studies, 29 comparisons, 892 participants, respectively), heterogeneity was considerable with many comparisons in each equation under- and overpredicting RMR (both equations *I*^2^ = 93%; *p* < 0.0001). The Ten-Haaf (2014) (age, weight, height) equation showed a trivial ES (ES = 0.04 [95% CI − 0.16 to 0.23], 4 studies, 7 comparisons, 204 participants) and no heterogeneity (*p* = 0.48, *I*^2^ = 0%).

#### Meta-analysis Accuracy—Subgroup Analysis

Results of the accuracy meta-analysis and accuracy subgroup analysis for each equation separately are shown in Supplementary Document 9 (see ESM).

##### Sex

A significant difference was observed between male and female subgroups for the De Lorenzo (1999) (age, weight, height) equation (*I*^2^ = 95.4%; *p* < 0.0001) and a trend for subgroup differences for the Harris-Benedict (1918) (age, weight, height) (*I*^2^ = 72.7%; *p* = 0.06) equation was observed. For the De Lorenzo (1999) (age, weight height) equation, a small and large ES was observed for males and females, respectively (males: ES = − 0.31 [95% CI − 0.73 to 0.11], *I*^2^ = 81%, *p* < 0.0001, six studies, six comparisons, 266 participants; females: ES = 0.93 [95% CI 0.63 to 1.23], *I*^2^ = 0%, *p* = 0.91, four studies, four comparisons, 93 participants). For the Harris-Benedict (1918) (age, weight, height) equation, a moderate and trivial ES was observed for males and females, respectively (males: ES = − 0.53 [95% CI − 0.93 to − 0.13], *I*^2^ = 84%, *p* < 0.0001, 11 studies, 11 comparisons, 357 participants; females: ES = 0.11 [95% CI − 0.40 to 0.63], *I*^2^ = 93%, *p* < 0.0001, 14 studies, 18 comparisons, 535 participants). There were no significant differences observed between male and female subgroups for any other equations analysed [8, 16, 17, 21, 26].

##### Body Composition Measurement Method

The Cunningham (1980) (LBM) equation was the only equation that satisfied criteria for subgroup analysis. When subgrouping studies that used DXA, BIA, and Bodpod to determine body composition, there were no significant subgroup differences observed.

##### Energy Status

Only the Cunningham (1980) (LBM) equation satisfied criteria for subgroup analysis by energy status of participants, with no significant subgroup differences observed.

##### Athlete Status

Only the Cunningham (1980) (LBM) and the Harris-Benedict (1918) (age, weight, height) equations satisfied criteria for subgroup analysis. No subgroup differences were observed for the Cunningham (1980) (LBM) equation. However, significant subgroup differences were observed for the Harris-Benedict (1918) (age, weight, height) equation with differences observed between Tier 1 and Tier 3, and between Tier 1 and Tier 4 athletes (*p* < 0.0001 for both). A significant large ES for overestimation of RMR was observed for Tier 1: recreationally active female athletes (studies included in this subgroup were female only) and a significant moderate ES for underestimation was observed for Tier 3: highly trained/national level and Tier 4: elite/international level athletes (Tier 1: ES = 1.50 [95% CI 1.26 to 1.73], *I*^2^ = 20%, *p* = 0.29, three studies, four comparisons, 243 participants; Tier 3: ES = − 0.42 [95% CI − 0.82 to − 0.02], *I*^2^ = 86%, *p* < 0.0001, 11 studies, 17 comparisons, 444 participants; Tier 4: ES = − 0.38 [95% CI − 1.00 to 0.25], *I*^2^ = 81%, *p* = 0.0003, three studies, five comparisons, 115 participants).

##### Heavy and Light Males and Females

The Cunningham (1980) (LBM) and the Harris-Benedict (1918) (age, weight, height) equations satisfied criteria for subgroup analysis in both males and females, and the Mifflin St. Jeor (1918) (age, weight, height) equation satisfied criteria for subgroup analysis in females only. A trend for subgroup differences between heavy and light males for the Harris-Benedict (1918) (age, weight, height) equation was observed (*I*^2^ = 68.8%; *p* = 0.07). A small and large ES was observed in males on average < 78.9 kg and > 78.9 kg, respectively (< 78.9 kg: ES = − 0.33 [95% CI − 0.85 to 0.20], *I*^2^ = 88%, *p* < 0.0001, seven studies, seven comparisons, 286 participants; > 78.9 kg: ES = − 0.91 [95% CI − 1.25 to − 0.56], *I*^2^ = 0%, *p* = 0.68, four studies, four comparisons, 71 participants). No significant differences were observed between subgroups for any other equation and/or subgrouping.

##### $\ge$24-Hour Physical Activity Abstinence

The Cunningham (1980) (LBM), the Harris-Benedict (1918) (age, weight, height), the Mifflin St. Jeor (1918) (age, weight, height), and the De Lorenzo (1999) (age, weight, height) equations satisfied criteria for subgroup analysis. No significant subgroup differences were observed for any equation.

##### Discard Period, Steady State, and Validated Extraction Method

The Cunningham (1980) (LBM), the Harris-Benedict (1918) (age, weight, height), the Mifflin St. Jeor (1918) (age, weight, height), and FAO/WHO/UNU (1985) (age, weight) equations satisfied criteria for subgroup analysis. No significant differences were observed; however, a trend for subgroup differences was observed in the Harris-Benedict (1918) (age, weight, height) equation (*p* = 0.06, *I*^2^ = 72%). A small ES for overestimation of RMR was observed in the subgroup when an RMR discard period, use of a steady-state model, and a validated RMR extraction method were omitted, and a moderate ES for underestimation of RMR was observed in the subgroup where these methodological criteria were present (Present: ES = − 0.47 [95% CI − 0.83 to − 0.12], *I*^2^ = 81%, *p* < 0.0001, eight studies, 15 comparisons, 400 participants; Omitted: ES = 0.25 [95% CI − 0.41 to − 0.91], *I*^2^ = 95%, *p* < 0.0001, nine studies, 14 comparisons, 492 participants). No significant differences between subgroups were observed for any other equation.

##### Pre-test Rest Versus No Pre-test Rest

The Cunningham (1980) (LBM), the Harris-Benedict (1918) (age, weight, height), the Mifflin St. Jeor (1918) (age, weight, height), the De Lorenzo (1999) (age, weight, height), and the FAO/WHO/UNU (1985) (age, weight) equations satisfied criteria for subgroup analysis. No significant differences were observed between subgroups for any equation.

##### Fasting Status and Preparation

Only the Cunningham (1980) (LBM) and the Harris-Benedict (1918) (age, weight, height) equations satisfied criteria for subgroup analysis. For the Cunningham (1980) (LBM) equation, subgroup differences were observed between studies that implemented all three preparation procedures (fast, caffeine abstinence, and nicotine abstinence) versus those that implemented only one (1/3 vs 3/3: *p* < 0.0001, *I*^2^ = 95%). For the Harris-Benedict (1918) (age, weight, height) equation, subgroup differences were observed between studies that implemented only one preparation procedure versus those that implemented two and three preparation procedures (1/3 vs 2/3: *p* = 0.003, *I*^2^ = 89%); (1/3 vs 3/3: *p* < 0.0001, *I*^2^ = 95%). For subgroups where all three preparation procedures were implemented, significant overestimation was observed in both the Cunningham (1980) (LBM) equation (ES = 0.97 [95% CI 0.56 to 1.37], *I*^2^ = 78%, *p* = 0.0001, four studies, seven comparisons, 314 participants) and the Harris-Benedict (1918) (age, weight, height) equation (ES = 0.71 [95% CI 0.07 to 1.35], *I*^2^ = 93%, *p* < 0.0001, five studies, eight comparisons, 365 participants). For subgroups where two of three preparation procedures were implemented, no significant magnitudes of effect were observed in either equation. For subgroups where one of three preparation procedures were implemented, significant ES was observed for the Harris-Benedict (1918) (age, weight, height) equation only (ES = − 0.92 [95% CI − 1.23 to − 0.61], *I*^2^ = 32%, *p* = 0.18, four studies, seven comparisons, 134 participants).

#### Meta-analysis Precision—Description of Included Studies

Ten studies were included in the meta-analysis for prediction equation precision [3, 9, 10, 21, 27, 28, 32, 33, 51, 54], involving a total of 497 participants (mean [SD]: age 27.3 [9.4] years, height 1.72 [0.01] m, weight 72.8 [10.5] kg). Of these participants, 225 were female (age 28.4 [9.6] years, height 1.66 [0.07] m, weight 67.9 [15.4] kg) and 272 were male (age 26.1 [9.0] years, height 1.76 [0.09] m, weight 75.7 [12.8] kg).

#### Meta-analysis Precision—Overall Result

Tabulated results of the meta-analysis evaluating precision for individual equations is shown in Table 3. Overall, the Ten-Haaf (2014) (age, weight, height) equation was found to be the most precise equation predicting 80.2% of participants to be within ± 10% of measured values with all other included equations ranging from 40.7 to 63.7%. Subgroup analysis was only possible for precision for six equations for sex and athlete status, with precision being poor across all subgroups (range 19–57%) (Table 3).

**Table 3:** Precision and subgroup analysis of RMR calculated by RMR prediction equation versus RMR measured via indirect calorimetry

Overall/subgroup name Name of prediction equation	Subgroup	Number of studies (refs)	Pooled number of participants	Precision (%) (weighted mean)
Overall				
Cunningham (1980) (LBM)		7 [3, 9, 10, 21, 27, 28, 54]	233	54.08
De Lorenzo (1999) (weight, height)		3 [21, 32, 33]	190	63.69
Harris-Benedict (1918) (age, weight, height)		8 [3, 9, 21, 27, 28, 32, 33, 51]	490	53.67
Mifflin St. Jeor (1990) (age, weight, height)		6 [21, 27, 28, 32, 33, 51]	414	52.17
Mifflin St. Jeor (1990) (FFM)		3 [21, 32, 51]	303	44.88
Owen (1988) (weight)		4 [21, 32, 33, 51]	354	41.24
Ten-Haaf (2014) (age, weight, height)		3 [9, 21, 33]	177	80.22
WHO/FAO/UNU (1985) (age, weight, height)		4 [21, 27, 33, 51]	335	51.64
WHO/FAO/UNU (1985) (age, weight)		3 [21, 27, 28]	150	40.67
Sex				
Cunningham (1980) (LBM)	Males	6 [3, 10, 21, 27, 28, 54]	125	51.20
	Females	4 [3, 9, 21, 27]	108	57.41
Harris-Benedict (1918) (age, weight, height)	Males	7 [3, 21, 27, 28, 32, 33, 51]	271	57.20
	Females	6 [3, 9, 21, 27, 32, 51]	219	49.31
Mifflin St. Jeor (1990) (age, weight, height)	Males	6 [21, 27, 28, 32, 33, 51]	251	49.80
	Females	4 [21, 27, 32, 51]	163	55.83
Mifflin St. Jeor (1990) (FFM)	Males	3 [21, 32, 51]	155	49.03
	Females	3 [21, 32, 51]	148	40.54
Owen (1988) (weight)	Males	4 [21, 32, 33, 51]	206	46.11
	Females	3 [21, 32, 51]	148	35.81
WHO/FAO/UNU (1985) (age, weight, height)	Males	4 [21, 27, 33, 51]	196	53.57
	Females	3 [21, 27, 51]	139	48.92
Athlete status				
Cunningham (1980) (LBM)	Tier 3	3 [3, 10, 27]	76	19.74
	Tier 4	3 [9, 28, 54]	67	55.22

*FFM* fat free mass, *LBM* lean body mass, *RMR* resting metabolic rate

### Adherence to Best Practice Resting Metabolic Rate Measurement Guidelines

An overview of the adherence of each study to the Fullmer et al. [45] best practice guidelines is shown in Supplementary Document 10 (see ESM). In summary, of the 29 included studies in this review, the average number of criteria satisfied [mean (SD, range)] was 5 (2, 2–9) out of 10 criteria.

## Discussion

Given the widespread use of RMR prediction equations in athletes, the present systematic review and meta-analysis aimed to investigate (i) the most accurate and (ii) the most precise equations for use in athletes, and (iii) whether differences exist based on factors such as athlete or methodological characteristics. Overall, it is evident that a variety of equations are being used in athletes with 100 different equations used across the included studies.

### Accuracy

Regarding accuracy, several studies assessed and/or used only one equation to predict RMR, making it difficult to infer their overall accuracy and precision. However, narrative synthesis revealed that in studies in which multiple equations were evaluated, no single equation consistently performed better than any other. Despite this, the most consistently top performing equations were locally derived equations. In all cases where a population-derived equation was developed and compared with other equations (*n* = 8 studies), the derived equations were always the most accurate (within ± 1% of measured RMR). This is no surprise as these prediction equations were derived from subject characteristics such as age, weight, height, and sex from all participants in their study. Using regression analysis, a relationship is then established between these characteristics and measured RMR. Unless the subject characteristics of the athlete whose predicted RMR is required are very similar to the subject characteristics of the population in which the equation was derived, there is a high probability that the estimation of RMR will be inaccurate.

Meta-analysis for accuracy identified the Ten-Haaf (2014) (age, weight, height) equation as the best performing, showing the smallest ES and 95% CIs and no heterogeneity. This is most likely a result of the physical characteristics of the athletes within the included studies (e.g., age, height, weight) being similar to the population in which the Ten-Haaf equation was derived. In the one comparison where RMR tended towards underestimation of RMR using the Ten-Haaf equation [23], the athletes in this study were much heavier than the individuals from which the Ten-Haaf equation was derived.

### Precision

Precision was evaluated by only ten of all studies included in this review, with only two of these studies assessing precision of locally derived equations [9, 21]. In both studies, locally derived equations performed exceptionally well (83–93%), with one study’s locally derived equation only being slightly outperformed by the Ten-Haaf (2014) (FFM) equation and identical in precision to the Ten-Haaf (2014) (age, weight, height) equation [9]. It must be noted that in this study, the Ten-Haaf (2014) (age, weight height) equation was also found to be highly accurate, predicting RMR within 1% [9]. Although some equations in other studies performed somewhat well, none exceeded the performance of the locally derived equations and the Ten-Haaf (2014) (age, weight, height) equation.

Similar to accuracy, meta-analysis for precision revealed the best performing equation to be the Ten-Haaf (2014) (age, weight, height), predicting 80% of participants within ± 10% of measured RMR. Once again, this may be explained by participants having similar physical characteristics to the original population in which the equation was derived. Indeed, one of the studies that found the Ten-Haaf (2014) (age, weight, height) equation to be the most precise was the original study proposing the equation [21].

### Equations that Performed Poorly

In addition to examining the equations that performed best, it is worth noting that there were some equations that consistently underperformed (for accuracy and precision) in athlete populations and should be avoided. These equations include the Mifflin St. Jeor (1990) (age, weight, height), the Owen (1988) (weight), the FAO/WHO/UNU (1985) (age, weight, height), the FAO/WHO/UNU (1985) (age, weight), and the Nelson (1992) (FFM, FM). Although no one specific reason exists as to why these equations consistently underperformed, it is hypothesised that the physical characteristics of the athletes in the studies included did not match the physical characteristics of the populations in which the equations were derived.

### Sex Differences

Significant heterogeneity was observed across all equations (except Ten-Haaf [2014] [age, height, weight]) included in the meta-analysis, which we attempted to explore when possible through subgroup analysis. Subgroup differences were found for sex for both the De Lorenzo (1999) (age, weight, height) and the Harris-Benedict (1918) (age, weight, height) equations. A large overestimation of RMR predicted by the De Lorenzo (1999) equation was observed in female-only comparisons. This could be expected, as the De Lorenzo equation was based on 51 males who on average were heavier and larger [19]. Interestingly, in males who closely matched the physical characteristics of those in the original study, trivial to small effect sizes were observed [21, 32, 33, 61], whereas a large underestimation was observed in the two studies in which males weighed on average ~ 17 kg heavier than those in the original De Lorenzo study [12, 23]. In the case of the Harris-Benedict (1918) equation, RMR was underestimated in the majority of male-only comparison groups, except for one (involving a group of Danish Royal ballet dancers) where it was overestimated [3]. In contrast, for females there was no overall difference between predicted and measured RMR, although heterogeneity remained high. This suggests that sex should be considered when using these equations in practice, whereas for the other equations analysed (FAO, Owen, Mifflin St. Jeor, Cunningham, Ten-Haaf), sex does not appear to influence accuracy.

### Athlete Status

It is also possible that some equations may be more appropriate for athletes of a specific athlete status. We explored this using a recent classification framework [39] for the Harris-Benedict (1918) (age, weight, height) and the Cunningham (1980) (LBM) equations where sufficient data allowed. The trend for underestimation of RMR using the Harris-Benedict equation observed in Tier 3 and Tier 4 athletes may be explained by the fact that the equation was developed in a non-athlete population and does not include the measurement of FFM [7]. However, RMR was still significantly overestimated in Tier 1 recreational athletes. With possible differences in body composition between these groups, it is difficult to infer whether the variable performance of the Harris-Benedict (1918) (age, weight, height) equation is due to differences in competitive status or body composition alone. Nevertheless, the findings suggest athlete status may influence the accuracy of the equation. Interestingly, the Cunningham equation did not perform any differently in those of differing athlete status, suggesting athlete status is not a key factor to consider when using this equation in practice.

### Body Weight

As previously highlighted, the performance of an equation can be considerably influenced by the physical characteristics of an individual in relation to those of the population from which the equation was derived. It is conceivable that equations formulated based on general population metrics may yield inadequate results when applied to athletes, who typically possess significantly larger statures (for instance, bodybuilders or rugby players). To assess whether the accuracy of equations may differ in heavier versus lighter athletes, studies were split by those with a mean body weight greater or equal to the mean body weight of all studies included in this review (≥ 78.9 kg males, ≥ 62.7 kg females) versus those below the mean.

Although not statistically significant, a tendency toward subgroup differences was observed in males with the Harris-Benedict (1918) equation. While the performance of the equation was inconclusive in lighter males, there was a consistent trend towards underestimation in heavier males. On closer examination, studies in which this underestimation was prevalent included those featuring bodybuilders, rugby players, American footballers, baseball players, and heavyweight rowers. The average mean weight within these studies ranged from 93 to > 100 kg [12, 14, 23, 60]. Similarly, although not possible to perform meta-analysis, in the two studies that showed the De Lorenzo (1999) equation to underestimate RMR in males, mean body weight was higher. While further studies are necessary to make a definitive statement that the Harris-Benedict (1918) equation (and potentially other equations) tend to underestimate RMR at higher body weights, the trends observed should be considered. Hence, the application of the Harris-Benedict (1918) and De Lorenzo (1999) equations to heavier male athletes should be approached with caution.

### Energy Availability Status, Body Composition Method and Prior Exercise Avoidance

Other characteristics were also explored to try and identify factors that may contribute to heterogeneity in results, including the mean body weight or energy availability status of included athletes, method of body composition measurement or how long participants were required to avoid exercise prior to RMR measurement. However, none of these factors showed significant differences. For some of these analyses, however, it should be noted that the number of equations that satisfied criteria for inclusion were limited and, therefore, further studies are required to explore the influence of these factors on the accuracy of different equations. For example, the body composition variables and measurement technique used to develop equations should be considered when interpreting the accuracy of equations, given the wide range of methods used in athletes. A common issue is the interchangeable use of LBM versus FFM in the Cunningham (1980) equation. This may influence the accuracy of calculations and warrants further study and consideration when applying equations in practice [64].

With regard to athlete energy status, the ratio of measured RMR to predicted RMR (RMR ratio) is being increasingly used as a proxy indicator of energy availability [3–6, 15, 53]. An RMR ratio of < 0.9 is considered indicative of low energy availability, meaning a difference between measured and predicted RMR of only 10% could be interpreted as the suppression of RMR [15]. The Cunningham (1980) (LBM) equation has been used to determine RMR ratio as a proxy indicator for LEA in numerous studies [3–6, 15, 53]. However, as evident in the results of this meta-analysis, observed ES of individual studies for the Cunningham (1980) (LBM) equation ranged from large overestimation (ES = 3.08) to large underestimation (ES = − 2.11), with 13 out of 30 comparisons having ESs greater or less than 1 and − 1, respectively. Furthermore, ESs varied widely between equations, highlighting that classification of suppressed RMR will depend on the equation used. Unless a specific RMR equation has been shown to accurately predict an individual’s RMR at different body weights (within 10%), it appears ill-advised to use an arbitrary or even ‘commonly used’ equation to detect the suppression of RMR from a single measurement. A more suitable use may be in longitudinal monitoring, and interpretation of directly measured RMR and body composition. RMR values relative to body weight and/or FFM can then be compared to detect the suppression of RMR. Whilst considering adaptive thermogenesis in cases where body weight is being lost or gained, there are several scenarios that could be considered an indicator of supressed RMR. For example, decreasing RMR when body weight remains unchanged or greater than expected losses to RMR during weight loss.

### Measurement Methods and Test Preparation Procedures

Differences in RMR measurement methodologies and preparation procedures could also influence RMR results, with a wide variety of methods and procedures evident across included studies. Some methodologies include the use of a discard period, a steady-state model, and a validated RMR extraction method, whereas others do not. For the Harris-Benedict (1918) equation, a small overestimation was observed when these protocols were omitted, whereas a moderate underestimation was observed when these protocols were present. Although other equations (the Cunningham [1980], Mifflin St. Jeor and FAO/WHO/UNU [age, weight]) did not differ with the presence or lack of these protocols, these protocols should be employed to ensure accuracy of measurement according to best practice [45].

Furthermore, some studies additionally omitted subject preparation protocols whilst others included these protocols. Such protocols include fasting for at least 7 h and abstaining from both caffeine/stimulants and nicotine for at least 4 and 2.5 h, respectively, before the measurement of RMR. Only studies using the Cunningham (1980) (LBM) and the Harris-Benedict (1918) (age, weight, height) equations were possible to analyse. These showed differences between subgroups depending on implementation of the criteria or not. However, no clear pattern of results was evident, and accuracy was not shown to be improved by their implementation. Once again, large heterogeneity was evident for most subgroups. It is also unclear whether these protocols were employed in the original studies in which these equations were derived [7, 8]. Although there is mention of fasting and physical rest during testing, there is no information on any other methodological procedures that were employed [7, 8]. Therefore, it is inappropriate to deduce a true effect of the presence/lack of these preparation protocols on the accuracy of the equations, and they remain best practice.

### Methodological Considerations

Some methodological aspects of the current review should be considered. Variability between studies is inevitable in a systematic review [44]. Similar to a meta-analysis of studies examining activity energy expenditure monitors [65], this review demonstrated large heterogeneity between and within RMR prediction equations. Taking the variability into account, a random effects model was employed, and narrative synthesis and pre-specified subgroup analysis were conducted to examine the role of participant and methodological diversity. In addition, most studies provided comparable data for meta-analysis similar to those examining the accuracy of RMR equations in other populations [66]. Therefore, the present analysis should contribute to any future research on the accuracy/precision of equations.

It is also acknowledged that meta-analysis results will be influenced by the number of comparisons made. For example, although the Ten-Haaf (2014) (age, weight, height) equation was found to be the best performing equation and had the least heterogeneity, only seven comparison groups from four separate studies contributed to this result. An increase in the number of comparisons and studies to approximately match the Cunningham (1980) (LBM) and the Harris-Benedict (1918) (age, weight, height) equations (29 and 30 comparisons, 17 and 18 separate studies, respectively) is required to better understand the performance of the Ten-Haaf (2014) (age, weight, height) equation relative to more frequently used equations.

For locally derived equations, it is important to note that potential for bias exists when assessing the performance of the equation within the same cohort from which it was initially derived. This may inflate the reported efficacy of the equation. To mitigate this risk, validation of these locally derived equations within separate cohorts is recommended to facilitate an impartial evaluation of their predictive performance. As shown in Table 2, the majority of locally derived equations were not cross-validated internally or externally in athletes. Therefore, until such validation studies are performed, caution is warranted before using these in practice. In addition, the criteria reported for appropriately validating an equation should be considered. This review focused on mean ± SD bias between predicted and measured values, and precision. These variables were selected for several reasons, including being (i) the most commonly reported or possible to derive from existing studies; (ii) able to provide insight into equation performance at the group and individual level; and (iii) in line with previous literature whereby accurate predicted values were defined as those falling within ± 10% of measured values [34–36]. Focusing on mean bias alone could mask important inter-individual differences. For example, Balci et al. [32] found no significant bias between measured and predicted values by Harris-Benedict (1918) equation, but only 40% of participants were calculated to be within 10% of measured values. Therefore, while mean bias may indicate direction of values on a group level, it should be considered alongside the limits of agreement and percentage precision of an equation when determining the most appropriate equation for an individual. While some studies reported root mean square error, it was not consistently reported or possible to derive. For standardising reporting in future studies, the root mean square error would be valuable to report alongside bias, limits of agreement and precision.

The characteristics of athletes included in the present study should be considered when interpreting the generalisability of findings. Despite inclusion criteria spanning adult athletes aged 18–65 years, data extraction revealed that studies meeting inclusion criteria involved adults of 18–35 years. Studies in masters athletes aged 35–84 years inclusive [67] and in youth athletes [68, 69] have been conducted but did not provide a breakdown of equation performance by age category. Consequently, these studies were not eligible for inclusion but are of interest when considering RMR prediction for athletes in these categories. Further research is needed to validate equations in adult athletes aged 35–65 years to inform recommendations for this age group. Racial differences in RMR should also be considered when interpreting findings. The majority of studies included did not specify participant race, solely reporting nationality and only in some cases. Given evidence that race may influence RMR [70], further studies are needed to incorporate and compare athletes of different racial backgrounds to determine whether this may impact on choice of equation.

#### Emerging Research and Practical Implications

The increasing interest in identifying the best equations for predicting RMR in athlete groups is evident by the number of recent publications on this topic and new equations proposed. As shown in Table 2, out of 14 studies proposing equations based on athlete populations, the first was in 1999 by De Lorenzo, and the majority were published in the last decade. It should also be noted that between the final search date (November 2021) and June 2023, five further studies were published that fit the inclusion criteria. These studies are not included in the narrative synthesis and meta-analyses presented. However, in order to compare with the overall findings of the current review, the key findings from these studies along with the performance of the Ten-Haaf (2014) (age, weight, height) equation (found to be most accurate and precise overall) are discussed below and findings noted in Table 2.

Of these five studies, three [71–73] did not include the Ten-Haaf (2014) (age, weight, height) equation. In these studies, the key findings were as follows:

- In NCAA collegiate men and women athletes, all prediction equations investigated (Cunningham, De Lorenzo, Freire, Harris-Benedict, Mifflin St. Jeor, Nelson, Owen, Tinsley, Watson, Schofield) were found to underestimate RMR [72].
- In Korean collegiate soccer players, the Taguchi (2011) equation performed best out of five FFM-based RMR equations [71].
- In groups of active resistance trained females and males, the Cunningham (1991) and the DeLorenzo (1999) equations respectively were closest to measured values of seven equations studied [73].

In the two studies that included the Ten-Haaf (2014) (age, weight, height) equation [74, 75], both showed that predicted RMR values did not differ significantly from measured values. Inclusion of these data in the overall meta-analysis for the Ten-Haaf (2014) (age, weight, height) equation resulted in an ES of − 0.02 (95% CI − 0.17 to 0.14, *p* = 0.83, *I*^2^ = 0%) and MD − 2.6 kcal/24h (95% CI − 29.6 to 24.4), compared with the original meta-analysis findings reported of ES = 0.04 (95% CI − 0.16 to 0.23, *p* = 0.70, *I*^2^ = 0%). Therefore, these data support the overall findings of our review.

In relation to the two latter studies, Freire et al. [74] also proposed two new equations derived from 71 ‘high-level’ Brazilian athletes, all minimum national level (majority Tier 4–5, 87% World Championship, 45% Olympic level) from 21 different sports. The equation was cross-validated in a further sample of 31 athletes in the same study. Elsewhere, Van Hooren et al. found the Ten-Haaf (2014) (age, weight, height) equation performed best, while the Oxford equation [20] underestimated RMR in 25 professional cyclists [75]. The authors also developed a new equation for use in professional cyclists. These equations require further validation in future studies.

**Fig. 3:**
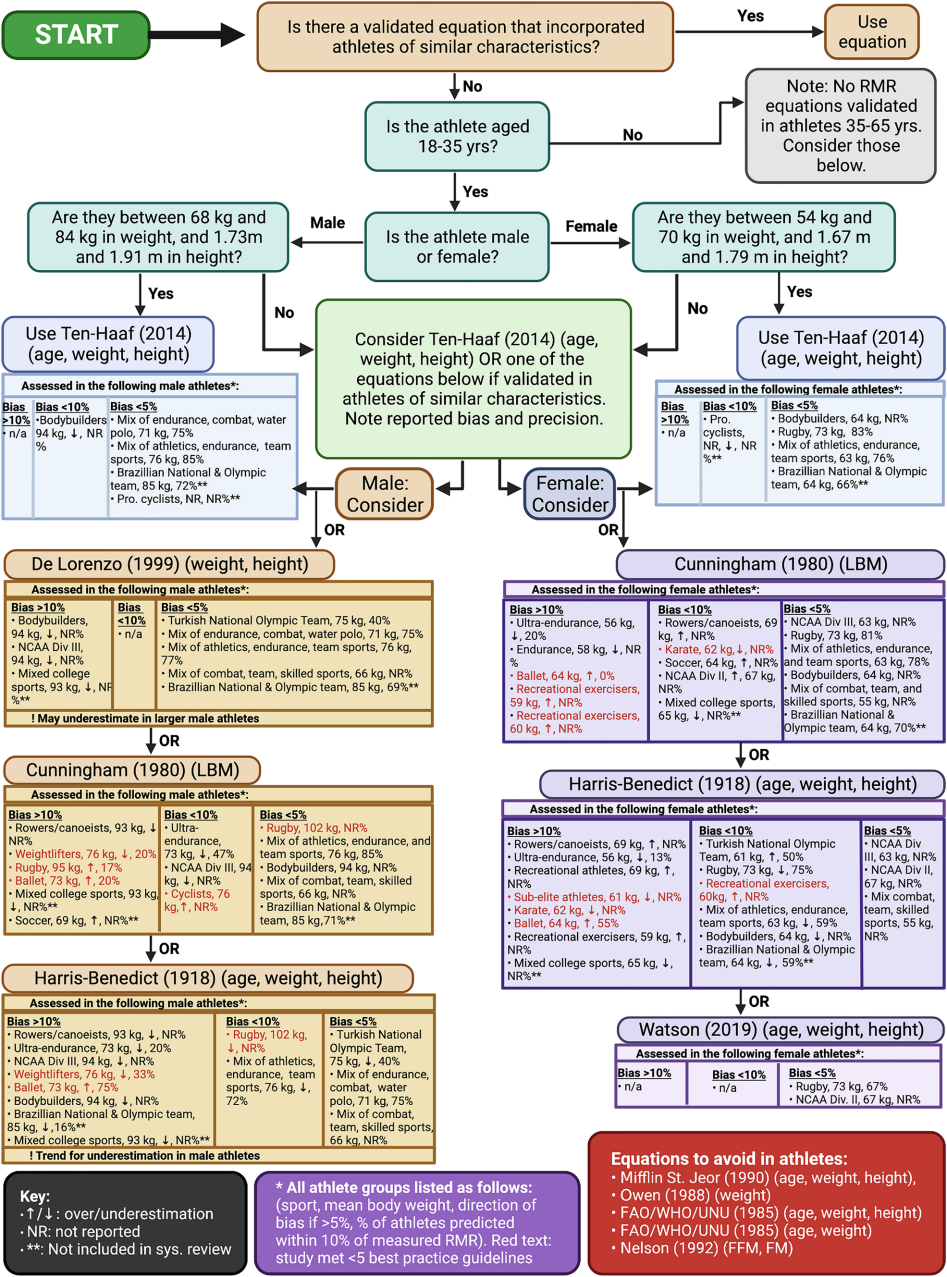
Flow chart to guide choice of equation for predicting resting metabolic rate (RMR) in an athlete. Ideally an equation developed in or validated in athletes of similar characteristics (considering age, sex, body composition) should be used. If not available, the equations shown (which demonstrated no overall mean bias in the present meta-analysis and have been validated for some athlete groups) could be considered. Under each equation, the key characteristics of athletes that have been studied (including sport and mean body weight), mean bias and precision (where available) are reported. This information should be considered when selecting an equation to best match the athlete/s of interest. Studies that met below average best practice RMR measurement guidelines are shown in red text and should be interpreted more cautiously. Full details of each equation, the population(s) it was developed and validated in along with references are shown in Table 2 and Supplementary Document 7 (see ESM). Created in Biorender.com. *LBM* lean body mass, *RMR* resting metabolic rate

To aid decision making regarding choice of equation, an overview of considerations based on the evidence in this review is presented in Fig. 3. Due to emerging studies and new equations, the best equations for use are likely to evolve. Therefore, when determining the equation to use, practitioners and researchers should first consider whether there is an appropriately validated equation developed in athletes of similar characteristics to the athlete/s of interest. If not available, equations that have been externally validated in studies of athletes of similar characteristics should be considered and reported accuracy and precision considered when interpreting results (Fig. 3, Table 2, Supplementary Document 7 (see ESM)).

## Conclusion

Many different RMR prediction equations have been used in athletes. These can differ widely in accuracy and precision. Choosing a prediction equation based on an athlete population of similar characteristics (physical characteristics, sex, sport, athlete status) is the preferred option. While no single equation is guaranteed to be superior, the Ten-Haaf (2014) (age, weight, height) equation appears to be most accurate and precise in estimating RMR in general athlete groups. In addition, some equations have been observed to consistently underperform in athletes and should be avoided. Caution should be applied when utilising any prediction equation for the cross-sectional calculation of RMR ratio as a proxy indicator of LEA.

### Supplementary Information

Below is the link to the electronic supplementary material.

Supplementary file1 (DOCX 4774 KB)

## Declarations

### Data Availability

All of the data generated and/or analysed during the current study are available from the corresponding author on reasonable request.

## References

[1] Thomas DT, Erdman KA, Burke LM (2016). Position of the Academy of Nutrition and Dietetics, Dietitians of Canada, and the American College of Sports Medicine: Nutrition and Athletic Performance. J Acad Nutr Diet.

[2] Poehlman ET (1989). A review: exercise and its influence on resting energy metabolism in man. Med Sci Sports Exerc.

[3] Staal S, Sjödin A, Fahrenholtz I, Bonnesen K, Melin AK (2018). Low RMR (ratio) as a surrogate marker for energy deficiency, the choice of predictive equation vital for correctly identifying male and female ballet dancers at risk. Int J Sport Nutr Exerc Metab.

[4] Stenqvist TB, Torstveit MK, Faber J, Melin AK (2020). Impact of a 4-week intensified endurance training intervention on markers of relative energy deficiency in sport (RED-S) and performance among well-trained male cyclists. Front Endocrinol.

[5] Stenqvist TB, Melin AK, Garthe I (2021). Prevalence of surrogate markers of relative energy deficiency in male Norwegian olympic-level athletes. Int J Sport Nutr Exerc Metab.

[6] De Souza MJ, Hontscharuk R, Olmsted M, Kerr G, Williams NI (2007). Drive for thinness score is a proxy indicator of energy deficiency in exercising women. Appetite.

[7] Harris JA, Benedict FG (1918). A biometric study of human basal metabolism. Proc Natl Acad Sci USA.

[8] Cunningham JJ (1980). A reanalysis of the factors influencing basal metabolic rate in normal adults. Am J Clin Nutr.

[9] O’Neill JERG, Walsh CS, McNulty SJ (2020). Resting metabolic rate in female rugby players: differences in measured versus predicted values. J Strength Cond Res.

[10] Morehen JC, Bradley WJ, Clarke J (2016). The assessment of total energy expenditure during a 14-day in-season period of professional rugby league players using the doubly labelled water method. Int J Sport Nutr Exerc Metab.

[11] Flack KD, Siders WA, Johnson L, Roemmich JN (2016). Cross-validation of resting metabolic rate prediction equations. J Acad Nutr Diet.

[12] Jagim AR, Camic CL, Kisiolek J (2018). Accuracy of resting metabolic rate prediction equations in athletes. J Strength Cond Res.

[13] Kim JH, Kim MH, Kim GS, Park JS, Kim EK (2015). Accuracy of predictive equations for resting metabolic rate in Korean athletic and non-athletic adolescents. Nutr Res Pract.

[14] Carlsohn A, Scharhag-Rosenberger F, Cassel M, Mayer F (2011). Resting metabolic rate in elite rowers and canoeists: difference between indirect calorimetry and prediction. Ann Nutr Metab.

[15] Schofield KL, Thorpe H, Sims ST (2019). Resting metabolic rate prediction equations and the validity to assess energy deficiency in the athlete population. Exp Physiol.

[16] Owen OE, Holup JL, D’Alessio DA (1987). A reappraisal of the caloric requirements of men. Am J Clin Nutr.

[17] Mifflin MD, St Jeor ST, Hill LA, Scott BJ, Daugherty SA, Koh YO (1990). A new predictive equation for resting energy expenditure in healthy individuals. Am J Clin Nutr.

[18] Nelson KM, Weinsier RL, Long CL, Schutz Y (1992). Prediction of resting energy expenditure from fat-free mass and fat mass. Am J Clin Nutr.

[19] De Lorenzo A, Bertini I, Candeloro N, Piccinelli R, Innocente I, Brancati A (1999). A new predictive equation to calculate resting metabolic rate in athletes. J Sports Med Phys Fit.

[20] Henry CJK (2005). Basal metabolic rate studies in humans: measurement and development of new equations. Public Health Nutr.

[21] ten Haaf T, Weijs PJM (2014). Resting energy expenditure prediction in recreational athletes of 18–35 years: confirmation of Cunningham equation and an improved weight-based alternative. PLoS One.

[22] Jagim A, Camic C, Askow A (2019). Sex differences in resting metabolic rate among athletes. J Strength Cond Res.

[23] Tinsley GM, Graybeal AJ, Moore ML (2019). Resting metabolic rate in muscular physique athletes: validity of existing methods and development of new prediction equations. Appl Physiol Nutr Metab Physiol Appl Nutr Metab.

[24] Wang Z, Heshka S, Gallagher D, Boozer CN, Kotler DP, Heymsfield SB (2000). Resting energy expenditure-fat-free mass relationship: new insights provided by body composition modeling. Am J Physiol Endocrinol Metab.

[25] Watson AD, Zabriskie HA, Witherbee KE, Sulavik A, Gieske BT, Kerksick CM (2019). Determining a resting metabolic rate prediction equation for collegiate female athletes. J Strength Cond Res.

[26] Energy and protein requirements. Report of a joint FAO/WHO/UNU Expert Consultation. World Health Organ Tech Rep Ser. 1985;724:1–206. 3937340

[27] Devrim-Lanpir A, Kocahan T, Deliceoglu G, Tortu E, Bilgic P (2019). Is there any predictive equation to determine resting metabolic rate in ultra-endurance athletes?. Prog Nutr.

[28] Joseph M, Gupta RD, Prema L, Inbakumari M, Thomas N (2017). Are predictive equations for estimating resting energy expenditure accurate in Asian Indian male weightlifters?. Indian J Endocrinol Metab.

[29] Mackay KJ, Schofield KL, Sims ST, McQuillan JA, Driller MW (2019). The validity of resting metabolic rate-prediction equations and reliability of measured RMR in female athletes. Int J Exerc Sci.

[30] Strock NCA, Koltun KJ, Southmayd EA, Williams NI, De Souza MJ (2020). Indices of resting metabolic rate accurately reflect energy deficiency in exercising women. Int J Sport Nutr Exerc Metab.

[31] McInnes MDF, Moher D, Thombs BD (2018). Preferred reporting items for a systematic review and meta-analysis of diagnostic test accuracy studies: the PRISMA-DTA statement. JAMA.

[32] Balci A, Badem EA, Yılmaz AE (2021). Current predictive resting metabolic rate equations are not sufficient to determine proper resting energy expenditure in olympic young adult national team athletes. Front Physiol.

[33] Marra M, Di Vincenzo O, Cioffi I, Sammarco R, Morlino D, Scalfi L (2021). Resting energy expenditure in elite athletes: development of new predictive equations based on anthropometric variables and bioelectrical impedance analysis derived phase angle. J Int Soc Sports Nutr.

[34] Weijs PJM (2008). Validity of predictive equations for resting energy expenditure in US and Dutch overweight and obese class I and II adults aged 18–65 y. Am J Clin Nutr.

[35] Frankenfield DC, Rowe WA, Smith JS, Cooney RN (2003). Validation of several established equations for resting metabolic rate in obese and nonobese people. J Am Diet Assoc.

[36] Frankenfield D, Roth-Yousey L, Compher C (2005). Comparison of predictive equations for resting metabolic rate in healthy nonobese and obese adults: a systematic review. J Am Diet Assoc.

[37] Phang PT, Rich T, Ronco J (1990). A validation and comparison study of two metabolic monitors. JPEN J Parenter Enteral Nutr.

[38] Landis JR, Koch GG (1977). The measurement of observer agreement for categorical data. Biometrics.

[39] McKay AKA, Stellingwerff T, Smith ES (2022). Defining training and performance caliber: a participant classification framework. Int J Sports Physiol Perform.

[40] Review Manager (RevMan). Published online 2020.

[41] Hedges LV (1981). Distribution theory for glass’s estimator of effect size and related estimators. J Educ Stat.

[42] Cohen J (1988). Statistical power analysis for the behavioral sciences.

[43] Higgins JPT, Thompson SG, Spiegelhalter DJ (2009). A re-evaluation of random-effects meta-analysis. J R Stat Soc Ser A Stat Soc.

[44] Higgins JPT, Green S. Cochrane handbook for systematic reviews of interventions, Version 5.1.0 [Updated March 2011]. The Cochrane Collaboration; 2011.

[45] Fullmer S, Benson-Davies S, Earthman CP (2015). Evidence analysis library review of best practices for performing indirect calorimetry in healthy and non-critically ill individuals. J Acad Nutr Diet.

[46] Büttner F, Winters M, Delahunt E (2020). Identifying the ’incredible’! Part 2: spot the difference—a rigorous risk of bias assessment can alter the main findings of a systematic review. Br J Sports Med.

[47] Whiting P, Rutjes AWS, Reitsma JB, Bossuyt PMM, Kleijnen J (2003). The development of QUADAS: a tool for the quality assessment of studies of diagnostic accuracy included in systematic reviews. BMC Med Res Methodol.

[48] Whiting PF, Rutjes AWS, Westwood ME (2011). QUADAS-2: a revised tool for the quality assessment of diagnostic accuracy studies. Ann Intern Med.

[49] Wolff RF, Moons KGM, Riley RD (2019). PROBAST: a tool to assess the risk of bias and applicability of prediction model studies. Ann Intern Med.

[50] Sterne JA, Hernán MA, Reeves BC (2016). ROBINS-I: a tool for assessing risk of bias in non-randomised studies of interventions. BMJ.

[51] Nichols S, George D, Prout P, Dalrymple N (2020). Accuracy of resting metabolic rate prediction equations among healthy adults in Trinidad and Tobago. Nutr Health.

[52] Gravante G, Pomara F, Angelomè C, Russo G, Truglio G (2001). The basal energy expenditure of female athletes vs. sedentary women as related to their family history of type 2 diabetes. Acta Diabetol.

[53] Melin A, Tornberg A, Skouby S (2015). Energy availability and the female athlete triad in elite endurance athletes. Scand J Med Sci Sports.

[54] Langan-Evans C, Germaine M, Artukovic M (2020). The psychological and physiological consequences of low energy availability in a male combat sport athlete. Med Sci Sports Exerc.

[55] Moss SL, Randell RK, Burgess D (2021). Assessment of energy availability and associated risk factors in professional female soccer players. Eur J Sport Sci.

[56] Thompson J, Manore MM, Skinner JS (1993). Resting metabolic rate and thermic effect of a meal in low- and adequate-energy intake male endurance athletes. Int J Sport Nutr.

[57] Strock N, Koltun K, Mallinson R, Williams N, De Souza M (2020). Characterizing the resting metabolic rate ratio in ovulatory exercising women over 12 months. Scand J Med Sci Sports.

[58] Koehler K, Williams NI, Mallinson RJ, Southmayd EA, Allaway HCM, De Souza MJ (2016). Low resting metabolic rate in exercise-associated amenorrhea is not due to a reduced proportion of highly active metabolic tissue compartments. Am J Physiol Endocrinol Metab.

[59] Sjodin A, Forslund A, Westerterp K, Andersson A, Forslund J, Hambraeus L (1996). The influence of physical activity on BMR. Med Sci Sports Exerc.

[60] MacKenzie-Shalders KL, Byrne NM, King NA, Slater GJ (2019). Are increases in skeletal muscle mass accompanied by changes to resting metabolic rate in rugby athletes over a pre-season training period?. Eur J Sport Sci.

[61] Wong JE, Poh BK, Nik Shanita S (2012). Predicting basal metabolic rates in Malaysian adult elite athletes. Singapore Med J.

[62] Marques LR (2021). Basal metabolic rate for high-performance female karate athletes. Nutr Hosp.

[63] Cunningham JJ (1991). Body composition as a determinant of energy expenditure: a synthetic review and a proposed general prediction equation. Am J Clin Nutr.

[64] Sterringer T, Larson-Meyer DE (2022). RMR ratio as a surrogate marker for low energy availability. Curr Nutr Rep.

[65] O’Driscoll R, Turicchi J, Beaulieu K (2020). How well do activity monitors estimate energy expenditure? A systematic review and meta-analysis of the validity of current technologies. Br J Sports Med.

[66] Macena M de L, Paula DT da C, da Silva Júnior AE, et al. Estimates of resting energy expenditure and total energy expenditure using predictive equations in adults with overweight and obesity: a systematic review with meta-analysis. Nutr Rev. 2022;80(11):2113–5. 10.1093/nutrit/nuac031 10.1093/nutrit/nuac031 35551409

[67] Frings-Meuthen P, Henkel S, Boschmann M (2021). Resting energy expenditure of master athletes: accuracy of predictive equations and primary determinants. Front Physiol.

[68] Hannon MP, Carney DJ, Floyd S (2020). Cross-sectional comparison of body composition and resting metabolic rate in Premier League academy soccer players: implications for growth and maturation. J Sports Sci.

[69] Reale RJ, Roberts TJ, Lee KA, Bonsignore JL, Anderson ML (2020). Metabolic rate in adolescent athletes: the development and validation of new equations, and comparison to previous models. Int J Sport Nutr Exerc Metab.

[70] Reneau J, Obi B, Moosreiner A, Kidambi S (2019). Do we need race-specific resting metabolic rate prediction equations?. Nutr Diabetes.

[71] Lee S, Moto K, Oh T, Taguchi M (2022). Comparison between predicted and measured resting energy expenditures in Korean male collegiate soccer players. Phys Act Nutr.

[72] Fields JB, Magee MK, Jones MT (2022). The accuracy of ten common resting metabolic rate prediction equations in men and women collegiate athletes. Eur J Sport Sci.

[73] Sordi AF, Mariano IR, Silva BF, Magnani Branco BH (2022). Resting metabolic rate in bodybuilding: differences between indirect calorimetry and predictive equations. Clin Nutr ESPEN.

[74] Freire R, Pereira G, Alcantara J, Santos R, Hausen M, Itaborahy A (2023). New predictive resting metabolic rate equations for high-level athletes: a cross-validation study. Med Sci Sports Exerc.

[75] Van Hooren B, Cox M, Rietjens G, Plasqui G (2023). Determination of energy expenditure in professional cyclists using power data: validation against doubly labeled water. Scand J Med Sci Sports.

[76] Taguchi M, Ishikawa-Takata K, Tatsuta W (2011). Resting energy expenditure can be assessed by fat-free mass in female athletes regardless of body size. J Nutr Sci Vitaminol (Tokyo).

[77] Handu D, Moloney L, Wolfram T, Ziegler P, Acosta A, Steiber A (2016). Academy of nutrition and dietetics methodology for conducting systematic reviews for the evidence analysis library. J Acad Nutr Diet.

[78] Pařízková J, Bůžková P (1971). Relationship between skinfold thickness measured by Harpenden caliper and densitometric analysis of total body fat in men. Hum Biol.

[79] Mountjoy M, Sundgot-Borgen J, Burke L (2018). International Olympic Committee (IOC) Consensus Statement on Relative Energy Deficiency in Sport (RED-S): 2018 Update. Int J Sport Nutr Exerc Metab.

[80] Melin A, Tornberg A, Skouby S (2014). The LEAF questionnaire: a screening tool for the identification of female athletes at risk for the female athlete triad. Br J Sports Med.

[81] Jackson AS, Pollock ML (1985). Practical Assessment of Body Composition. Phys Sportsmed.

[82] Watson WS (2001). Predictive equations for skeletal muscle mass. Am J Clin Nutr.

[83] Fleisch AL (1951). Metabolisme basal standard et sa determination au moyen du’metabocalculator’. Helv Med Acta.

[84] Robertson JD, Reid DD (1952). Standards for the basal metabolism of normal people in Britain. Lancet.

[85] Bernstein RS, Thornton JC, Yang MU (1983). Prediction of the resting metabolic rate in obese patients. Am J Clin Nutr.

[86] Roza AM, Shizgal HM (1984). The Harris Benedict equation reevaluated: resting energy requirements and the body cell mass. Am J Clin Nutr.

[87] Schofield WN (1985). Predicting basal metabolic rate, new standards and review of previous work. Hum Nutr Clin Nutr.

[88] *Nutrient Requirements and Recommended Dietary Allowances for Indians: A Report of the Expert Group of the Indian Council of Medical Research.* 1st ed. Indian Council of Medical Research; 1990.

[89] Henry CJ, Rees DG (1991). New predictive equations for the estimation of basal metabolic rate in tropical peoples. Eur J Clin Nutr.

[90] Liu HY, Lu YF, Chen WJ (1995). Predictive equations for basal metabolic rate in Chinese adults: a cross-validation study. J Am Diet Assoc.

[91] Westerterp KR, Donkers JH, Fredrix EW, Boekhoudt P (1995). Energy intake, physical activity and body weight: a simulation model. Br J Nutr.

[92] Società Italiana di Nutrizione (SINU). Recommended Assumption Levels of Energy and Nutrients for Italian Population—Livelli di Assunzione Raccomandata di Nutrienti per la Popolazione Italiana (L.A.R.N); 1996.

[93] Ismail M, Chee S, Roslee R, Zawiah H (1998). Predictive equations for the estimation of basal metabolic rate in Malaysian adults. Malays J Nutr.

[94] McArdle WD, Katch FI, Katch VL (2010). Exercise physiology: nutrition, energy, and human performance.

[95] Hayes M, Chustek M, Wang Z (2002). DXA: potential for creating a metabolic map of organ-tissue resting energy expenditure components. Obes Res.

[96] Lührmann PM, Herbert BM, Krems C, Neuhäuser-Berthold M (2002). A new equation especially developed for predicting resting metabolic rate in the elderly for easy use in practice. Eur J Nutr.

[97] Huang KC, Kormas N, Steinbeck K, Loughnan G, Caterson ID (2004). Resting metabolic rate in severely obese diabetic and nondiabetic subjects. Obes Res.

[98] Müller MJ, Bosy-Westphal A, Klaus S (2004). World Health Organization equations have shortcomings for predicting resting energy expenditure in persons from a modern, affluent population: generation of a new reference standard from a retrospective analysis of a German database of resting energy expenditure. Am J Clin Nutr.

[99] Livingston EH, Kohlstadt I (2005). Simplified resting metabolic rate-predicting formulas for normal-sized and obese individuals. Obes Res.

[100] Johnstone AM, Rance KA, Murison SD, Duncan JS, Speakman JR (2006). Additional anthropometric measures may improve the predictability of basal metabolic rate in adult subjects. Eur J Clin Nutr.

[101] Korth O, Bosy-Westphal A, Zschoche P, Glüer CC, Heller M, Müller MJ (2007). Influence of methods used in body composition analysis on the prediction of resting energy expenditure. Eur J Clin Nutr.

[102] Lazzer S, Agosti F, Resnik M, Marazzi N, Mornati D, Sartorio A (2007). Prediction of resting energy expenditure in severely obese Italian males. J Endocrinol Investig.

[103] Horie LM, Gonzalez MC, Torrinhas RS, Cecconello I, Waitzberg DL (2011). New specific equation to estimate resting energy expenditure in severely obese patients. Obes Silver Spring Md.

[104] Sabounchi NS, Rahmandad H, Ammerman A (2013). Best-fitting prediction equations for basal metabolic rate: informing obesity interventions in diverse populations. Int J Obes.

