# Supplementary material for: Accuracy of Resting Metabolic Rate Prediction Equations in Athletes: A Systematic Review with Meta-analysis
Source: Sports Med. 2023 Aug 26;53(12):2373–98. doi: 10.1007/s40279-023-01896-z (PMC10687135; doi:10.1007/s40279-023-01896-z)
Supplement: Supplementary file 1 — Supplementary file1 (DOCX 4774 KB) [file 40279_2023_1896_MOESM1_ESM.docx]

**Article title:** Accuracy of resting metabolic rate prediction equations in athletes: a systematic review and meta-analysis

**Journal:** Sports medicine

**Authors:**

Mr. Jack Eoin Rua O’Neill^1^ (ORCiD: 0000-0001-5785-5665)

Prof. Clare A. Corish^2^ (ORCiD: 0000-0002-8986-677X)

Dr. Katy Horner^1^ (ORCiD: 0000-0002-5046-3658)

^1^Institute for Sport and Health and School of Public Health, Physiotherapy & Sport Science, University College Dublin, Ireland.

^2^ School of Public Health, Physiotherapy & Sport Science, University College Dublin, Ireland.

**Corresponding Author:**

Jack Eoin Rua O’Neill,

UCD Institute for Sport and Health,

University College Dublin,

Belfield,
Dublin 4.

Email: jack.o-neill.1@ucdconnect.ie

**Title of supplementary material:** Supplementary Document 1 – Search strategy for systematic review

Primary Search Commenced at 11:15 on 10^th^ DEC 2020

PubMed Search = 94 hits

((athlete*[tiab]) OR (athletes[MeSH Terms]) OR (athlete[MeSH Terms]) OR (sport*[tiab]) OR (sports[MeSH Terms]) OR (exercis*[tiab]) OR (exercise[MeSH Terms])) AND ((basal metabolism[MeSH Terms]) OR ("basal metabolism"[tiab]) OR ("resting energy expenditure*"[tiab]) OR ("REE"[tiab]) OR ("resting metabolic rate*"[tiab]) OR ("RMR"[tiab]) OR (calorimetry, indirect[MeSH Terms]) OR ("indirect calorimetry"[tiab])) AND (("prediction equation*"[tiab]) OR ("predictive equation*"[tiab]))

Embase via OVID = 270 hits

(athlete*.tw. OR exp athletes/ OR exp athlete/ OR sport*.tw. OR exp sports/ OR exercis*.tw. OR exp exercise/) AND (exp basal metabolism/ OR "basal metabolism".tw. OR "resting energy expenditure*".tw. OR "REE".tw. OR "resting metabolic rate*".tw. OR "RMR".tw.) OR exp indirect calorimetry OR "indirect calorimetry".tw.) AND ("prediction equation*".tw. OR "predictive equation*".tw.)

CINAHL Plus = 26 hits

((MH “athletes”) OR (MH “athlete”) OR (MH “sports”) OR (MH “exercise”) OR TI “athlete*” OR AB “athlete*” OR TI “sport*” OR AB “sport*” OR TI “exercise*” OR AB “exercise*”) AND ((MH “basal metabolism”) OR (MH “indirect calorimetry”) OR TI “basal metabolism” OR AB “basal metabolism” OR TI “resting energy expenditure*” OR AB “resting energy expenditure*” OR TI “REE” OR AB “REE” OR TI “resting metabolic rate*” OR AB “resting metabolic rate*” OR TI “RMR” OR AB “RMR” OR TI “indirect calorimetry” OR AB “indirect calorimetry”) AND (TI “prediction equation*” OR AB “prediction equation*” OR TI “predictive equation*” OR AB “predictive equation*”)

SPORTdiscus = 33 hits

((SU “athletes”) OR (SU “athlete”) OR (SU “sports”) OR (SU “exercise”) OR TI “athlete*” OR AB “athlete*” OR TI “sport*” OR AB “sport*” OR TI “exercise*” OR AB “exercise*”) AND ((SU “basal metabolism”) OR (SU “indirect calorimetry”) OR TI “basal metabolism” OR AB “basal metabolism” OR TI “resting energy expenditure*” OR AB “resting energy expenditure*” OR TI “REE” OR AB “REE” OR TI “resting metabolic rate*” OR AB “resting metabolic rate*” OR TI “RMR” OR AB “RMR” OR TI “indirect calorimetry” OR AB “indirect calorimetry”) AND (TI “prediction equation*” OR AB “prediction equation*” OR TI “predictive equation*” OR AB “predictive equation*”)

Web of Science = 80 hits

(SU=athletes OR SU=athlete OR SU=sports OR SU=exercise OR TI=athlete* OR AB=athlete* OR TI=sport* OR AB=sport* OR TI=exercise* OR AB=exercise*) AND (SU=basal metabolism OR SU=indirect calorimetry OR TI=basal metabolism OR AB=basal metabolism OR TI=resting energy expenditure* OR AB=resting energy expenditure* OR TI=REE OR AB=REE OR TI=resting metabolic rate* OR AB=resting metabolic rate* OR TI=RMR OR AB=RMR OR TI=indirect calorimetry OR AB=indirect calorimetry) AND (TI=prediction equation* OR AB=prediction equation* OR TI=predictive equation* OR AB=predictive equation*)

Secondary Search Commenced at 08:50 on 12^th^ NOV 2021

PubMed Search = 109 hits

((athlete*[tiab]) OR (athletes[MeSH Terms]) OR (athlete[MeSH Terms]) OR (sport*[tiab]) OR (sports[MeSH Terms]) OR (exercis*[tiab]) OR (exercise[MeSH Terms])) AND ((basal metabolism[MeSH Terms]) OR ("basal metabolism"[tiab]) OR ("resting energy expenditure*"[tiab]) OR ("REE"[tiab]) OR ("resting metabolic rate*"[tiab]) OR ("RMR"[tiab]) OR (calorimetry, indirect[MeSH Terms]) OR ("indirect calorimetry"[tiab])) AND (("prediction equation*"[tiab]) OR ("predictive equation*"[tiab]))

Embase via OVID = 297 hits

(athlete*.tw. OR exp athletes/ OR exp athlete/ OR sport*.tw. OR exp sports/ OR exercis*.tw. OR exp exercise/) AND (exp basal metabolism/ OR "basal metabolism".tw. OR "resting energy expenditure*".tw. OR "REE".tw. OR "resting metabolic rate*".tw. OR "RMR".tw.) OR exp indirect calorimetry OR "indirect calorimetry".tw.) AND ("prediction equation*".tw. OR "predictive equation*".tw.)

CINAHL Plus = 29 hits

((MH “athletes”) OR (MH “athlete”) OR (MH “sports”) OR (MH “exercise”) OR TI “athlete*” OR AB “athlete*” OR TI “sport*” OR AB “sport*” OR TI “exercise*” OR AB “exercise*”) AND ((MH “basal metabolism”) OR (MH “indirect calorimetry”) OR TI “basal metabolism” OR AB “basal metabolism” OR TI “resting energy expenditure*” OR AB “resting energy expenditure*” OR TI “REE” OR AB “REE” OR TI “resting metabolic rate*” OR AB “resting metabolic rate*” OR TI “RMR” OR AB “RMR” OR TI “indirect calorimetry” OR AB “indirect calorimetry”) AND (TI “prediction equation*” OR AB “prediction equation*” OR TI “predictive equation*” OR AB “predictive equation*”)

SPORTdiscus = 36 hits

((SU “athletes”) OR (SU “athlete”) OR (SU “sports”) OR (SU “exercise”) OR TI “athlete*” OR AB “athlete*” OR TI “sport*” OR AB “sport*” OR TI “exercise*” OR AB “exercise*”) AND ((SU “basal metabolism”) OR (SU “indirect calorimetry”) OR TI “basal metabolism” OR AB “basal metabolism” OR TI “resting energy expenditure*” OR AB “resting energy expenditure*” OR TI “REE” OR AB “REE” OR TI “resting metabolic rate*” OR AB “resting metabolic rate*” OR TI “RMR” OR AB “RMR” OR TI “indirect calorimetry” OR AB “indirect calorimetry”) AND (TI “prediction equation*” OR AB “prediction equation*” OR TI “predictive equation*” OR AB “predictive equation*”)

Web of Science = 93 hits

(SU=athletes OR SU=athlete OR SU=sports OR SU=exercise OR TI=athlete* OR AB=athlete* OR TI=sport* OR AB=sport* OR TI=exercise* OR AB=exercise*) AND (SU=basal metabolism OR SU=indirect calorimetry OR TI=basal metabolism OR AB=basal metabolism OR TI=resting energy expenditure* OR AB=resting energy expenditure* OR TI=REE OR AB=REE OR TI=resting metabolic rate* OR AB=resting metabolic rate* OR TI=RMR OR AB=RMR OR TI=indirect calorimetry OR AB=indirect calorimetry) AND (TI=prediction equation* OR AB=prediction equation* OR TI=predictive equation* OR AB=predictive equation*)

Tertiary Search Commenced at 12:05 on 23^rd^ MAY 2023

PubMed Search = 127 hits

((athlete*[tiab]) OR (athletes[MeSH Terms]) OR (athlete[MeSH Terms]) OR (sport*[tiab]) OR (sports[MeSH Terms]) OR (exercis*[tiab]) OR (exercise[MeSH Terms])) AND ((basal metabolism[MeSH Terms]) OR ("basal metabolism"[tiab]) OR ("resting energy expenditure*"[tiab]) OR ("REE"[tiab]) OR ("resting metabolic rate*"[tiab]) OR ("RMR"[tiab]) OR (calorimetry, indirect[MeSH Terms]) OR ("indirect calorimetry"[tiab])) AND (("prediction equation*"[tiab]) OR ("predictive equation*"[tiab]))

Embase via OVID = 318 hits

(athlete*.tw. OR exp athletes/ OR exp athlete/ OR sport*.tw. OR exp sports/ OR exercis*.tw. OR exp exercise/) AND (exp basal metabolism/ OR "basal metabolism".tw. OR "resting energy expenditure*".tw. OR "REE".tw. OR "resting metabolic rate*".tw. OR "RMR".tw.) OR exp indirect calorimetry OR "indirect calorimetry".tw.) AND ("prediction equation*".tw. OR "predictive equation*".tw.)

CINAHL Plus = 34 hits

((MH “athletes”) OR (MH “athlete”) OR (MH “sports”) OR (MH “exercise”) OR TI “athlete*” OR AB “athlete*” OR TI “sport*” OR AB “sport*” OR TI “exercise*” OR AB “exercise*”) AND ((MH “basal metabolism”) OR (MH “indirect calorimetry”) OR TI “basal metabolism” OR AB “basal metabolism” OR TI “resting energy expenditure*” OR AB “resting energy expenditure*” OR TI “REE” OR AB “REE” OR TI “resting metabolic rate*” OR AB “resting metabolic rate*” OR TI “RMR” OR AB “RMR” OR TI “indirect calorimetry” OR AB “indirect calorimetry”) AND (TI “prediction equation*” OR AB “prediction equation*” OR TI “predictive equation*” OR AB “predictive equation*”)

SPORTdiscus = 45 hits

((SU “athletes”) OR (SU “athlete”) OR (SU “sports”) OR (SU “exercise”) OR TI “athlete*” OR AB “athlete*” OR TI “sport*” OR AB “sport*” OR TI “exercise*” OR AB “exercise*”) AND ((SU “basal metabolism”) OR (SU “indirect calorimetry”) OR TI “basal metabolism” OR AB “basal metabolism” OR TI “resting energy expenditure*” OR AB “resting energy expenditure*” OR TI “REE” OR AB “REE” OR TI “resting metabolic rate*” OR AB “resting metabolic rate*” OR TI “RMR” OR AB “RMR” OR TI “indirect calorimetry” OR AB “indirect calorimetry”) AND (TI “prediction equation*” OR AB “prediction equation*” OR TI “predictive equation*” OR AB “predictive equation*”)

Web of Science = 108 hits

(SU=athletes OR SU=athlete OR SU=sports OR SU=exercise OR TI=athlete* OR AB=athlete* OR TI=sport* OR AB=sport* OR TI=exercise* OR AB=exercise*) AND (SU=basal metabolism OR SU=indirect calorimetry OR TI=basal metabolism OR AB=basal metabolism OR TI=resting energy expenditure* OR AB=resting energy expenditure* OR TI=REE OR AB=REE OR TI=resting metabolic rate* OR AB=resting metabolic rate* OR TI=RMR OR AB=RMR OR TI=indirect calorimetry OR AB=indirect calorimetry) AND (TI=prediction equation* OR AB=prediction equation* OR TI=predictive equation* OR AB=predictive equation*)

**Title of supplementary material:** Supplementary Document 2 – Athlete Classification Framework as described by McKay et al. (2022) [39]

**Athlete Classification Framework**

| **Tier** | **Criteria for Classification** |
| --- | --- |
| **Tier 5: World Class**  =<0.00006% of the global population  =<0.001% of the Australian population | •Olympic and/or world medalists.  •World-record holders and athletes achieving within 2% of world-record performance and/or world-  leading performance.  •Top 3–20 in world rankings and/or top 3–10 at an Olympics/World Championships (ie, ﬁnalists in  their event), with this number determined based on size and depth of competition in the event.  •Top players within top teams (teams which medal or are in the most competitive leagues) or athletes  achieving individual accolades (ie, most valuable player, player of the year).  •Maximal, or nearly maximal training, within the given sports norms.  •Exceptional skill-level achieved (ie, running biomechanics, ball skills, acquired decision-making  components)  •Olympic and/or world medalists.  •World-record holders and athletes achieving within 2% of world-record performance and/or world-leading performance.  •Top 3–20 in world rankings and/or top 3–10 at an Olympics/World Championships (i.e., ﬁnalists in their event), with this number determined based on size and depth of competition in the event.  •Top players within top teams (teams which medal or are in the most competitive leagues) or athletes achieving individual accolades (i.e., most valuable player, player of the year).  •Maximal, or nearly maximal training, within the given sports norms.  •Exceptional skill-level achieved (i.e., running biomechanics, ball skills, acquired decision-making components). |
| Tier 4: Elite/International Level  =∼0.0025% of the global population  =∼0.0055% of the Australian population  **Tier 4: Elite/International Level**  =∼0.0025% of the global population  =∼0.0055% of the Australian population | •Competing at the international level (individuals or team-sport athletes on a national team).  •Team-sport athletes competing in international leagues/tournaments.  •Top 4–300 in world rankings, with this number dependant on size and depth of competition in the event.  •Achievement of within ∼7% of world-record performance and/or world-leading performance.  •NCAA Division I athletes.  •Maximal, or nearly maximal training, within the given sports norms, with intention to complete at top-level competition.  •Highly proﬁcient in skills required to perform sport (i.e., biomechanics, ball skills, acquired decision-making components). |
| **Tier 3: Highly Trained/National Level**  (Provincial/State or Academy Programs)  =∼0.014% of the global population  =∼0.027% of the Australian population | •Competing at the national level.  •Team-sport athletes competing in national and/or state leagues/tournaments.  •Achievement of within ∼20% of world-record performance and/or world-leading performance.  •NCAA Division II and III athletes.  •Completing structured and periodized training and developing towards (within 20%) of maximal or nearly maximal norms within the given sport.  •Developing proﬁciency in skills required to perform sport (i.e., biomechanics, ball skills, acquired decision-making components). |
| **Tier 2: Trained/Developmental**  =∼12%–19% of the global population  =∼18% of the Australian population | •Local-level representation.  •Regularly training ∼3 times per week.  •Identify with a speciﬁc sport.  •Training with a purpose to compete.  •Limited skill development. |
| **Tier 1: Recreationally Active**  =∼35%–42% of the global population  =∼30% of the Australian population | •Meet World Health Organization minimum activity guidelines: Adults aged 18–64 years old  completing at least 150 to 300 min moderate-intensity activity or 75–150 min of vigorous-intensity activity a week, plus muscle-strengthening activities 2 or more days a week.  •May participate in multiple sports/forms of activity. |
| **Tier 0: Sedentary**  =∼46% of the global population  =∼52% of the Australian population | •Do not meet minimum activity guidelines.  •Occasional and/or incidental physical activity (e.g., walking to work, household activities). |

**Title of supplementary material:** Supplementary Document 3 – Funnel Plots

**Accuracy MA - Funnel plots for Cunningham (1980) (FFM)**

1. Cunningham (1980) (FFM)


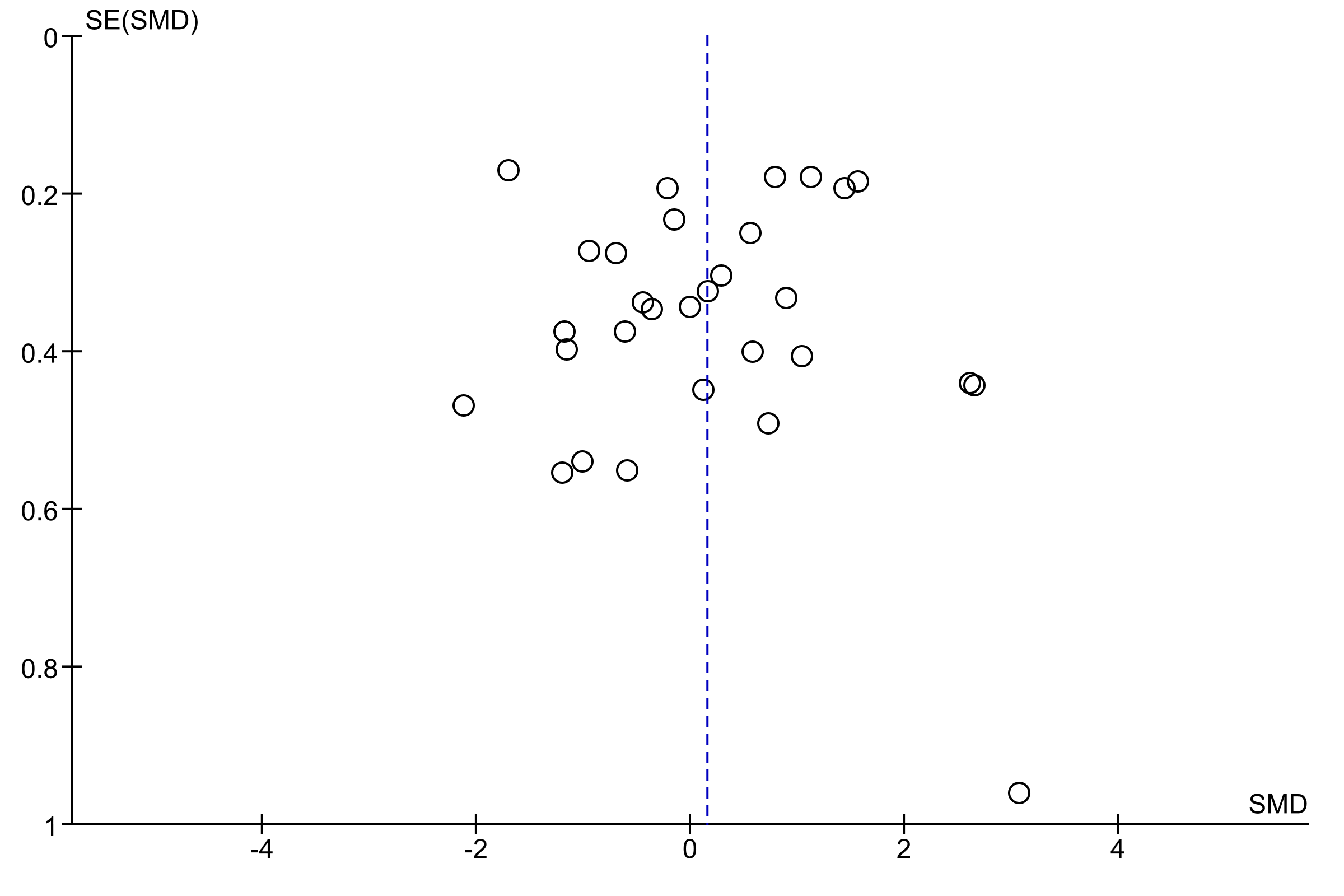


1. Cunningham (1980) (FFM) – Sex


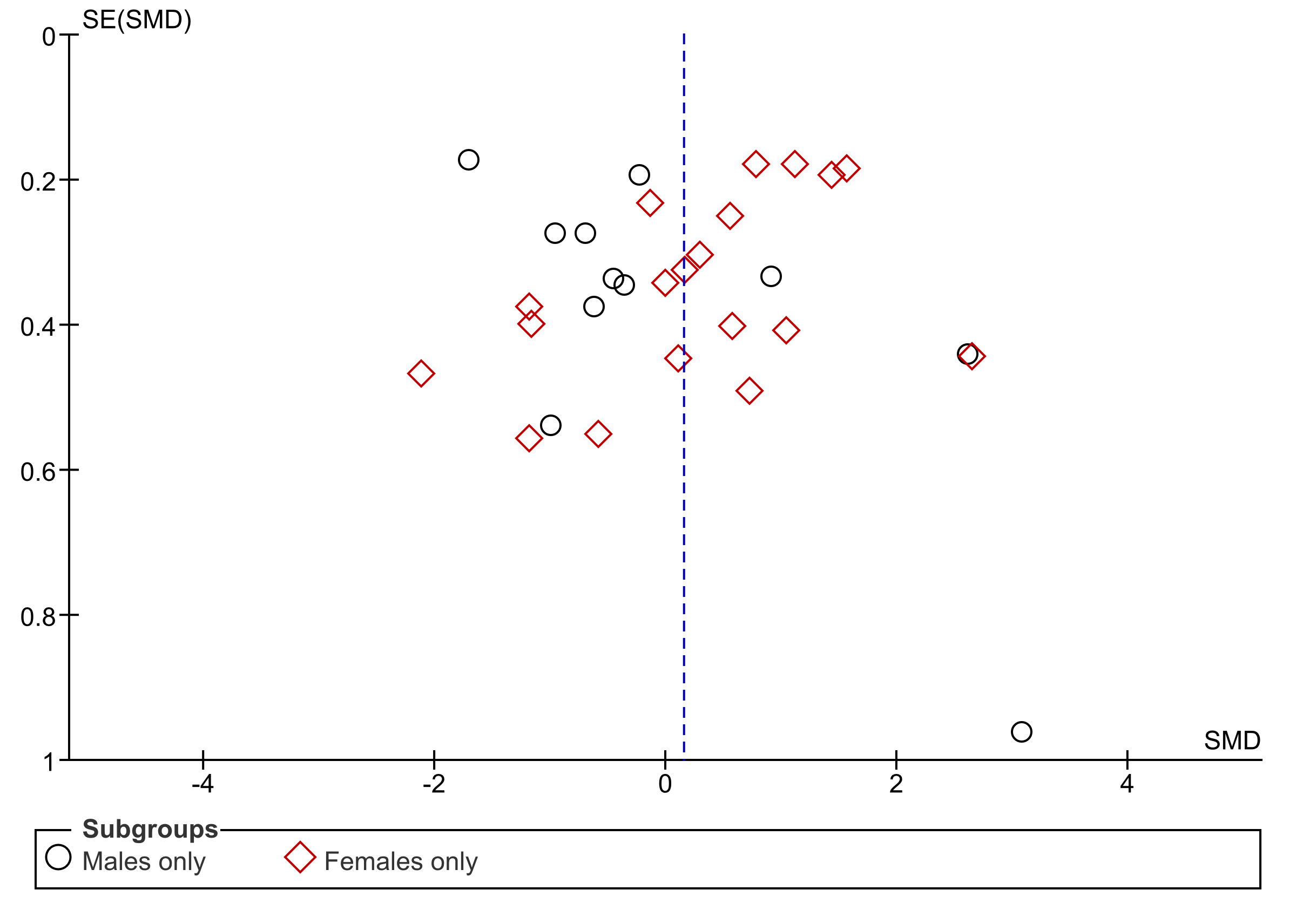


1. Cunningham (1980) (FFM) – Body Composition Method


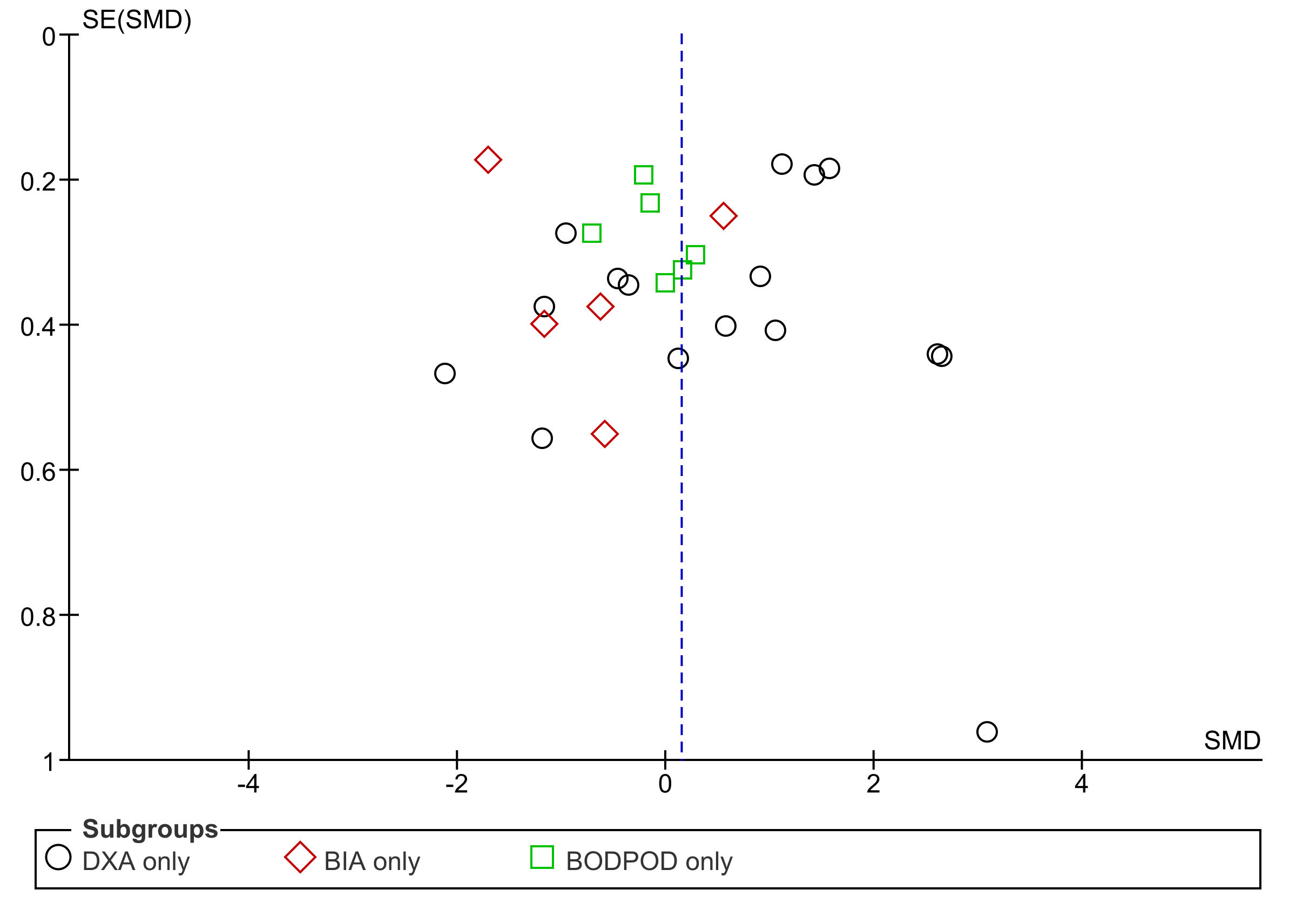


1. Cunningham (1980) (FFM) – Low Energy Availability (LEA)
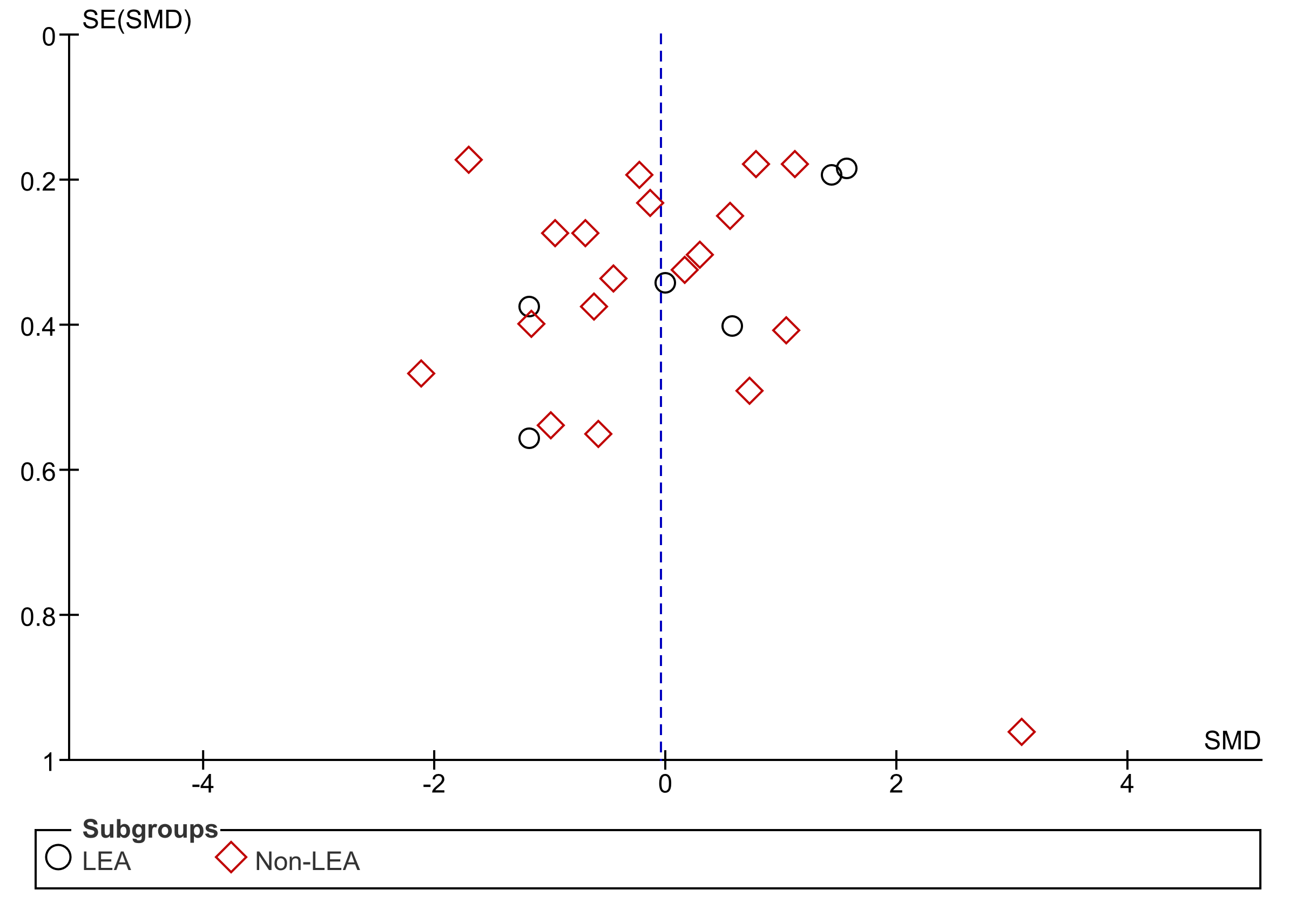

2. Cunningham (1980) (FFM) – Athlete Status


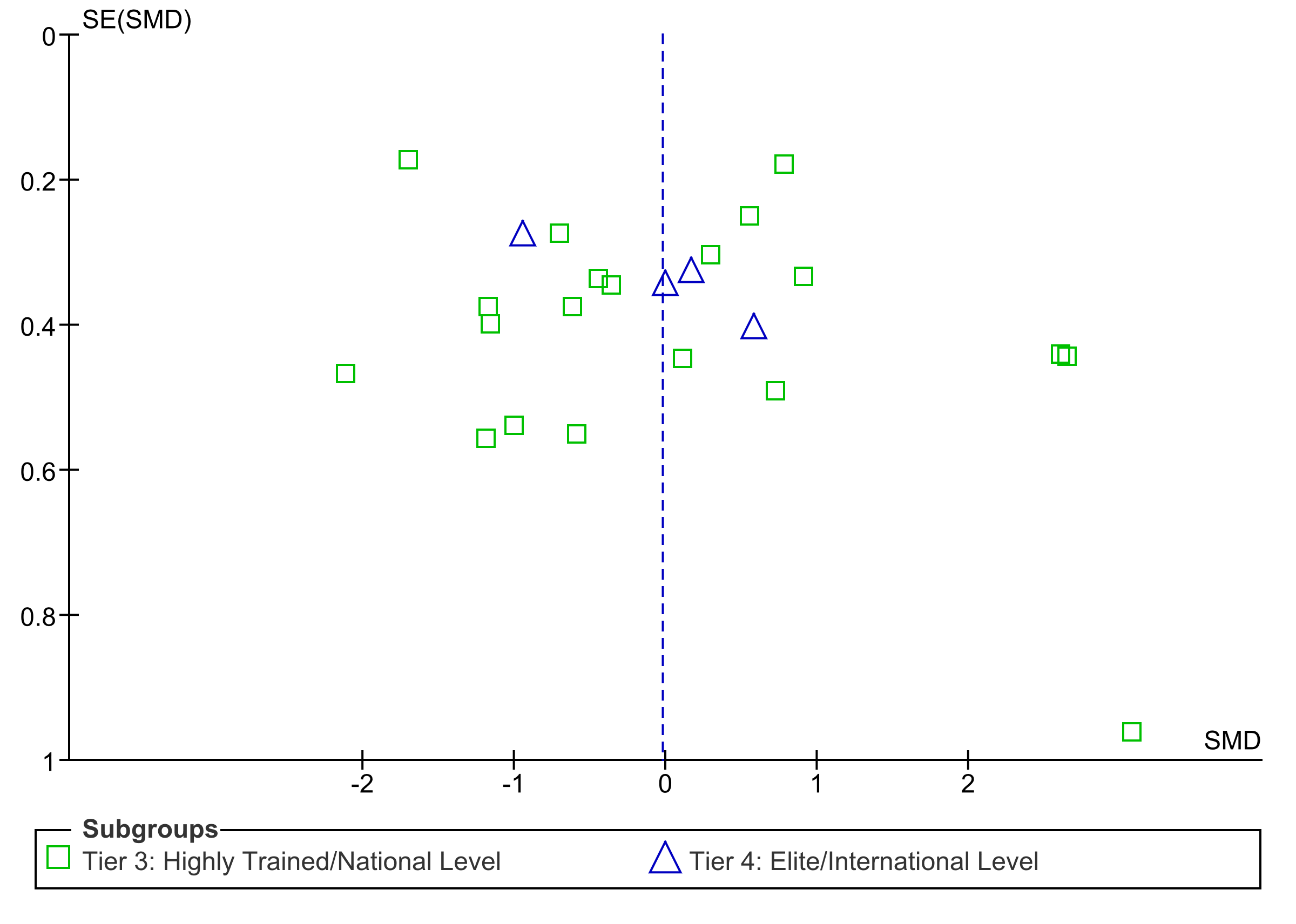


1. Cunningham (1980) (FFM) – Average Weight >/< 62.7kg Females


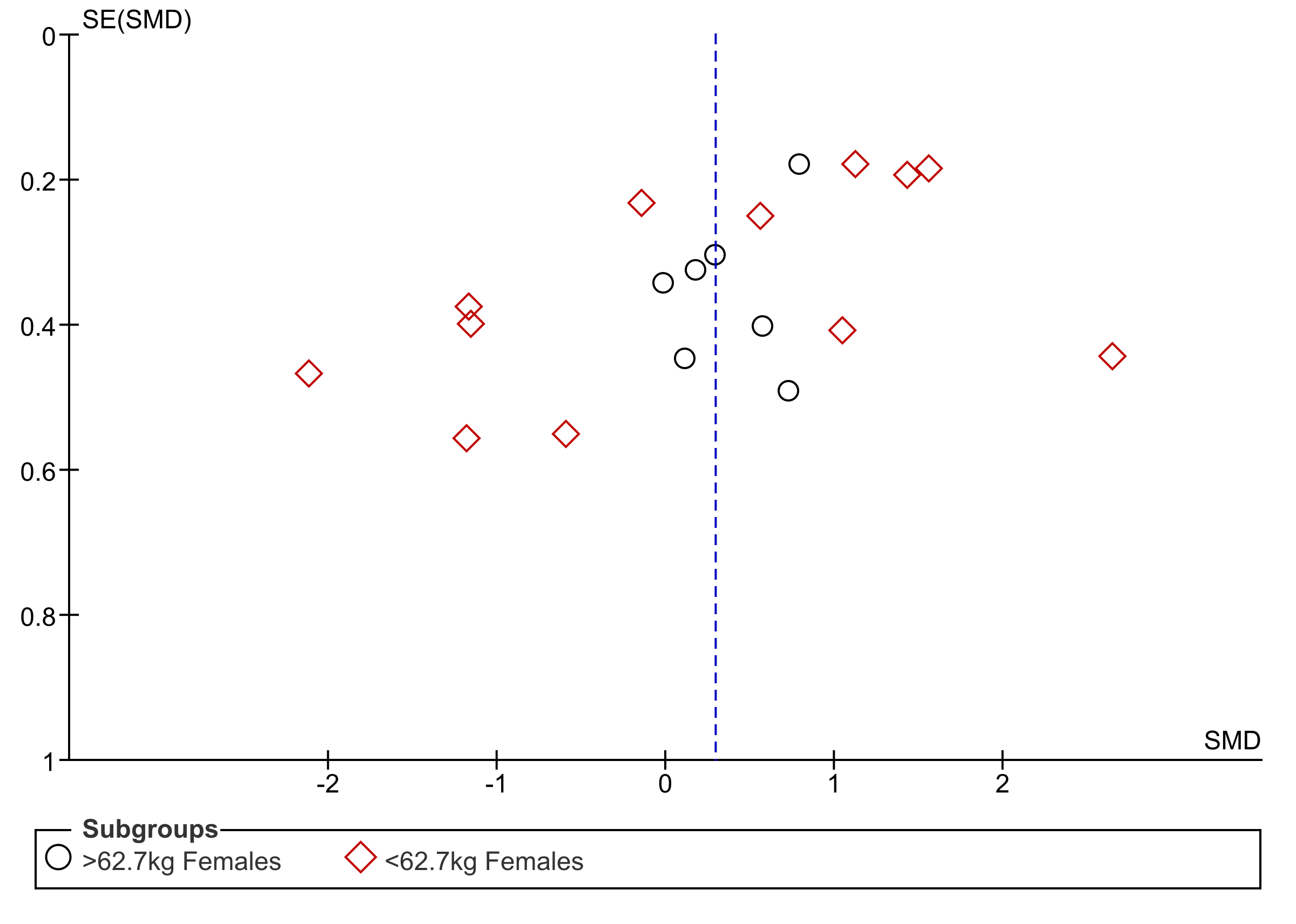


1. Cunningham (1980) (FFM) – Average Weight >/<78.9kg Males


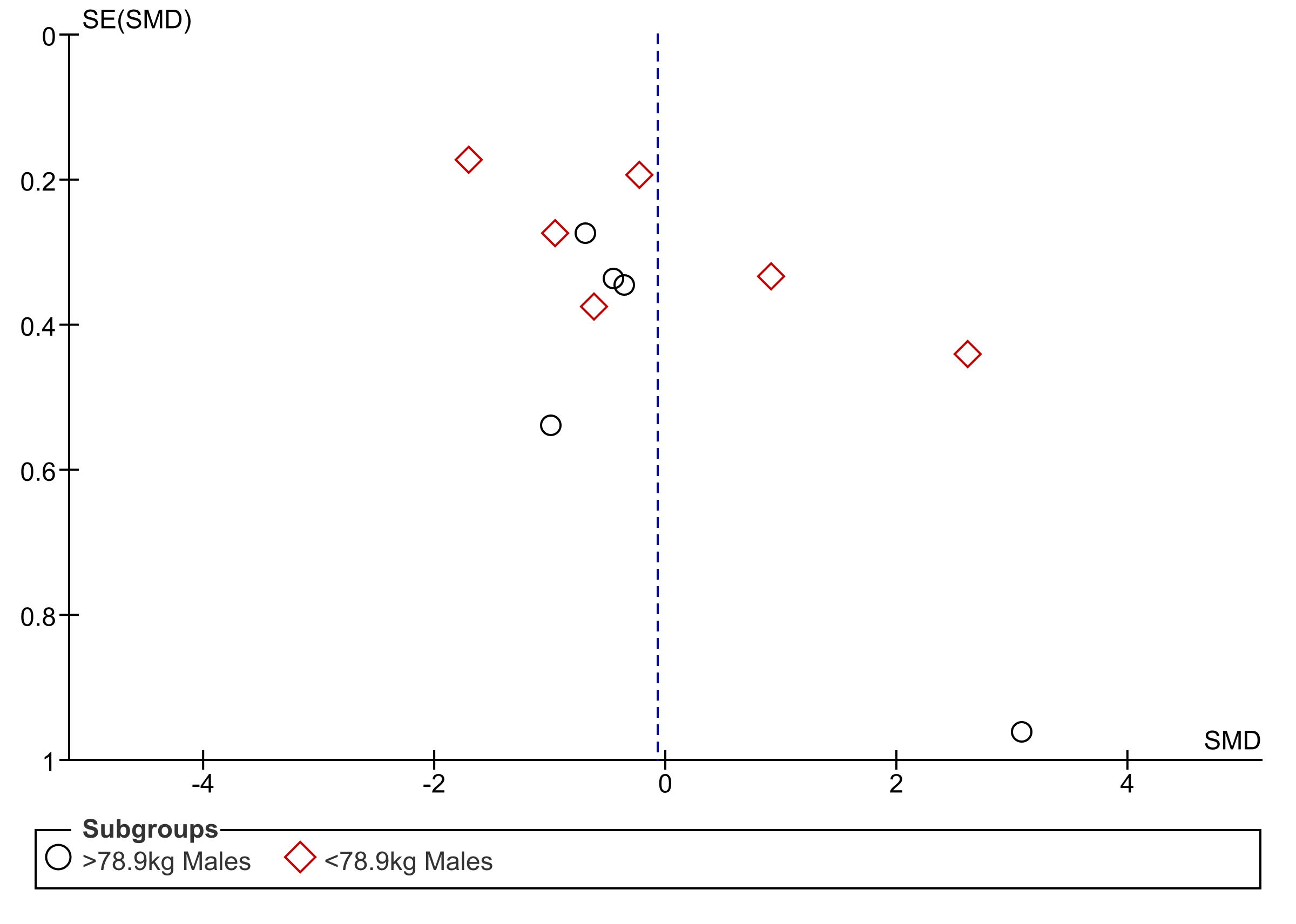


1. Cunningham (1980) (FFM) – 24hr Physical Activity Abstinence


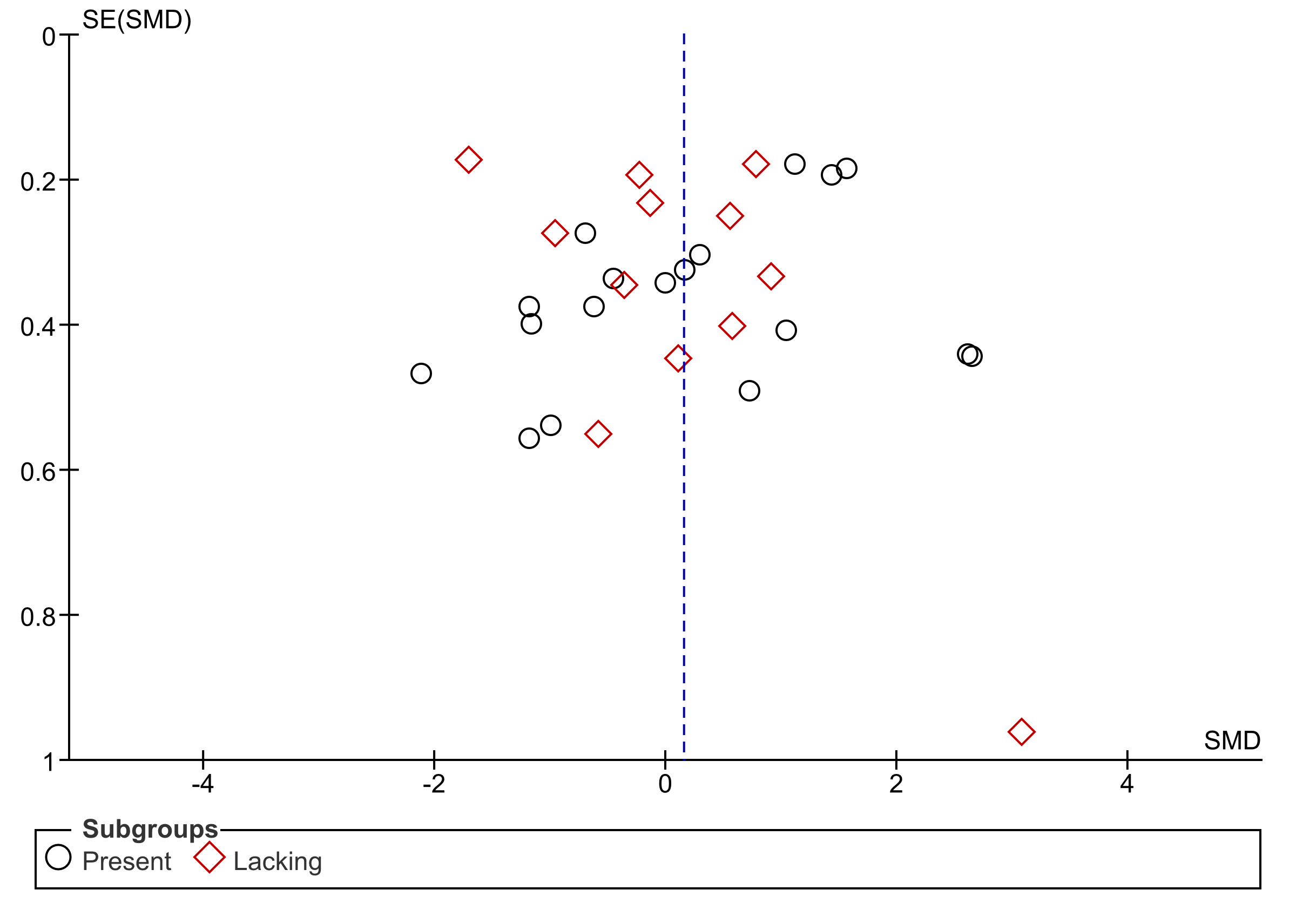


1. Cunningham (1980) (FFM) – Discard, Steady State, & Validated Extraction Method


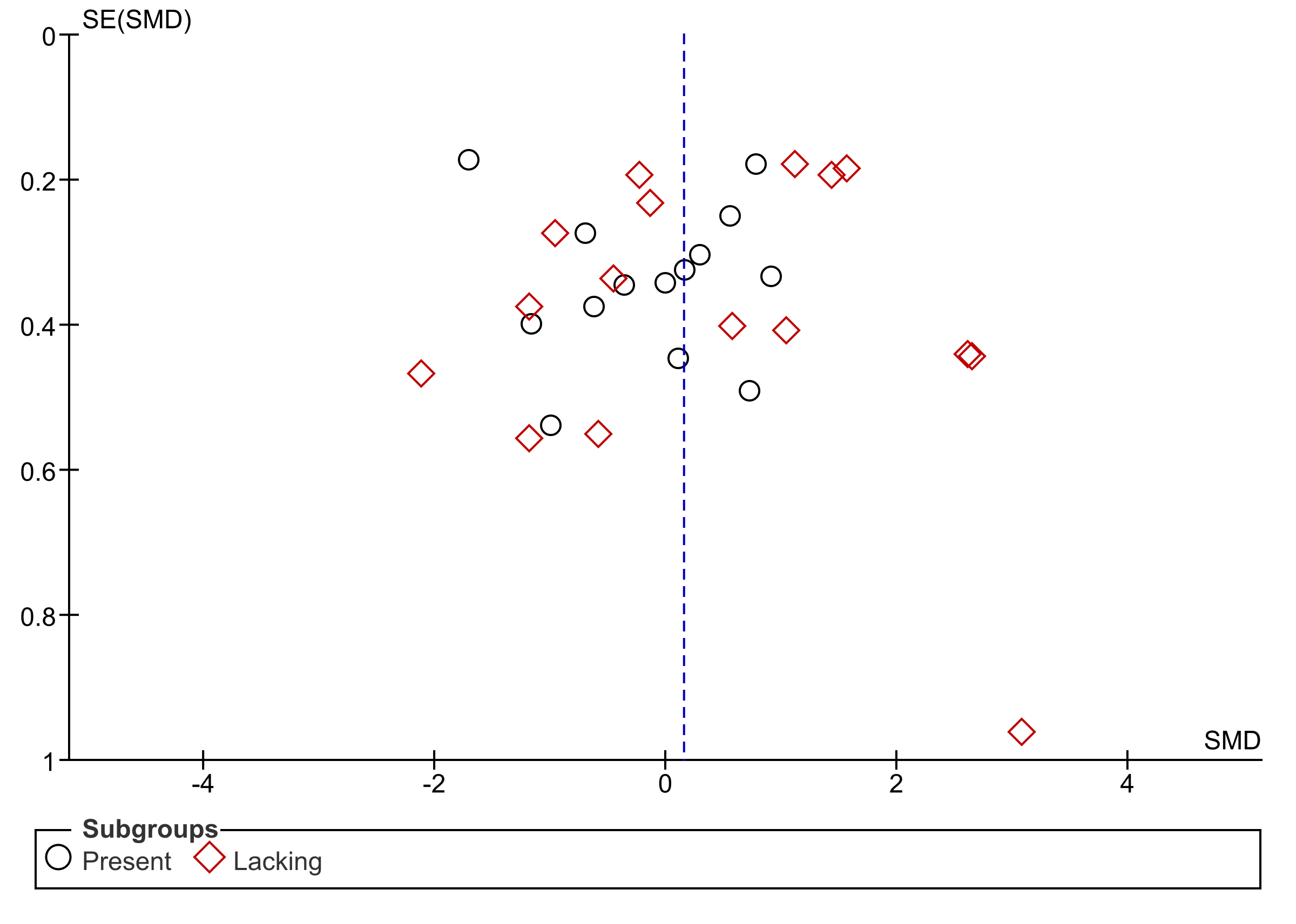


1. Cunningham (1980) (FFM) – Pre-test Rest vs No Rest


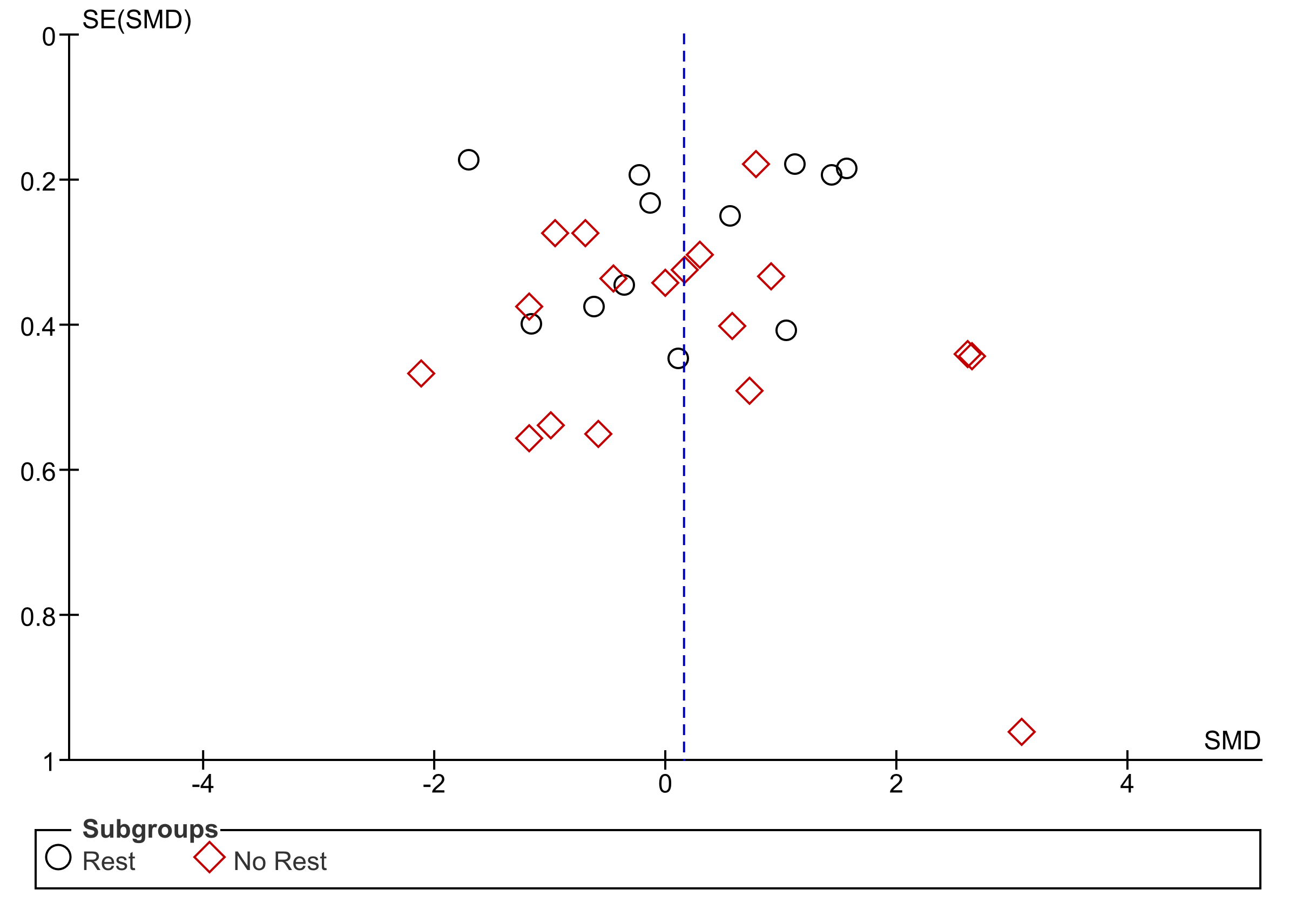


1. Cunningham (1980) (FFM) – Nutritional/Stimulants Status


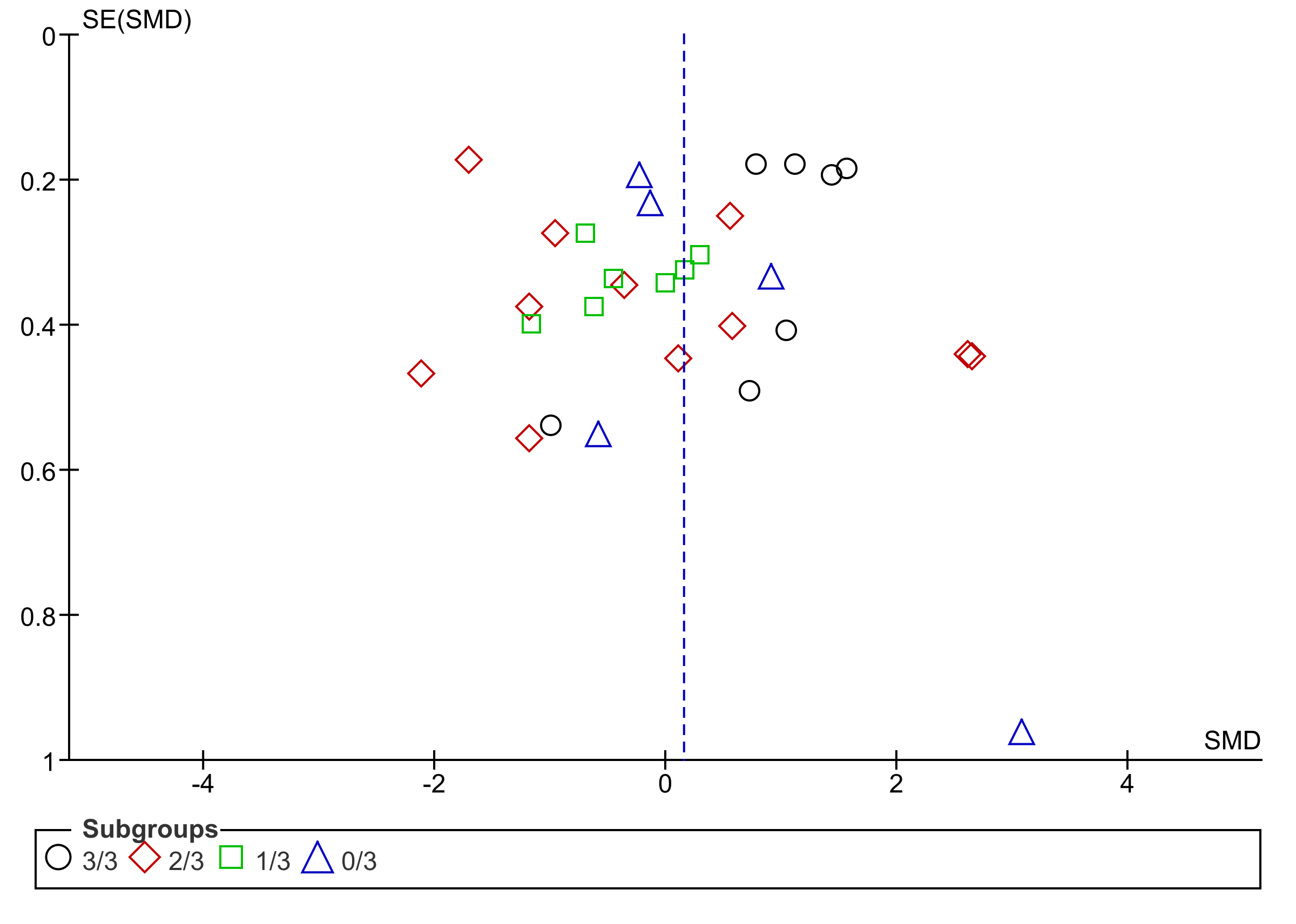


**Accuracy MA - Funnel plots for Harris-Benedict (1918) (age, weight, height)**

1. Harris-Benedict (1918) (age, weight, height)
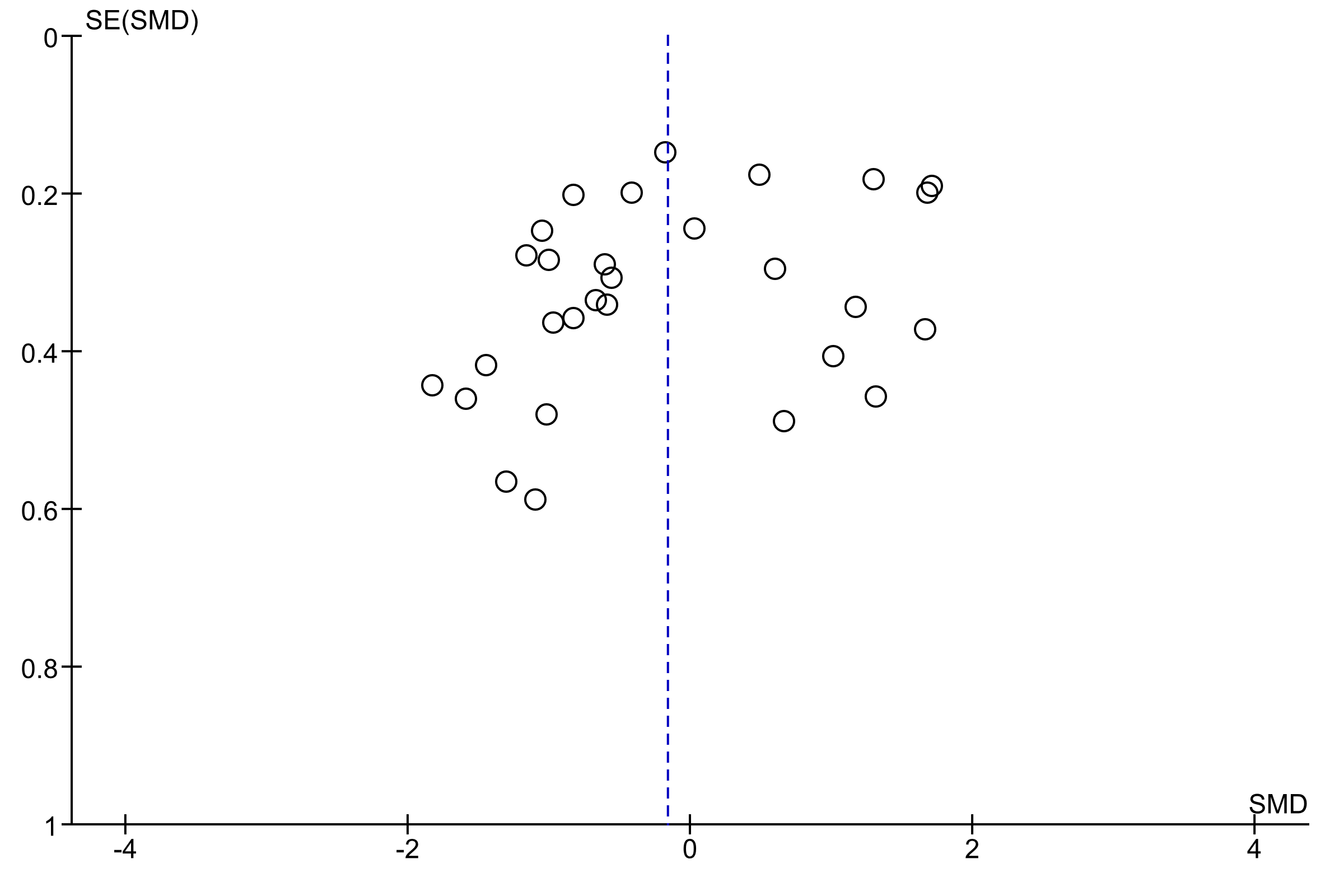

2. Harris-Benedict (1918) (age, weight, height) – Sex


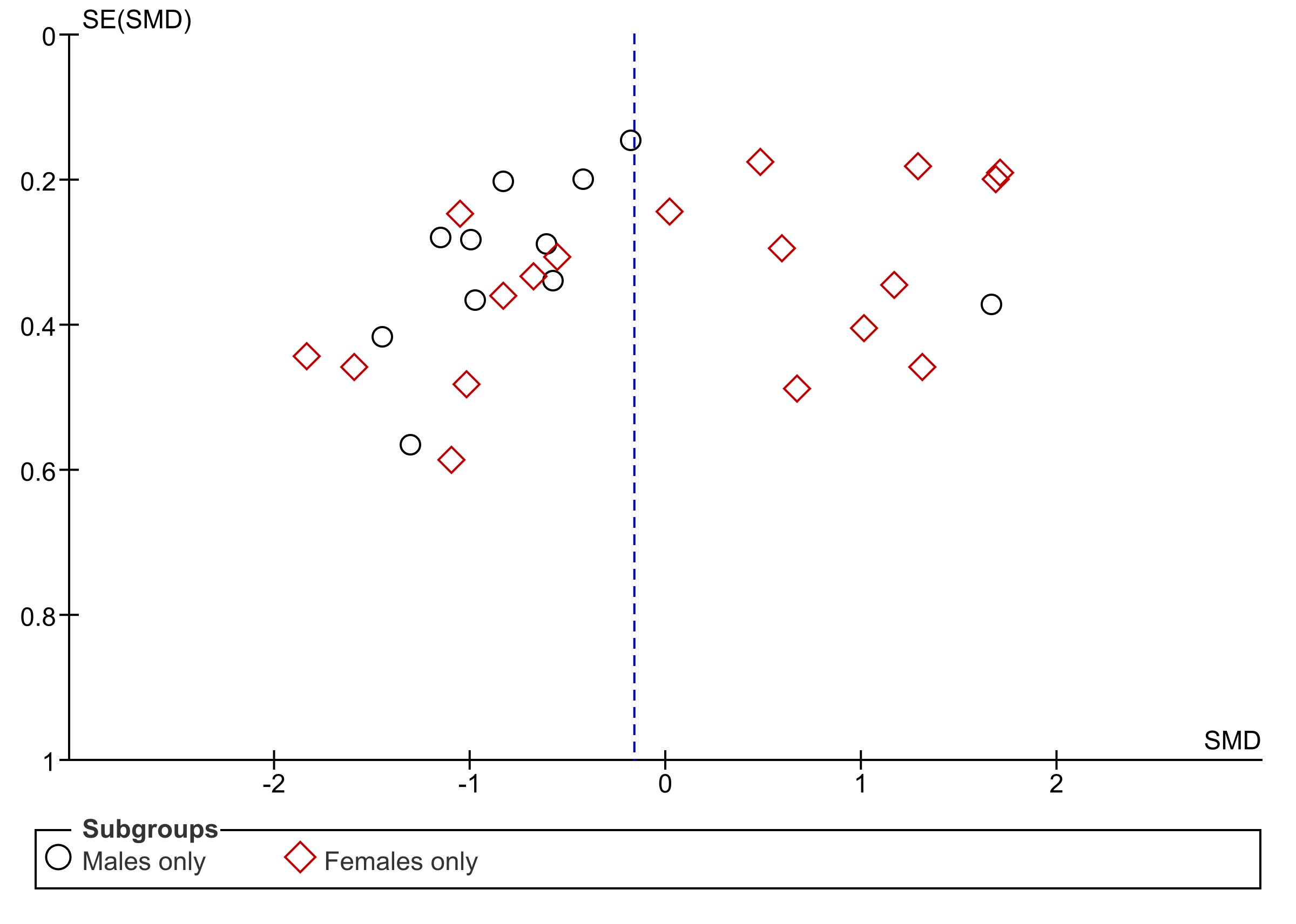


1. Harris-Benedict (1918) (age, weight, height) – Athlete Status


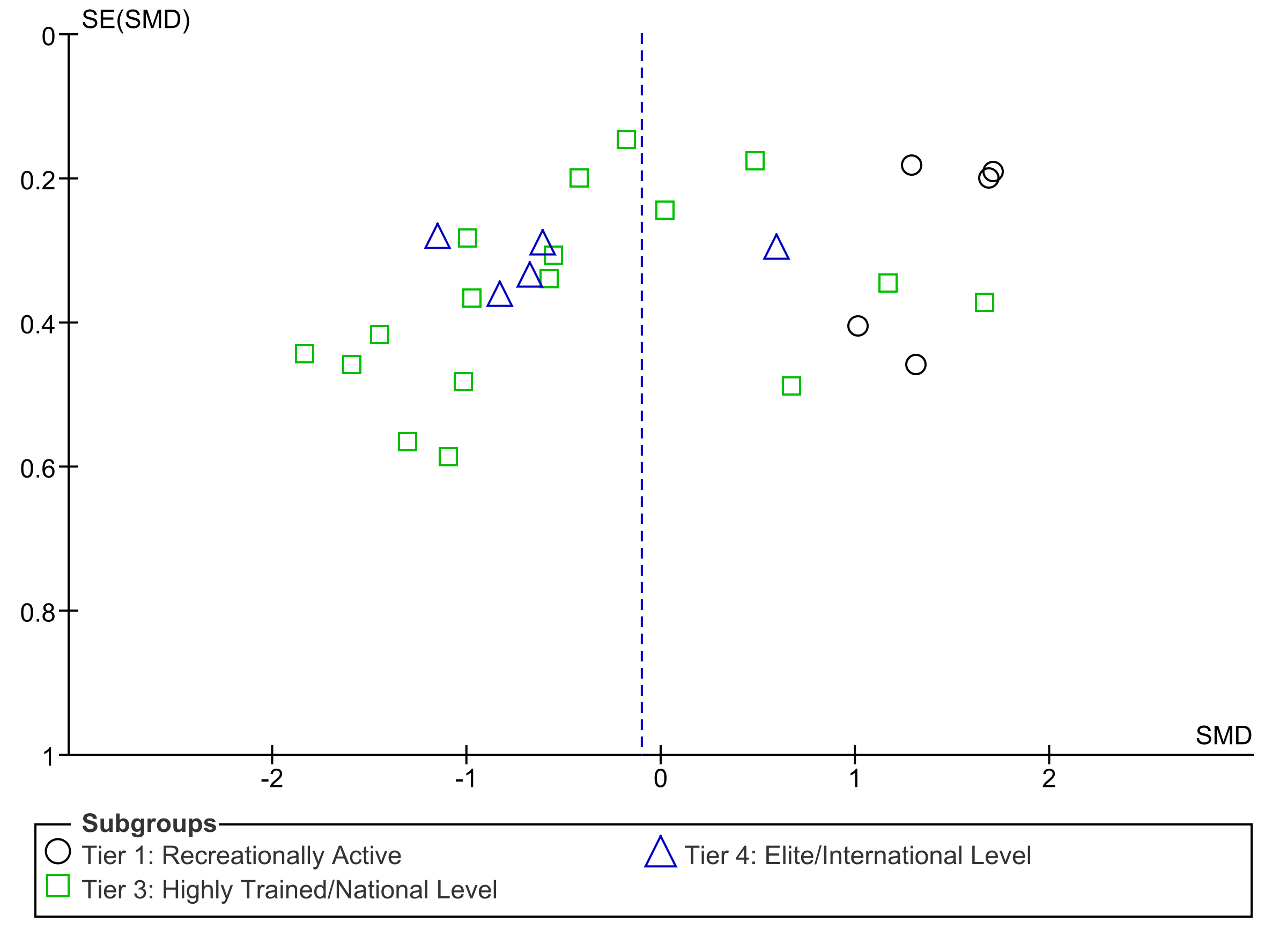


1. Harris-Benedict (1918) (age, weight, height) – Average Weight >/< 62.7kg Females


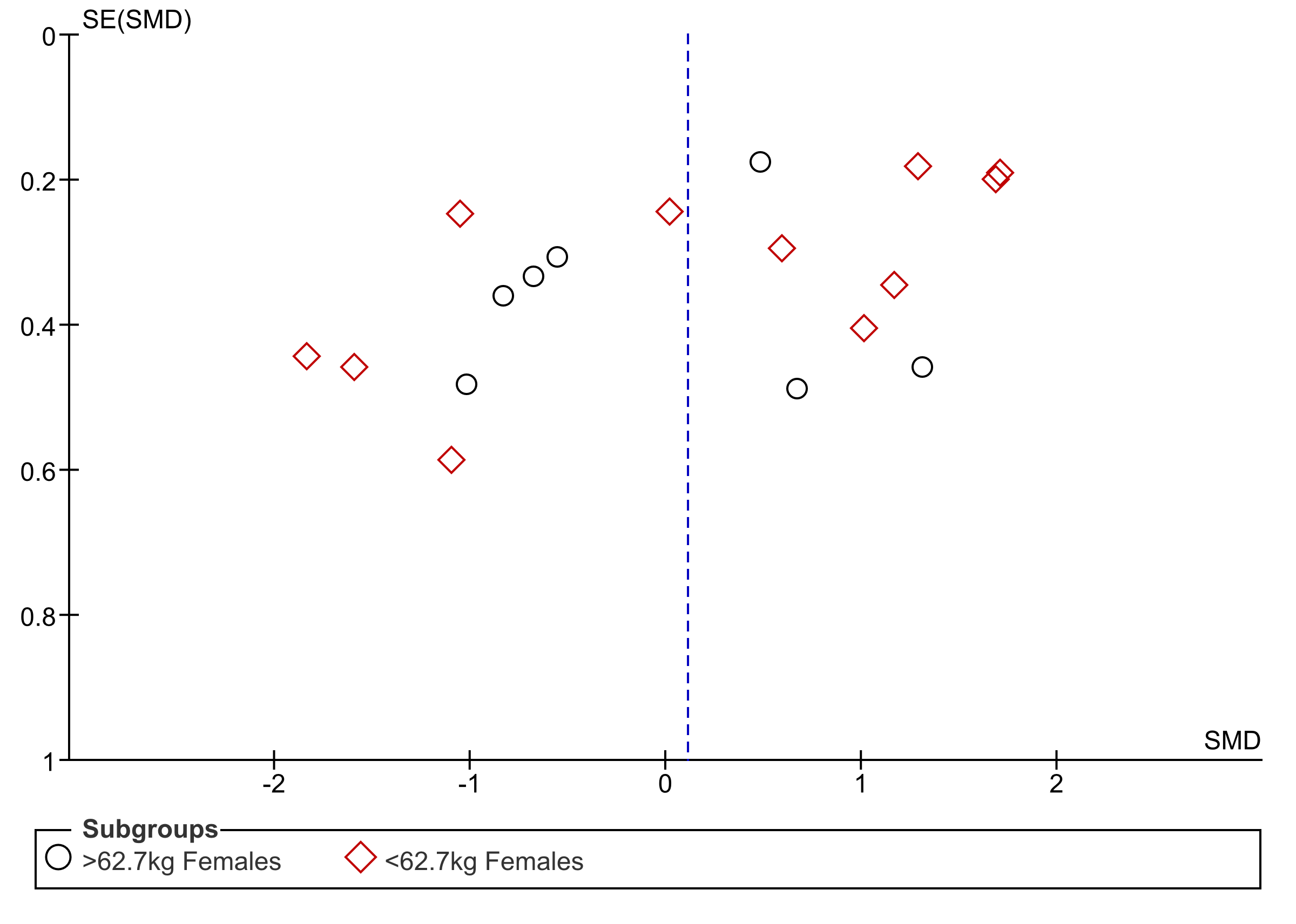


1. Harris-Benedict (1918) (age, weight, height) – Average Weight >/<78.9kg Males


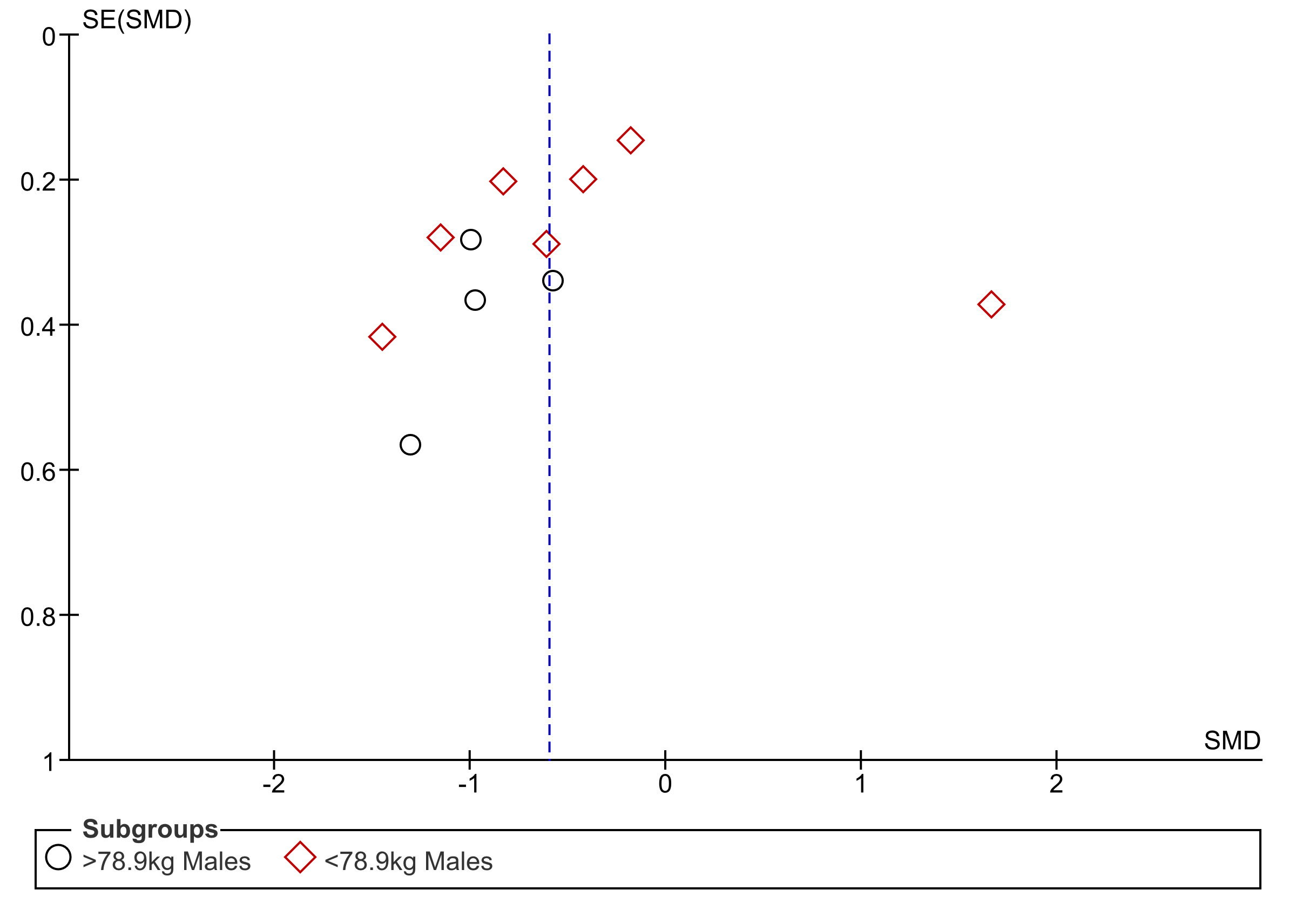


1. Harris-Benedict (1918) (age, weight, height) – 24hr Physical Activity Abstinence


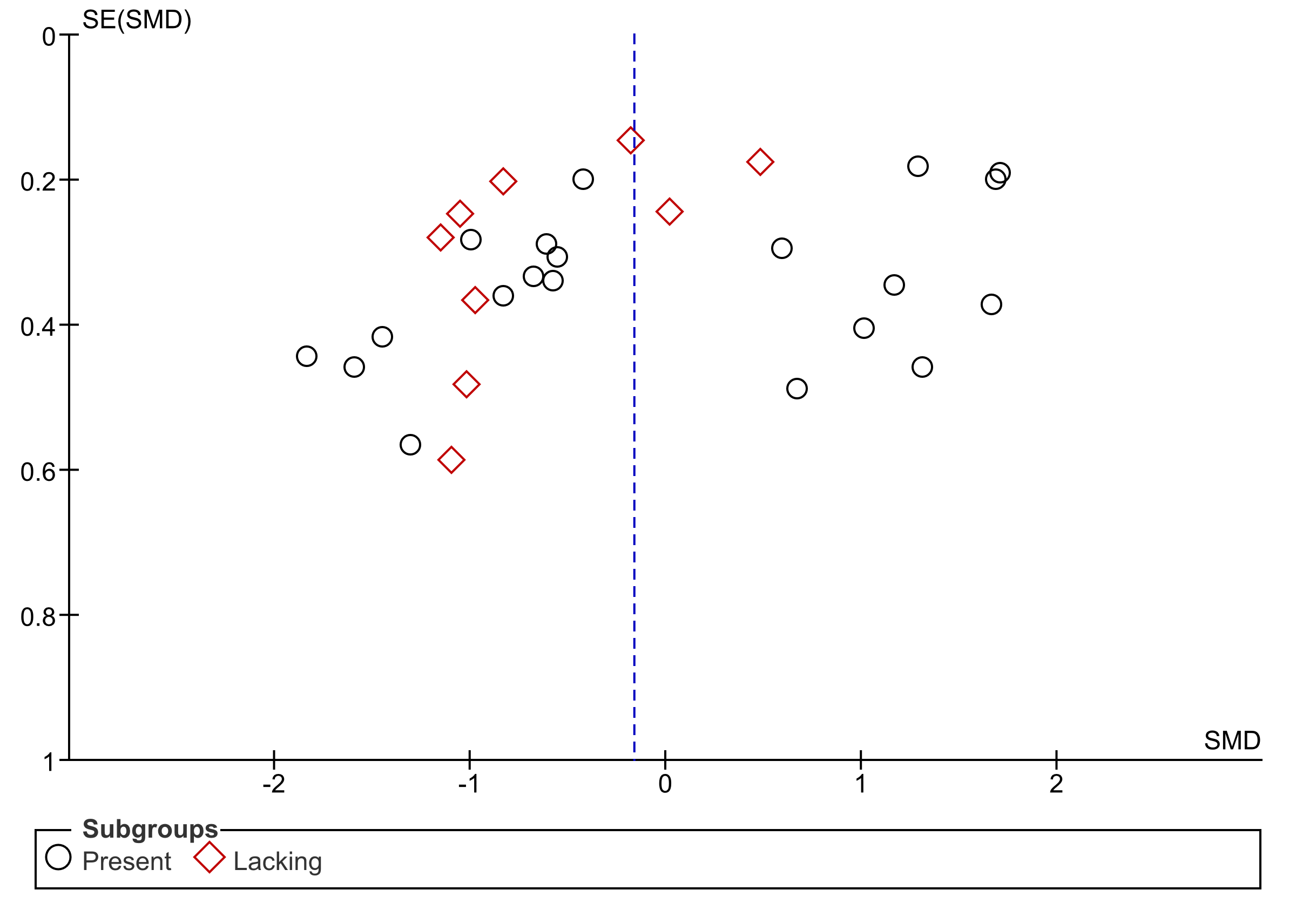


1. Harris-Benedict (1918) (age, weight, height) – Discard, Steady State, & Validated Extraction Method


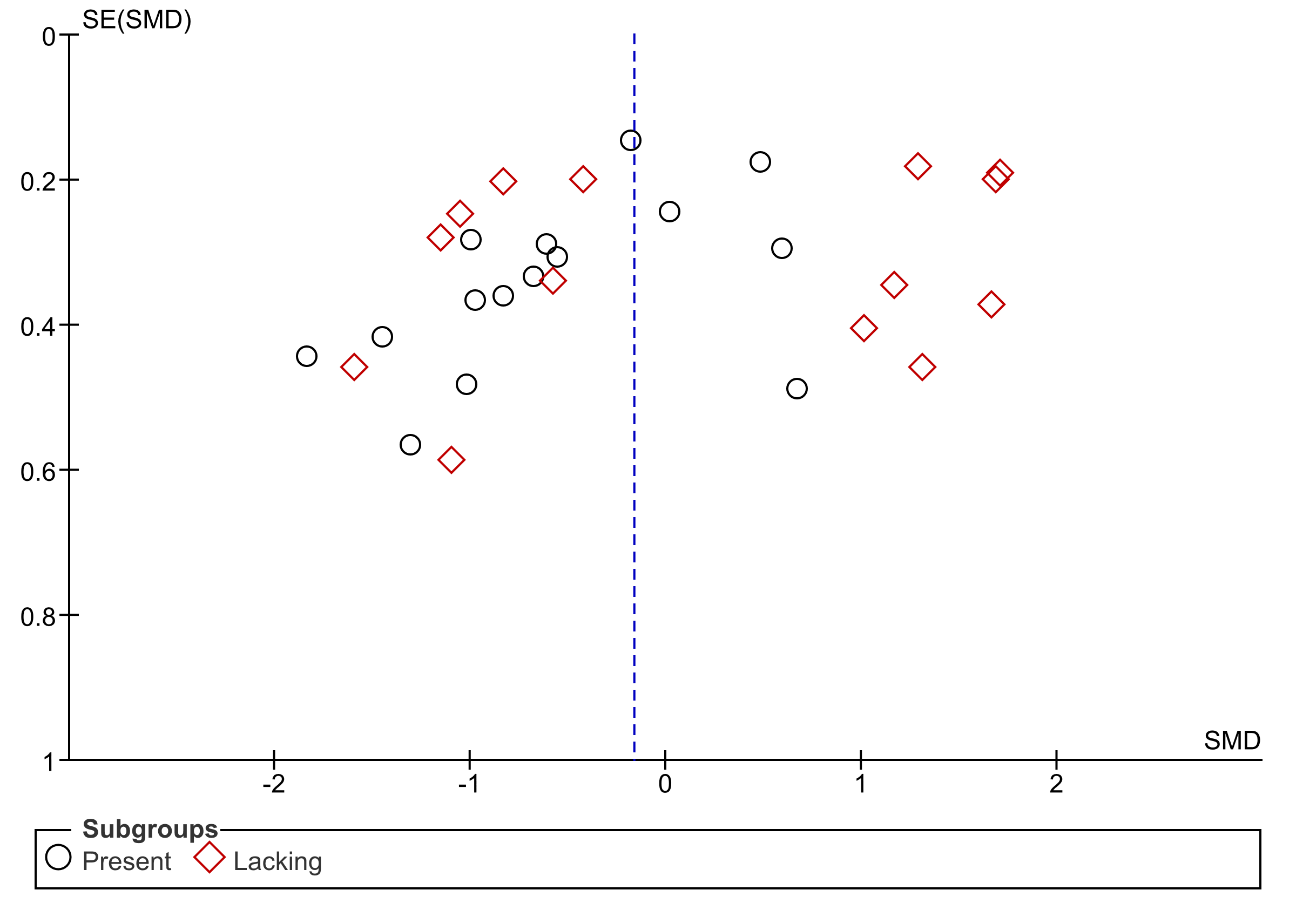


1. Harris-Benedict (1918) (age, weight, height) – Pre-test Rest vs No Rest


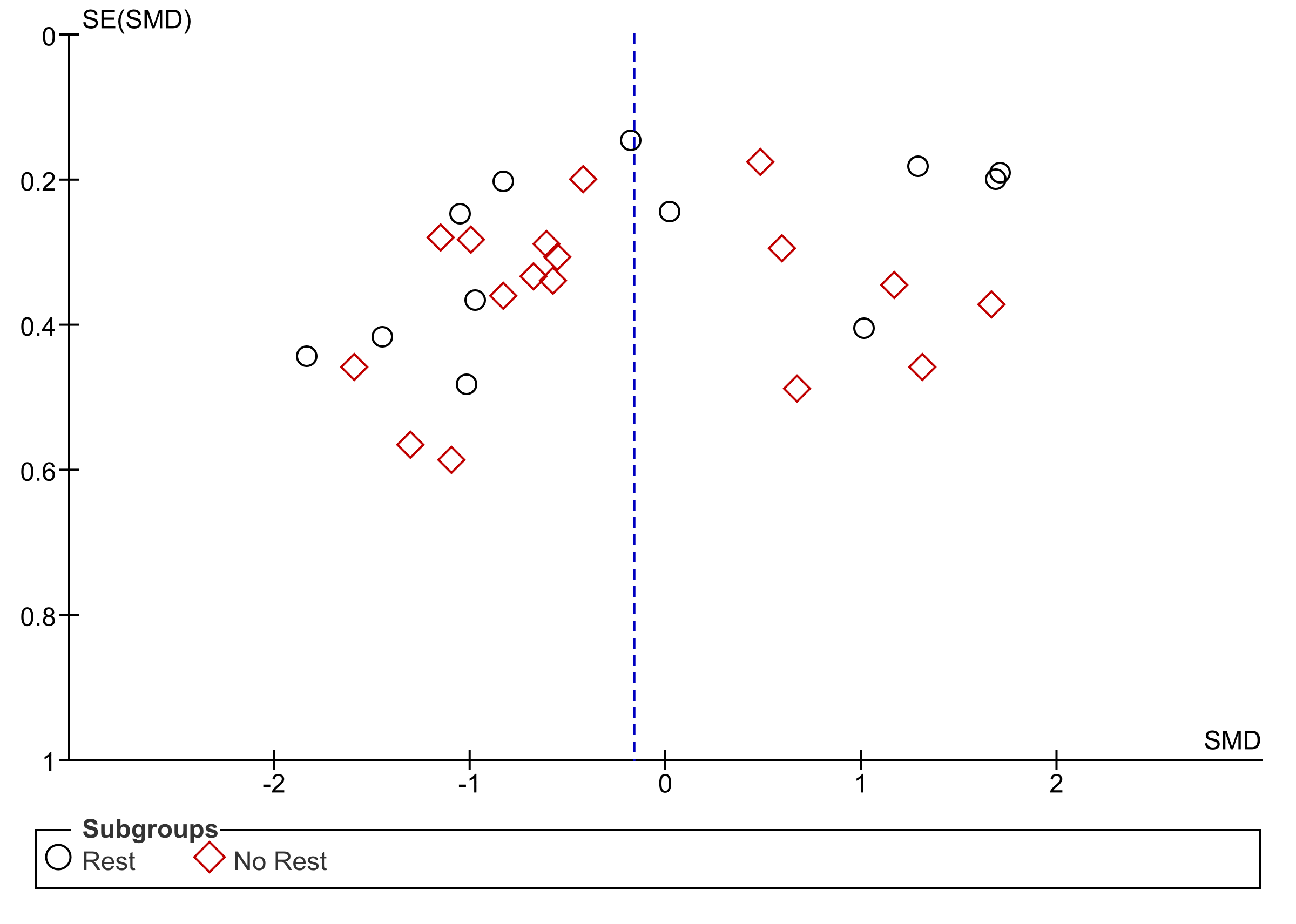


1. Harris-Benedict (1918) (age, weight, height) – Nutritional/Stimulants Status


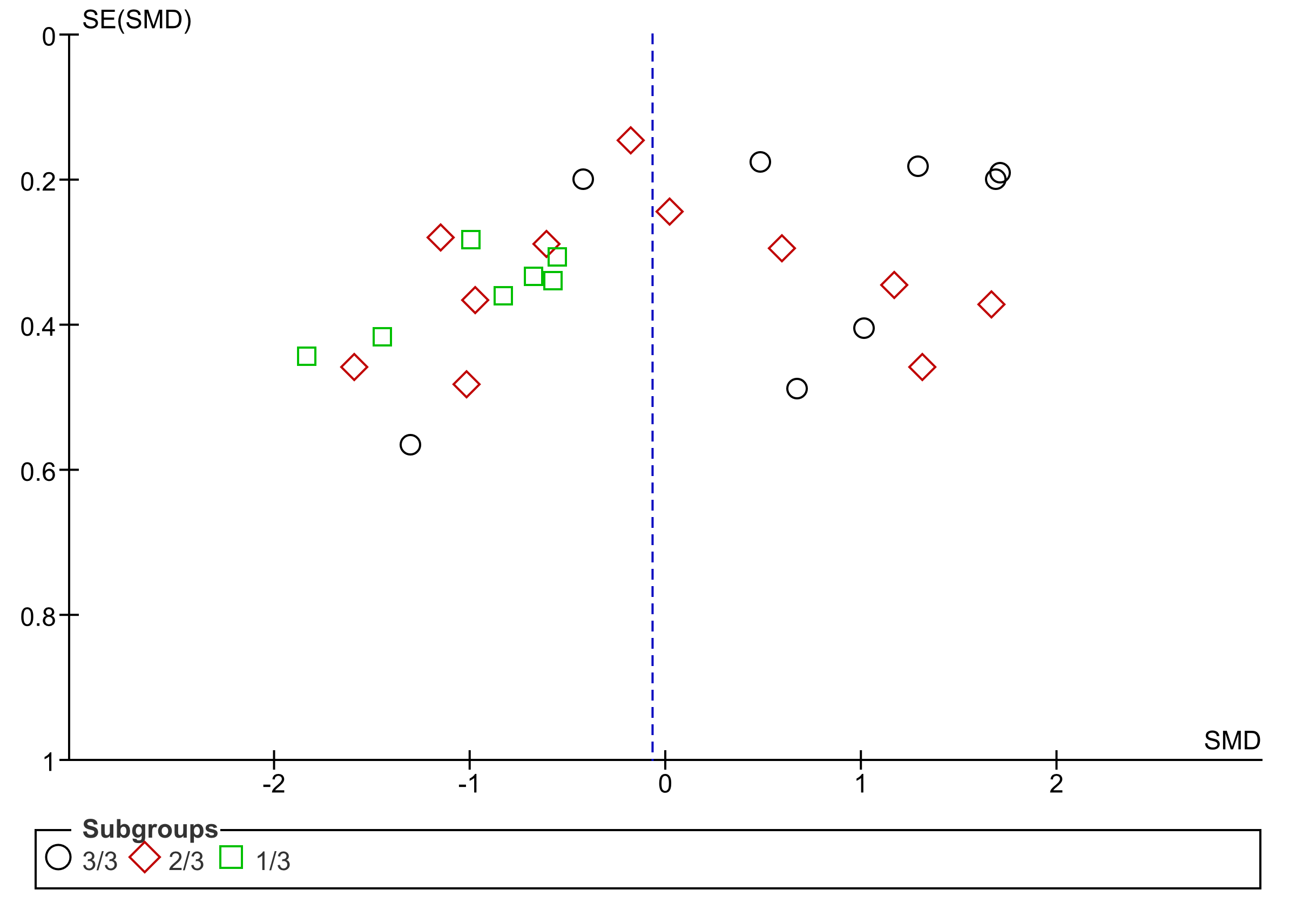


**Accuracy MA - Funnel plots for Cunningham (1991) (FFM)**

1. Cunningham (1991) (FFM)


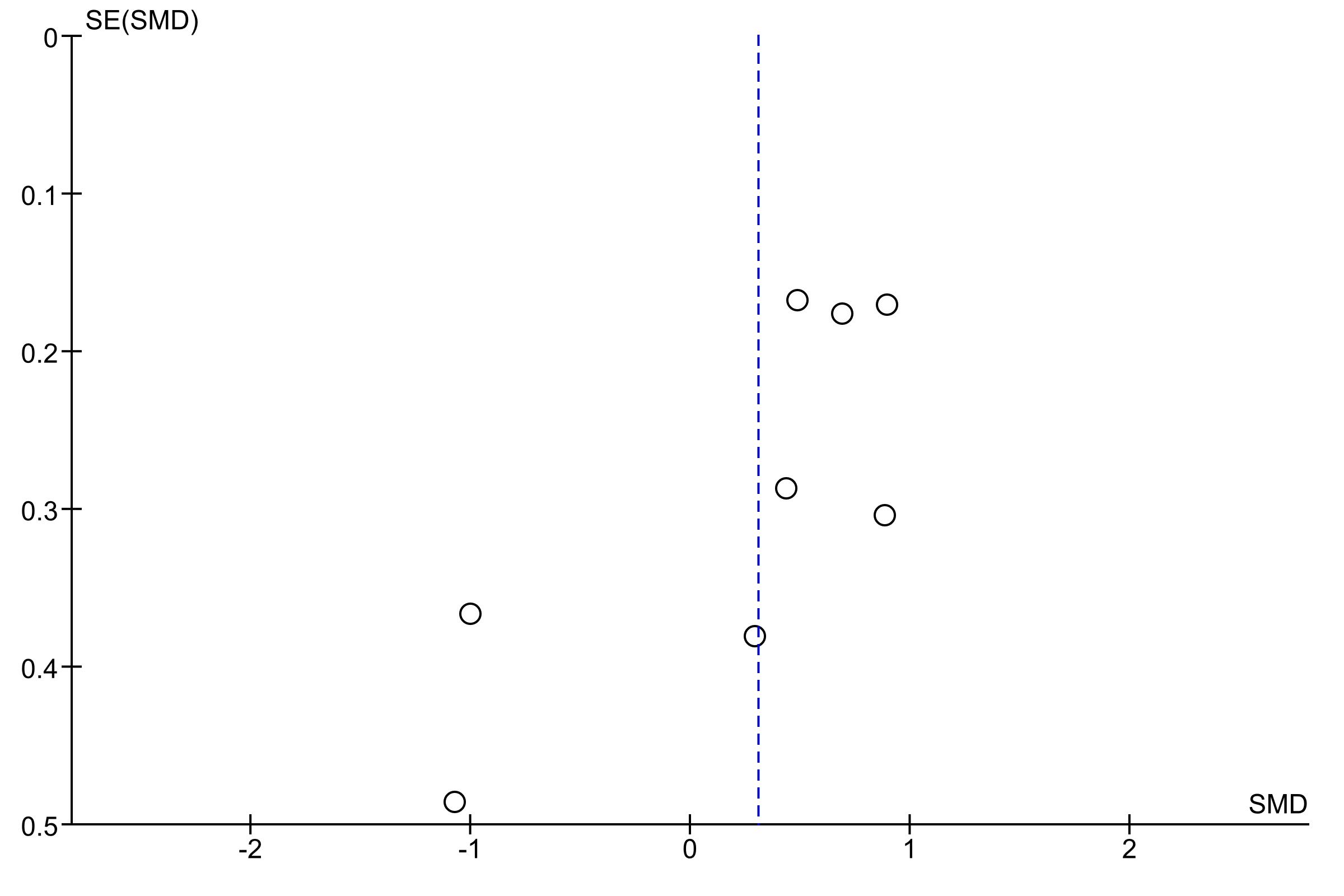


**Accuracy MA - Funnel plots for Mifflin St. Jeor (1990) (age, weight, height)**

1. Mifflin St. Jeor (1990) (age, weight, height)


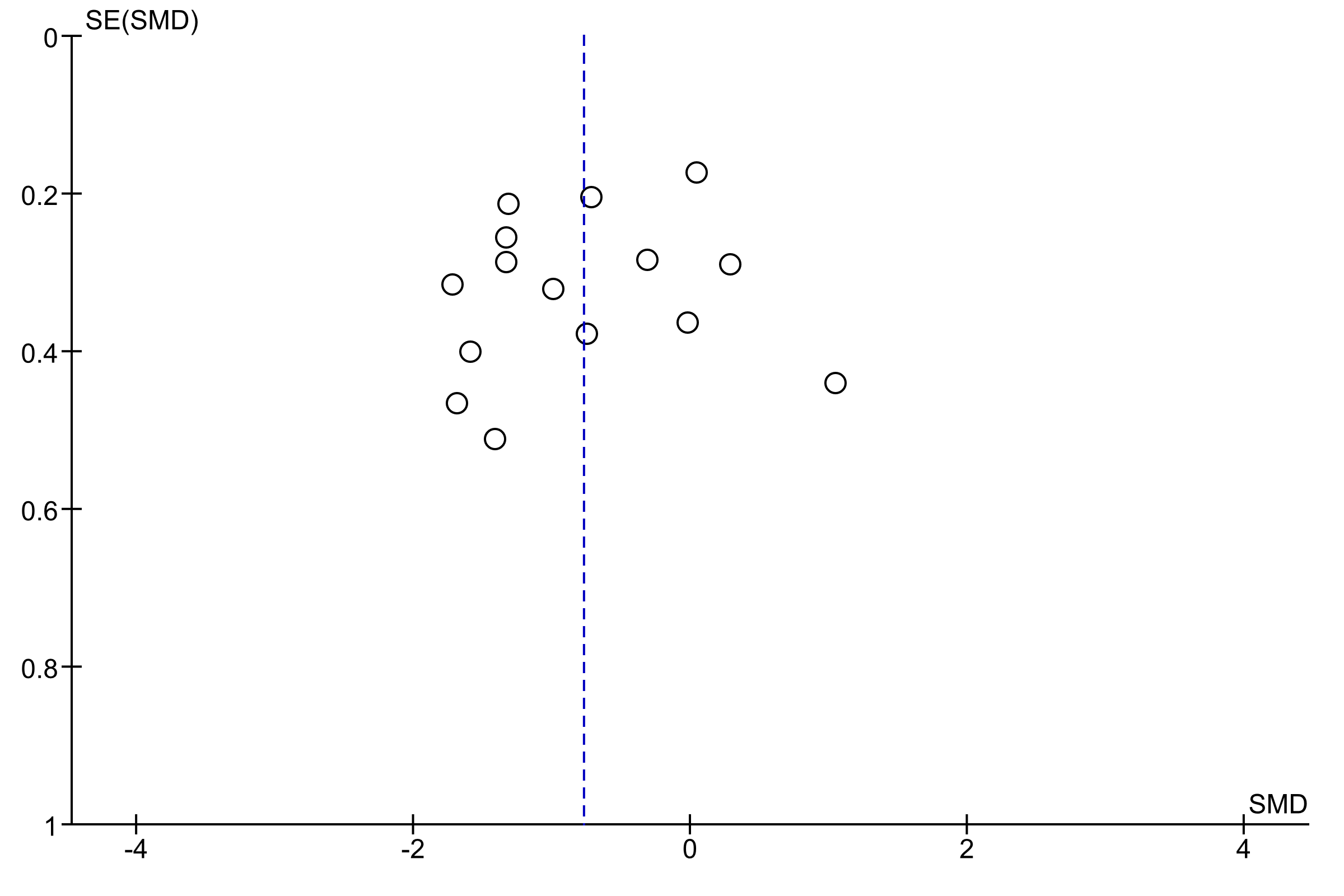


1. Mifflin St. Jeor (1990) (age, weight, height) – Sex


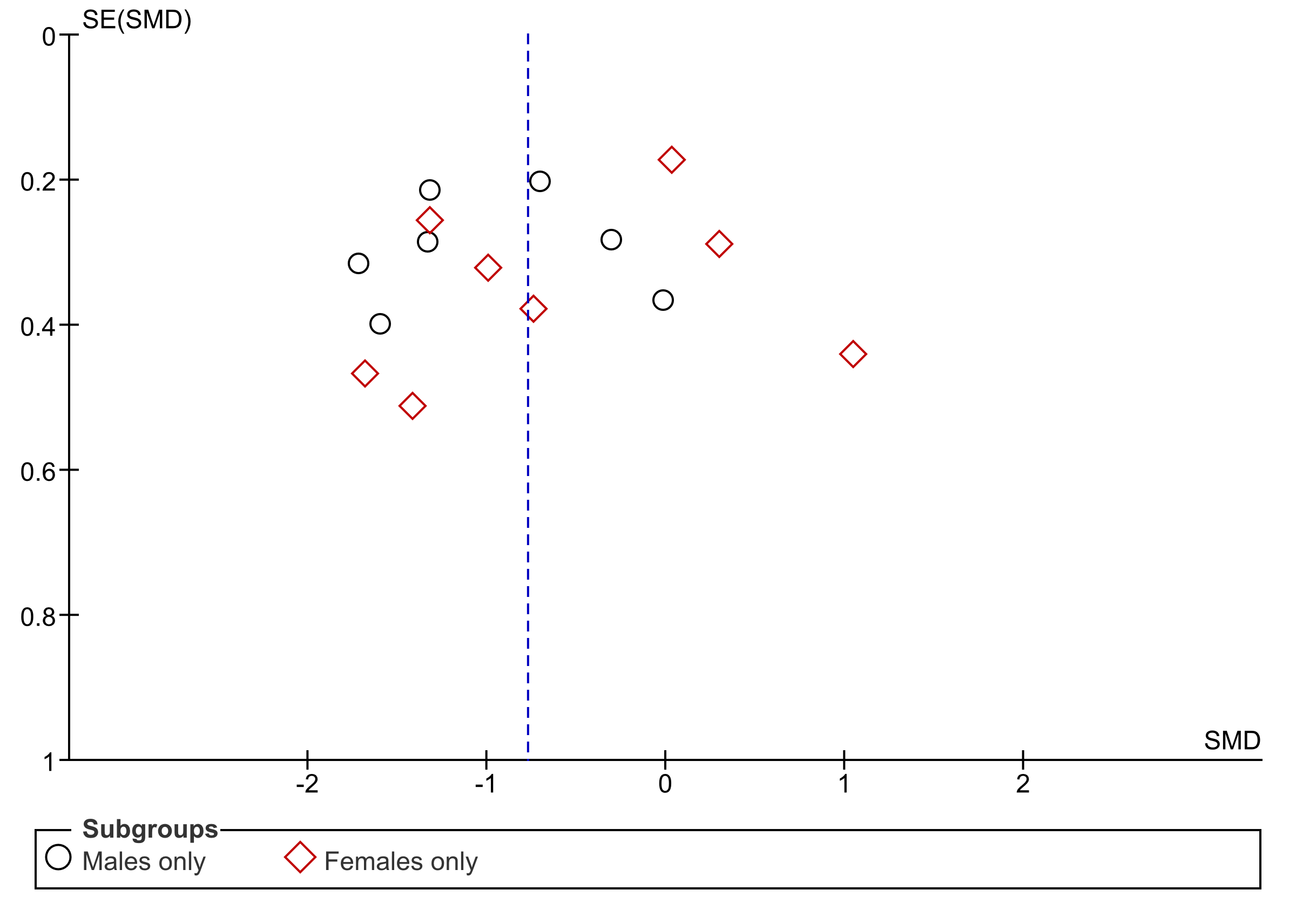


1. Mifflin St. Jeor (1990) (age, weight, height) – Average Weight >/< 62.7kg Females


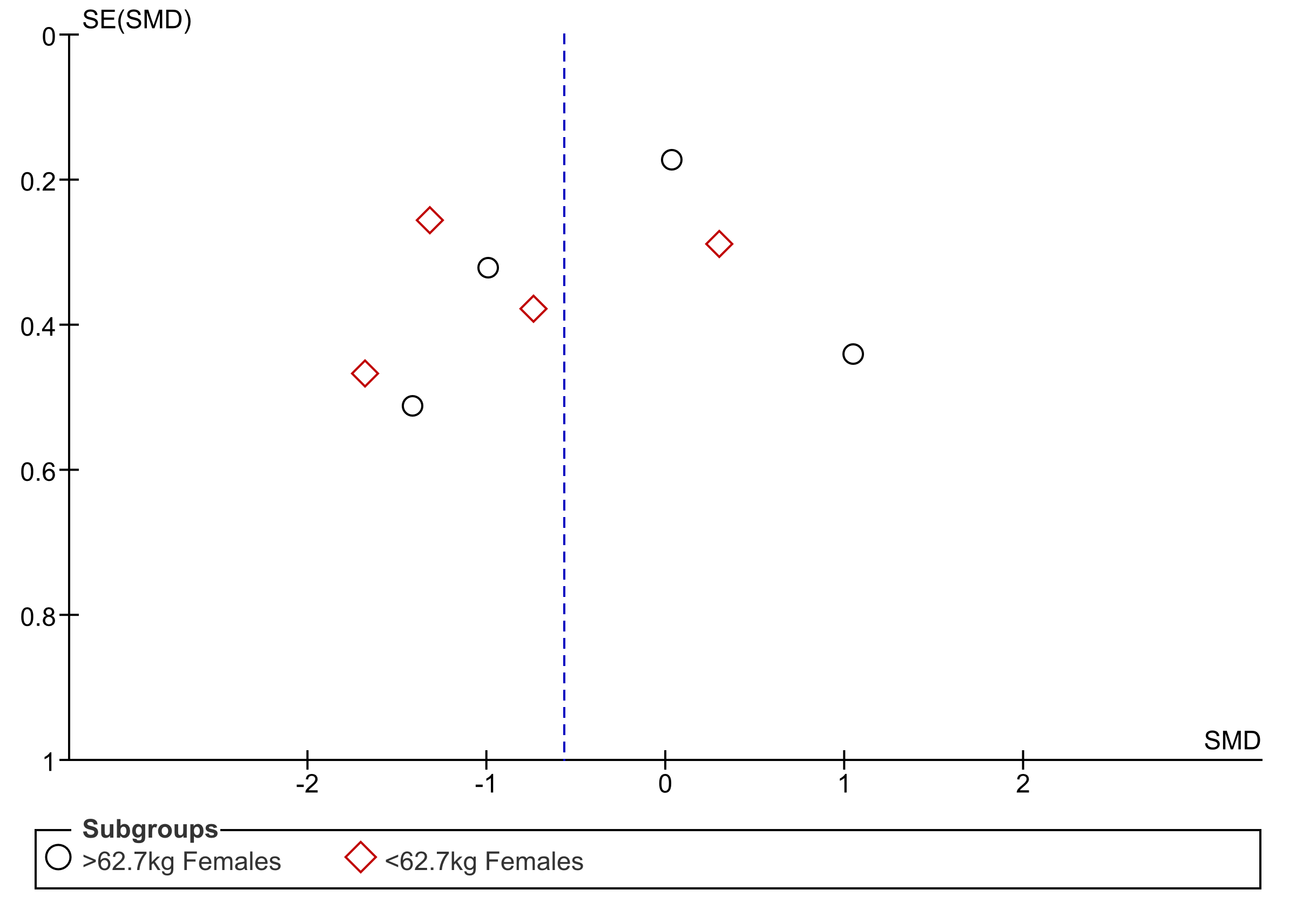


1. Mifflin St. Jeor (1990) (age, weight, height) – 24hr Physical Activity Abstinence


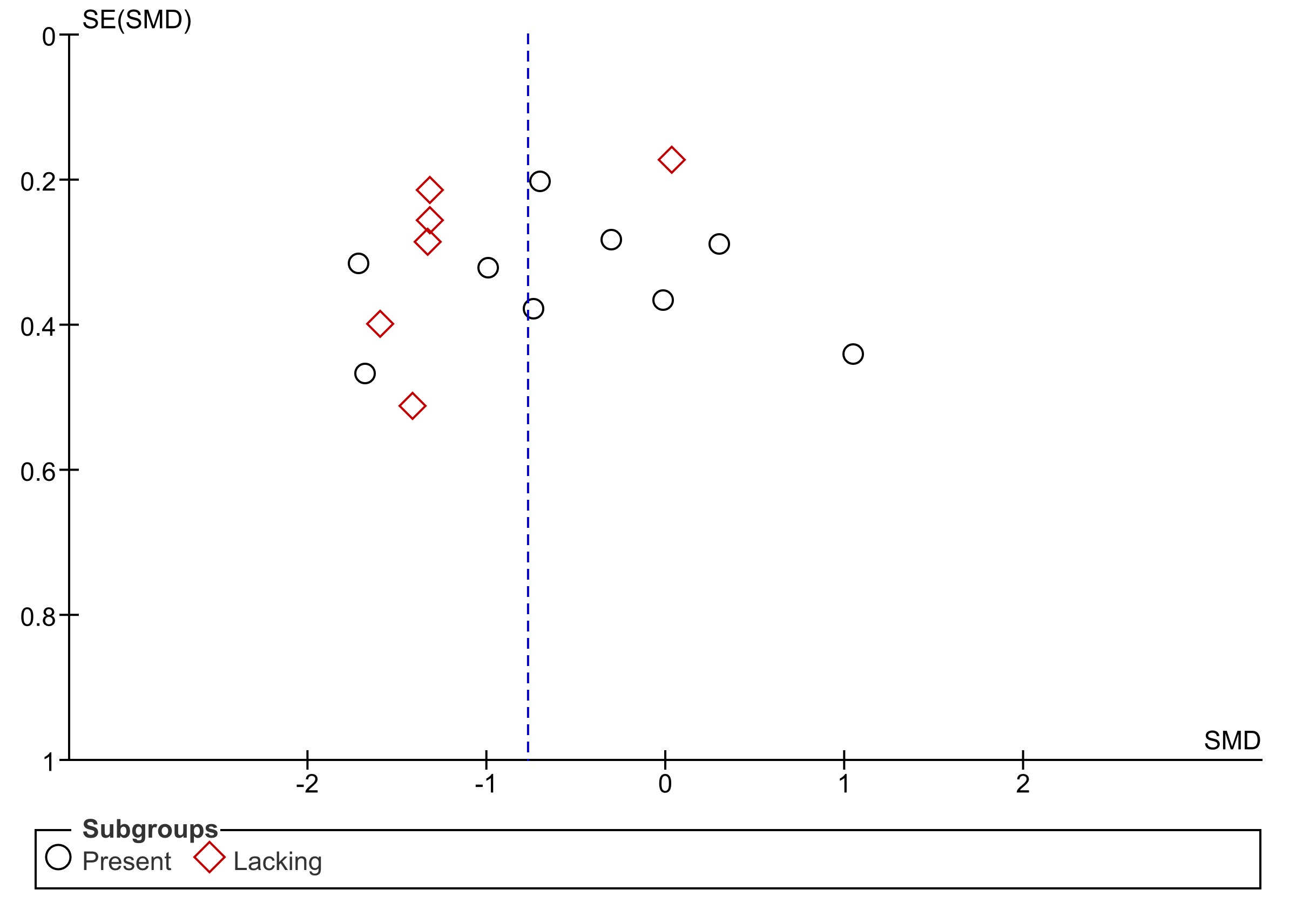


1. Mifflin St. Jeor (1990) (age, weight, height) – Discard, Steady State, & Validated Extraction Method


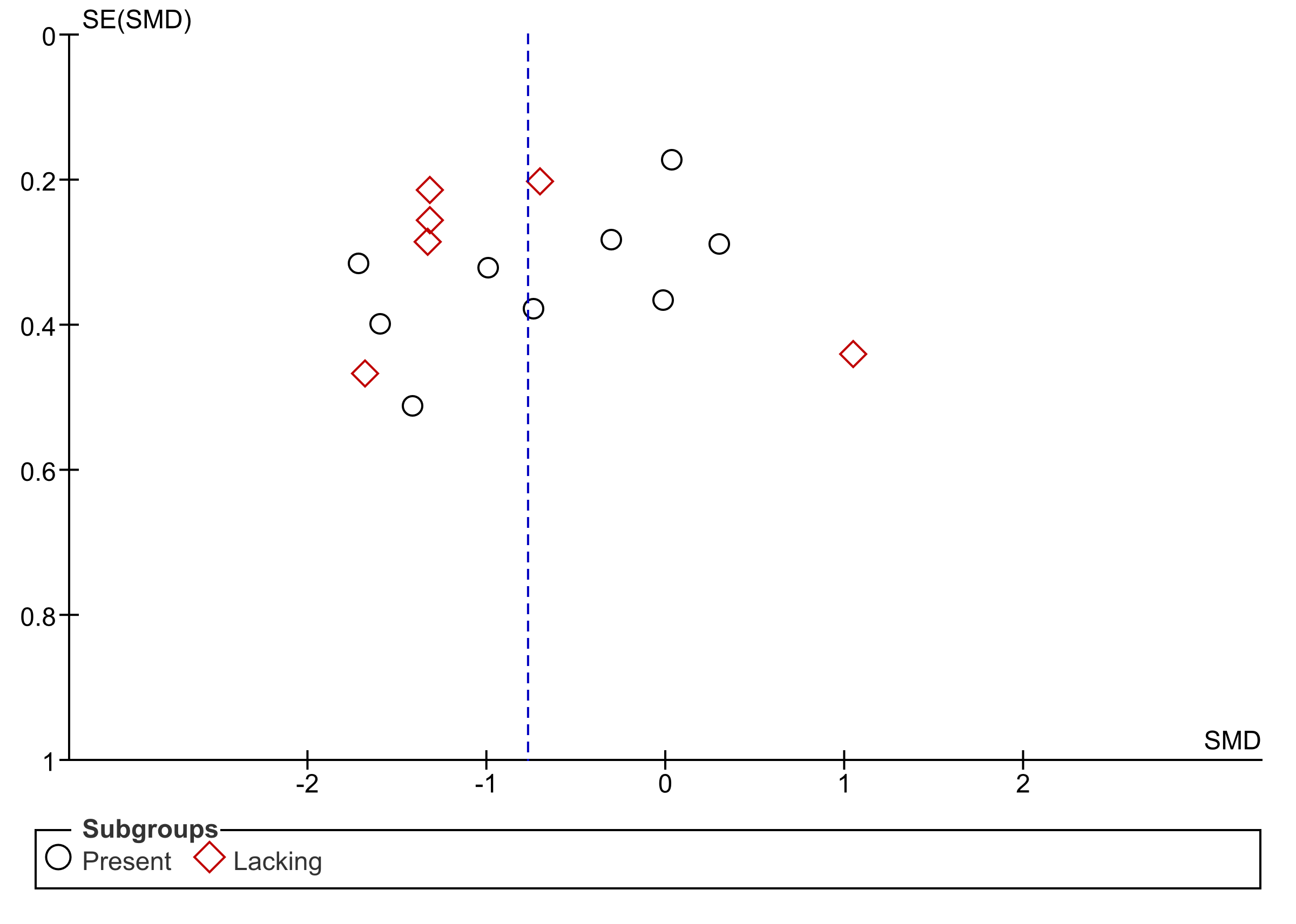


1. Mifflin St. Jeor (1990) (age, weight, height) – Pre-test Rest vs No Rest


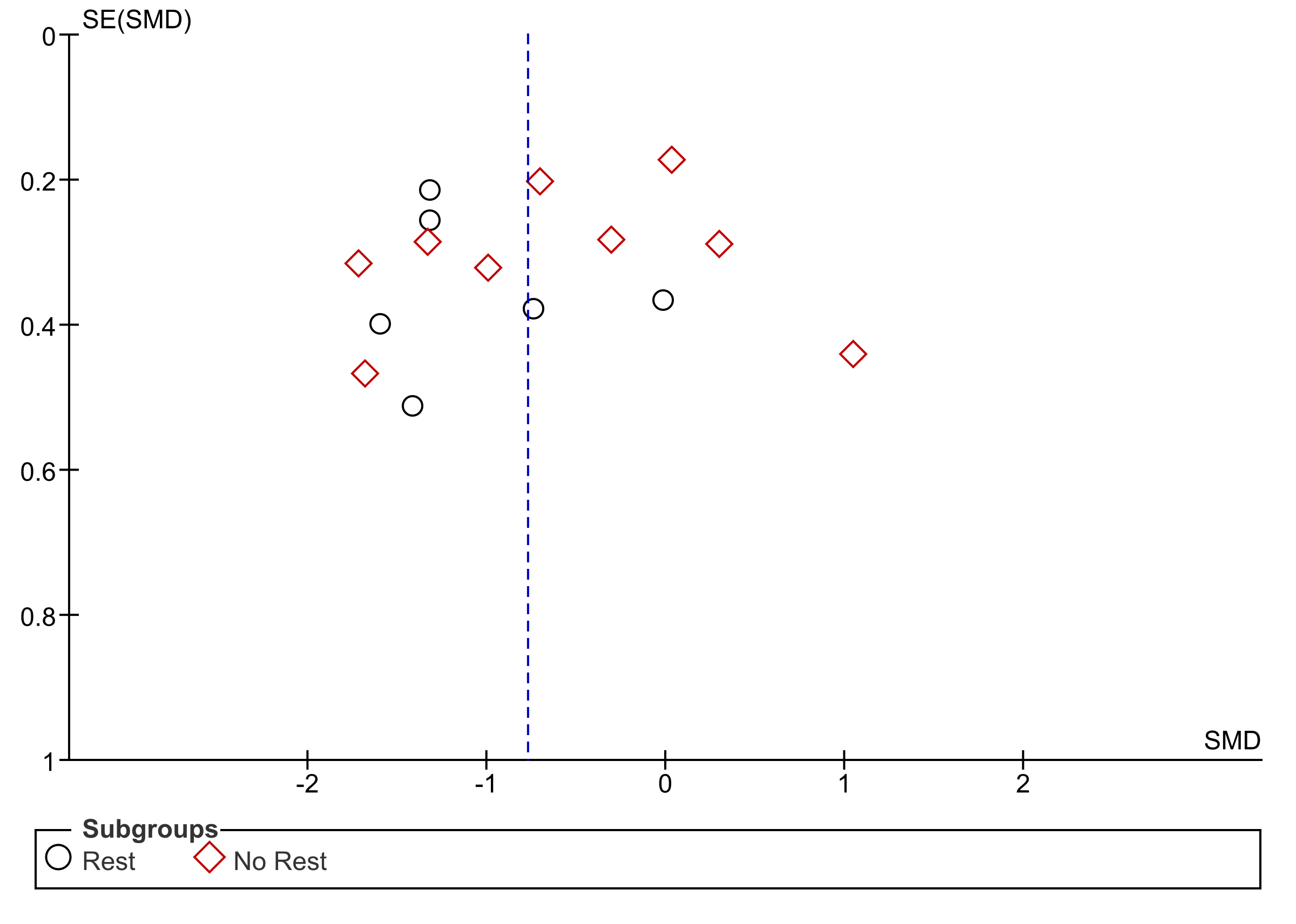


**Accuracy MA - Funnel plots for Owen (1988) (weight)**

1. Owen (1988) (weight)


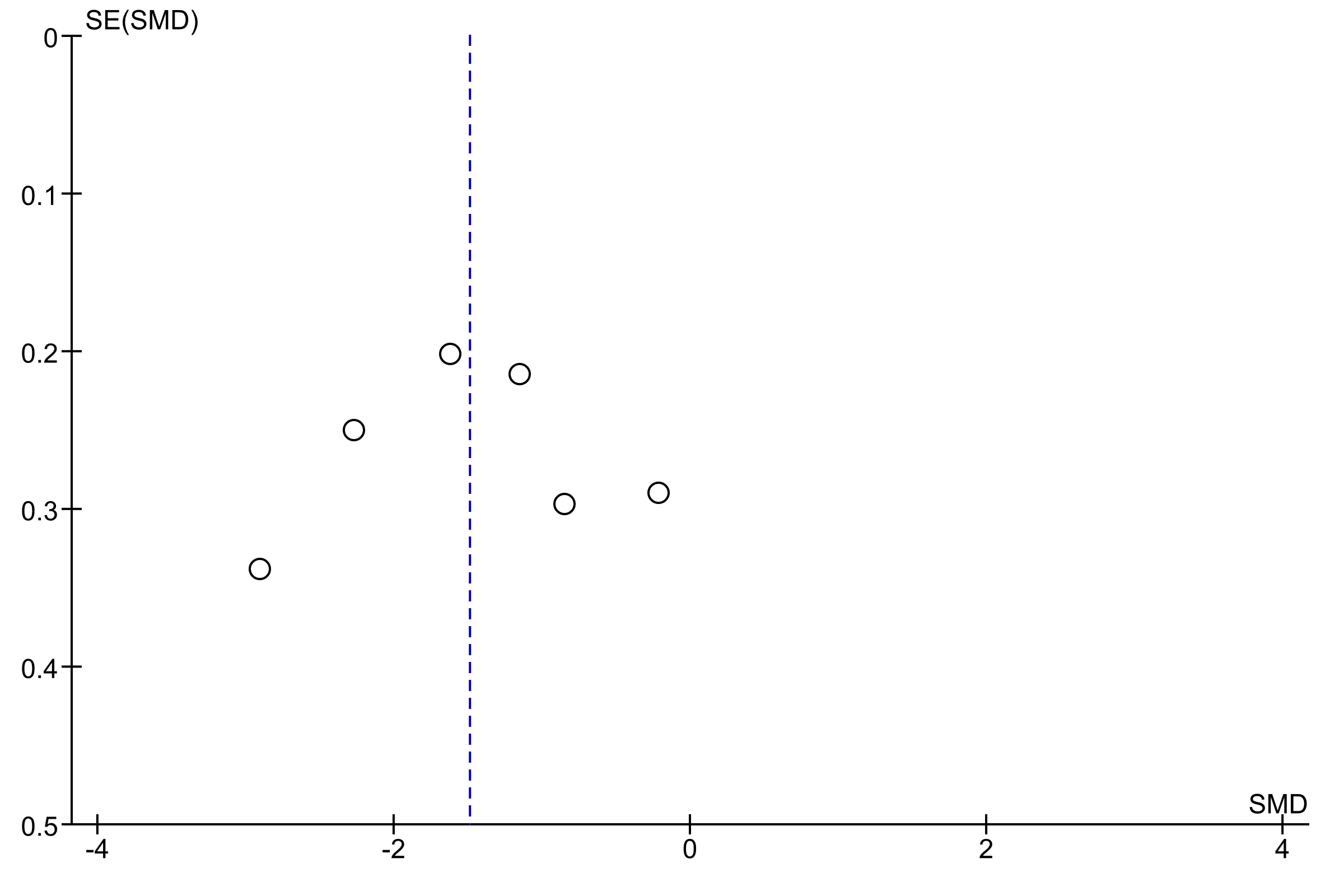


1. Owen (1988) (weight) – Sex


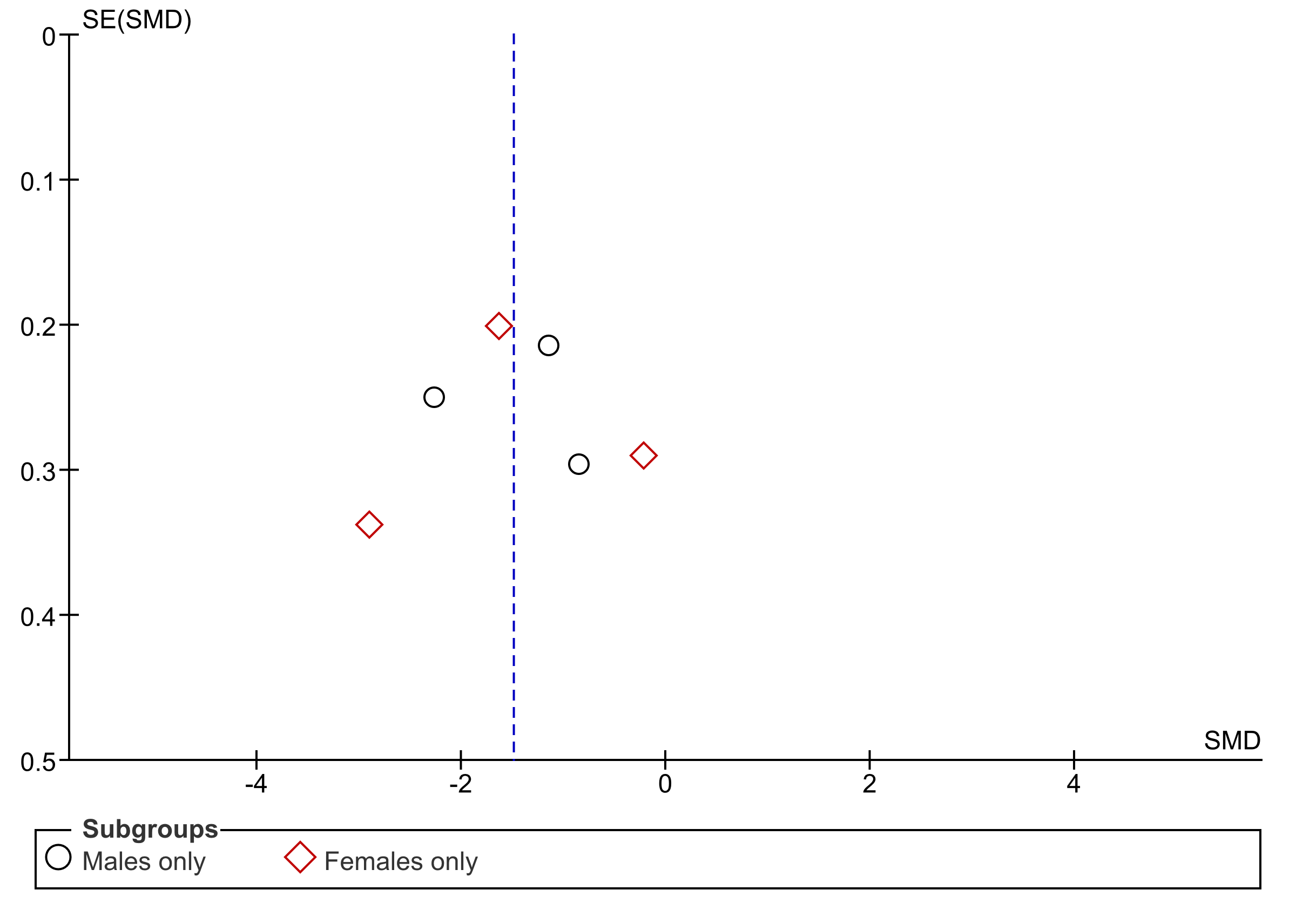


**Accuracy MA - Funnel plots for De Lorenzo (1999) (age, weight, height)**

1. De Lorenzo (1999) (age, weight, height)


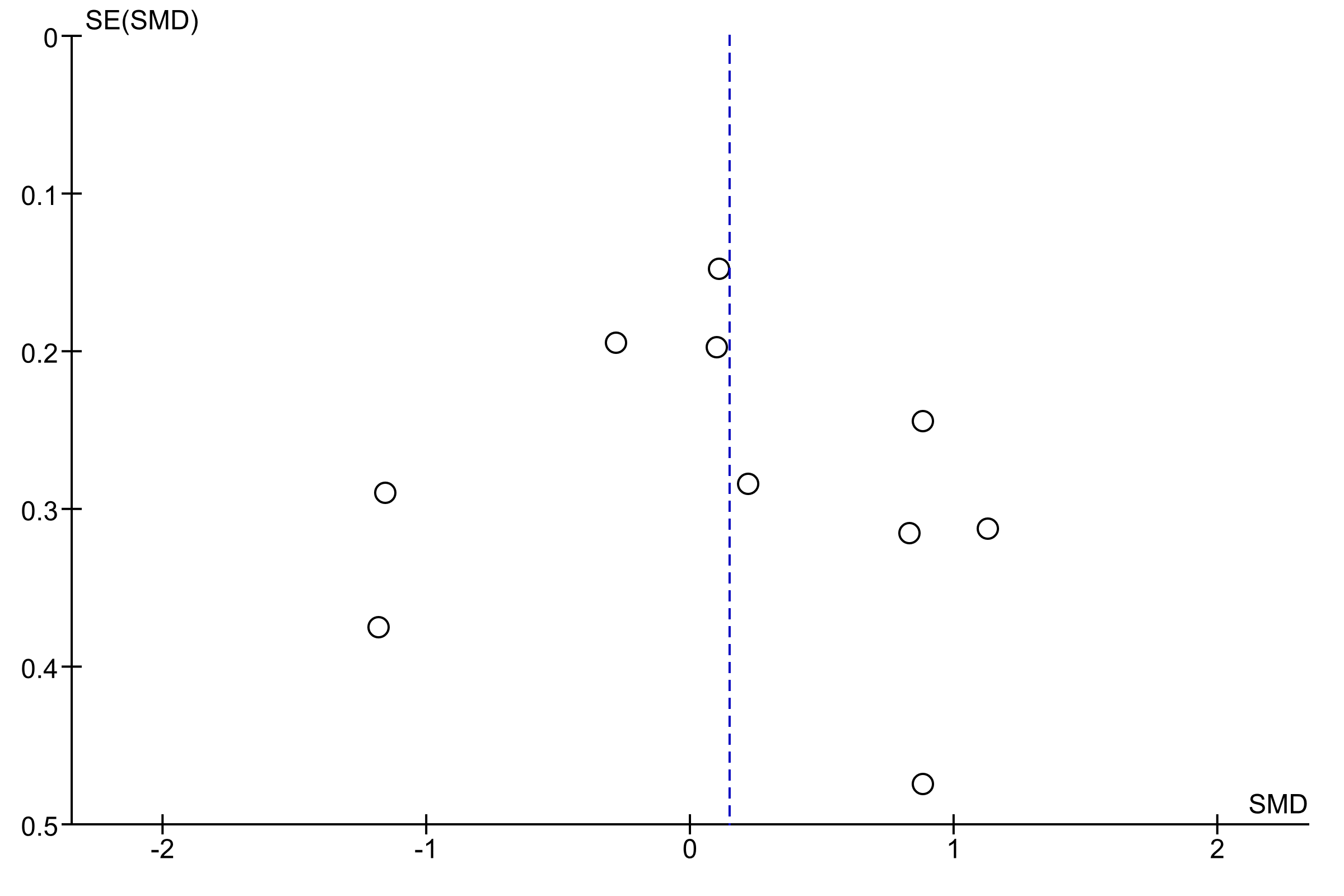


1. De Lorenzo (1999) (age, weight, height) – Sex


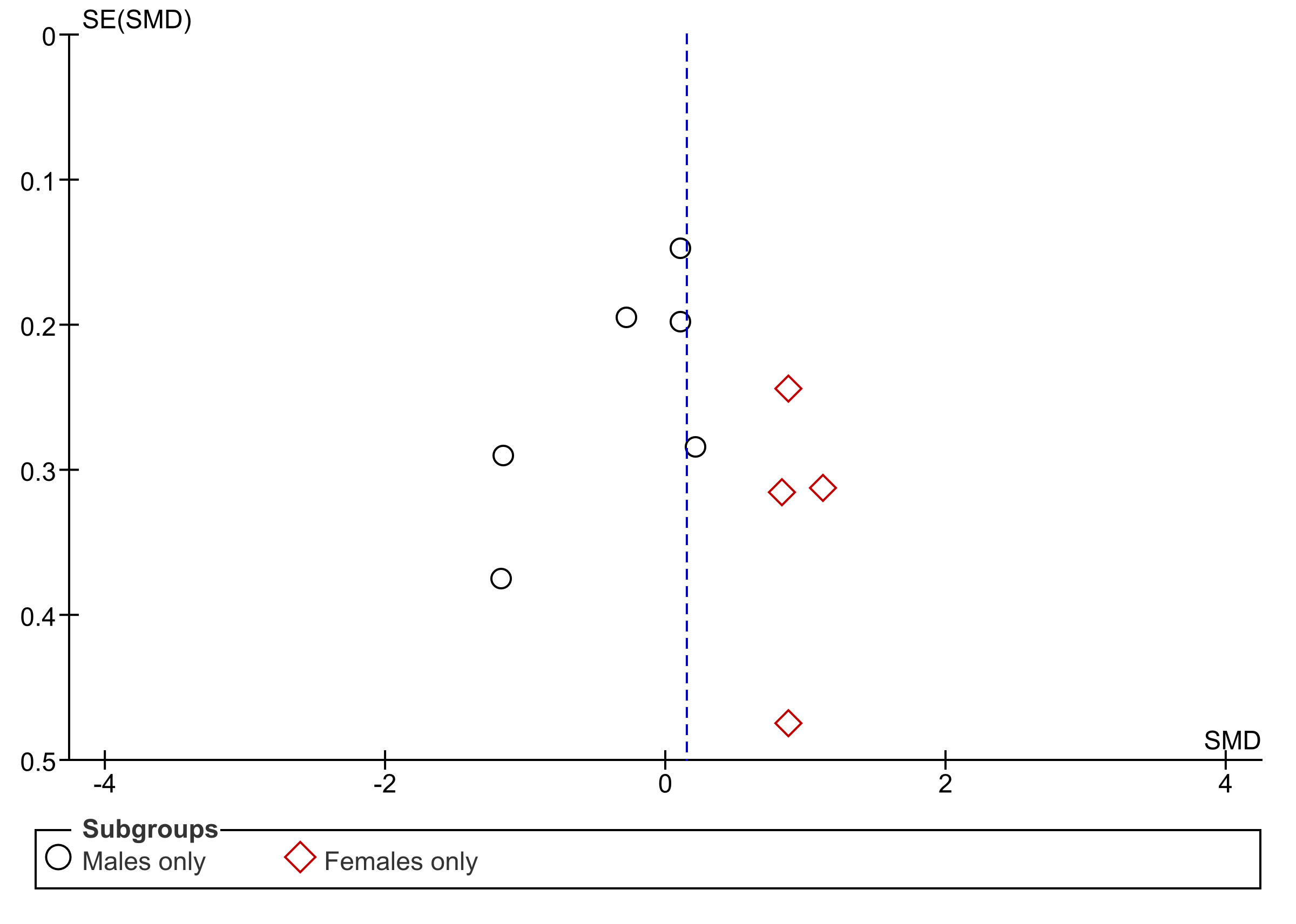


1. De Lorenzo (1999) (age, weight, height) – 24hr Physical Activity Abstinence


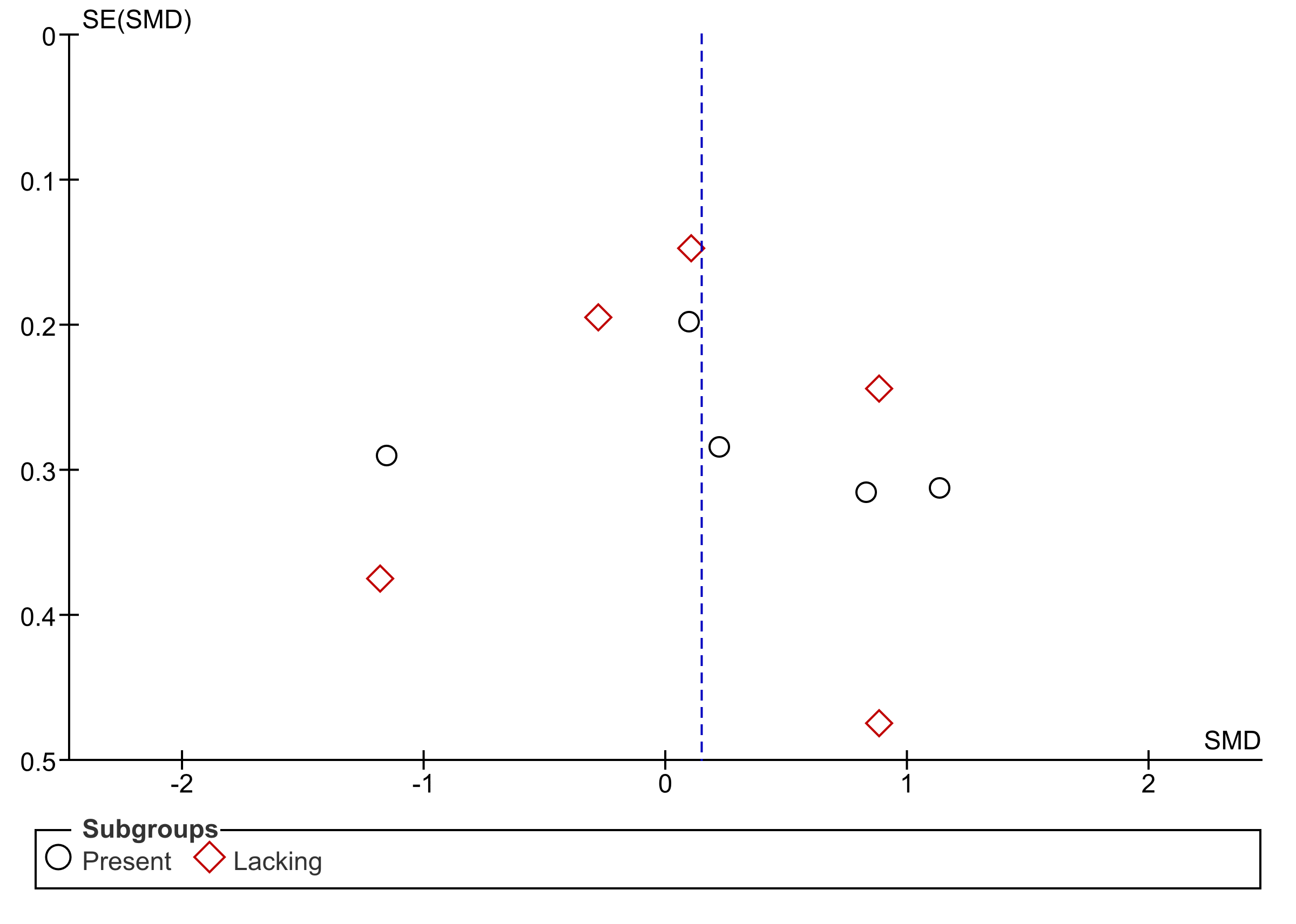


1. De Lorenzo (1999) (age, weight, height) – Pre-test Rest vs No Rest


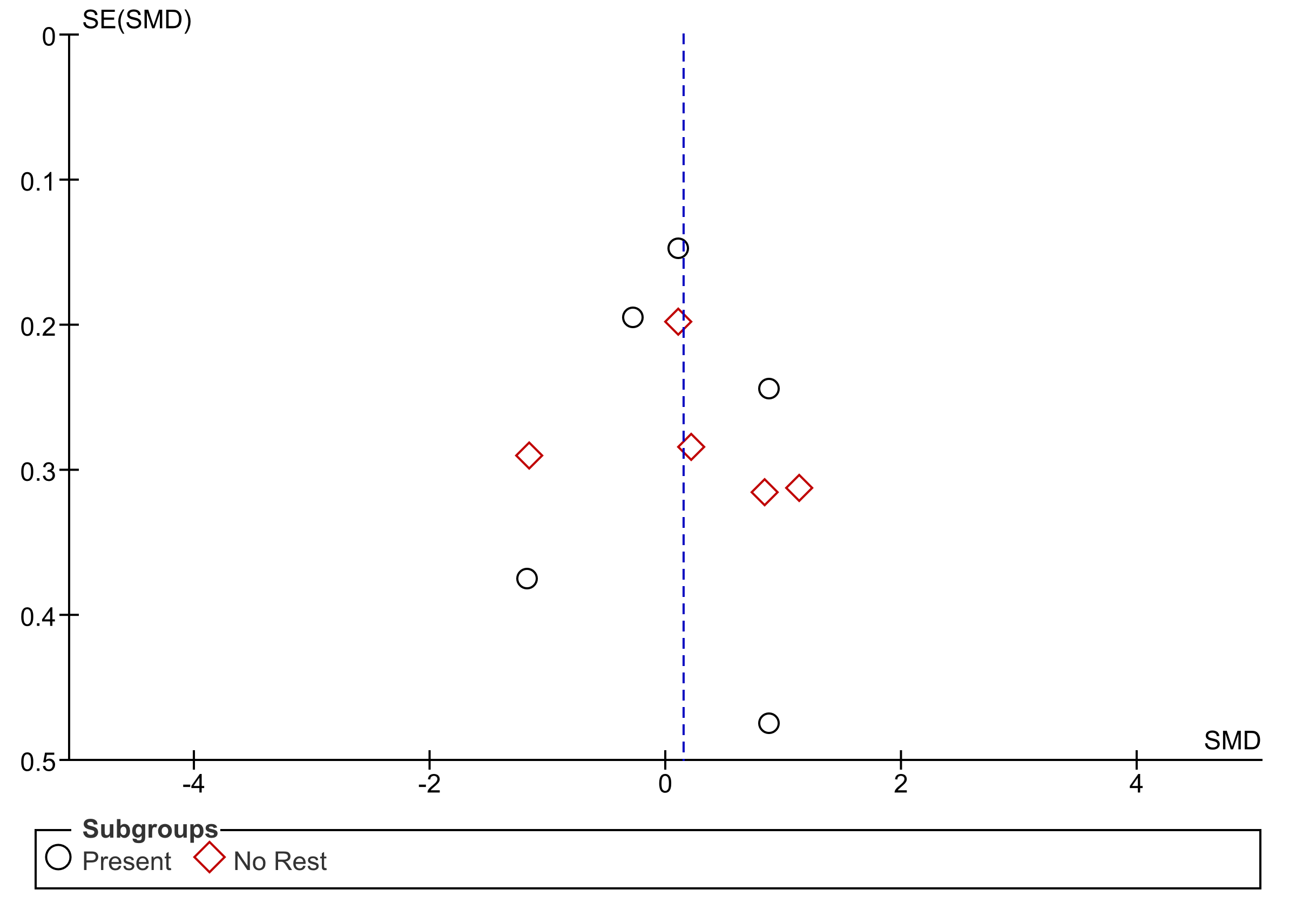


**Accuracy MA - Funnel plots for Koehler DXA (2016)**

1. Koehler DXA (2016)


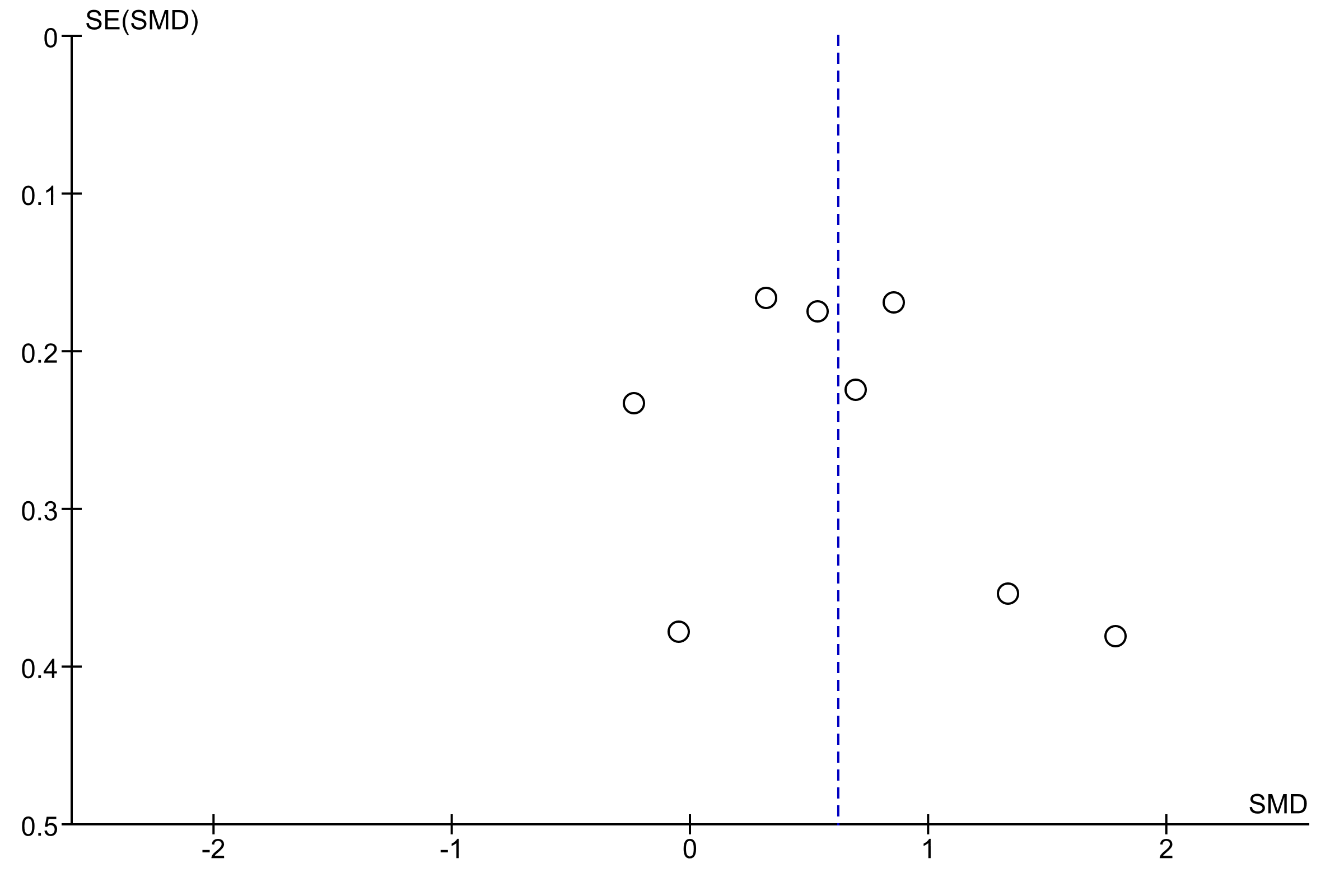


**Accuracy MA - Funnel plots for WHO.FAO.UNU (1985) (age, weight, height)**

1. WHO.FAO.UNU (1985) (age, weight, height)


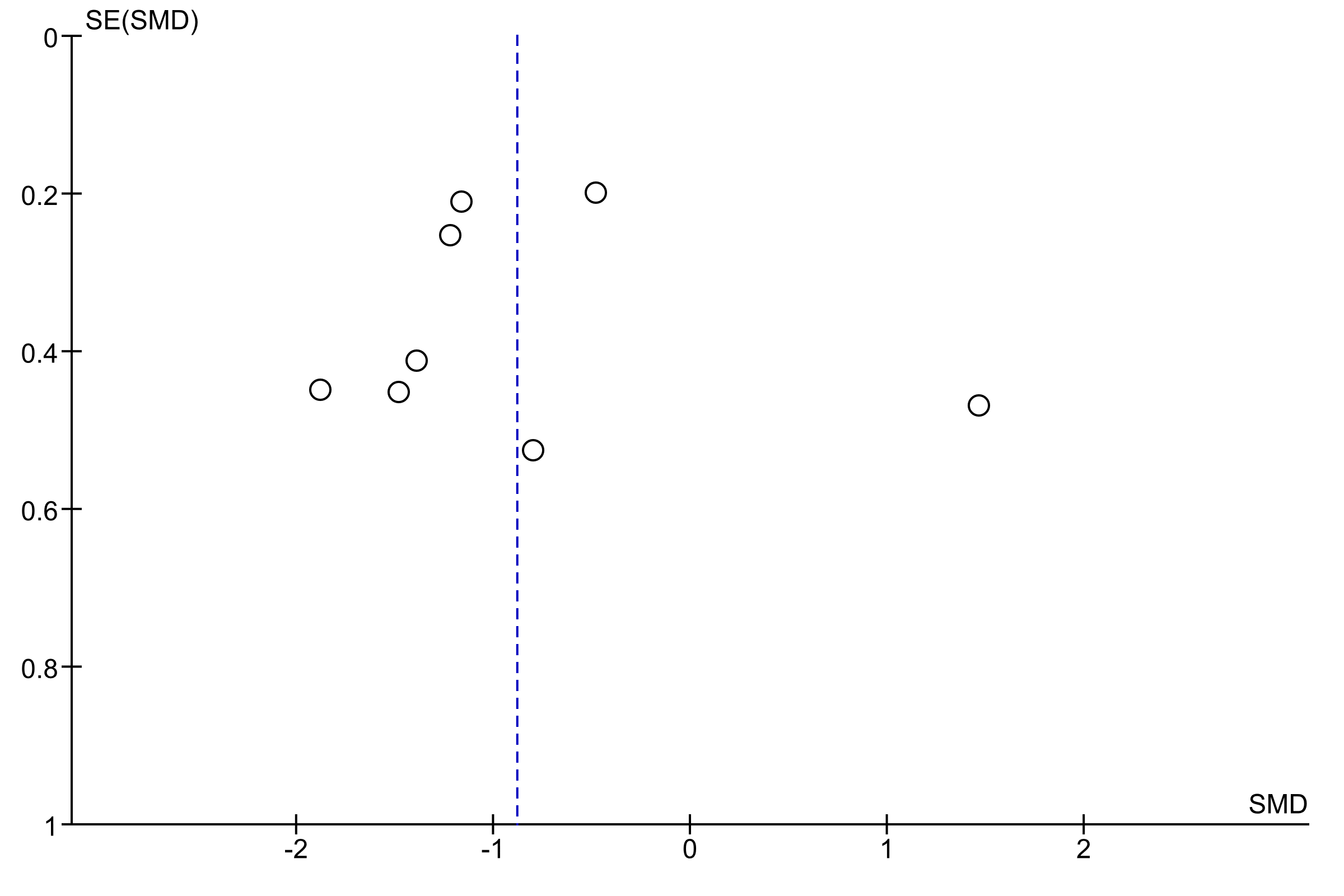


1. WHO.FAO.UNU (1985) (age, weight, height) – Sex


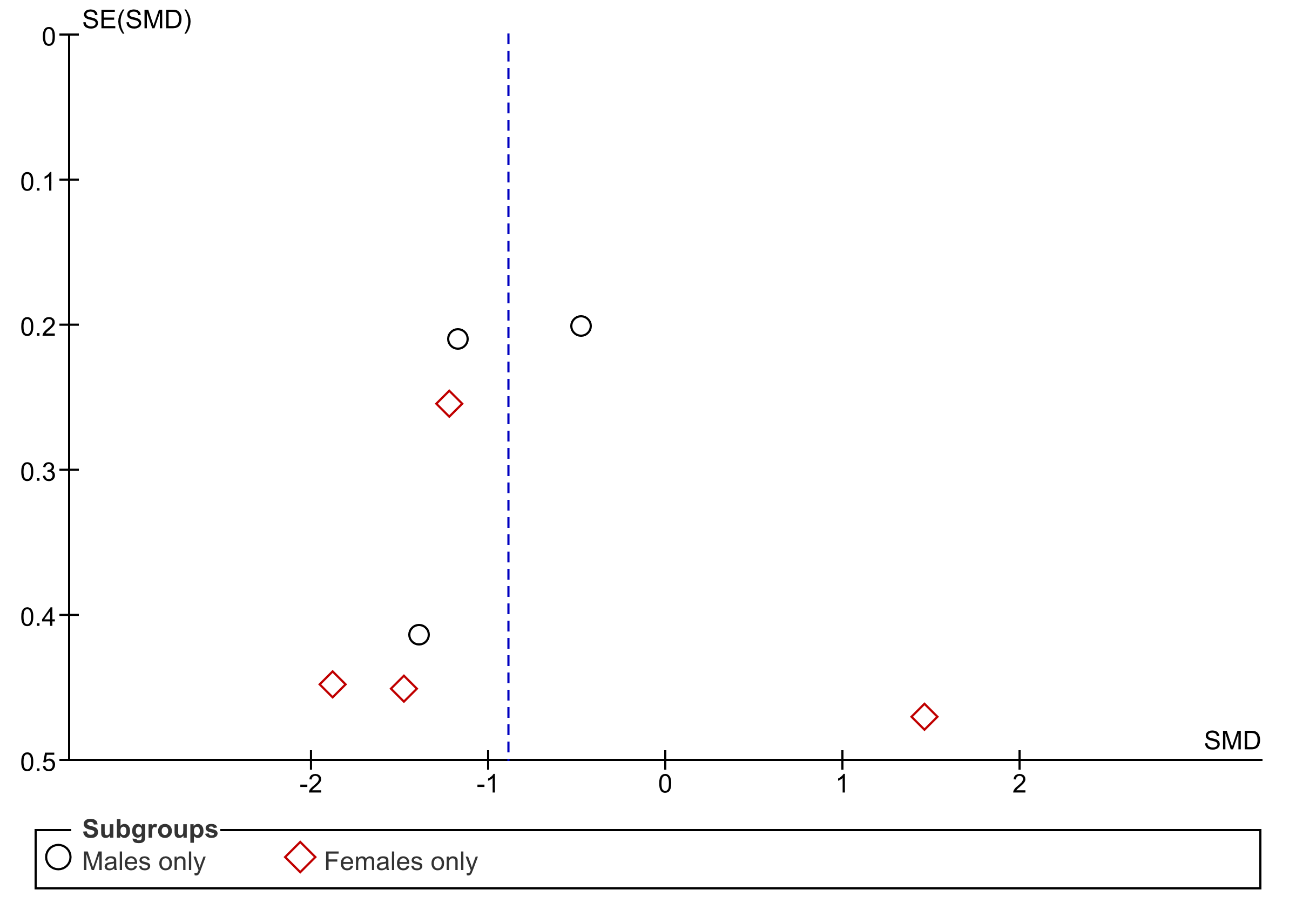


**Accuracy MA - Funnel plots for WHO.FAO.UNU (1985) (age, weight)**

1. WHO.FAO.UNU (1985) (age, weight)


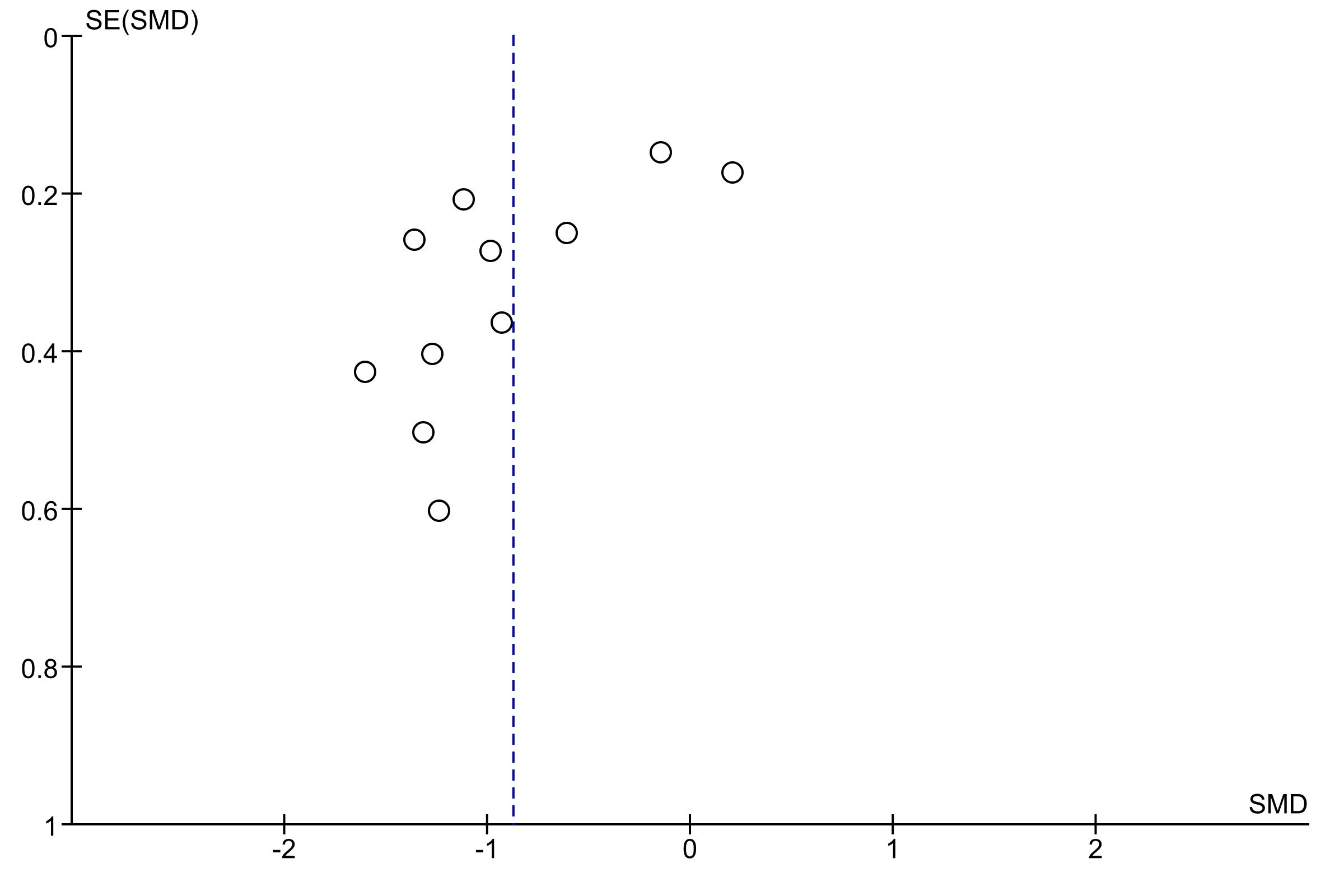


1. WHO.FAO.UNU (1985) (age, weight) – Sex


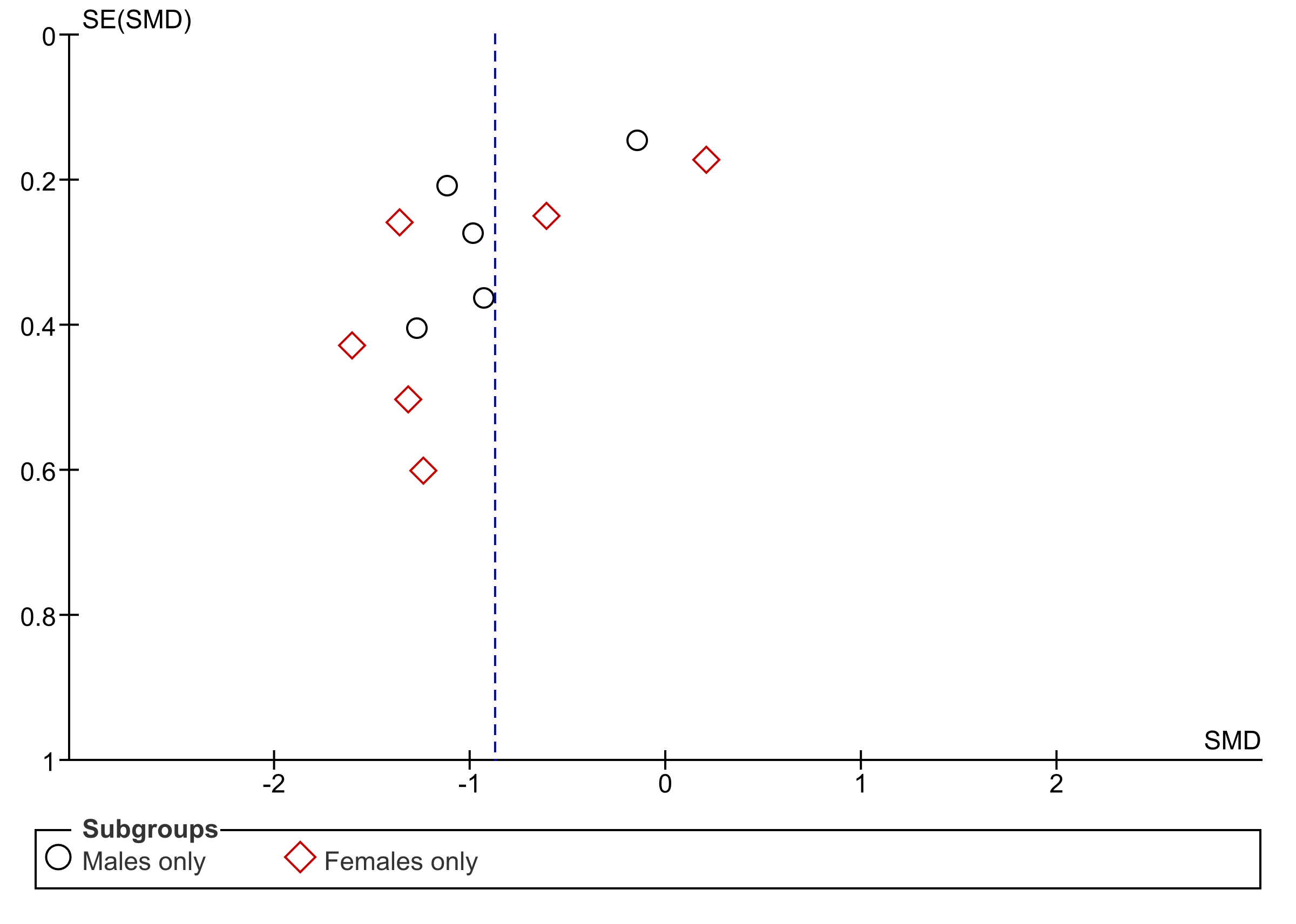


1. WHO.FAO.UNU (1985) (age, weight) – Discard, SS, & Validated Extraction Method


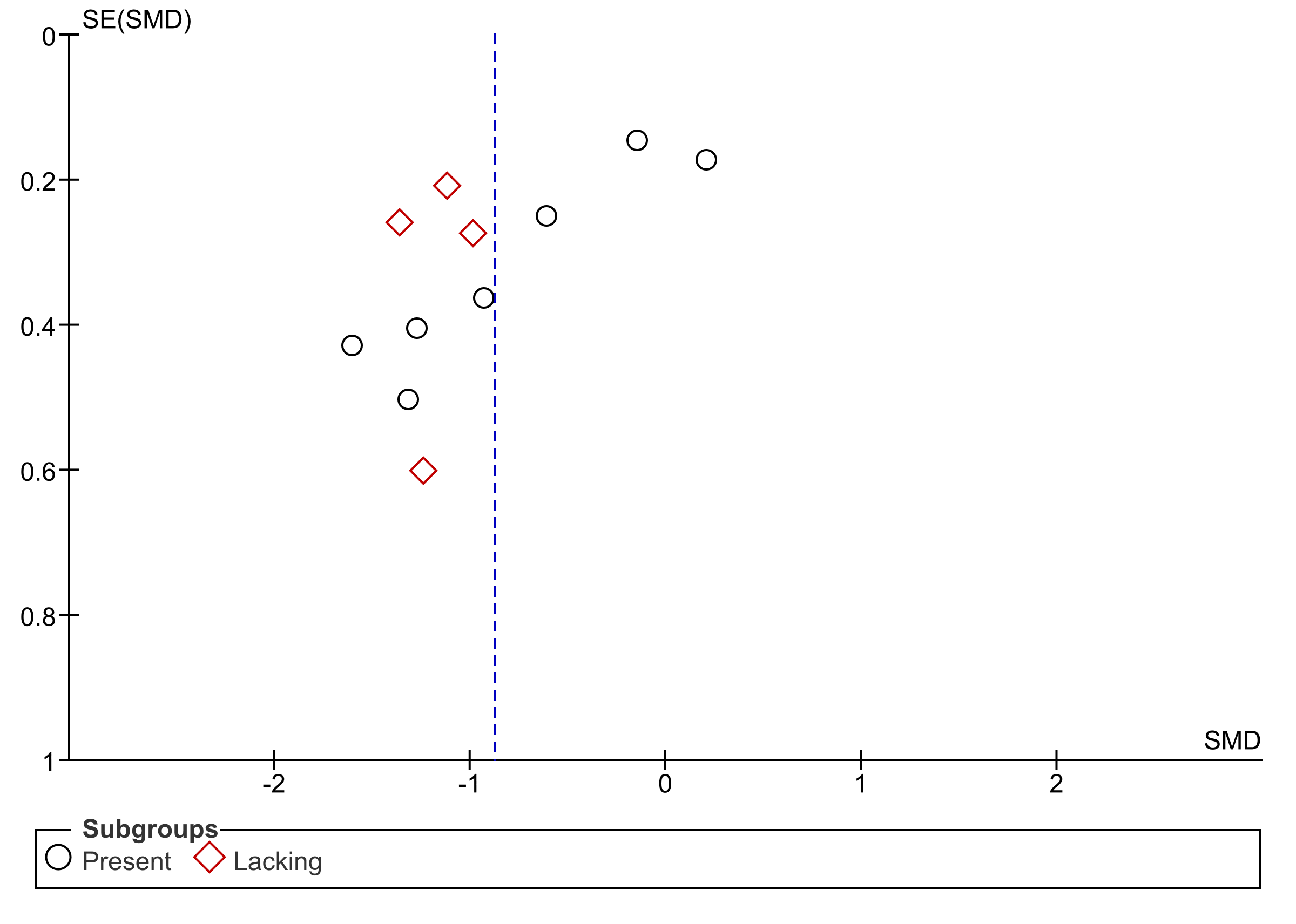


1. WHO.FAO.UNU (1985) (age, weight) – Pre-test Rest vs No Rest


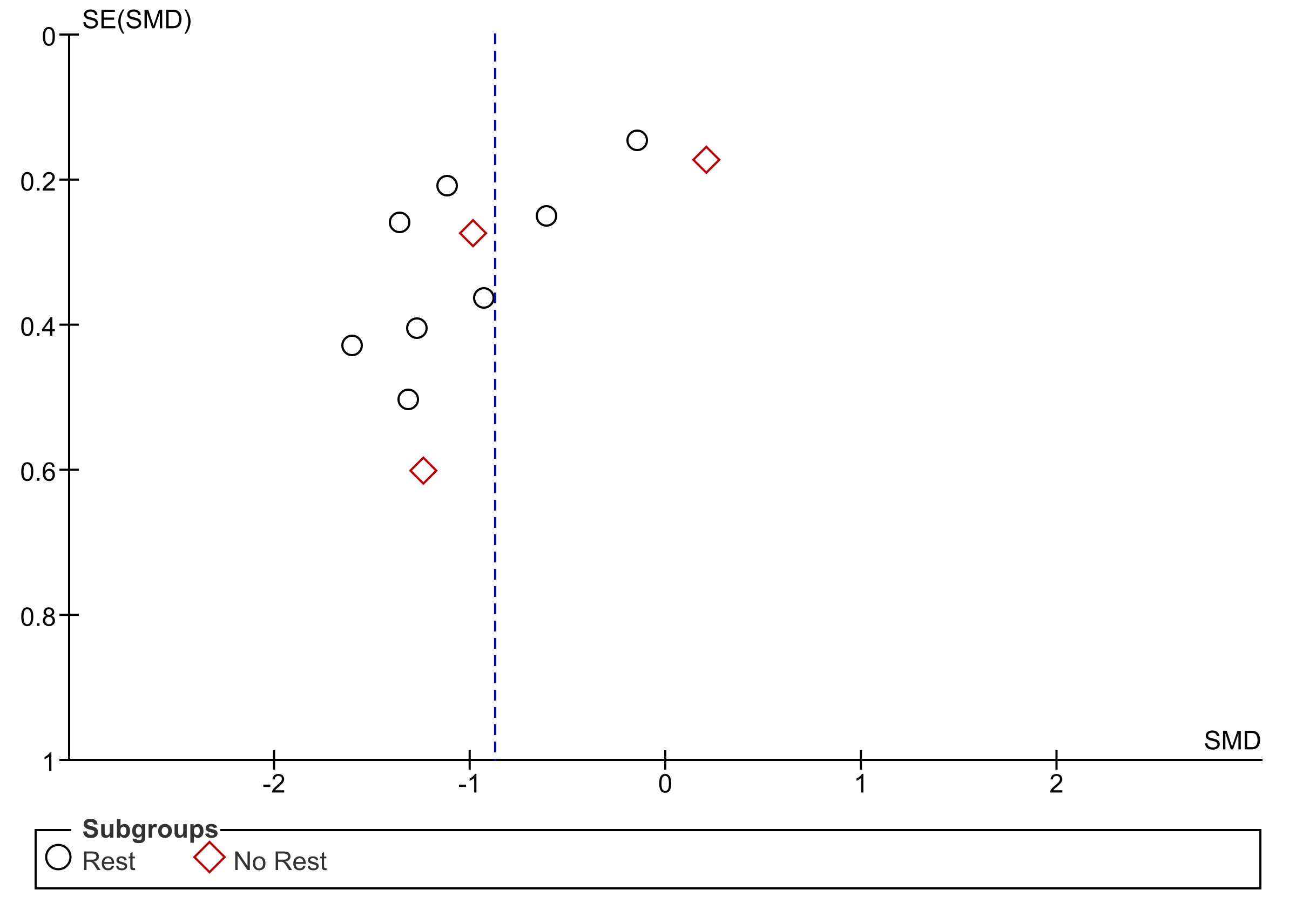


**Accuracy MA - Funnel plots for Ten-Haaf (2014) (age, weight, height)**

1. Ten-Haaf (2014) (age, weight, height)


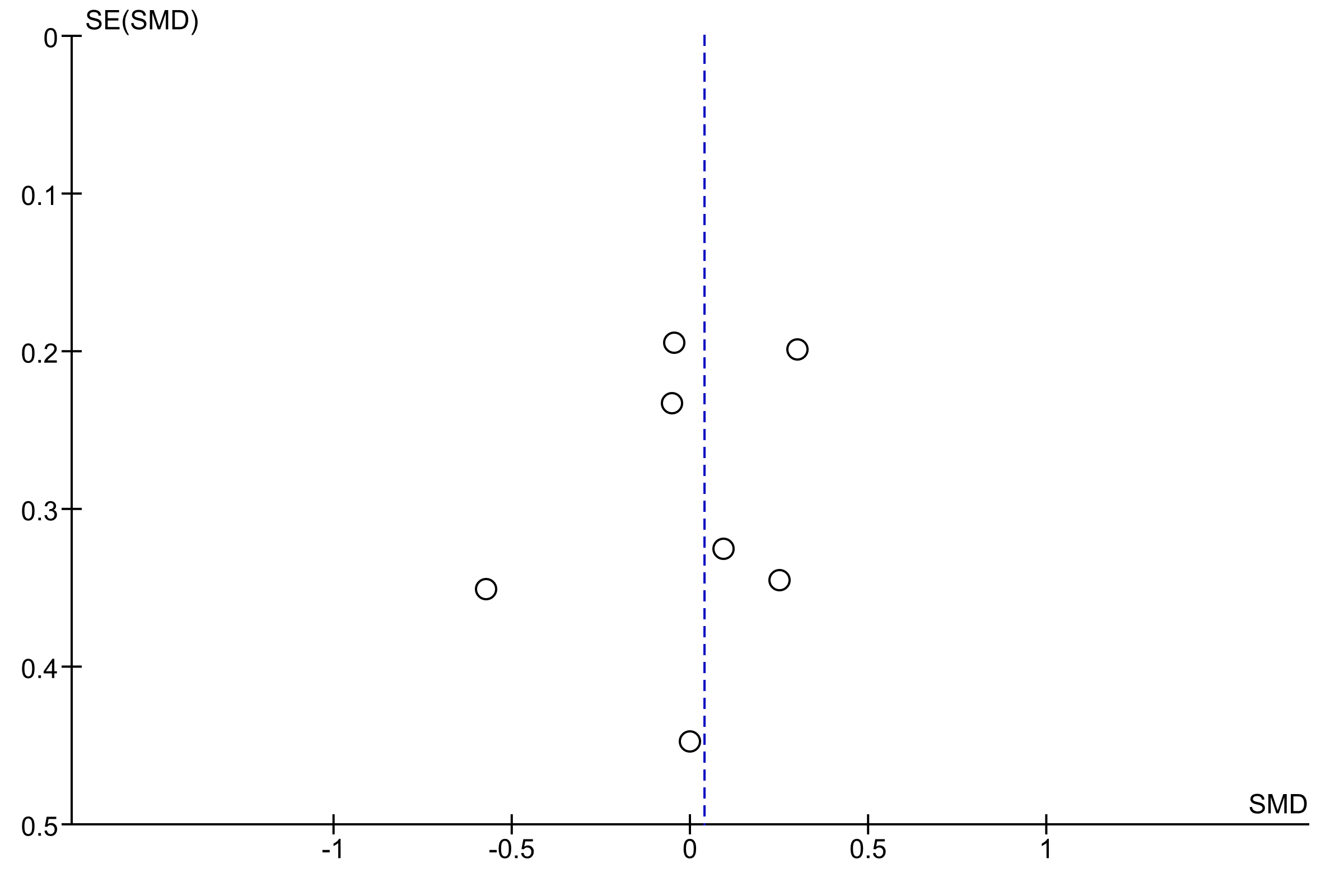


1. Ten-Haaf (2014) (age, weight, height) – Sex


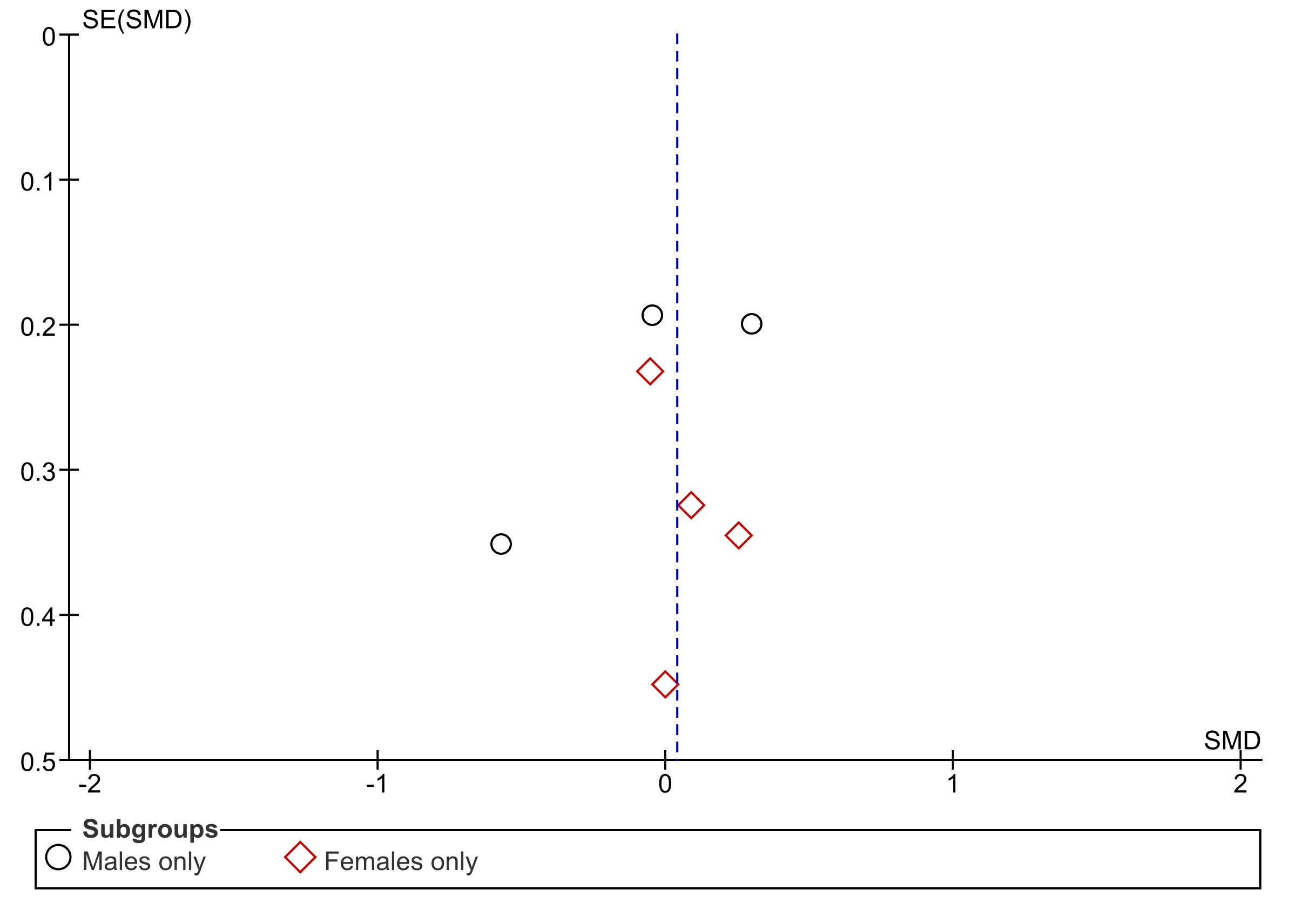


**Accuracy MA - Funnel plots for Nelson (1992) (FFM, FM)**

1. Nelson (1992) (FFM, FM)


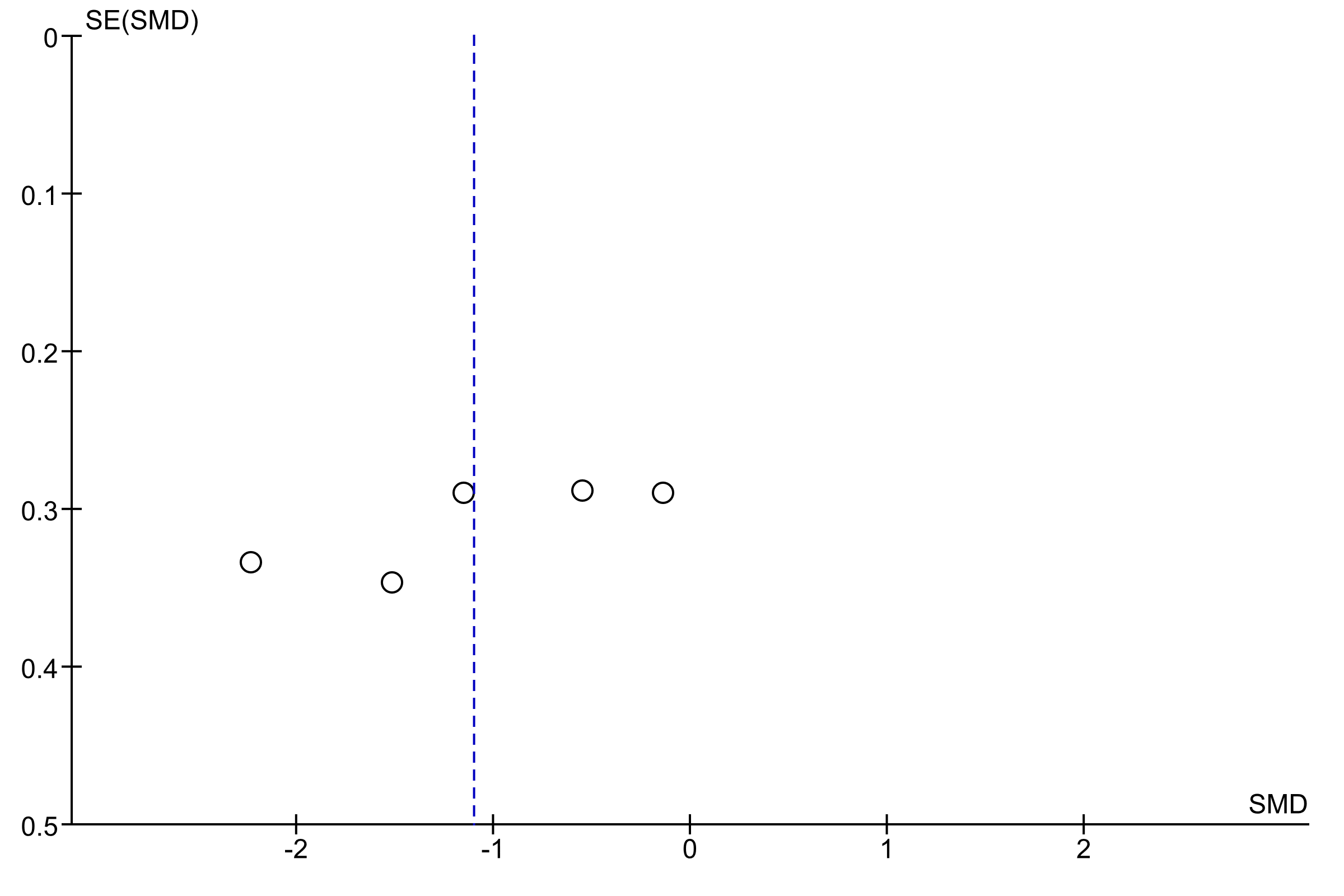


**Title of supplementary material:** Supplementary Document 4 – Fullmer Best Practise Guidelines [45]

| **Best practise question**  (all best practise questions are in relation to healthy and non-critically ill adults) | **Best practise guideline/recommendation as per Fullmer et al.** [45] | **Conclusion Grade**  (one to five depending on factors such as quality, consistency, sample size, clinical impact, and generalizability of studies; where I: good, II: fair, III: limited, IV: expert opinion only, V: not assignable) [77] | **Recommendation rating**  **(**Recommendations are rated as strong, fair, weak, consensus, or insufficient, and are considered “conditional” (the statement clearly defines a specific situation) or “imperative” (the statement is broadly applicable to a target population with restraints on their pertinence)) [77] |
| --- | --- | --- | --- |
| How long a rest period is needed before the measurement of RMR? | Practitioner should aim for a 30-min rest period before starting a measurement of RMR. If this is not possible, a 20-min rest period may be sufficient. | Grade I | Strong; Imperative |
| What kinds of activities can be done during the rest period? | Practitioner should ensure participants rest quietly and not engage in any activity during the 30-min rest period | Grade V | Consensus; Imperative |
| How long should the duration of the RMR measurement be to achieve a steady state? | Practitioners should discard the data for the first 5 min, and then use a validated steady state definition to determine the duration of the remainder of the measurement | Grade III | Weak; Conditional |
| If steady state cannot be achieved, how long should the duration of an indirect calorimetry measurement be? | None | Grade V | None |
| Is there a difference in RMR measurements related to the effects of different body positions? | Practitioner should conduct RMR measurements in the supine position when possible. | Grade II | Fair; Imperative |
| Is there a difference in RMR measurements related to different types of gas-collection devices (such as face mask, mouthpieces/nose clips, or ventilated hood/canopy)? | Practitioner may select any gas collection device (ventilated hood/canopy, mouthpiece and nose clip, or face mask) for an RMR measurement | Grade III | Weak; Imperative |
| What is the effect of diurnal (time of day) variation on RMR? | Practitioner may conduct a measurement of RMR at any time of day in a healthy adult, if resting conditions can be achieved | Grade III | Weak; Imperative |
| What are the room conditions (in terms of temperature) required for RMR measurement? | Practitioner should minimize the effect of ambient temperature on RMR in a healthy adult, by keeping the room temperature between 22C to 25C (72F to 77F) or providing a blanket during the measurement | Grade II | Fair; Imperative |
| What are the room conditions (in terms of humidity, lighting, and noise) required for RMR measurement? | Practitioner should measure RMR in a quiet room. | Grade V | Consensus; Imperative |
| How long should participants fast before an RMR measurement to avoid the thermic effect of food (TEF)? | Practitioner should ensure the participant has fasted at least 7 h to minimize the TEF. If a 7-h fast is not clinically feasible before measurement of RMR in the participant, the practitioner should instruct the individual that a small meal (<300 kcal) may be consumed 4 h before the measurement | Grade II | Fair; Imperative |
| How long should participants refrain from consuming caffeine or other stimulants before an RMR measurement? | Practitioner should ensure that a healthy adult refrains from ingesting caffeine or other stimulants for at least 4 h before an RMR measurement | Grade III | Fair; Imperative |
| How long should participants refrain from smoking and nicotine intake before an RMR measurement? | If participant uses nicotine products, the practitioner should ask the individual to abstain from such products for longer than 140 min before an RMR measurement | Grade III | Weak; Conditional |
| How long should participants refrain from resistance exercise before an RMR measurement? | None | Grade III | None |
| How long should participants refrain from very light intensity physical activity (e.g., getting dressed, driving, walking <5 min, etc) before an RMR measurement? | If a participant engages in very light intensity physical activity before an RMR measurement, the practitioner should ensure a 30-min rest period before the RMR measurement | Grade III | Weak; Conditional |
| How long should participants refrain from light to vigorous intensity physical activity before an RMR measurement? | If a participant engages in light to vigorous intensity physical activity, the practitioner should instruct the individual to refrain from physical activity before the RMR measurement for a period of time (e.g., 12-48 h for moderate to vigorous physical activity) | Grade V | Consensus; Conditional |
| Can respiratory quotient (RQ) be used to detect error in a measurement of RMR? | If the RQ falls outside the physiologic range (<0.67 or >1.3), the practitioner should suspect an error and repeat the RMR measurement | Grade II | Consensus; Conditional |
|  | If the RQ falls between 0.67 and 0.90, the practitioner should accept the measurement because RQ values within this range cannot reliably be used to detect feeding protocol violations | None | Consensus; Conditional |
|  | If the RQ is between 0.91 and 1.3 in a participant who has fasted, the practitioner should suspect a problem and consider repeating the measurement | None | Consensus; Conditional |

**Probing questions for checklist**

- Refrained from vigorous physical activity for at least 24hours prior to measurement?
- Refrained from eating for at least 7hours prior to measurement?
- Avoided caffeine and other stimulants for at least 4hours prior to measurement?
- Avoided nicotine for at least 2.5 hours prior to measurement?
- Rested quietly engaging in no activities for at least 30min prior to measurement?
- If participant not rested for 30 minutes prior to measurement, did they rest quietly engaging in no activities for at least 20 minutes prior to measurement?
- Tested in supine position?
- Room temperature between 22C and 25C and/or blanket provided?
- Quiet room with lights dimmed?
- *Note* Measurement can be completed at any time of day
- Discarded at least first 5 minutes of measure?
- Steady state achieved using a validated SS method (must be VO2 & VCO2 <10% CV)?

**Title of supplementary material:** Supplementary Document 5 – Individual study characteristics of all included studies

| **Study (author, year)** | **Participant characteristics (n, sex, age, height, weight, body composition)** | **Sports (by sex)** | **Athlete classification by McKay et al. 2022** [39]  **(with reasoning)** | **Study Design & Protocol** | **Name of prediction equations included in study (Name of Eq., year of publication, equation components)** | **Criterion comparison (IC) (Measurement device and test duration)** | **Body composition measurement method** | **Were participants sub-grouped when assessing equation performance? If so, how? And sub-group physical characteristics** |
| --- | --- | --- | --- | --- | --- | --- | --- | --- |
| Balci et al.  (2021) | N=49 (24 F)  Age (y): 19.69 ± 1.9  Weight (kg): 68.15 ± 14.49  Height (m): 1.71 ± 0.10  BMI (kg/m^2^): 22.8 ± 3.31  FFM (kg): 57.05 ± 11.93  BF (%): 15.16 ± 6.27 | Track and field (4 M, 4 F)  Long-distance swimming (4 M, 2 F)  Modern pentathlon (1 M, 4 F)  Fencing (1 M, 2 F)  Karate (5 M, 5 F)  Taekwondo (5 M)  Boxing (3 M, 3 F)  Soccer (2 M, 4 F) | Tier 3: Highly Trained/National Level  ("49 Turkish Olympic young adult national team athletes") | Cross-sectional  Body composition and RMR measurements  Single day of testing | Bernstein (1983) (FFM, FM, age)  Cunningham (1991) (FFM)  De Lorenzo (1999) (weight, height)  Harris-Benedict (1918) (age, weight, height)  Johnstone (2006) (FFM, FM, age)  Liu’s (1995) (age, weight, height)  Mifflin St.Jeor (1990) (age, weight, height)  Mifflin St.Jeor (1990) (FFM),  Nelson (1992) (FFM, FM),  Owen (2013) (weight)  Roza (1984) (age, weight, height) | Fitmate GS (Cosmed, Rome, Italy)  Test duration: 30 minutes  Acclimation/exclusion period: 5 minutes | BIA  (Tanita MC-980, 1,000 kHz, 0.1 accuracy, Japan) | Yes  **Males (N=25)**  Age (y): 19.1 ± 1.5  Weight (kg): 75.4 ± 12.4  Height (m): 1.79 ± 0.06  BMI (kg/m^2^): 22.9 ± 2.3  FFM (kg): 66.7 ± 7.6  BF (%): 10.6 ± 3.9  **Females (N=24)**  Age (y): 20.3 ± 2.1  Weight (kg): 60.6 ± 12.7  Height (m): 1.63 ± 0.07  BMI (kg/m^2^): 22.7 ± 4.1  FFM (kg): 47.0 ± 5.7  BF (%): 15.16 ± 6.27 |
| Carlsohn et al.  (2011) | N=17 (9 F)  Age (y): 23.16 ± 4.07  Weight (kg): 80.41 ± 15.81  Height (m): 1.83 ± 0.11  BMI (kg/m^2^): 24.0 ± 2.0  FFM (kg): 72.0 ± 14.0  BF (%): 15.77 ± 4.33 | Rowing (3 M, 5 F)  Canoe racing (5 M, 4 F) | Tier 3: Highly Trained/National Level  (“athletes of the German national teams were enrolled into this study") | Cross-sectional  Medical check, fasting blood samples, and body composition and RMR measurements  Two days of testing | Cunningham (1980) (LBM)  Harris-Benedict (1918) (age, weight, height) | Zan 600 CPET (nSpire Health Group, United Kingdom)  Test duration: 30.9 ± 4 minutes  Acclimation/exclusion period: 10 minutes | Skinfold thickness whilst fasted.  Same examiner.  Lange calliper (SKSH Ltd., Zurich, Switzerland)  10-site skin fold method of Parizkova and Buzkova [78] | Yes  **Males (N=8)**  Age (y): 23.0 ± 5.0  Weight (kg): 92.9 ± 10.0  Height (m): 1.93 ± 0.07  BMI (kg/m^2^): 25.0 ± 2.0  FFM (kg): 81.0 ± 8.0  BF (%): 12.7 ± 2.0  **Females (N=9)**  Age (y): 23.3 ± 3.0  Weight (kg): 69.3 ± 11.0  Height (m): 1.75 ± 0.07  BMI (kg/m^2^): 22.5 ± 2.0  FFM (kg): 56.1 ± 7.0  BF (%): 18.5 ± 4.0 |
| De Lorenzo et al.  (1999)*  *Not included in MA | N=51 (0 F)  Age (y): 22.3 ± 3.5  Weight (kg): 78.0 ± 11.5  Height (m): 1.78 ± 0.07  BMI (kg/m^2^): 24.4 ± 2.5  FFM (kg): 67.0 ± 6.9  BF (%): 12.4 ± 4.1 | Waterpolo (22 M)  Judo (12 M)  Karate (17 M) | Tier 2: Trained / Developmental  (Although subjects reported large training volumes (3 hours per day, 6 days per week), no description of training sessions or level of competition reported. Sample is heterogenous consisting of a variety of sports with unknown standards for highly trained training volumes) | Cross-sectional  Body composition and RMR measurements  Unknown number of testing days | Cunningham (1980) (LBM)  De Lorenzo – current population (1999) (weight, height)  Fleisch (1951) (BSA)  Harris-Benedict (1918) (age, weight, height)  Mifflin St.Jeor (1990) (age, weight, height)  Owen (1988) (FFM)  Robertson & Reid (1952) (BSA)  FAO/WHO/UNU (1985) (age, weight, height) | SensorMedics 2900, (Anaheim, CA)  Test duration: 30 minutes  Acclimation/exclusion period: 0 minutes | DXA  (model DPX, Lunar, software version 3.6, Madison, WI)  DXA not performed in n=8 subjects due to problems with instrument. | No  **Males only (N=51)** |
| Devrim-Lanpir et al.  (2019) | N=30 (15 F)  Age (y): 37.80 ± 6.75  Weight (kg): 64.74 ± 10.20  Height (m): 1.70 ± 0.10  BMI (kg/m^2^): ?? ± ??  FFM (kg): 54.34 ± 10.44  BF (%): 16.40 ± 4.80 | Triathletes (10 M, 6 F)  Ultra-marathoners (5 M, 9 F) | Tier 3: Highly Trained/National Level  (Endurance exercise weekly training volumes of >15 hours per week for at least 3 years. Participation in ultra-endurance events) | Cross-sectional  Body composition and RMR measurements  Unknown number of testing days | Cunningham (1980) (LBM)  Harris-Benedict (1918) (age, weight, height)  Mifflin-St.Jeor (1990) (age, weight, height)  Sabounchi et al (2013 - Structure 11 (FFM, FM)  Sabounchi et al (2013 - Structure 5 (FFM)  Sabounchi et al (2013) - Structure 4 (age, FFM, FM)  Wang (2000) (FFM)  FAO/WHO/UNU (1985) (age, weight, height)  FAO/WHO/UNU (1985)(age, weight) | Cosmed K5 metabolic cart, (Cosmed, ROME Italy)  Test duration: 20 minutes  Acclimation/exclusion period: 5 minutes | BIA  (MF-BIA, TANTICA MC-780, Japan) | Yes  **Males (N=15)**  Age (y): 38.46 ± 5.32  Weight (kg): 73.01 ± 7.38  Height (m): 1.78 ± 0.07  BMI (kg/m^2^): ?? ± ??  FFM (kg): 63.36 ± 6.39  BF (%): 13.16 ± 3.89  **Females (N=15)**  Age (y): 37.13 ± 7.87  Weight (kg): 56.46 ± 4.07  Height (m): 1.63 ± 0.04  BMI (kg/m^2^): ?? ± ??  FFM (kg): 45.31 ± 2.78  BF (%): 19.64 ± 3.14 |
| Gravante et al.  (2001)*  *Not included in MA | N=16 (16 F)  Age (y): 24.74 ± 5.80  Weight (kg): 58.08 ± 8.34  Height (m): 1.61 ± 0.06  BMI (kg/m^2^): 22.48 ± 2.69  FFM (kg): 42.03 ± 4.60  BF (%): 27.21 ± 5.05 | Soccer (16 F) | Tier 3: Highly Trained/National Level  “16 professional sportswomen” | Cross-sectional  Food records, blood samples, and body composition and RMR measurements  Unknown number of testing days | LARN (1996) | Vmax 29N (Sensormedics Italia, Milan, Italy)  Test duration: 30 minutes  Acclimation/exclusion period: 10-15 minutes | BIA  (model BIA-109, RJL Systems, Detroit, MI) | No  **Females only (N=16)** |
| Jagim et al.  (2018)*  *Not included in MA | N=116 (48 F)  Age (y): 19.81 ± 1.46  Weight (kg): 81.16 ± 21.1  Height (m): 1.75 ± 0.10  BMI (kg/m^2^): 25.83 ± 5.58  FFM (kg): 65.47 ± 16.09  BF (%): 18.45 ± 8.14 | American football (56 M)  Baseball (8 M)  Track (distance) (4 M, 18 F)  Track (sprinters) (6 F)  Track (throwers) (3 F)  Swimming (4 F)  Soccer (15 F)  Tennis (2 F) | Tier 3: Highly Trained/National Level  ("National Collegiate Athletic Association (NCAA) Division III") | Cross-sectional  Body composition and RMR measurements  Single day of testing | Jagim – current population (2018) (weight) | ParvoMedics True One Metabolic System (Sandy, Utah, USA)  Test duration: 20 minutes  Acclimation/exclusion period: 10 minutes | BODPOD (Cosmed, Rome, Italy) | Yes  **Males (N=68)**  Age (y): 20.1 ± 1.5  Weight (kg): 93.7 ± 16.3  Height (m): 1.82 ± 0.06  BMI (kg/m^2^): 27.9 ± 5.5  FFM (kg): 77.3 ± 8.1  BF (%): 16.3 ± 8.6  **Females (N=48)**  Age (y): 19.4 ± 1.3  Weight (kg): 63.4 ± 12.7  Height (m): 1.67 ± 0.06  BMI (kg/m^2^): 22.9 ± 4.2  FFM (kg): 48.7 ± 7.3  BF (%): 21.5 ± 6.3 |
| Jagim et al.  (2019) | N=50 (22 F)  Age (y): 20.02 ± 1.5  Weight (kg): 81.10 ± 20.4  Height (m): 1.75 ± 0.10  BMI (kg/m^2^): ?? ± ??  FFM (kg): 66.28 ± 16.12  BF (%): 17.95 ± 8.05 | American football (21 M)  Baseball (3 M)  Track & Field (4 M, 3 F)  Swimming/diving (4 F)  Soccer (15 F) | Tier 3: Highly Trained/National Level  ("National Collegiate Athletic Association (NCAA) Division III") | Cross-sectional  Body composition and RMR measurements  Single day of testing | Cunningham (1980) (LBM)  De Lorenzo (1999) (weight, height)  Harris-Benedict (1918) (age, weight, height)  Mifflin St.Jeor (1990) (age, weight, height)  Nelson (1992) (FFM, FM) | ParvoMedics True One Metabolic System (Sandy, Utah, USA)  Test duration: 20 minutes  Acclimation/exclusion period: 10 minutes | BODPOD (Cosmed, Rome, Italy) | Yes  **Males (N=28)**  Age (y): 20.29 ± 1.56  Weight (kg): 94.54 ± 16.24  Height (m): 1.82 ± 0.07  BMI (kg/m^2^): ?? ± ??  FFM (kg): 79.08 ± 7.71  BF (%): 15.07 ± 8.45  **Females (N=22)**  Age (y): 19.67 ± 1..43  Weight (kg): 63.18 ± 7.25  Height (m): 1.66 ± 0.05  BMI (kg/m^2^): ?? ± ??  FFM (kg): 49.22 ± 4.36  BF (%): 21.79 ± 5.55 |
| Joseph et al.  (2017) | N=30 (0 F)  Age (y): 21.50 ± 2.87  Weight (kg): 75.95 ± 11.36  Height (m): 1.69 ± 0.06  BMI (kg/m^2^): 26.09 ± 4.27  FFM (kg): 63.94 ± 9.49  BF (%): 15.15 ± 5.28 | Weightlifting (30 M) | Tier 4: Elite / International level  (“ 30 selected professional male weightlifters in the age group of 17–28 years who were actively competing at national/international level”.  Authors report training volumes to be maximal/near maximal for weightlifting "4–5 h daily, 5–6 days a week".  Additionally, framework informs that when participants are split between two adjacent tiers, the higher of the two tiers should be selected) | Cross-sectional  Body composition and RMR measurements  Unknown number of testing days | Cunningham (1980) (LBM)  FAO/WHO/UNU (1985) (age, weight)  Harris‑Benedict (1918) (age, weight, height)  ICMR - Indian Council of Medical Research (1990)  Joseph (2017) (weight)  Katch‑McArdle (2001) (LBM)  Mifflin‑St. Jeor (1990) (age, weight, height)  Nelson (1992) (FFM, FM)  Owen (1988) (age, weight, height) | Jaeger Oxycon Pro (Muggensturm, Germany)  Test duration: 40 minutes  Acclimation/exclusion period: 0 minutes | DXA and skinfolds  (DXA, Hologic Delphi W [S/N 70471 DXA scanner])  (Harpenden caliper, CMS instruments, London) | No  **Males only (N=30)** |
| Koehler et al.  (2016) | N=79 (79 F)  Age (y): 23.42 ± 5.48  Weight (kg): 58.31 ± 8.11  Height (m): 1.66 ± 0.09  BMI (kg/m^2^): 21.17 ± 2.33  FFM (kg): 42.16 ± 6.42  BF (%): 22.72 ±  7.71 | Recreational aerobic exercise (57 F)  Recreational resistance training (3 F)  Recreational concurrent exercise (19 F) | Tier 1: Recreationally Active  (Participants reported to only exceed WHO PA guidelines. Association with sport not reported.  "≥2 hr of purposeful moderate- to high-intensity exercise per week") | Cross-sectional  Medical check, blood and urine samples, and body composition and RMR measurements  Number of testing days varied | Koehler DXA-predicted – current population (2016) (brain mass, skeletal muscle mass, adipose tissue mass, bone mass, residual mass) | CareFusion (Yorba Linda, CA, United States)  Test duration: approx. 30 minutes  Acclimation/exclusion period: unknown | DXA  (enCORE 2002 software, version 6.50.069) | Yes  **Amenorrhoeic group (N=42)** **”exercising women with amenorrhea”*  Age (y): 22.3 ± 4.44  Weight (kg): 58.4 ± 8.89  Height (m): 1.68 ± 0.09  BMI (kg/m^2^): 20.8 ± 1.94  FFM (kg): 43.1 ± 5.18  BF (%): 21.5 ±  5.83  **Ovulatory group (N=37)**  **”exercising women with eumenorrheic, ovulatory menstrual cycles*  Age (y): 24.7 ± 6.22  Weight (kg): 58.2 ± 7.11  Height (m): 1.64 ± 0.09  BMI (kg/m^2^): 21.6 ± 1.22  FFM (kg): 41.1 ± 3.65  BF (%): 24.1 ± 4.87 |
| Langan-Evans et al.  (2020)*  *Not included in MA | N=1 (0 F)  Age (y): 19  Weight (kg): 72.5  Height (m): 1.66  BMI (kg/m^2^): 26.31  FFM (kg): 54.5  BF (%): 16.7 | Taekwondo (1 M) | Tier 4: Elite / International level  ("was a male international standard (>5 yr experience) Taekwondo competitor, who typically competed 10 times per year") | Longitudinal case study (9 weeks)  Repeat measures of blood and urine samples, body composition, RMR, VO2 max, and food records. | Cunningham (1980) (LBM) | (GEM Open Circuit Indirect Calorimeter; GEMNutrition Ltd., Warrington, UK)  Test duration: unknown  Acclimation/exclusion period: unknown | DXA and skinfolds  (DXA- QDR Series Discovery A; Hologic Inc., Bedford, MA, USA - software  version 12:4:3)  (Harpenden, Baty Int., West Sussex, Great Britain) | No  **Single male case study (N=1)** |
| Mackay et al.  (2019) | N=25 (25 F)  Age (y): 30.10 ± 10.36  Weight (kg): 64.79 ± 9.06  Height (m): 1.69 ± 0.08  BMI (kg/m^2^): 22.96 ± 2.72  FFM (kg): ?? ± ??  BF (%): ?? ± ?? | N/a  Authors did not report any descriptions of sports played, participated, and/or competed in. Only weekly training volumes reported.  **Sub-elite group (hr/wk)**  16.0 ± 4.0  **Recreational group (hr/wk)**  8.0 ± 3.6 | **Sub-elite group**  Tier 4: Elite / International level  **(“**Inclusion criteria for the sub-elite population compromised of being free of any illness or injury, be currently regularly training ( > 10hr.wk^−1^), and of having represented New Zealand in their given sport in the last 3 years”)  **Recreational group**  Tier 1: Recreationally Active  (“Criteria for inclusion as a recreationally-trained female comprised of being free of any illness or injury and a minimum exercise requirement of at least 3 hr.wk^−1^”) | Cross-sectional  Body composition and RMR measurements  Two days of testing | Harris-Benedict (1918) (age, weight, height)  Mifflin St.Jeor (1990) (age, weight, height)  FAO/WHO/UNU (1985) (age, weight, height) | ParvoMedics True One Metabolic System (Sandy, Utah, USA)  Test duration: 20 minutes  Acclimation/exclusion period: 5 minutes | No body composition  Height and weight only | Yes  **Sub-elite group (N=13)**  Age (y): 32.5 ± 7.4  Weight (kg): 60.9 ± 6.7  Height (m): 1.68 ± 0.08  BMI (kg/m^2^): 22 ± 2  FFM (kg): ?? ± ??  BF (%): ?? ± ??  **Recreational group (N=12)**  Age (y): 27.5 ± 12.3  Weight (kg): 69.0 ± 9.4  Height (m): 1.69 ± 0.07  BMI (kg/m^2^): 24 ± 3  FFM (kg): ?? ± ??  BF (%): ?? ± ?? |
| MacKenzie-Shalders et al.  (2020) | N=18 (0 F)  Age (y): 20.2 ± 1.7  Weight (kg): 101.6 ± 14.5  Height (m): 1.84 ± 0.08  BMI (kg/m^2^): ?? ± ??  FFM (kg): 81.3 ± 8.0  BF (%): ?? ± ?? | Rugby (18 M) | Tier 3: Highly Trained/National Level  (Author reports athletes to be members of an elite rugby development squad; “developing elite rugby union athletes”) | Subsection of a group completing an observational study assessing dietary intake and changes in body composition over a rugby preseason.  Baseline pre- and post-season testing | MacKenzie-Shalders – current population (2020) (weight)  MacKenzie-Shalders – current population (2020) (LBM)  MacKenzie-Shalders – current population (2020) (LBM, FM)  Cunningham (1980) (LBM)  Harris-Benedict 1 (1919) (age, weight, height)  Harris-Benedict 2 (1985) (age, weight, height) | ParvoMedics True One 2400 Metabolic System (Sandy, Utah, USA)  Test duration: 30 minutes  Acclimation/exclusion period: 0 minutes | DXA  (Lunar Prodigy Advance, Encore Version 13.60 software) | Yes  **Baseline pre-season (N=18)**  **Post-season (N=18)**  *only baseline physical characteristics and prediction equation performance extracted. Change in physical characteristics from baseline to post-season is negligible. |
| Marra et al.  (2021) | N=126 (0 F)  Age (y): 26.9 ± 9.1  Weight (kg): 71.3 ± 10.9  Height (m): 1.77 ± 0.07  BMI (kg/m^2^): 22.8 ± 2.7  FFM (kg): ?? ± ??  BF (%): ?? ± ?? | N/a  Unknown ratio of sports played/participated in for the validation group.  The following totals represent the sample in the original study (N=126), from which the validation group was composed of;  Masters swimming (24 M)  Cycling (22 M)  Running (21 M)  Karate (17 M)  Water polo (16 M)  Ballet dance (15 M)  Boxing (11M) | Tier 3: Highly Trained/National Level  "in elite athletes defined as those who have previously competed as regional and/or national players" | Retrospective analysis of elite athletes (N=126)  Body composition and RMR measurements  Unknown days of testing  Validation group compared measured and predicted RMR (N=51) | De Lorenzo (1999) (weight, height)  Harris-Benedict (1918) (age, weight, height)  Mifflin St.Jeor (1990) (age, weight, height)  Owen (1988) (weight)  Schofield (1985) (age, weight, height)  Ten-Haaf (2014) (age, weight, height)  FAO/WHO/UNU (1985) (age, weight, height)  Wong (2012) (weight)  Marra – current population (2021) (age, weight)  Marra – current population (2021) (weight, BIA-derived phase angle) | Vmax® Encore system  (CareFusion Corporation, San Diego, CA, United States)  Test duration: 45 minutes  Acclimation/exclusion period: 5 minutes | BIA  (Human IM Touch, DS Medica S.r.l., Milan, Italy) | Yes  **Calibration group (N=75)**  Age (y): 26.8 ± 9.0  Weight (kg): 71.4 ± 11.3  Height (m): 1.77 ± 0.07  BMI (kg/m^2^): 22.8 ± 2.8  FFM (kg): ?? ± ??  BF (%): ?? ± ??  **used to develop new RMR prediction equation*  **Validation group (N=51)**  **used to validate newly developed RMR prediction equation*  Age (y): 27.1 ± 9.5  Weight (kg): 71.1 ± 10.6  Height (m): 1.77 ± 0.07  BMI (kg/m^2^): 22.8 ± 2.6  FFM (kg): ?? ± ??  BF (%): ?? ± ?? |
| Marques  (2021) | N=7 (7 F)  Age (y): 21.7 ± 3.0  Weight (kg): 62.1 ± 6.0  Height (m): 1.63 ± 0.04  BMI (kg/m^2^): 23.15 ± 1.95  FFM (kg): 47.8 ± 5.6  BF (%): 23.1 ± 2.7 | Karate (7 F) | Tier 3: Highly Trained/National Level  (Author's report "The sample comprised seven high-performance female karate athletes participating in the modalities “kata” (moves) and  “kumite” (fighting).") | Cross-sectional with a retro-analytical component  Body composition and RMR measurements  Unknown number of testing days | Cunningham (1980) (LBM)  FAO/WHO/UNU (1985) (age, weight)  Harris-Benedict (1919) (age, weight, height)  Henry & Rees (1991) (age, weight) | MetaCheck Koor®  (Medical Technologies, Salt Lake City, Utah,  USA)  Test duration: 20 minutes  Acclimation/exclusion period: 0 minutes | Biodynamics 310E device  (San Antonio, TX, United States) | No  **Females only (N=7)** |
| Melin et al.  (2015) | N=40 (40 F)  Age (y): 26.3 ± 5.7  Weight (kg): 58.4 ± 6.9  Height (m): 1.69 ± 0.05  BMI (kg/m^2^): 20.5 ± 1.9  FFM (kg): 46.1 ± 8.4  BF (%): 20.0 ± 3.5 | “Elite endurance athletes” (40 F)  Authors did not report the sport participated in or describe the modality of endurance exercise. Only weekly training volumes reported (h/wk);  **Total (N=40):**  11.4 ± 4.5  **Optimal EA group (N=15):**  9.6 ± 2.8  **Reduced EA group (N=17):**  11.8 ± 4.6  **Low EA group (N=8):**  14.1 ± 5.8 | Tier 3: Highly Trained/National Level  ("13 national team level athletes and 27 from competitive endurance sports clubs.") | Cross-sectional  Medical check, blood samples, VO2 max, food records, and body composition and RMR measurements  Two days of testing | Cunningham (1980) (LBM) | Oxycon Pro 4  (Jeager, Germany)  Test duration: 35 minutes  Acclimation/exclusion period: 15 minutes | DXA  (Hologic, Model Discovery 2009,  Hologic Inc., Waltham, MA, USA) | Yes  **Optimal EA group (N=15)**  **EA* $\geq$ *45 kcal/kg FFM/day* [79]  Age (y): 26.9 ± 6.0  Weight (kg): 56.8 ± 6.3  Height (m): 1.67 ± 0.04  BMI (kg/m^2^): 20.4 ± 1.7  FFM (kg): 44.1 ± 8.4  BF (%): 20.0 ± 3.0  **Reduced EA group (N=17)**  **EA between 30.0 and 44.9 kcal/kg FFM/day* [79]  Age (y): 26.2 ± 6.3  Weight (kg): 58.7 ± 7.3  Height (m): 1.70 ± 0.06  BMI (kg/m^2^): 20.4 ± 2.1  FFM (kg): 46.4 ± 7.8  BF (%): 19.8 ± 3.4  **Low EA group (N=8)**  **EA < 30 kcal/kg FFM/day* [79]  Age (y): 25.5 ± 3.9  Weight (kg): 60.7 ± 7.1  Height (m): 1.71 ± 0.03  BMI (kg/m^2^): 20.6 ± 2.1  FFM (kg): 47.9 ± 6.2  BF (%): 20.5 ± 5.1 |
| Morehan et al.  (2016) | N=6 (0 F)  Age (y): ?? ± ?? (not reported)  Weight (kg): 94.7 ± 6.7  Height (m): 1.83 ± 0.03  BMI (kg/m^2^): 28.34 ± 1.52  FFM (kg): 76.7 ± 4.8  BF (%): 12.4 ± 1.2 | Rugby League forwards (3 M)  Rugby League backs (3 M) | Tier 3: Highly Trained/National Level  ("Six professional RL players from the same club volunteered for the study.") | Cross-sectional  Body composition and RMR measurements  Unknown number of testing days | Cunningham (1980) (LBM) | Moxus Modular Metabolic  System  (AEI Technologies, IL, USA)  Test duration: 15 minutes  Acclimation/exclusion period: 0 minutes | DXA  (Hologic QDR Series, Discovery A,  Bedford, MA, USA) | No  **Males only (N=6)** |
| Moss et al.  (2021) | N=13 (13 F)  Age (y): 23.7 ± 3.4  Weight (kg): 63.7 ± 7.0  Height (m): 1.69 ± 0.08  BMI (kg/m^2^): ?? ± ??  FFM (kg): 49.5 ± 5.3  BF (%): 17.8 ± 4.4 | Soccer (13 F) | Tier 4: Elite / International level  ("Players competed in the top division of the Women’s Super League (WSL1) and trained ∼10 hrs per week.") | Short-term monitoring period  Medical check, medical questionnaires, blood samples, food records, and body composition and RMR measurements  5 days of testing | Cunningham (1980) (LBM) | FitMate™ metabolic system  (Cosmed,  Cosmed, Rome, Italy)  Test duration: 30 minutes  Acclimation/exclusion period: 10 minutes | DXA  (Lunar Prodigy Advance, GE Healthcare Encore  version 14.00.439) | No  **Females only (N=13)** |
| Nichols et al.  (2021)*  *Not included in MA | N=164 (87 F)  Age (y): 31.94 ± 11.11  Weight (kg): 78.35 ± 17.65  Height (m): 1.68 ± 0.10  BMI (kg/m^2^): 27.71 ± 6.45  FFM (kg): 51.98 ± 11.29  BF (%): 32.36 ± 12.03 | Recreational Exercisers (77 M. 87 F) | Tier 1: Recreationally Active  (Participants reported to only exceed WHO PA guidelines. No mention of sport association) | Cross-sectional  Body composition and RMR measurements  Unknown number of testing days | **Anthropometry-based**  Bernstein (1983) (age, weight, height)  De Lorenzo (2001) (age, weight, height)  Harris–Benedict (1918) (age, weight, height)  Harris–Benedict (1985) (age, weight, height)  Henry (2005) (age, weight, height)  Horie & Waitzberg (2011) (FFM, weight)  Huang (2004) (age, weight, height)  Korth (2007) (age, weight, height)  Lazzer (2007) (age, weight, height)  Liu (1995) (age, weight, height)  Livingston & Kohlstadt (2005) (age, weight)  Lührmann (2002) (age, weight)  Mifflin St.Jeor (1990) (age, weight, height)  Müller (MJ/d) (2004) (age, weight, height)  Nicols (2021) (age, weight, height)  Owen (1988) (weight)  Schofield (MJ/d) (1985) (age, weight)  FAO/WHO/UNU (1985) (age, weight, height)  **Body composition (BC)-based**  Bernstein (1983) (FFM, FM, age)  Cunningham (1991) (FFM)  Huang (2004) (FFM, FM, age)  Johnstone (2006) (FFM, FM, age)  Katch & McArdle (2001) (FFM)  Korth (2007) (FFM)  Lazzer (2007) (FFM)  Mifflin-St. Jeor (1990) (FFM)  Müller (MJ/d) (2004) (FFM, FM)  Nicols (2021) (FFM, FM, age)  Owen (1988) (FFM)  Wang (2001) (FFM) | MedGem® IDC  (Microlife, FL, United States)  Test duration: 30 minutes  Acclimation/exclusion period: 20-25 minutes | BIA  (HBF-510 body fat analyser, OMRON Healthcare, Inc. Illinois, USA) | Yes  **Males (N=77)**  Age (y): 29.5 ± 10.6  Weight (kg): 81.0 ± 115.7  Height (m): 1.74 ± 0.08  BMI (kg/m^2^): 26.7 ± 5.6  FFM (kg): 61.0 ± 8.2  BF (%): 23.5 ± 7.9  **Males <25 BMI (N=33)**  *No physical characteristics reported  **Males** $\boldsymbol{\geq}$**25 BMI (N=44)**  *No physical characteristics reported  **Females (N=87)**  Age (y): 34.1 ± 11.1  Weight (kg): 76.0 ± 18.9  Height (m): 1.63 ± 0.07  BMI (kg/m^2^): 28.6 ± 7.0  FFM (kg): 44.0 ± 6.7  BF (%): 40.2 ± 9.3  **Females <25 BMI (N=31)**  *No physical characteristics reported  **Females** $\boldsymbol{\geq}$**25 BMI (N=56)**  *No physical characteristics reported |
| O’Neill et al.  (2022) | N=36 (36 F)  Age (y): 25.47 ± 4.83  Weight (kg): 73.0 ± 10.4  Height (m): 1.68 ± 0.06  BMI (kg/m^2^): 26.0 ± 3.2  FFM (kg): 52.97 ± 5.66  BF (%): 26.95 ± 6.39 | Rugby forwards (19 F)  Rugby backs (17 F) | Tier 4: Elite / International level  ("Elite, has represented nationally, and/or provincially, and/or professionally in the past 12 months") | Cross-sectional  Blood sample, questionnaires, and body composition and RMR measurements  Single day of testing | Cunningham (1980) (LBM)  Harris-Benedict (1918) (age, weight, height)  Jagim (2019) (weight)  O’Neill – current population (2022) (age, weight, height)  O’Neill – current population (2022) (FFM)  Ten-Haaf (2014) (age, weight, height)  Ten-Haaf (2014) (FFM)  Watson (2019) (age, weight, height)  Watson (2019) (FFM) | Quark CPET metabolic cart  (COSMED, Rome, Italy)  Test duration: approx. 20-30 minutes  Acclimation/exclusion period: 10 minutes | BODPOD  (Cosmed, Italy) | Yes  **LEAF-Q <8 group (N=19)**  **scored less than 8 on the LEAF-Q* [80]  Age (y): 26.47 ± 4.60  Weight (kg): 70.29 ± 10.91  Height (m): 1.66 ± 0.05  BMI (kg/m^2^): 25.36 ± 3.75  FFM (kg): 51.91 ± 5.49  BF (%): 25.55 ± 5.94  **LEAF-Q** $\boldsymbol{\geq}$**8 group (N=17)**  **scored 8 or more on the LEAF-Q* [80]  Age (y): 24.35 ± 4.97  Weight (kg): 76.14 ± 9.09  Height (cm): 168.73 ± 6.95  BMI (kg/m^2^): 26.71 ± 2.31  FFM (kg): 54.16 ± 5.77  BF (%): 28.52 ± 6.69 |
| Sjodin et al.  (1996) | N=8 (4 F)  Age (y): 25.5 ± 2.06  Weight (kg): 64.75 ± 11.49  Height (m): 1.73 ± 0.08  BMI (kg/m^2^): ?? ± ??  FFM (kg): 55.70 ± 11.41  BF (%): 15 ± 3.74 | Cross country skiing (4 M, 4 F) | Tier 4: Elite / International level  (“Four female and four male cross-country skiers from top international level”) | Cross-sectional  Body composition and RMR measurements  Two days of testing | Westerterp (MJ/d) (1995) (FFM, FM)  FAO/WHO/UNU (1985) (age, weight, height) | Sensor-Medics 2900Z  (Anaheim, CA)  Test duration: 30 minutes  Acclimation/exclusion period: 0 minutes | Hydrometry by isotope dilution (deuterium) | No  **Males and females combined (N=8)** |
| Staal et al.  (2018) | N=40 (20 F)  Age (y): 25 ± 2.04  Weight (kg): 64.08 ± 12.78  Height (m): 1.73 ± 0.08  BMI (kg/m^2^): 20.20 ± 2.02  FFM (kg): 56.15 ± 11.59  BF (%): 12.65 ± 6.65 | Ballet (20 M, 20 F) | Tier 3: Highly Trained/National Level  ("Inclusion criteria were female and male professional dancers at the Royal Danish Ballet between 18 and 40 years.") | Cross-sectional  Medical check, questionnaires, body composition and RMR measurements  Single day of testing | Cunningham (1980) (LBM)  Harris-Benedict (1918) (age, weight, height)  Koehler DXA-predicted (2016) (brain mass, skeletal muscle mass, adipose tissue mass, bone mass, residual mass) | Oxycon Pro 4  (Jeager, Hoechberg, Germany)  Test duration: 35 minutes  Acclimation/exclusion period: 15 minutes | DXA  (Lunar Prodigy Pro, GE Medical Systems, Madison, WI) | Yes  **Males (N=20)**  Age (y): 24.5 ± 7.5  Weight (kg): 72.8 ± 4.6  Height (m): 1.83 ± 0.04  BMI (kg/m^2^): 21.7 ± 1.4  FFM (kg): 66.7 ± 4.7  BF (%): 8.0 ± 4.8  **Females (N=20)**  Age (y): 25.1 ± 4.8  Weight (kg): 63.95 ± 10.17  Height (m): 1.70 ± 0.04  BMI (kg/m^2^): 18.7 ± 1.3  FFM (kg): 45.6 ± 4.9  BF (%): 17.3 ± 4.47 |
| Stenqvist et al.  (2020) | N=20 (0 F)  Age (y): 33.3 ± 6.7  Weight (kg): 75.8 ± 7.3  Height (m): 1.81 ± 0.05  BMI (kg/m^2^): 23.2 ± 1.9  FFM (kg): 65.5 ± 5.2  BF (%): 14.9 ± 5.2 | Cycling (20 M) | Tier 3: Highly Trained/National Level  ("All participants' were classified at performance level 3–4" = further investigation into the reference accompanying this statement, level 3-4 equates to be "Trained/Well trained") | Longitudinal prospective intervention study  Repeat measures of blood samples, food records, VO2 max, and body composition and RMR measurements  4 weeks duration | Cunningham (1980) (LBM) | Oxycon Pro 4  (Jeager, Hoechberg, Germany)  Test duration: 30 minutes  Acclimation/exclusion period: 10 minutes | DXA  (GE-Lunar Prodigy, Madison, WI, USA, EnCore software  version 15) | Yes  **Pre-testing (N=20)**  **Post-testing (N=20)**  *only pre-testing physical characteristics and prediction equation performance extracted. Change in physical characteristics from pre- to post-testing is negligible. |
| Strock et al.  (2020)  “Strock A” | N=217 (217 F)  Age (y): 21.7 ± 3.5  Weight (kg): 58.74 ± 7.84  Height (m): ?? ± ??  BMI (kg/m^2^): 21.55 ± 2.38  FFM (kg): 43.48 ± 5.08  BF (%): 25.90 ± 5.64 | Recreational exercisers (217 F) | Tier 1: Recreationally Active  (Participants reported to only exceed WHO PA guidelines. No mention of sport association. "≥2 hr of purposeful moderate- to vigorous-intensity exercise per week") | Cross-sectional secondary analysis  Body composition and RMR measurements  Single day of testing | Cunningham (1980) (LBM)  Cunningham (1991) (FFM)  Harris-Benedict (1918) (age, weight, height)  Koehler DXA-predicted (2016) (brain mass, skeletal muscle mass, adipose tissue mass, bone mass, residual mass) | SensorMedics Vmax Series  (CareFusion, Yorba Linda, CA)  Test duration: 30-45 minutes  Acclimation/exclusion period: 0-15 minutes | DXA  (GE Lunar  Prodigy DXA scanner; Madison, WI; enCORE 2002 software, version 6.50.069)  (GE Lunar iDXA scanner; Madison,  WI; enCORE 2008 software version 12.10.113)  (Hologic  QDR4500W DXA scanner; Hologic, Malborough, MA, United States) | Yes  **Ovulatory group (N=73)**  **”exercising eumenorrheic women with ovulatory menstrual cycles and no luteal phase defects”*  Age (y): 24.6 ± 4.6  Weight (kg): 59.9 ± 8.9  Height (m): ?? ± ??  BMI (kg/m^2^): 22.1 ± 2.7  FFM (kg): 43.4 ± 5.6  BF (%): 27.4 ± 5.7  **Amenorrhoea group (N=76)**  **”exercising amenorrhoeic women”*  Age (y): 21.6 ± 3.3  Weight (kg): 56.7 ± 7.0  Height (m): ?? ± ??  BMI (kg/m^2^): 20.7 ± 2.2  FFM (kg): 42.9 ± 4.8  BF (%): 24.0 ± 5.9  **Sub-clinical menstrual disturbances group (N=68)**  **”exercising women with subclinical menstrual disturbances (sMD) including oligomenorrhea, anovulation, and luteal phase defects”*  Age (y): 21.7 ± 3.5  Weight (kg): 60.2 ± 7.0  Height (m): ?? ± ??  BMI (kg/m^2^): 21.9 ± 1.9  FFM (kg): 44.2 ± 4.7  BF (%): 26.4 ± 4.6 |
| Strock et al.  (2020)  “Strock B” | N=14 (14 F)  Age (y): 25.9 ± 5.4  Weight (kg): 59.6 ± 5.2  Height (m): ?? ± ??  BMI (kg/m^2^): 22.3 ± 1.4  FFM (kg): 44.2 ± 4.2  BF (%): 26.1 ± 3.9 | Recreational exercisers (14 F) | Tier 1: Recreationally Active  (Participants reported to only exceed WHO PA guidelines. No mention of sport association. "≥2 hr of purposeful moderate- to vigorous-intensity exercise per week") | Longitudinal study  Medical checks, questionnaires, blood samples, and body composition and RMR measurements  12 months | Cunningham (1980) (LBM)  Cunningham (1991) (FFM)  Harris-Benedict (1918) (age, weight, height)  Koehler DXA-predicted (2016) (brain mass, skeletal muscle mass, adipose tissue mass, bone mass, residual mass) | SensorMedics Vmax Series  (CareFusion, Yorba Linda, CA)  Test duration: 30-45 minutes  Acclimation/exclusion period: 0-15 minutes | DXA  (GE Lunar  Prodigy DXA scanner; Madison, WI; enCORE 2002 software, version 6.50.069) | Yes  **0 month (N=14)**  **3 month (N=14)**  **6 month (N=14)**  **9 month (N=14)**  **12 month (N=14)**  *only 0 month physical characteristics and prediction equation performance extracted. Change in physical characteristics from 0 to 12 months is negligible. |
| Ten Haaf et al.  (2014) | N=90 (37 F)  Age (y): 23.2 ± 4.8  Weight (kg): 70.3 ± 9.7  Height (m): 1.78 ± 0.09  BMI (kg/m^2^): 22.2 ± 2.1  FFM (kg): 59.4 ± 10.4  BF (%): 15.7 ± 6.8 | Athletics – Long distance (5M, 9F)  Athletics – Sprint (3M, 2F)  Cycling (4M, 5F)  Fitness (3M, 5F)  Rowing/Canoeing (7M, 5F)  Swimming (2M, 1F)  Team sports* (17M, 4F)  Remaining sports** (6M, 2F)  *Hockey, korfball, soccer, volleyball  **Dancing, Martial arts, skating, tennis | Tier 2: Trained / Developmental  Although referred to as "Dutch recreational athletes", participants of this study are Level 2: Trained / developmental due to reported training volumes "exercising on average 9.1±5.0 hours a week and 5.0±1.8 times a week" | Cross-sectional  Questionnaires, body composition and RMR measurements  Appears to be single day of testing | Cunningham (1980) (LBM)  De Lorenzo (1999) (weight, height)  Harris-Benedict (1918) (age, weight, height)  Harris-Benedict (1989) (age, weight, height)  Mifflin St.Jeor (1990) (age, weight, height)  Mifflin St.Jeor (1990) (FFM)  Owen (1988) (weight)  Schofield (1985) (age, weight)  Schofield (1985) (age, weight, height)  Ten-Haaf (2014) (age, weight, height)  Ten-Haaf (2014) (FFM)  FAO/WHO/UNU (1985) (age, weight)  FAO/WHO/UNU (1985) (age, weight, height) | Vmax Encore n29 (Viasys Healthcare, Houten, Netherlands)  Test duration: 30 minutes  Acclimation/exclusion period: 5 minutes | BODPOD  (Life Measurement Inc, Concord, CA) | Yes  **Males (N=53)**  Age (y): 23.1 ± 4.7  Weight (kg): 75.7 ± 7.8  Height (m): 1.82 ± 0.09  BMI (kg/m^2^): 22.8 ± 2.0  FFM (kg): 66.73 ± 6.65  BF (%): 11.7 ± 4.9  **Females (N=37)**  Age (y): 23.5 ± 5.0  Weight (kg): 62.6 ± 6.6  Height (m): 1.72 ± 0.05  BMI (kg/m^2^): 21.3 ± 1.9  FFM (kg): 48.92 ± 3.63  BF (%): 21.6 ± 4.7 |
| Thompson et al.  (1993)*  *Not included in MA | N=24 (0 F)  Age (y): 25.54 ± 3.53  Weight (kg): 69.67 ± 7.97  Height (m): 1.78 ± 0.06  BMI (kg/m^2^): 22.06 ± 1.88  FFM (kg): 63.35 ± 7.13  BF (%): 8.96 ± 2.9 | Runners. Triathletes, or biathletes (24 M) | Tier 3: Highly Trained/National Level  (Based on participants self-reported 10km times/ running/cycling/swimming distances per week, and weekly training hours) | Cross-sectional  Body composition and RMR measurements  taken from two separate studies  Multiple testing sessions | Harris-Benedict (1918) (age, weight, height) | SensorMedics Metabolic  Cart  (Anaheim, CA)  Test duration: 30 minutes  Acclimation/exclusion period: 0 minutes | Hydrostatic Weighing  (N/a) | Yes  **Low EI group (N=13)**  **”If the intake was more negative than -800 kcal/d compared to expenditure, the athlete was classified as LOW”*  Age (y): 25 ± 4  Weight (kg): 69.4 ± 6.7  Height (m): 1.75 ± 0.06  BMI (kg/m^2^): 22.6 ± 1.2  FFM (kg): 63.4 ± 5.6  BF (%): 8.8 ± 2.5  **Adequate EI group (N=11)**  **”If the difference was more positive than -350 kcal/d, the athlete was classified as ADQ”*  Age (y): 26 ± 3  Weight (kg): 69.9 ± 8.9  Height (m): 1.80 ± 0.05  BMI (kg/m^2^): 21.6 ± 2.2  FFM (kg): 63.3 ± 8.2  BF (%): 9.1 ± 3.2 |
| Tinsley et al.  (2019) | N=27 (10 F))  Age (y): 25.9 ± 6.0  Weight (kg): 82.9 ± 17.0  Height (m): 176 ± 0.09  BMI (kg/m^2^): 26.6 ± 3.5  FFM (kg): ?? ± ??  BF (%): 15.0 ± 4.4 | Bodybuilder/Physique athlete (17 M, 10 F)  Noteworthy point from the authors*  "The rates of self-reported  anabolic androgenic steroid (AAS) use were 26% (M: 35%, F: 10%) for current use and 41%  (M: 59%, F: 10%) for use in the previous 3 years. However, it is believed that under-reporting of  AAS usage may have occurred." | Tier 3: Highly Trained/National Level  ("To be eligible for inclusion in this analysis, participants were required to meet at least one of the following criteria: 1) have competed in a bodybuilding or physique competition within the last year; 2) have plans to compete within the  next year; or 3) self-identify as a bodybuilder and exhibit a physique commensurate with competitive physique athletes") | Cross-sectional  Body composition and RMR measurements  Single day of testing | **Organ/tissue equation**  Hayes (2002) (brain mass, skeletal muscle mass, adipose tissue mass, bone mass, residual mass)  **FFM-based equations**  Cunningham (1980) (LBM)  Cunningham (1991) (FFM)  Mifflin St.Jeor (1990) (FFM)  Owen (1988) (FFM)  Ten-Haaf (2014) (FFM)  Tinsley – current population (2019) (FFM)  **BW-based equations**  De Lorenzo (1999) (weight, height)  Harris-Benedict (1918) (age, weight, height)  Mifflin St.Jeor (1990) (age, weight, height)  Ten-Haaf (2014) (age, weight, height)  Tinsley – current population (2019) (weight)  FAO/WHO/UNU (1985) (age, weight) | TrueOne® 2400 (ParvoMedics, Sandy, UT, USA)  Test duration: 30 minutes  Acclimation/exclusion period: 5 minutes | DXA  (GE Lunar Prodigy scanner with enCORE software (v. 16.2)) | Yes  **Males (N=17)**  Age (y): 26.0 ± 6.5  Weight (kg): 94.0 ± 9.7  Height (m): 1.80 ± 0.07  BMI (kg/m^2^): 28.8 ± 2.0  FFM (kg): ?? ± ??  BF (%): 12.5 ± 2.7  **Females (N=10)**  Age (y): 25.8 ± 5.4  Weight (kg): 63.8 ± 5.7  Height (m): 1.68 ± 0.06  BMI (kg/m^2^): 22.8 ± 1.6  FFM (kg): ?? ± ??  BF (%): 19.2 ± 3.4 |
| Watson et al.  (2019) | N=66 (66 F))  Age (y): 19.7 ± 1.1  Weight (kg): 67.3 ± 8.9  Height (m): 1.69 ± 0.09  BMI (kg/m^2^): ?? ± ??  FFM (kg): 49.5 ± 5.8  BF (%): 26.2 ± 4.9 | Basketball (9 F)  Field hockey (7 F)  Golf (8 F)  Gymnastics (6 F)  Ice hockey (11 F)  Tennis (3 F)  Softball (8 F)  Volleyball (11 F)  Other (3 F) | Tier 3: Highly Trained/National Level  ("Sixty-six female NCAA Division II athletes") | Cross-sectional  Body composition and RMR measurements  Single day of testing | Cunningham (1980) (LBM)  Harris Benedict (1918) (age, weight, height)  Mifflin-St.Jeor (1990) (age, weight, height)  Owen (1986) (weight)  Schofield (MJ/d) (1985) (age, weight)  Taguchi (2011) (weight)  Watson (2019) (age, weight, height)  Watson (2019) (age, FFM, height)  FAO/WHO/UNU (1985) (age, weight) | Parvo Medics TrueOne Metabolic System (Sandy, UT, USA)  Test duration: 20 minutes  Acclimation/exclusion period: 0 minutes | Skinfolds  (Lange skinfold calliper, unknown brand)  Body density calculated through 3-site skinfold measurements (triceps, suprailiac, and thigh) [81] | No  **Females only (N=66)** |
| Wong et al.  (2012) | N=125 (33 F))  Age (y): 20.9 ± 2.64  Weight (kg): 60.75 ± 9.0  Height (m): 1.66 ± 0.08  BMI (kg/m^2^): 22.05 ± 2.52  FFM (kg): 50.15 ± 9.08  BF (%): 17.7 ± 5.66 | Combat sports (boxing, karate, taekwondo, silat and wushu)  (N=44)  Racquet sports (squash and badminton)  (N=28)  Team sports (football and  hockey)  (N=22)  Skilled sports (fencing and archery) (N=21)  Others (diving, artistic gymnastics, rhythmic gymnastics, weightlifting) (N=10)  *Ratios of males to females for each sport not reported | Tier 3: Highly Trained/National Level  (“The inclusion criteria for participants  included: (a) men or women in the adult age group; (b) athletes  who had been undergoing sports training for an average of six  hours a day; (c) athletes who had been training centrally under  the national elite sports programme for a minimum of one year  in their specialised sport;") | Cross-sectional  Body composition and RMR measurements  Two days of testing | Cunningham (1980) (LBM)  De Lorenzo (1999) (weight, height)  Harris-Benedict (1918) (age, weight, height)  Ismail (MJ/d) (1998) (weight)  FAO/WHO/UNU (1985) (age, weight)  Wong – current population (2012) (weight) | Deltatrac Metabolic Monitor MBM-200  (Datex-Ohmeda, Helsinki, Finland)  Test duration: 40 minutes  Acclimation/exclusion period: 10 minutes | BIA  (Bodystat Model 1500 MDD; Bodystat Ltd, Isle of Man, British Isles). | Yes  **Males (N=92)**  Age (y): 21.4 ± 3.0  Weight (kg): 66.1 ± 8.5  Height (m): 1.71 ± 0.07  BMI (kg/m^2^): 22.7 ± 2.8  FFM (kg): 57.1 ± 7.4  BF (%): 13.7 ± 4.0  **Females (N=33)**  Age (y): 20.4 ± 2.1  Weight (kg): 55.4 ± 5.7  Height (m): 1.61 ± 0.04  BMI (kg/m^2^): 21.4 ± 2.0  FFM (kg): 43.2 ± 3.7  BF (%): 21.7 ± 4.0 |

**Title of supplementary material:** Supplementary Document 6 – List of included sports (by category)

*Please note that the list of sports below do not represent distinct studies as multiple studies included participants from several sports

| **Classification / Sport (Reference(s))** | **Males** | **Females** | **Total** |
| --- | --- | --- | --- |
| **Team/Field sports** |  |  |  |
| American football [12], [22] | 77 | 0 | 77 |
| Baseball [12], [22] | 11 | 0 | 11 |
| Basketball | 0 | 9 | 9 |
| Field hockey [25] | 0 | 7 | 7 |
| Ice hockey [25] | 0 | 11 | 11 |
| Rugby League [10] | 6 | 0 | 6 |
| Rugby Union [9], [60] | 18 | 36 | 54 |
| Soccer [12], [22], [32], [52], [55] | 2 | 63 | 65 |
| Softball [25] | 0 | 8 | 8 |
| Volleyball [25] | 0 | 11 | 11 |
| Waterpolo [19] | 22 | 0 | 22 |
| Team/Field sports total | 136 | 145 | 281 |
| **Endurance sports** |  |  |  |
| Cycling [4], [21] | 26 | 4 | 30 |
| Recreational aerobic exercisers [58] | 0 | 57 | 57 |
| Runners. Triathletes, or biathletes [56] | 24 | 0 | 24 |
| Swimming [12], [21], [22], [32] | 6 | 11 | 17 |
| Triathletes [27] | 10 | 6 | 16 |
| Ultra-marathoners [27] | 5 | 9 | 14 |
| Undefined "Elite endurance athletes” [53] | 0 | 40 | 40 |
| Endurance sports total | 71 | 127 | 198 |
| **Combat sports** |  |  |  |
| Boxing [32] | 3 | 3 | 6 |
| Fencing [32] | 1 | 2 | 3 |
| Judo [19] | 12 | 0 | 12 |
| Karate [19], [32], [62] | 22 | 12 | 34 |
| Taekwondo [32], [54] | 6 | 0 | 6 |
| Combat sports total | 44 | 17 | 61 |
| **Track & Field** |  |  |  |
| Track & field (distance) [22] | 9 | 27 | 36 |
| Track & field (sprinters) [22] | 3 | 8 | 11 |
| Track & field (throwers) [22] | 0 | 3 | 3 |
| Track & field (unspecified) [12], [32] | 8 | 7 | 15 |
| Track & Field total | 20 | 45 | 65 |
| **Aesthetic sports** |  |  |  |
| Ballet [3] | 20 | 20 | 40 |
| Bodybuilder/Physique athlete [23] | 17 | 10 | 27 |
| Gymnastics [25] | 4 | 11 | 15 |
| Aesthetic sports total | 41 | 41 | 82 |
| **Other** |  |  |  |
| Rowing [14] | 3 | 5 | 8 |
| Rowing or canoeing [21] | 7 | 5 | 12 |
| Canoe racing [14] | 5 | 4 | 9 |
| Cross country skiing [59] | 4 | 4 | 8 |
| Golf [25] | 0 | 8 | 8 |
| Modern pentathlon [32] | 1 | 4 | 5 |
| Recreational concurrent exercises [58] | 0 | 19 | 19 |
| Recreational resistance training exercisers [58] | 0 | 3 | 3 |
| Tennis [22], [25] | 0 | 5 | 5 |
| Weightlifting [28] | 30 | 0 | 30 |
| Other total | 50 | 57 | 107 |
| **Unattributable/Unspecified** |  |  |  |
| “Fitness” [21] | 3 | 5 | 8 |
| Other [25] | 0 | 3 | 3 |
| Recreational exercisers [58] | 77 | 330 | 407 |
| “Remaining sports” [21] | 6 | 2 | 8 |
| “Team sports” [21] | 17 | 4 | 21 |
| Unattributable [33] | 51 | 0 | 51 |
| Unattributable [61] | 92 | 33 | 125 |
| Unspecified "Sub-elite" [29] | 0 | 13 | 13 |
| Unattributable/Unspecified total | 246 | 390 | 636 |
| Sum total of all categories | 608 | 822 | 1430 |

**Title of supplementary material:** Supplementary Document 7 - Chronological list of prediction equations used in studies in athletes, and based on athlete samples.

| **General population-based equations used in studies of athletes** | | | | |  |  |
| --- | --- | --- | --- | --- | --- | --- |
| **Equation Name (year) (components)** | **RMR Equation** | **Population equation based on** | | **Bias > 10% (population, % bias, (precision %))** | **Bias <10% (population, % bias, (precision %))** | **Bias <5% (population, (precision %))** |
|  |  | **Number (male (M), female (F)), age** | **Other characteristics described** |  |  |  |
| Harris-Benedict (1918)  (age, weight, height) [7] | **Males (kcal/24h):** 66.473 + (13.7516 x wt (kg)) + (5.0033 x ht (cm)) – (6.755 x Age (yrs))  **Females (kcal/24h):** 655.0955 + (9.5634 x wt (kg)) + (1.8496 x ht (cm)) – (4.6756 x Age (yrs)) | n=136 M, n = 103 F; 21-70yrs | Wt, 25 – 124.9 kg; Ht, 151 – 200 cm. In good health, typical of the general population. N=16 athletes (all male) included: wt, 56.3 – 108.9 kg; ht, 160 – 198 cm; 19 – 29 yrs. | **Overestimation**  Carlsohn (2011) – Female, Rowers/Canoeists, 10%, (P NR%)  McKay (2019) - Female recreational exercisers, 18%, (P NR%)  Staal (2018) – Male and female, Ballet, males: 12%, females: 12% (Males: P 75%, Females: P 55%)  Strock A (2020) – Females, Recreational exercisers, ovulatory group: 14%, amenorrheic group: 20%, sub-clinical menstrual dysfunction: 16% (P NR%)  Nichols (2020) - <25 BMI Females, recreational exercisers, 7.4% (<25 BMI: P 39%)  **Underestimation**  Carlsohn (2011) – Male, Rowers/Canoeists, 20%, (P NR%)  Devrim-Lampir (2017) – Male and female, Ultra-endurance, males: 17%, females: 26%, (Males: P 20%, Females: P 55%)  Jagim (2019) - Males, NCAA Div III mixed sports, 11%, (P NR%)  Joseph (2017) - Male, Indian weightlifters, 19%, (P 33%)  McKay (2019) - Female, Sub-elite endurance, 15%, (P NR%)  Marques (2021) – Female, karate, 14%, (P NR%)  Thompson (1993) – Males, runners, triathletes, and biathletes, negative energy balance group, 12%, (P NR%)  Tinsley (2019) - Male, Bodybuilders, 11%, (P NR%)  Freire, (2023) – Male, Brazilian National & Olympic Team, 19%, (P 16%)** | **Overestimation**  Balci (2021) – Female, members of the Turkish National Olympic Team, 8%**,** (P 50%)  Strock B (2020) – Female, recreational exercisers, 9%, (P NR%)  Nichols (2020) - >/<25 BMI Males and >25 BMI Females, recreational exercisers, male <25 BMI: 7%, male >25 BMI: 5%, female >25 BMI: 3% (Male <25 BMI: P 46%, Male >25 BMI: P 55%, Female >25 BMI: P 39%)  **Underestimation** O’Neill (2022) - Female, Rugby, 6%, (P 75%)  Tinsley (2019) – Female, Bodybuilders, 7%, (P NR%)  Freire, (2023) – Female, Brazilian National & Olympic Team, 6%, (P 59%)**  Mackenzie-Shalders (2020) – Male, Elite Rugby, 6%, (P NR%)  Ten-Haaf (2014) – Males and females, Mix of athletics, endurance, and team sports, males: 7%, females: 8% (Males: P 68%, Females: P 59%) | Balci (2021) – Males, members of the Turkish National Olympic Team (P 40%)  De Lorenzo (1999) – Males, waterpolo, judo, and karate (P NR%)  Thompson (1993) – Males, runners, triathletes, and biathletes, adequate energy balance group (P NR%),  Marra (2021) – Males, Mix of endurance, combat, and water polo, (P 75%)  Jagim (2019) – Females, NCAA Div III mixed sports, (P NR%)  Watson (2019) – Females, NCAA Div II mixed sports, (P NR%)  Wong (2012) – Males & Females, Mix of combat, team, and skilled sports, (P NR%) |
| Fleisch (1951)  (BSA) [83] | Published tables to determine kilocalories per square meter of body surface per hour, where BSA is the formula of DuBois (1916)  BSA = (wt (kg) 0.425 x ht (cm) 0.725) x 0.007184. | NA |  | **Underestimation**  De Lorenzo (1999) – Males, waterpolo, judo, and karate, NR%, (P NR%) |  |  |
| Robertson & Reid (1952)  (BSA) [84] | Published tables to determine kilocalories per square meter of body surface per hour, where BSA is the formula of DuBois (1916)  BSA = (wt (kg) 0.425 x ht (cm) 0.725) x 0.007184 | n=883 M, n=1184 F; 3-40yrs |  | **Underestimation**  De Lorenzo (1999) – Males, waterpolo, judo, and karate, NR%, (P NR%) |  |  |
| Cunningham (1980)  (FFM) [8] | **Unisex (kcal/24h):** 500 + (22 x LBM (kg)) | n=223 M and F, add age, height, weight range | Healthy untrained based on participants of the Harris-Benedict study, athletes were excluded | **Overestimation**  Carlsohn (2011) – Female, Rowers/Canoeists, 10%, (P NR%)  Morehen (2016) – Male, Elite rugby, 17%, (P 17%%)  Staal (2018) – Male and female, Ballet, males: 16%, females: 24% (Males: P 20%, Females: P 0%)  Stenqvist (2020) - Male, Cyclists, 10%, (P NR%)    Strock A (2020) – Females, Recreational exercisers, ovulatory group: 13%, amenorrheic group: 19%, sub-clinical menstrual dysfunction: 14% (P NR%)  Strock B (2020) – Female, recreational exercisers, 9%, 10%, (P NR%)  **Underestimation**  Devrim-Lampir (2017) – Female, Ultra-endurance, 17%, (P 47%)  Joseph (2017) - Male, Indian weightlifters, 17%, (P 20%)  Melin (2015) - Female, elite endurance, 10%, (P NR%)  Carlsohn (2011) – Male, Rowers/Canoeists, 16%, (P NR%) | **Overestimation**  Devrim-Lampir (2017) – Male, Ultra-endurance, 7%, (P 47%)  Moss (2021) - Female, Soccer, 6%, (P NR%)  Watson (2019) - NCAA Div. II mixed sports, 8%, (P NR%)  **Underestimation**  Jagim (2019) - Males, NCAA Div III mixed sports, 7%, (P NR%)  Marques (2021) – Female, karate, 8%, (P NR%) | De Lorenzo (1999) – Males, waterpolo, judo, and karate, (P NR%)  Jagim (2019) - Females, NCAA Div III mixed sports, (P NR%)  Langan-Evans (2020) - n=1 male taekwondo athlete (P NR%)  Mackenzie-Shalders (2020) - Male, Elite Rugby, (P NR%)  O’Neill (2022) - Female, Rugby, (P 81%)  Tinsley (2019) – Male and female, Bodybuilders, (P NR%)  Wong (2012) – Males & Females, Mix of combat, team, and skilled sports, (P NR%)  Ten-Haaf (2014) – Males and females, Mix of athletics, endurance, and team sports, (Males: P 85%, Females: P 78%) |
| Bernstein (1983)  (age, weight, height) [85] | **Males (kcal/24h):** (11.02 x Wt (kg)) + (10.23 x Ht (cm)) – (5.8 x age (yrs)) - 1032  **Females (kcal/24h):** (7.48 x Wt (kg)) + (0.42 x Ht (cm)) - (3 x age (yrs)) - 1032 | n=48 M, n=154 F | Individuals with obesity enrolled in a weight reduction program | **Underestimation**  Nichols (2020) - >/<25 BMI Males and >/<25 BMI Females, recreational exercisers, male <25 BMI: 15%, male >25 BMI: 16%, female <25 BMI: 12%, female >25 BMI: 16% (Male <25 BMI: P 30%, Male >25 BMI: P 21%, Female <25 BMI: P 52%, Female >25 BMI: P 25%) |  |  |
| Bernstein (1983)  (FFM, FM, age) [85] | **Unisex (kcal/24h):** 19.02 x FFM (kg) + 3.72 x FM (kg) + 236.7 | “” |  | **Overestimation**  Balci (2021) – Female, members of the Turkish National Olympic Team, 17%**,** (P 17%)  Nichols (2020) - >/<25 BMI Males and >/<25 BMI Females, recreational exercisers, male <25 BMI: 21%, male >25 BMI: 14%, female <25 BMI: 26%, female >25 BMI: 18% (Male <25 BMI: P 12%, Male >25 BMI: P 32%, Female <25 BMI: P 13%, Female >25 BMI: P 27%)  **Underestimation**  Balci (2021) – Males, members of the Turkish National Olympic Team, 19%, (P 20%) |  |  |
| Harris–Benedict (1984) by Roza & Shizgal  (age, weight, height) [86] | **Males (kcal/24h):** 88.362 + (13.397 x wt (kg)) + (4.799 x ht (cm)) – (5.677 x Age (yrs))  **Females (kcal/24h):** 447.593 + (9.247 x wt (kg)) + (3.098 x ht (cm)) – (4.330 x Age (yrs)) | n=168 M, n=169 F | Mainly normal weight subjects |  | **Overestimation**  Balci (2021) – Female, members of the Turkish National Olympic Team, 9%**,** (P 38%)  Nichols (2020) - <25 BMI Males and <25 BMI Females, recreational exercisers, male <25 BMI: 6%, female <25 BMI: 7% (Male <25 BMI: P 52%, Female <25 BMI: P 42%)  **Underestimation**  Ten-Haaf (2014) – Males, Mix of athletics, endurance, and team sports, 7%, (P 68%)  Mackenzie-Shalders (2020) – Male, Elite Rugby, 7%, (P NR%) | Balci (2021) – Males, members of the Turkish National Olympic Team, (P 24%)  Ten-Haaf (2014) – Females, Mix of athletics, endurance, and team sports, (P 76%)  Nichols (2020) - >25 BMI Males and >25 BMI Females, recreational exercisers, male >25 BMI: 5%, female >25 BMI: 3% (Male >25 BMI: P 55%, Female >25 BMI: P 61%) |
| Schofield (1985) (age, weight, height) [87] | **Males 18-30 (MJ/24h):** 63 x Wt (kg) -42 Ht + 2953  **Males 30-60 (MJ/24h):** 48 x Wt (kg) -11 Ht + 3670  **Females 18-30 (MJ/24h):** 62 x Wt (kg) + 1148 Ht + 411  **Females 30-60 (MJ/24h):** 34 x Wt (kg) + 6 Ht + 3530 |  |  |  | **Overestimation**  Nichols (2020) - <25 BMI Males, recreational exercisers, 8%, (P 50%)  Nichols (2020) - >25 BMI Males, recreational exercisers, 7%, (P 46%)  **Underestimation**  Ten-Haaf (2014) – Males, Mix of athletics, endurance, and team sports, 10%, (P 47%)  Ten-Haaf (2014) – Females, Mix of athletics, endurance, and team sports, 9%, (P 57%) | Watson (2019) – Females, NCAA Div II mixed sports, (P NR%)  Nichols (2020) - <25 BMI Females, recreational exercisers, 3%, (P 58%)  Nichols (2020) - >25 BMI Females, recreational exercisers, 2%, (P 59%) |
| Schofield (1985)  (age, weight) [87] | **Males 18-30 (MJ/24h):** 63 x Wt (kg) + 2896  **Males 30-60 (MJ/24h):** 48 x Wt (kg) + 3653  **Females 18-30 (MJ/24h):** 62 x Wt (kg) + 2036  **Females 30-60 (MJ/24h):** 34 x Wt (kg) + 3538 | n=3575 M, n=1239 F | 47% were Italian | **Underestimation**  Ten-Haaf (2014) – Females, Mix of athletics, endurance, and team sports, 12%, (P 41%)  Ten-Haaf (2014) – Males, Mix of athletics, endurance, and team sports, 10%, (P 47%) | **Overestimation**  Balci (2021) – Female, members of the Turkish National Olympic Team, 7%**,** (P 54%)  **Underestimation**  Marra (2021) – Males, Mix of endurance, combat, and water polo, 5%, (P 75%) | Balci (2021) – Males, members of the Turkish National Olympic Team (P 44%) |
| FAO/WHO/UNU (1985)  (age, weight, height) [26] | **Males 18-30(kcal/24h):** 15.4 x Wt (kg) - 27 x Ht (m) + 717  **Males 30-60 (kcal/24h):** 11.3 x Wt (kg) - 16 x Ht (m) + 901  **Females 18-30(kcal/24h):** 13.3 x Wt (kg) + 334 x Ht (m) + 35  **Females 30-60 (kcal/24h):** 8.7 x Wt (kg) - 25 x Ht (m) + 865 | Unclear numbers for each individual equation (based on n=11,000 overall including M, F and children) | Based on data mainly from Schofield (1985) | **Underestimation**  Devrim-Lampir (2017) – Male and female, Ultra-endurance, males: 15%, females: 26%, (Males: P 20%, Females: P 13%)  Marques (2021) – Female, karate, 17%, (P NR%)  Sjodin (1996) – Male and female cross-country skiers, 14%, (P NR%)  Ten-Haaf (2014) – Females, Mix of athletics, endurance, and team sports, 11%, (P 49%)  Ten-Haaf (2014) – Males, Mix of athletics, endurance, and team sports, 10%, (P 47%) | **Overestimation**  Nichols (2020) - <25 BMI Males, recreational exercisers, 6%, (P 52%)  Nichols (2020) - >25 BMI Males, recreational exercisers, 5%, (P 50%) | De Lorenzo (1999) – Males, waterpolo, judo, and karate (P NR%)  Marra (2021) – Males, Mix of endurance, combat, and water polo, (P 75%)  Nichols (2020) - <25 BMI Females, recreational exercisers, 4%, (P 58%)  Nichols (2020) - >25 BMI Females, recreational exercisers, 3%, (P 54%) |
| FAO/WHO/UNU (1985)  (age, weight) [26] | **Males 18-30(kcal/24h):** 15.3 x Wt (kg) + 679  **Males 30-60 (kcal/24h):** 11.6 x Wt (kg) + 879  **Females 18-30(kcal/24h):** 14.7 x Wt (kg) + 496  **Females 30-60 (kcal/24h):** 8.7 x Wt (kg) + 829 | “” |  | **Overestimation**  McKay (2019) - Female, Recreational exercisers, 20%, (P NR%)  **Underestimation**  Devrim-Lampir (2017) – Male and female, Ultra-endurance, males: 14%, females: 22%, (Males: P 20%, Females: P 27%)  Joseph (2017) - Male, Indian weightlifters, 18%, (P 37%)  McKay (2019) - Female, Sub-elite endurance, 14%, (P NR%)  Ten-Haaf (2014) – Females, Mix of athletics, endurance, and team sports, 11%, (P 43%)  Ten-Haaf (2014) – Males, Mix of athletics, endurance, and team sports, 9%, (P 51%) | **Underestimation**  Wong (2012) – Males & Females, Mix of combat, team, and skilled sports, (P NR%) | Wong (2012) – Males Mix of combat, team, and skilled sports, (P NR%)  Watson (2019) – Females, NCAA Div II mixed sports, (P NR%) |
| Owen (1987)  (weight) [16] | **Males (kcal/24h):** 879 + (10.2 x wt (kg)  **Females (kcal/24h):** 795 + (7.18 x wt (kg)) | n=60 M; 18–82 years. | Lean individuals and individuals with obesity. Wt, 60–171 kg; ht, 163–188 cm. Trained athletes were excluded. | **Underestimation**  Marra (2021) – Males, Mix of endurance, combat, and water polo, 12%, (P 45%)  Watson (2019) – Females, NCAA Div II mixed sports, 13% (P NR%)  Ten-Haaf (2014) – Females, Mix of athletics, endurance, and team sports, 22%, (P 5%)  Ten-Haaf (2014) – Males, Mix of athletics, endurance, and team sports, 18%, (P 15%) | **Underestimation**  Nichols (2020) - <25 BMI Females, recreational exercisers, 6%, (P 61%)  Nichols (2020) - >25 BMI Females, recreational exercisers, 9%, (P 43%) | Nichols (2020) - <25 BMI Males, recreational exercisers, (P 85%)  Nichols (2020) - >25 BMI Males, recreational exercisers, (P 64%) |
| Owen (1987)  (FFM) [16] | **Unisex (kcal/24h):** 290 + (22.3 x FFM (kg)) | “” |  | **Underestimation**  Tinsley (2019) – Female, Bodybuilders, 17%, (P NR%)  Tinsley (2019) - Male, Bodybuilders, 12%, (P NR%)  Nichols (2020) - >25 BMI Females, recreational exercisers, 19%, (P 46%)  Ten-Haaf (2014) – Females, Mix of athletics, endurance, and team sports, 19%, (P 11%)  Ten-Haaf (2014) – Males, Mix of athletics, endurance, and team sports, 12%, (P 43%) | **Overestimation**  Nichols (2020) - <25 BMI Females, recreational exercisers, 10%, (P 61%)  **Underestimation**  De Lorenzo (1999) – Males, waterpolo, judo, and karate, 7%, (P NR%)  Nichols (2020) - >25 BMI Males, recreational exercisers, 7%, (P 66%) | Nichols (2020) - <25 BMI Males, recreational exercisers, (P 85%) |
| Owen (1998) (age, weight, height) [16] | **(kcal/24h)** = 655.096 + 1.8496 (height in cm) + 9.5634 (weight in kg) − 4.6759 (age) |  |  | **Underestimation**  Joseph (2017) - Male, Indian weightlifters, 29%, (P 10%) |  |  |
| Indian Council of Medical Research (1990)  (age, weight) [88] | **Males 18-30(kcal/24h):** 14.5 x Wt (kg) + 645  **Males 30-60 (kcal/24h):** 10.9 x Wt (kg) + 833  **Females 18-30(kcal/24h):** 14.0 x Wt (kg) + 471  **Females 30-60 (kcal/24h):** 8.3 x Wt (kg) + 788 | NA |  | **Underestimation**  Joseph (2017) - Male, Indian weightlifters, 22%, (P 27%) |  |  |
| Mifflin-St Jeor (1990)  (age, weight, height) [17] | **Males (kcal/24h):** (9.99 x wt (kg)) + (6.25 x ht (cm)) – (4.92 x age (yrs)) + 5  **Females (kcal/24h):** (9.99 x wt (kg)) + (6.25 x ht (cm)) – (4.92 x age (yrs)) -161 | n=251 M, n=247 F; 19–78 yrs | Normal wt and individuals with overweight and obesity. Normal wt: 80 to <119% ideal body weight; obese: ≥120% ideal body weight; Ht, 146–201 cm | **Overestimation**  McKay (2019) - Female, Recreational exercisers, 15%, (P NR%)  **Underestimation**  Devrim-Lampir (2017) – Female, Ultra-endurance, 10%, (P 20%)  Jagim (2019) - Males, NCAA Div III, 17%, (P NR%)  Jagim (2019) – Females, NCAA Div III, 8% (P NR%)  McKay (2019) - Female, Sub-elite endurance, 16%, (P NR%)  Joseph (2017) - Male, Indian weightlifters, 23%, (P 23%)  Tinsley (2019) – Female, Bodybuilders, 11%, (P NR%)  Tinsley (2019) - Male, Bodybuilders, 17%, (P NR%) | **Underestimation**  Marra (2021) – Males, Mix of endurance, combat, and water polo, (P 65%)  De Lorenzo (1999) – Males, waterpolo, judo, and karate, 7%, (P NR%) | Devrim-Lampir (2017) – Female, Ultra-endurance, (P 47%)  Balci (2021) – Males and females, members of the Turkish National Olympic Team (Males: P 40%, Females: P 71%)  Watson (2019) – Females, NCAA Div II mixed sports, (P NR%)  Nichols (2020) - <25 BMI Females, recreational exercisers, (P 58%)  Nichols (2020) - >25 BMI Females, recreational exercisers, (P 48%)  Nichols (2020) - <25 BMI Males, recreational exercisers, (P 67%)  Nichols (2020) - >25 BMI Males, recreational exercisers, (P 68%) |
| Mifflin St Jeor (1990)  (FFM) [17] | **Unisex (kcal/24h):** 19.7 x FFM (kg) + 413 | “” |  | **Underestimation**  Tinsley (2019) – Female, Bodybuilders, 12%, (P NR%)  Tinsley (2019) - Male, Bodybuilders, 15%, (P NR%)  Nichols (2020) - >25 BMI Females, recreational exercisers, 14%, (P 35%)  Nichols (2020) - >25 BMI Males, recreational exercisers, 10%, (P 48%) | **Underestimation**  Balci (2021) – Male, members of the Turkish National Olympic Team, 7%, (P 60%)  Balci (2021) – Female, members of the Turkish National Olympic Team, 6%, (P 58%)  Nichols (2020) - <25 BMI Females, recreational exercisers, 8%, (P 52%) | Nichols (2020) - <25 BMI Males, recreational exercisers, (P 76%) |
| Cunningham (1991)  (FFM) [63] | (kcal/24h) = 370 + (21.6 x FFM (kg)) | n=1483 M and F; adults | Weighted mean equation from previously published studies. Individuals with and without obesity | **Overestimation**  Balci (2021) – Female, members of the Turkish National Olympic Team, 12%, (P 54%)  Strock A (2020) – Females, Recreational exercisers, amenorrheic group: 11%, (P NR%)  **Underestimation**  Tinsley (2019) – Female, Bodybuilders, 11%, (P NR%)  Tinsley (2019) - Male, Bodybuilders, 9%, (P NR%) | **Overestimation**  Balci (2021) – Male, members of the Turkish National Olympic Team, 6%, (P 40%)  Strock A (2020) – Females, Recreational exercisers, ovulatory group: 6%, sub-clinical menstrual dysfunction: 7% (P NR%)  Nichols (2020) - <25 BMI Males, recreational exercisers, 9%, (P 55%)  **Underestimation**  Nichols (2020) - <25 BMI Females, recreational exercisers, 6%, (P 61%) | Strock B (2020) – Female, recreational exercisers, (P NR%)  Nichols (2020) - >25 BMI Females, recreational exercisers, (P 64%)  Nichols (2020) - >25 BMI Males, recreational exercisers, (P 52%) |
| Henry & Rees (1991)  (age, weight) [89] | **Unisex 18 – 30 (kcal/24h):** ((0.048 x wt (kg)) + 2.562) x 239 | Male and female adults living in the tropics |  | **Underestimation**  Marques (2021) – Female, karate, 22%, (P NR%) |  |  |
| Nelson (1992)  (FFM, FM) [18] | **Unisex (kcal/24h):** (25.8 x FFM (kg)) + (4.04 x FM (kg)) | Based on data from previous studies that included measurement of FFM. Inactive individuals with and without obesity. | Based on data from previous studies that included measurement of FFM. Inactive individuals with and without obesity | **Underestimation** Joseph (2017) - Male, Indian weightlifters, 42%, (P 10%)  Jagim (2019) - Males, NCAA Div III, 14%, (P NR%)  Jagim (2019) – Females, NCAA Div III, 13% (P NR%) | **Underestimation**  Balci (2021) – Males, members of the Turkish National Olympic Team, 8% (P 60%) | Balci (2021) – Female, members of the Turkish National Olympic Team, (P 33%) |
| Liu (1995)  (age, weight, height) [90] | **Males (kcal/24h):** 13.88 x Wt (kg) + 4.16 x Ht (cm) – 3.43 x Age (yrs) + 54.34  **Females (kcal/24h):** 13.88 x Wt (kg) + 4.16 x Ht (cm) – 3.43 x Age (yrs) – 58.06 | n=102 M, n=121 F; 20- 78 yrs | 223 healthy Chinese adults | **Overestimation**  Nichols (2020) - <25 BMI Females, recreational exercisers, 14% (P 48%)  Nichols (2020) - >25 BMI Females, recreational exercisers, 21% (P 25%) | **Underestimation**  Balci (2021) – Males, members of the Turkish National Olympic Team, 8% (P 48%) | Balci (2021) – Female, members of the Turkish National Olympic Team, (P 67%)  Nichols (2020) - <25 BMI Males, recreational exercisers, (P 67%)  Nichols (2020) - >25 BMI Males, recreational exercisers, (P 61%) |
| Westerterp (1995) (FFM, FM) [91] | **Unisex (Mj/24h):** (0.102 x FFM (kg)) + (0.024 x FM (kg)) + 0.85 | NA |  | **Underestimation**  Sjodin (1996) – Male and female cross-country skiers, 12%, (P NR%) |  |  |
| LARN (1996) [92] | NA | NA |  | **Underestimation**  Gravante (2001) – Female soccer players, 13%, (P NR%) |  |  |
| Ismail (MJ/d) (1998)  (weight) [93] | **Males (MJ/24h):** (0.055 * Wt (kg)) + 2.480  **Females (MJ/24h):** (0.0535 * Wt (kg)) + 1.994 | n=307 M, n=349 F, 18-60 yrs | Healthy Malaysian adults | **Underestimation**  Wong (2012) – Males & Females, Mix of combat, team, and skilled sports, Males: 15%, Females: 14%, (P NR%) |  |  |
| Wang (2000)  (FFM) [24] | **Unisex (kcal/24h):** 21.5 x FFM (kg) + 407 | Composite FFM equation derived from n=15 other FFM based RMR prediction equations |  | **Underestimation**  Devrim-Lampir (2017) – Male and female, Ultra-endurance, males: 15%, females: 28%, (Males: P 27%, Females: P 13%) | **Underestimation**  Nichols (2020) - >25 BMI Females, recreational exercisers, 9%, (P 45%) | Nichols (2020) - <25 BMI Females, recreational exercisers, (P 61%)  Nichols (2020) - <25 BMI Males, recreational exercisers, (P 79%)  Nichols (2020) - >25 BMI Males, recreational exercisers, (P 64%) |
| Katch & McArdle (2001) (FFM) [94] | **Kcal/24h =** 9.7976 x FFM (kg) + 370 | Not available |  | **Underestimation** Joseph (2017) - Male, Indian weightlifters, 24%, (P 13%)  Nichols (2020) - >25 BMI Females, recreational exercisers, 11%, (P 40%) | **Underestimation**  Nichols (2020) - <25 BMI Females, recreational exercisers, 5%, (P 52%)  Nichols (2020) - >25 BMI Males, recreational exercisers, 5%, (P 61%) | Nichols (2020) - <25 BMI Males, recreational exercisers, (P 88%) |
| Hayes (2002)  (brain mass, skeletal muscle mass, adipose tissue mass, bone mass, residual mass) [95] | **Unisex (kcal/24h):** (240 x brain mass (kg)) + (13 x skeletal muscle mass (kg)) + (2.3 x bone mass (kg)) + (4.5 x adipose tissue mass (kg)) + (43 x resisdual mass (kg)) | Developed from 65 healthy males (n=17) and females (n=48) under the age of 50 |  | **Underestimation**  Tinsley (2019) – Female, Bodybuilders, 7%, (P NR%)  Tinsley (2019) - Male, Bodybuilders, 8%, (P NR%) |  |  |
| Lührmann (2002)  (age, weight) [96] | **Males (kj/24h):** 50 x Wt (kg) – 15.3 x Age (yrs) + 3915  **Females (kj/24h):** 50 x Wt (kg) – 15.3 x Age (yrs) + 3169 | Elderly males and females (ratio of males to females unknwon) |  |  | **Overestimation**  Nichols (2020) - >25 BMI Females, recreational exercisers, 6%, (P 52%) | Nichols (2020) - <25 BMI Females, recreational exercisers, (P 52%)  Nichols (2020) - <25 BMI Males, recreational exercisers, (P 64%)  Nichols (2020) - >25 BMI Males, recreational exercisers, (P 55%) |
| Huang (2004)  (age, weight, height) [97] | **Males (kcal/24h):** 10.158 x Wt (kg) + 3.933 x Ht (cm) – 1.44 x Age (yrs) + 334.476  **Females (kcal/24h):** 10.158 x Wt (kg) + 3.933 x Ht (cm) – 1.44 x Age (yrs) + 60.655 | Obese and severely obese male and female adults (n=1088; mean age = 44.9 +/- 12.7 years) with BMI > or = 35 kg/m^2^. N=142 subjects with type 2 diabetes. |  |  | **Overestimation**  Nichols (2020) - <25 BMI Males, recreational exercisers, 6%, (P 64%) | Nichols (2020) - <25 BMI Females, recreational exercisers, (P 61%)  Nichols (2020) - >25 BMI Females, recreational exercisers, (P 55%)  Nichols (2020) - >25 BMI Males, recreational exercisers, (P 64%) |
| Huang (2004)  (FFM, FM, age) [97] | **Males (kcal/24h):** 14.118 x FFM (kg) + 9.3367 x FM (kg) – 1.515 x Age (yrs) + 742.858  **Females (kcal/24h):** 14.118 x FFM (kg) + 9.3367 x FM (kg) – 1.515 x Age (yrs) + 521.995 | “” |  |  | **Underestimation**  Nichols (2020) - <25 BMI Females, recreational exercisers, 6%. (P 58%) | Nichols (2020) - >25 BMI Females, recreational exercisers, (P 53%)  Nichols (2020) - <25 BMI Males, recreational exercisers, 6%, (P 79%)  Nichols (2020) - >25 BMI Males, recreational exercisers, (P 66%) |
| Müller (MJ/d) (2004)  (age, weight, height) [98] | **Males (MJ/24h):** 0.02606 x Wt (kg) + 0.04129 x Ht (cm) – 0.08369 x Age (yrs) – 0.497  **Females (MJ/24h):** 0.02606 x Wt (kg) + 0.04129 x Ht (cm) – 0.08369 x Age (yrs) – 0.808 | Cross-sectional and retrospective analysis of data on REE and body composition obtained from n=2528 subjects aged 5-91 y between 1985 and 2002 |  |  | **Overestimation**  Nichols (2020) - <25 BMI Males, recreational exercisers, 6%, (P 58%) | Nichols (2020) - <25 BMI Females, recreational exercisers, (P 58%)  Nichols (2020) - >25 BMI Females, recreational exercisers, (P 57%)  Nichols (2020) - >25 BMI Males, recreational exercisers, (P 57%) |
| Müller (MJ/d) (2004)  (FFM, FM) [98] | **Males (MJ/24h):** 0.07885 x FFM (kg) + 0.02132 x FM (kg) + 3.021  **Females (MJ/24h):** 0.07885 x FFM (kg) + 0.02132 x FM (kg) + 2.694 | “” |  |  |  | Nichols (2020) - <25 BMI Females, recreational exercisers, (P 65%)  Nichols (2020) - >25 BMI Females, recreational exercisers, (P 58%)  Nichols (2020) - <25 BMI Males, recreational exercisers, (P 67%)  Nichols (2020) - >25 BMI Males, recreational exercisers, (P 54%) |
| Henry (2005)  (age, weight, height) [20] | **Males 18-30(kcal/24h):** 14.4 x Wt (kg) + 313 x Ht (m) + 113  **Males 30-60 (kcal/24h):** 11.4 x Wt (kg) + 541 x Ht (m) + 137  **Females 18-30(kcal/24h):** 10.4 x Wt (kg) + 615 x Ht (m) -282  **Females 30-60 (kcal/24h):** 8.18 x Wt (kg) + 502 x Ht (m) – 116 | Using data from previous studies. 18-30yrs equation based on n=2816 men; 30-60yrs (n=1006 men), excluding all Italian subjects & including a large number from the tropics |  |  |  | Nichols (2020) - <25 BMI Females, recreational exercisers, (P 61%)  Nichols (2020) - >25 BMI Females, recreational exercisers, (P 55%)  Nichols (2020) - <25 BMI Males, recreational exercisers, (P 67%)  Nichols (2020) - >25 BMI Males, recreational exercisers, (P 66%) |
| Livingston & Kohlstadt (2005) (age, weight) [99] | **Males (kcal/24h):** 293 x Wt^0..4330^ (kg) – 5.92 x Age (yrs)  **Females (kcal/24h):** 248 x Wt^0..4356^ (kg) – 5.09 x Age (yrs) | This equation was derived using data from the original Harris-Benedict and Owen databases, and n=327 male and female patients enrolled in a weight management program. |  |  |  | Nichols (2020) - <25 BMI Females, recreational exercisers, (P 58%)  Nichols (2020) - >25 BMI Females, recreational exercisers, (P 57%)  Nichols (2020) - <25 BMI Males, recreational exercisers, (P 61%)  Nichols (2020) - >25 BMI Males, recreational exercisers, (P 59%) |
| Johnstone (2006)  (FFM, FM, age) [100] | **Unisex (kJ/24h):** 90.2 x FFM (kg) + 31.6 x FM (kg) – 12.2 x Age (yrs) + 1613 | Cross-sectional study of 150 Caucasian adults (males n=43, females n=107) from Scotland, with a body mass index range of 16.7-49.3 kg/m^2^. |  | **Underestimation**  Balci (2021) – Female, members of the Turkish National Olympic Team, 10%, (P 54%) |  | Balci (2021) – Males, members of the Turkish National Olympic Team (P 52%)  Nichols (2020) - <25 BMI Females, recreational exercisers, (P 58%)  Nichols (2020) - >25 BMI Females, recreational exercisers, (P 58%)  Nichols (2020) - <25 BMI Males, recreational exercisers, (P 79%)  Nichols (2020) - >25 BMI Males, recreational exercisers, (P 68%) |
| Korth (2007)  (age, weight, height) [101] | **Males (kJ/24h):** 41.5 x Wt (kg) + 35.0 x Ht (cm) – 19.1 x Age (yrs) – 623.8  **Females (kJ/24h):** 41.5 x Wt (kg) + 35.0 x Ht (cm) – 19.1 x Age (yrs) – 1731.2 | N=50 men (age 37.1 ± 15.1 years, body mass index (BMI) 25.9 ± 4.1 kg/m^2^) and n=54  women (age 35.3 ± 15.4 years, BMI 25.5 ± 4.4 kg/m^2^) |  | **Overestimation**  Nichols (2020) - <25 BMI Males, recreational exercisers, 18%, (P 21%)  Nichols (2020) - >25 BMI Males, recreational exercisers, 10%, (P 41%) | **Overestimation**  Nichols (2020) - <25 BMI Females, recreational exercisers, 9%, (P 55%) | Nichols (2020) - >25 BMI Females, recreational exercisers, (P 61%) |
| Korth (2007)  (FFM) [101] | **Unisex (kJ/24h):** 105.5 x FFM (kg) + 1388 | “” |  |  | **Overestimation**  Nichols (2020) - <25 BMI Males, recreational exercisers, 9%, (P 46%)  Nichols (2020) - >25 BMI Males, recreational exercisers, 5%, (P 48%) | Nichols (2020) - <25 BMI Females, recreational exercisers, (P 58%)  Nichols (2020) - >25 BMI Females, recreational exercisers, (P 25%) |
| Lazzer (2007)  (age, weight, height) [102] | **Unisex (MJ/24h):** Wt (kg) x 0.048 + Ht (m) x 4.655 – Age x 0.020 – 3.605 | N=164 severely obese males aged  20 to 65 yrs (mean body mass  index (BMI): 45.4 kg/m2; 50.2% fat mass) |  | **Underestimation**  Nichols (2020) - <25 BMI Males, recreational exercisers, 14%, (P 33%)  Nichols (2020) - >25 BMI Males, recreational exercisers, 15% (P 30%) | **Underestimation**  Nichols (2020) - <25 BMI Females, recreational exercisers, 9%, (P 42%)  Nichols (2020) - >25 BMI Females, recreational exercisers, 7%, (P 48%) |  |
| Lazzer (2007)  (FFM) [102] | **Unisex (MJ/24h):** FFM (kg) x 0.081 + FM (kg) x 0.049 – Age x 0.019 – 2.194 | “” |  |  | **Overestimation**  Nichols (2020) - <25 BMI Males, recreational exercisers, 7%, (P 15%)  Nichols (2020) - >25 BMI Males, recreational exercisers, 10%, (P 39%)  **Underestimation**  Nichols (2020) - <25 BMI Females, recreational exercisers, 7%, (P 58%)  Nichols (2020) - >25 BMI Females, recreational exercisers, 6%, (P 53%) |  |
| Horie & Waitzberg (2011) (FFM, weight) [103] | **Unisex (kcal/24h):** 560.43 + (5.39 × Wt kg) + (14.14 × FFM (kg) | 120 severely obese patients were evaluated (n=37 males and n=83 women), with BMI ranged from 34.4 to 61.0 kg/m^2^ and age ranged from 18 to 62 years |  | **Overestimation**  Nichols (2020) - <25 BMI Females, recreational exercisers, 11%, (P 42%)  Nichols (2020) - >25 BMI Females, recreational exercisers, 9%, (P 51%) | **Overestimation**  Nichols (2020) - <25 BMI Males, recreational exercisers, 8%, (P 52%)  Nichols (2020) - >25 BMI Males, recreational exercisers, 5%, (P 55%) |  |
| Sabounchi (2013)  (FFM, FM) [104] | **Males (kcal/24h):** 361 + (21.1 x FFM (kg)) + (4.77 x FM (kg))  **Females (kcal/24h):** 360 + (21 x FFM (kg)) + (4.68 x FM (kg)) | Using a subset of 47 studies that developed their own RMR prediction equations, meta-regression equations were developed |  | **Underestimation**  Devrim-Lampir (2017) – Male and female, Ultra-endurance, males: 24%, females: 15%, (Males: P 20%, Females: P 27%) |  |  |
| Sabounchi (2013)  (FFM) [104] | **Males (kcal/24h):** 503 + (18.3 x FFM (kg))  **Females (kcal/24h):** 473 + (20.1 x FFM (kg)) | “” |  | **Underestimation**  Devrim-Lampir (2017) – Male and female, Ultra-endurance, males: 23%, females: 19%, (Males: P 27%, Females: P 13%) |  |  |
| Sabounchi (2013)  (age, FFM, FM) [104] | **Males (kcal/24h):** 898 + (14.3 x FFM (kg)) + (6.46 x FM (kg)) - (3.32 x Age (yrs))  **Females (kcal/24h):** 682 + (12.9 x FFM (kg)) + (5.9 x FM (kg)) - (3.08 x Age (yrs)) | “” |  | **Underestimation**  Devrim-Lampir (2017) – Male and female, Ultra-endurance, males: 35%, females: 15%, (Males: P 7%, Females: P 20%) |  |  |

| **Equations Developed in Athlete Populations** | | | | |  |  |
| --- | --- | --- | --- | --- | --- | --- |
| **Equation Name (year) (components), country** | **RMR Equation** | **Population equation based on** | | **Bias > 10% (% bias)** | **Bias <10% (precision (P) % where reported)** | **Bias <5% (precision (P) % where reported)** |
|  |  | **Number (male (M), female (F)), age** | **Other characteristics described** |  |  |  |
| De Lorenzo (1999)  (weight, height), Italy [19] | **Males (kcal/24h):** (9 x wt (kg)) + (11.7 x ht (cm)) - 857 | n=51 M, 22.3 ± 3.5 yrs | Athletes exercising ≥3 h/d, 6d/wk in water polo, judo or karate. Wt, 78.0±11.5 kg; ht, 178.4±7.1 cm; body fat 12.4±4.1% | **Overestimation**  Balci (2021) – Female, members of the Turkish National Olympic Team, 17%, (P 42%)  **Underestimation**  Jagim (2019) - Males, NCAA Div III mixed sports, 12%, (P NR%)  Tinsley (2019) - Male, Bodybuilders, 13%, (P NR%) | **Overestimation** Jagim (2019) – Females, NCAA Div III mixed sports, 7%, (P NR%)  Tinsley (2019) – Female, Bodybuilders, 7%, (P NR%)  Ten-Haaf (2014) – Females, Mix of athletics, endurance, and team sports, 7%, (P 59%)  Nichols (2020) - <25 BMI Females, recreational exercisers, 7%, (P 52%)  Nichols (2020) - >25 BMI Females, recreational exercisers, 6%, (P 57%)  Nichols (2020) - <25 BMI Males, recreational exercisers, 8%, (P 46%) | Balci (2021) – Males, members of the Turkish National Olympic Team (P 40%)  Marra (2021) – Males, mix of endurance, combat, and water polo, (P 75%)  Ten-Haaf (2014) – Males, Mix of athletics, endurance, and team sports, (P 77%)  Wong (2012) – Males, Mix of combat, team, and skilled sports, (P NR%)  Nichols (2020) - >25 BMI Males, recreational exercisers, (P 57%) |
| Taguchi (2011), Japan  (weight) [76] | **Females (kcal/24h):** 26.9 x FFM (kg) + 36 | N=93 F; 20.3 ± 1.2 yrs | N=93 Collegiate female athletes competing in Japan in inter-collegiate games from various sports including track and field, swimming, lacrosse, basketball, judo, rhythmic gymnastics, rowing, cheerleading, badminton, weightlifting. Wt, 57.0 ± 9.2 kg, ht, 162.8 ± 6.4 cm, body fat 20.0 ± 3.9 |  | **Underestimation** Watson (2019) – Females, NCAA Div II mixed sports, 7%, (P NR%) |  |
| Wong (2012)  (weight), Malaysia [61] | **Unisex (kcal/24h):**  669 + (13 x Wt (kg)) + (192 x sex (M=1, F=0)) | n=92 M, n=33 F; 18-31yrs | ‘Elite’ National Malaysian athletes from 15 sports (combat, racquet, team, skilled, other) training ≥ 6h/d for at least one year. M: wt, 66.1± 8.5 kg; ht, 170.6 ±6.5 cm; body fat 13.7 ±4.0%, F: wt, 55.4±5.7 kg; ht, 160.7±4.8 cm; body fat, 21.7±4.0 % |  |  | Wong (2012) – Males & Females, Mix of combat, team, and skilled sports, (P NR%)  Marra (2021) – Males, Mix of endurance, combat, and water polo, (P 75%) |
| Ten-Haaf (2014) (age, weight, height), The Netherlands [21] | **Unisex (kcal/24h):** (11.936 x Wt (kg)) + (587.728 x Ht (m)) -  (8.129 x age (yrs)) + (191.027 x sex (M=1, F=0)) + 29.279 | n=53 M, n=37 F; 18-35yrs | Dutch recreationally active adults (training 9.1 ± 5.0 h/wk, frequency, 5.0 ± 1.8 times/wk) from ~ 16 sports including athletics, cycling, gymnastics, fitness, rowing/canoeing, team sports, other). All: wt, 52.8-100.3 kg; ht, 161-205cm, body fat, 1.5-32.2 %. F: wt, 52.8-78.3 kg; ht, 161-184cm, body fat, 11.2-32.2 %. M: wt, 62.1-100.3 kg; ht, 163-205 cm, body fat, 1.5 – 26.3 %. |  | **Underestimation**  Tinsley (2019) - Male, Bodybuilders, 6%, (P NR%) | O’Neill (2022) - Female, Rugby, (P 83%)  Marra (2021) – Males, Mix of endurance, combat, and water polo, (P 75%)  Tinsley (2019) – Female, Bodybuilders, (P NR%)  Ten-Haaf (2014) – Males and females, Mix of athletics, endurance, and team sports, (Males: P 85%, Females: P 76%)  Van Hooren (2022) – Male and female, Professional cyclists (P NR%)**    Freire, (2023) – Male and female, Brazillian National & Olympic Team, (Males: P 72%, Females: P 66%))** |
| Ten-Haaf (2014) (FFM), The Netherlands [21] | **Unisex (kcal/24h):** (22.771 x FFM (kg)) + 484.264 | “” | “”; FFM by air displacement plethysmography (BodPod). |  |  | O’Neill (2022) - Female, Rugby, (P 86%)  Tinsley (2019) – Male and female, Bodybuilders, (P NR%) |
| Koehler DXA-predicted (2016) (brain mass, skeletal muscle mass, adipose tissue mass, bone mass, residual mass), USA, Canada [58] | **Unisex (kcal/24h):** (240 x brain mass (kg)) + (13 x skeletal muscle mass (kg)) + (2.3 x bone mass (kg)) + (4.5 x adipose tissue mass (kg)) + (43 x residual mass (kg)) | n=79 F, 18-35 yrs | All exercising (≥2h/wk), primarily (72%) aerobic (running, cycling, cardio training, swimming, triathlon, aerobics), others in team sports and resistance exercise. n=42 with amenorrhea (wt, 58.4±1.0kg; ht, 168.0±1.0cm; body fat, 21.5±0.9%; N=37 with ovulatory menstrual cycles (wt, 58.2±0.8 kg, ht, 164.0±1.0cm, body fat, 24.1±0.8%). Modelling of organ tissues based on DXA. | **Overestimation**  Staal (2018) – Female, Ballet, 13%, (P 65%)  Strock A (2020) – Females, Recreational exercisers, amenorrheic group: 11%, (P NR%) | **Overestimation**  Staal (2018) – Female, Ballet, 7%, (P 45%)  Strock A (2020) – Females, Recreational exercisers, sub-clinical menstrual dysfunction: 6% (P NR%)  Koehler (2016) – Female recreational exercisers, amenorrheic group, 9%, (P NR%) | Strock A (2020) – Females, Recreational exercisers, ovulatory group, (P NR%)  Strock B (2020) – Female, recreational exercisers, (P NR%)  Koehler (2016) – Female recreational exercisers, ovulatory group, (P NR%) |
| Joseph (2017) (weight), India [28] | **Males (kcal/24h):** -164.065 + (0.039 x LBM (kg)) | n=30 M; 18-28yrs | Indian international competitive weightlifters training ≥4h/d ≥5/d/wk, wt, 56.6-127.6 kg; ht 157.5-182.0 cm, body fat 8.4-30.3%. LBM determined by DXA. |  |  | Joseph (2017) - Male, Indian weightlifters, (P NR%) |
| Jagim (2019) (weight), USA [22] | **Males (kcal/24h):** 19.46 x Wt (kg) + 775.33  **Females (kcal/24h):** 21.10 x Wt (kg) + 288.6 | n=68 M, n=48 F; M: 20.1 ± 1.5 yrs, F: 19.4 ± 1.3 yrs | NCAA Division III athletes (M: football, track (distance), baseball; F: track (sprint, distance, throw), swimming, soccer, tennis). M: wt, 93.7 ± 16.3 kg; ht, 181.8 ± 5.9 cm; body  fat , 16.3 ± 8.6%. F: wt, 63.4 ± 12.7 kg, ht, 166.5 ± 6.0 cm; body fat, 21.5 ± 6.3% | **Overestimation**  O’Neill (2022) - Female, Rugby, 10%, (P 44%) |  | Jagim (2019) – Females and males, NCAA Div III, (P NR%) |
| Tinsley (2019)  (FFM), USA [23] | **Unisex (kcal/24h):** 25.9 x FFM (kg) + 284 | n=17 M, n=10 F, 25.9±6.0yrs | Subjects who self-identified as bodybuilders, training 5.7±0.9 d/wk. All: wt, 82.9± 17.0kg; ht, 175.6±9.2cm; body fat, 15.0± 4.4%. M: wt, 94.0± 9.7kg; ht, 180.4±7.2cm; body fat, 12.5±2.7 %. F: wt, 63.8±5.7; ht, 167.5± 5.7cm; body fat, 19.2 ± 3.4%. FFM determined by DXA. Note 26% reported anabolic steroid use. |  |  | Tinsley (2019) – Male and female, Bodybuilders, (P NR%). *Leave one out cross-validation performed |
| Tinsley (2019)  (weight), USA [23] | **Unisex (kcal/24h):** 24.8 x Wt (kg) + 10 | “” |  |  |  | Tinsley (2019) – Male and female, Bodybuilders, (P NR%) *Leave one out cross-validation performed in same study |
| Watson (2019)  (age, weight, height), USA [25] | **Females (kcal/24h):** 88.1 + (2.53 x Ht (cm)) + (8.42 x Wt (kg)) + (19.46 x Age (yrs)) | n=66 F, 19.7 ± 1.1 yrs | NCAA Division II athletes (ice hockey, volleyball, basketball, softball, golf, field hockey, gymnastics, tennis, other). Wt, 67.3 ± 8.9 kg; ht, 169 ± 9 cm; body fat, 26.2 ± 4.9 % | Watson (2019) - Cross-validated (paired t-test only, bias not reported) in sub-sample in same study (n=22), female rugby players |  | O’Neill (2022) - Female, Rugby, (P 67%) *Leave one out cross-validation performed in same study |
| Watson (2019)  (age, FFM, height), USA [25] | **Females (kcal/24h):** 120.81 + (4.88 x Ht (cm)) + (8.24 x FFM (kg)) + (5.71 x Age (yrs)) | “” | “”. FFM determined by 3-site skinfolds. | Watson (2019) - Cross-validated (paired t-test only, bias not reported) in sub-sample in same study (n=22), female rugby players | **Underestimation**  O’Neill (2022) - Female, Rugby, 8%, (P 53%) |  |
| MacKenzie-Shalders (2020) (LBM, FM), Australia [60] | **Males (kcal/24h):** 25.49 x LBM (kg) + 7.62 x FM (kg) + 162.93 | n=18 M, 20.2 ± 1.7 yrs | Developing elite rugby union athletes. Wt, 101.2 ± 14.5 kg; ht, 184.0 ± 8.4 cm, LBM, 81.3 ± 8.0 kg. LBM, FM by DXA. |  |  | Mackenzie-Shalders (2020) – Male, Elite Rugby, (P NR%) |
| MacKenzie-Shalders (2020) (LBM), Australia [60] | **Males (kcal/24h):** 29.71 x LBM (kg) – 24.562 | “” | “” |  |  | Mackenzie-Shalders (2020) – Male, Elite Rugby, 6%, (P NR%) |
| MacKenzie-Shalders (2020) (weight) [60] | **Males (kcal/24h):** 15.95 x Wt (kg) + 775.32 | “” |  |  |  | Mackenzie-Shalders (2020) – Male, Elite Rugby, 6%, (P NR%) |
| O’Neill (2022)  (age, weight, height), Ireland [9] | **Females (kcal/24h):** 1501 – (6.858 x Age (yrs)) – (2.946 x Ht (cm)) + (11.21 x Wt (kg)) | n= 36 F, 18–35 yrs | Elite and sub-elite rugby players. Wt, 59.4-99.9 kg; ht, 157.0-182.2cm; FFM, 43 –63 kg; body fat, 15–41 %. |  |  | O’Neill (2022) - Female, Rugby, (P 83%) |
| O’Neill (2022)  (FFM), Ireland [9] | **Females (kcal/24h):** 18.91 x FFM (kg) + 649.6 | “” | “”, FFM by air displacement plethysmography |  |  | O’Neill (2022) - Female, Rugby, (P 83%) |
| Marra (2021)  (age, weight), Italy [33] | **Males (kcal/24h):** (17.2 x Wt (kg)) – (5.95 x Age (yrs)) + 748 | n=75 M, 26.9 ± 9.1 yrs | Elite athletes (previously competed at regional/national level), training ≥24h/wk swimming, cycling, running, karate, water polo, ballet, boxing. Wt, 71.3 ± 10.9 kg; Ht, 177 ± 7 cm |  |  | Marra (2021) – Males, Mix of endurance, combat, and water polo, (P 82%) *Cross-validated in same study, similar population (n=51) |
| Marra (2021)  (weight, BIA-derived phase angle), Italy [33] | **Males (kcal/24h):** (16.3 x Wt (kg)) + (95.4 x PhA (degrees)) - 93 | “” |  |  |  | Marra (2021) – Males, Mix of endurance, combat, and water polo, (P 92%) *Cross-validated in same study, similar population (n=51) |
| **Equations since review search completed** | | | | |  |  |
| Freire (2022) (sex, age, weight, height), Brazil [74] | **Unisex (kcal/24h):**  729.50 + (175.84 x sex (M = 0, F = 1)) – (7.23 x age (yrs)) + (15.87 x wt (kg)) + (1.08 x ht (cm)) | n=34 M, n=37 F; 24.5-26.4yrs | ‘High level’ athletes (majority world championship and/or Olympic) from 21 sports including endurance, team, racquet, combat, skilled sports, weightlifting and wrestling; wt, 72.9-82.2kg; ht, 173.6-178.7cm |  |  | Freire, (2023) – Male and females, Brazillian National & Olympic Team, (P 61%) * Cross-validated in same study, similar population (n=31) |
| Freire (2022) (sex, age, weight, height), Brazil [74] | **Unisex (kcal/24h):**  -2688.12 + (521.08 x sex (M = 0, F = 1)) + (42.86 x age (yrs)) + (18.98 x wt (kg)) + (16.76 x ht (cm)) + (85.47 x Meso) + (140.54 x Endo) - (8.24 x (wt (kg) x sex (M = 0, F = 1))) + (1.53 x (wt (kg) x Endo)) – (0.65 (wt (kg) x age (yrs)) | “” | “” |  |  | Freire, (2023) – Male and females, Brazillian National & Olympic Team, (P 61%) * Cross-validated in same study, similar population (n=31) |
| Van Hooren, (2023) (sex, weight), Netherlands [75] | **Unisex (MJ/24h):**  0.963 - (0.186 x sex (M = 0, F = 1)) + (0.106 x wt (kg))  **Males only (MJ/24h):**  0.767 + (0.106 x wt (kg)) | n=21 M, n = 4 F; 27.0 ± 4.0 yrs | Professional cyclists from UCI World Tour Team, Wt, 66.8 ± 7.5 kg; Ht, 180.6 ± 6.8 cm |  |  | Van Hooren (2023) - Male professional cyclists, (P NR%)** |

*BMI body mass index, BIA bioelectrical impedance, Endo endomorphy, F female, FFM fat-free mass, FM fat mass, ht height, LBM lean body mass, Meso mesomorph, M male, N/a not applicable, NR not reported, P precision, Pha phase angle, RMR resting metabolic rate, wt weight. ** Study published since search completed, not included in narrative synthesis of systematic review or meta-analysis*

**Title of supplementary material:** Supplementary Document 8 – Table: Accuracy Meta-Analysis Results for all included equations

| **Equation** | **Comparisons** | **Studies** | **Participants** | **Standardised Mean Difference [95% CI]** | **Chi^2^**  **(P-value)** | **I^2^** | **Test for Overall Effect (Z) (P-value)** |
| --- | --- | --- | --- | --- | --- | --- | --- |
| Cunningham (1980) (FFM) | 30 | 18 | 846 | 0.15 [-0.26, 0.57] | 443.95 (P<0.0001) | 93% | Z=0.72 (P=0.47) |
| Harris-Benedict (1918) (age, weight. Height) | 29 | 17 | 892 | -0.14 [-0.52, 0.25] | 400.76 (P<0.00001) | 93% | Z = 0.69 (P = 0.49) |
| Cunningham (1991) (FFM) | 8 | 4 | 307 | 0.31 [-0.09, 0.70] | 35.54 (P<0.00001) | 80% | Z = 1.51 (P = 0.13) |
| Mifflin St. Jeor (1990) (age, weight, height) | 15 | 9 | 418 | -0.77 [-1.16, -0.39] | 92.25 (P < 0.00001) | 85% | Z = 3.93 (P < 0.0001) |
| Owen (1988) (weight) | 6 | 4 | 256 | -1.49 [-2.16, -0.81] | 54.09 (P <0.00001) | 91% | Z = 4.32 (P < 0.0001) |
| De Lorenzo (1999) (age, weight, height) | 10 | 6 | 359 | 0.15 [-0.27, 0.56] | 63.21 (P < 0.00001) | 86% | Z = 0.68 (P = 0.49) |
| Koehler DXA (2016) | 8 | 4 | 350 | 0.62 [0.25, 0.98] | 35.10 (P < 0.0001) | 80% | Z = 3.34 (P = 0.0008) |
| FAO.WHO.UNU (1985) (age, weight, height) | 8 | 5 | 204 | -0.88 [-1.43, -0.33] | 40.78 (P < 0.00001) | 83% | Z = 3.15 (P = 0.002) |
| FAO.WHO.UNU (1985) (age, weight) | 11 | 7 | 375 | -0.87 [-1.27, -0.48] | 59.73 (P < 0.00001) | 83% | Z = 4.31 (P < 0.0001) |
| Ten-Haaf (2014) (age, weight, height) | 7 | 4 | 204 | 0.04 [-0.16, 0.23] | 5.50 (P = 0.48) | 0% | Z = 0.38 (P = 0.70) |
| Nelson (1992) (FFM, FM) | 5 | 3 | 129 | -1.10 [-1.81, -0.39] | 27.51 (P < 0.0001) | 85% | Z = 3.05 (P = 0.002) |

*FFM* fat fee mass, *FM* fat mass.

**Title of supplementary material:** Supplementary Document 9 – Results for all subgroup analysis for all included equations

**Table a: Meta-Analysis Subgroup Results - Sex**

| **Equation** | **Subgroups** | **#**  **Comparisons** | **#**  **Studies** | **#**  **Participants** | **Standardised Mean Difference [95% CI]** | **Chi^2^**  **(P-value)** | **I^2^**  **(Within**  **Subgroup)** | **Test for Overall Effect (Z) (P-value)** | **Test for Subgroup Differences (Chi^2^) (P-value)** | **I^2^**  **(Between**  **Subgroups)** |
| --- | --- | --- | --- | --- | --- | --- | --- | --- | --- | --- |
| Cunningham (1980) (FFM) | Males only | 11 | 11 | 307 | -0.07 [-0.75, 0.60] | 134.96 (P < 0.00001) | 93% | Z = 0.22 (P = 0.83) | 0.81 (P = 0.37) | 0% |
|  | Females only | 19 | 14 | 539 | 0.29 [-0.14, 0.72] | 184.80 (P < 0.00001) | 90% | Z = 1.33 (P = 0.18) |  |  |
| Harris-Benedict (1918) (age, weight. Height) | Males only | 11 | 11 | 357 | -0.53 [-0.93, -0.13] | 60.66 (P < 0.00001) | 84% | Z = 2.58 (P = 0.010) | 3.66 (P = 0.06) | 72.7% |
|  | Females only | 18 | 14 | 535 | 0.11 [-0.41, 0.63] | 248.41 (P < 0.00001) | 93% | Z = 0.42 (P = 0.68) |  |  |
| Mifflin St. Jeor (1990) (age, weight, height) | Males only | 7 | 7 | 219 | -0.99 [-1.43, -0.56] | 26.07 (P = 0.0002) | 77% | Z = 4.46 (P < 0.00001) | 1.25 (P = 0.26) | 20% |
|  | Females only | 8 | 8 | 199 | -0.57 [-1.17, 0.03] | 51.19 (P < 0.00001) | 86% | Z = 0.42 (P = 0.06) |  |  |
| Owen (1988) (weight) | Males only | 3 | 3 | 129 | -1.42 [-2.25, -0.60] | 16.65 (P = 0.0002) | 88% | Z = 3.39 (P = 0.0007) | 0.03 (P = 0.86) | 0% |
|  | Females only | 3 | 3 | 127 | -1.57 [-2.90, -0.24] | 37.36 (P < 0.00001) | 95% | Z = 2.31 (P = 0.02) |  |  |
| De Lorenzo (1999) (age, weight, height) | Males only | 6 | 6 | 266 | -0.31 [-0.73, 0.11] | 26.23 (P < 0.0001) | 81% | Z = 1.43 (P = 0.15) | 21.86 (P < 0.00001) | 95.4% |
|  | Females only | 4 | 4 | 93 | 0.93 [0.63, 1.23] | 0.56 (P = 0.91) | 0% | Z = 5.99 (P < 0.00001) |  |  |
| FAO.WHO.UNU (1985) (age, weight, height) | Males only | 3 | 3 | 119 | -0.95 [-1.51, -0.39] | 7.44 (P = 0.02) | 73% | Z = 3.32 (P = 0.0009) | 0.05 (P = 0.82) | 0% |
|  | Females only | 4 | 4 | 77 | -0.79 [-2.07, 0.49] | 33.13 (P < 0.00001) | 91% | Z = 1.21 (P = 0.23) |  |  |
| FAO.WHO.UNU (1985) (age, weight) | Males only | 5 | 5 | 207 | -0.85 [-1.35, -0.34] | 20.88 (P = 0.0003) | 81% | Z = 3.25 (P = 0.001) | 0.03 (P = 0.86) | 0% |
|  | Females only | 6 | 6 | 168 | -0.93 [-1.62, -0.23] | 38.46 (P < 0.00001) | 87% | Z = 2.61 (P = 0.009) |  |  |
| Ten-Haaf (2014) (age, weight, height) | Males only | 3 | 3 | 121 | -0.03 [-0.45, 0.39] | 4.92 (P = 0.09) | 59% | Z = 0.14 (P = 0.89) | 0.09 (P = 0.76) | 0% |
|  | Females only | 4 | 3 | 83 | 0.05 [-0.25, 0.35] | 0.56 (P = 0.90) | 0% | Z = 1.75 (P = 0.08) |  |  |

**Table b: Meta-Analysis Subgroup Results – Body Composition Measurement Method**

| **Equation** | **Subgroups** | **#**  **Comparisons** | **#**  **Studies** | **#**  **Participants** | **Standardised Mean Difference [95% CI]** | **Chi^2^**  **(P-value)** | **I^2^**  **(Within**  **Subgroup)** | **Test for Overall Effect (Z) (P-value)** | **Test for Subgroup Differences (Chi^2^) (P-value)** | **I^2^**  **(Between**  **Subgroups)** |
| --- | --- | --- | --- | --- | --- | --- | --- | --- | --- | --- |
| Cunningham (1980) (FFM) | DXA only | 16 | 10 | 425 | 0.52 [-0.08, 1.12] | 218.04 (P<0.0001) | 93% | Z=1.69 (P=0.09) | 5.37 (P=0.07) | 62.8% |
|  | BIA | 5 | 3 | 162 | -0.70 [-1.71, 0.31] | 56.16 (P<0.0001) | 93% | Z=1.36 (P=0.17) |  |  |
|  | BODPOD only | 6 | 3 | 176 | -0.13 [-0.39, 0.13] | 7.38 (P=0.19) | 32% | Z=0.98 (P=0.33) |  |  |

**Table c: Meta-Analysis Subgroup Results - LEA**

| **Equation** | **Subgroups** | **#**  **Comparisons** | **#**  **Studies** | **#**  **Participants** | **Standardised Mean Difference [95% CI]** | **Chi^2^**  **(P-value)** | **I^2^**  **(Within**  **Subgroup)** | **Test for Overall Effect (Z) (P-value)** | **Test for Subgroup Differences (Chi^2^) (P-value)** | **I^2^**  **(Between**  **Subgroups)** |
| --- | --- | --- | --- | --- | --- | --- | --- | --- | --- | --- |
| Cunningham (1980) (FFM) | LEA | 6 | 4 | 199 | 0.27 [-0.63, 1.17] | 72.63 (P < 0.00001) | 93% | Z = 0.58 (P = 0.56) | 0.65 (P = 0.42) | 0% |
|  | Non-LEA | 19 | 14 | 560 | -0.15 [-0.62, 0.32] | 234.28 (P < 0.00001) | 92% | Z = 0.63 (P = 0.53) |  |  |

**Table d: Meta-Analysis Subgroup Results – Athlete Status**

| **Equation** | **Subgroups** | **#**  **Comparisons** | **#**  **Studies** | **#**  **Participants** | **Standardised Mean Difference [95% CI]** | **Chi^2^**  **(P-value)** | **I^2^**  **(Within**  **Subgroup)** | **Test for Overall Effect (Z) (P-value)** | **Test for Subgroup Differences (Chi^2^) (P-value)** | **I^2^**  **(Between**  **Subgroups)** |
| --- | --- | --- | --- | --- | --- | --- | --- | --- | --- | --- |
| Cunningham (1980) (FFM) | Tier 3: Highly Trained/National Level | 20 | 12 | 446 | -0.01 [-0.57, 0.55] | 265.20 (P<0.0001) | 93% | Z=0.04 (P=0.97) | 0.03 (P = 0.87) | 0% |
|  | Tier 4: Elite/International Level | 4 | 3 | 79 | -0.08 [-0.76, 0.59] | 12.83 (P=0.005) | 77% | Z=0.24 (P=0.81) |  |  |
| Harris-Benedict (1918) (age, weight. Height) | Tier 1: Recreationally Active | 5 | 3 | 243 | 1.50 [1.26, 1.73] | 4.98 (P = 0.29) | 20% | Z = 12.44 (P < 0.00001) | 83.38  (P < 0.00001) | 97.6% |
|  | Tier 3: Highly Trained/National Level | 17 | 11 | 444 | -0.42 [-0.82, -0.02] | 116.36 (P < 0.00001) | 86% | Z = 2.08 (P = 0.04) |  |  |
|  | Tier 4: Elite/International Level | 5 | 3 | 115 | -0.38 [-1.00, 0.25] | 21.32 (P = 0.0003) | 81% | Z = 1.19 (P = 0.24) |  |  |

**Table e: Meta-Analysis Subgroup Results - Average Mass >/< 62.7kg Females**

| **Equation** | **Subgroups** | **#**  **Comparisons** | **#**  **Studies** | **#**  **Participants** | **Standardised Mean Difference [95% CI]** | **Chi^2^**  **(P-value)** | **I^2^**  **(Within**  **Subgroup)** | **Test for Overall Effect (Z) (P-value)** | **Test for Subgroup Differences (Chi^2^) (P-value)** | **I^2^**  **(Between**  **Subgroups)** |
| --- | --- | --- | --- | --- | --- | --- | --- | --- | --- | --- |
| Cunningham (1980) (FFM) | >62.7kg | 7 | 6 | 156 | 0.45 [0.19, 0.71] | 7.16 (P = 0.31) | 16% | Z = 3.37 (P = 0.0008) | 0.41 (P = 0.52) | 0% |
|  | <62.7kg | 12 | 8 | 383 | 0.22 [-0.44, 0.87] | 174.64 (P < 0.00001) | 94% | Z = 0.65 (P = 0.52) |  |  |
| Harris-Benedict (1918) (age, weight. Height) | >62.7kg | 7 | 6 | 155 | -0.10 [-0.70, 0.51] | 34.54 (P < 0.00001) | 83% | Z = 0.31 (P = 0.76) | 0.46 (P = 0.50) | 0% |
|  | <62.7kg | 11 | 9 | 380 | 0.23 [-0.49, 0.94] | 188.59 (P < 0.00001) | 95% | Z = 0.63 (P = 0.53) |  |  |
| Mifflin St. Jeor (1990) (age, weight, height) | >62.7kg | 4 | 4 | 110 | -0.31 [-1.18, 0.57] | 21.49 (P < 0.0001) | 86% | Z = 0.69 (P = 0.49) | 0.68 (P = 0.41) | 0% |
|  | <62.7kg | 4 | 4 | 89 | -0.83 [-1.72, 0.05] | 21.81 (P < 0.0001) | 86% | Z = 1.84 (P = 0.07) |  |  |

**Table f: Meta-Analysis Subgroup Results - Average Mass >/< 78.9kg Males**

| **Equation** | **Subgroups** | **#**  **Comparisons** | **#**  **Studies** | **#**  **Participants** | **Standardised Mean Difference [95% CI]** | **Chi^2^**  **(P-value)** | **I^2^**  **(Within**  **Subgroup)** | **Test for Overall Effect (Z) (P-value)** | **Test for Subgroup Differences (Chi^2^) (P-value)** | **I^2^**  **(Between**  **Subgroups)** |
| --- | --- | --- | --- | --- | --- | --- | --- | --- | --- | --- |
| Cunningham (1980) (FFM) | >78.9kg | 5 | 5 | 77 | -0.25 [-0.96, 0.47] | 15.40 (P = 0.004) | 74% | Z = 0.67 (P = 0.50) | 0.11 (P = 0.74) | 0% |
|  | <78.9kg | 6 | 6 | 230 | -0.03 [-1.07, 1.01] | 118.98 (P < 0.00001) | 96% | Z = 0.06 (P = 0.95) |  |  |
| Harris-Benedict (1918) (age, weight. Height) | >78.9kg | 4 | 4 | 71 | -0.91 [-1.25, -0.56] | 1.53 (P = 0.68) | 0% | Z = 5.09 (P < 0.00001) | 3.21 (P = 0.07), | 68.8% |
|  | <78.9kg | 7 | 7 | 286 | -0.33 [-0.85, 0.20] | 51.57 (P < 0.00001) | 88% | Z = 1.21 (P = 0.22) |  |  |

**Table g: Meta-Analysis Subgroup Results – 24hr Physical Activity Abstinence**

| **Equation** | **Subgroups** | **#**  **Comparisons** | **#**  **Studies** | **#**  **Participants** | **Standardised Mean Difference [95% CI]** | **Chi^2^**  **(P-value)** | **I^2^**  **(Within**  **Subgroup)** | **Test for Overall Effect (Z) (P-value)** | **Test for Subgroup Differences (Chi^2^) (P-value)** | **I^2^**  **(Between**  **Subgroups)** |
| --- | --- | --- | --- | --- | --- | --- | --- | --- | --- | --- |
| Cunningham (1980) (FFM) | Present | 18 | 9 | 462 | 0.20 [-0.35, 0.76] | 239.87 (P<0.0001) | 93% | Z=0.72 (P=0.47) | 0.12 (P=0.73) | 0% |
|  | Lacking | 12 | 9 | 384 | 0.06 [-0.53, 0.64] | 149.27 (P<0.0001) | 93% | Z=0.20 (P=0.84) |  |  |
| Harris-Benedict (1918) (age, weight. Height) | Present | 20 | 11 | 547 | 0.07 [-0.45, 0.59] | 290.58 (P < 0.00001) | 93% | Z = 0.27 (P = 0.79) | 3.49 (P = 0.06) | 71.3% |
|  | Lacking | 9 | 6 | 345 | -0.57 [-0.99, -0.14] | 52.35 (P < 0.00001) | 85% | Z = 2.62 (P = 0.009) |  |  |
| Mifflin St. Jeor (1990) (age, weight, height) | Present | 9 | 5 | 205 | -0.54 [-1.04, -0.03] | 46.67 (P < 0.00001) | 83% | Z = 2.06 (P = 0.04) | 1.98 (P = 0.16) | 49.5% |
|  | Lacking | 6 | 4 | 213 | -1.12 [-1.75, -0.49] | 41.59 (P < 0.00001) | 88% | Z = 3.48 (P = 0.0005) |  |  |
| De Lorenzo (1999) (age, weight, height) | Present | 5 | 3 | 150 | 0.22 [-0.49, 0.93] | 34.72 (P < 0.00001) | 88% | Z = 0.61 (P = 0.54) | 0.09 (P = 0.76) | 0% |
|  | Lacking | 5 | 3 | 209 | 0.08 [-0.50, 0.65] | 28.20 (P < 0.0001) | 86% | Z = 0.26 (P = 0.80) |  |  |

**Table h: Meta-Analysis Subgroup Results – Discard, SS & Validated Extraction Method**

| **Equation** | **Subgroups** | **#**  **Comparisons** | **#**  **Studies** | **#**  **Participants** | **Standardised Mean Difference [95% CI]** | **Chi^2^**  **(P-value)** | **I^2^**  **(Within**  **Subgroup)** | **Test for Overall Effect (Z) (P-value)** | **Test for Subgroup Differences (Chi^2^) (P-value)** | **I^2^**  **(Between**  **Subgroups)** |
| --- | --- | --- | --- | --- | --- | --- | --- | --- | --- | --- |
| Cunningham (1980) (FFM) | Present | 14 | 8 | 371 | -0.14 [-0.67, 0.39] | 146.09 (P<0.0001) | 91% | Z=0.51 (P=0.61) | 1.84 (P=0.17) | 45.8% |
|  | Lacking | 16 | 10 | 475 | 0.42 [-0.18, 1.01] | 243.97 (P<0.0001) | 94% | Z=1.37 (P=0.17) |  |  |
| Harris-Benedict (1918) (age, weight. Height) | Present | 15 | 8 | 400 | -0.47 [-0.83, -0.12] | 74.26 (P < 0.00001) | 81% | Z = 2.64 (P = 0.008) | 3.57 (P = 0.06) | 72% |
|  | Lacking | 14 | 9 | 492 | 0.25 [-0.41, 0.91] | 283.44 (P < 0.00001) | 95% | Z = 0.73 (P = 0.46) |  |  |
| Mifflin St. Jeor (1990) (age, weight, height) | Present | 9 | 5 | 222 | -0.67 [-1.17, -0.17] | 47.34 (P < 0.00001) | 83% | Z = 2.64 (P = 0.008) | 0.42 (P = 0.52) | 0% |
|  | Lacking | 6 | 4 | 196 | -0.92 [-1.48, -0.36] | 30.77 (P < 0.0001) | 84% | Z = 3.20 (P = 0.001) |  |  |
| FAO.WHO.UNU (1985) (age, weight) | Present | 7 | 4 | 248 | -0.70 [-1.18, -0.22] | 33.14 (P < 0.00001) | 82% | Z = 2.87 (P = 0.004) | 2.62 (P = 0.11) | 61.8% |
|  | Lacking | 4 | 3 | 127 | -1.15 [-1.42, -0.89] | 1.06 (P = 0.79) | 0% | Z = 8.46 (P < 0.00001) |  |  |

**Table i: Meta-Analysis Subgroup Results – Pre-test Rest vs No Rest**

| **Equation** | **Subgroups** | **#**  **Comparisons** | **#**  **Studies** | **#**  **Participants** | **Standardised Mean Difference [95% CI]** | **Chi^2^**  **(P-value)** | **I^2^**  **(Within**  **Subgroup)** | **Test for Overall Effect (Z) (P-value)** | **Test for Subgroup Differences (Chi^2^) (P-value)** | **I^2^**  **(Between**  **Subgroups)** |
| --- | --- | --- | --- | --- | --- | --- | --- | --- | --- | --- |
| Cunningham (1980) (FFM) | Present | 12 | 6 | 503 | 0.15 [-0.54, 0.83] | 273.92 (P < 0.00001) | 96% | Z = 0.42 (P = 0.67) | 0.00 (P = 0.99) | 0% |
|  | Lacking | 18 | 12 | 343 | 0.15 [-0.38, 0.68] | 168.90 (P < 0.00001) | 90% | Z = 0.56 (P = 0.57) |  |  |
| Harris-Benedict (1918) (age, weight. Height) | Present | 12 | 6 | 503 | -0.10 [-0.78, 0.59] | 267.23 (P < 0.00001) | 96% | Z = 0.28 (P = 0.78) | 0.03 (P = 0.87) | 0% |
|  | Lacking | 18 | 11 | 389 | -0.17 [-0.58, 0.25] | 118.12 (P < 0.00001) | 86% | Z = 0.79 (P = 0.43) |  |  |
| Mifflin St. Jeor (1990) (age, weight, height) | Present | 12 | 3 | 147 | -1.07 [-1.50, -0.63] | 13.32 (P = 0.02) | 62% | Z = 4.81 (P < 0.00001) | 1.94 (P = 0.16) | 48.4% |
|  | Lacking | 17 | 6 | 271 | -0.59 [-1.11, -0.07] | 62.85 (P < 0.00001) | 87% | Z = 2.23 (P = 0.03) |  |  |
| De Lorenzo (1999) (age, weight, height) | Present | 5 | 3 | 209 | 0.08 [-0.50, 0.65] | 28.20 (P < 0.0001) | 86% | Z = 2.87 (P = 0.004) | 0.09 (P = 0.76) | 0% |
|  | Lacking | 5 | 3 | 150 | 0.22 [-0.49, 0.93] | 34.72 (P < 0.00001) | 88% | Z = 8.46 (P < 0.00001) |  |  |
| FAO.WHO.UNU (1985) (age, weight) | Present | 8 | 4 | 272 | -0.98 [-1.41, -0.56] | 32.67 (P < 0.0001) | 79% | Z = 4.58 (P < 0.00001) | 0.52 (P = 0.47) | 0% |
|  | Lacking | 3 | 3 | 103 | -0.59 [-1.58, 0.40] | 16.65 (P = 0.0002) | 88% | Z = 1.16 (P = 0.25) |  |  |

**Table j: Meta-Analysis Subgroup Results – Nutritional/Stimulant Status**

| **Equation** | **Subgroups** | **#**  **Comparisons** | **#**  **Studies** | **#**  **Participants** | **Standardised Mean Difference [95% CI]** | **Chi^2^**  **(P-value)** | **I^2^**  **(Within**  **Subgroup)** | **Test for Overall Effect (Z) (P-value)** | **Test for Subgroup Differences (Chi^2^) (P-value)** | **I^2^**  **(Between**  **Subgroups)** |
| --- | --- | --- | --- | --- | --- | --- | --- | --- | --- | --- |
| Cunningham (1980) (FFM) | 3/3 | 7 | 4 | 314 | 0.97 [0.56, 1.37] | 27.79 (P=0.0001) | 78% | Z=4.68 (P<0.0001) | 443.95 (P<0.0001) | 86.1% |
|  | 2/3 | 11 | 6 | 275 | -0.09 [-0.97, 0.78] | 194.09 (P<0.0001) | 95% | Z=0.21 (P=0.83) |  |  |
|  | 1/3 | 7 | 4 | 134 | -0.33 [-0.71, 0.05] | 14.28 (P=0.03) | 58% | Z=1.69 (=0.09) |  |  |
|  | 0/3 | 5 | 3 | 123 | 0.29 [-0.39, 0.97] | 20.34 (P=0.0004) | 80% | Z=0.84 (P=0.40) |  |  |
| Harris-Benedict (1918) (age, weight. Height) | 3/3 | 8 | 5 | 365 | 0.71 [0.07, 1.35] | 102.34 (P < 0.00001) | 93% | Z = 2.16 (P = 0.03) | 24.18  (P < 0.00001) | 91.7% |
|  | 2/3 | 11 | 6 | 296 | 0.01 [-0.51, 0.52] | 83.15 (P < 0.00001) | 88% | Z = 0.03 (P = 0.98) |  |  |
|  | 1/3 | 7 | 4 | 134 | -0.92 [-1.23, -0.61] | 8.85 (P = 0.18) | 32% | Z = 5.76 (P < 0.00001) |  |  |

**Title of supplementary material:** Supplementary Document 10 – Fullmer Best Practise Guidelines Checklist & Results

| Author | Title | Hyperlink to study | No Vig. Phys. Act. ≥24hrs? | Fasted ≥7hrs? | No caffeine/stims ≥4hrs? | No nicotine ≥2.5hrs? | Rested quietly ≥30mins? | If not 30… ≥20mins? | Supine? | Temp. 22C - 25C? | Quiet room & lights dimmed? | Discarded first 5 mins? | Validated SS method? |
| --- | --- | --- | --- | --- | --- | --- | --- | --- | --- | --- | --- | --- | --- |
| Balci (2021) | Current Predictive Resting Metabolic Rate Equations Are Not Sufficient to Determine Proper Resting Energy Expenditure in Olympic Young National Team Athletes | HERE | **✓** | **✕** | **✓** | **✕** | **✕** | **?** | **✓** | **✕** | **✓** | **✓** | **✓** |
| Carlsohn (2011) | Resting Metabolic Rate in Elite Rowers and Canoeists: Difference between Indirect Calorimetry and Prediction | HERE | **✓** | **✓** | **✓** | **✓** | **✕** | **✕** | **✓** | **✓** | **✓** | **✓** | **✓** |
| De Lorenzo (1999) | A new predictive equation to calculate resting metabolic rate in athletes | HERE | **✓** | **✓** | **NR** | **NR** | **✓** |  | **✓** | **✕** | **NR** | **✕** | **✓** |
| Devrim-Lanpir (2019) | Is there any predictive equation to determine resting metabolic rate in ultra-endurance athletes? | HERE | **✓** | **✕** | **✓** | **✕** | **✕** | **✓** | **✓** | **✕** | **✓** | **✓** | **✓** |
| Gravante (2001) | The basal energy expenditure of female athletes vs. sedentary women as related to their family history of type 2 diabetes | HERE | **✓** | **✓** | **NR** | **NR** | **✕** | **✕** | **✓** | **✓** | **✓** | **✓** | **✕** |
| Jagim (2019) | Sex Differences in Resting Metabolic Rate Among Athletes | HERE | **✓** | **✓** | **✓** | **NR** | **✕** | **✕** | **✓** | **NR** | **NR** | **✓** | **✓** |
| Jagim (2018) | The accuracy of resting metabolic rate equations in athletes | HERE | **✓** | **✓** | **NR** | **NR** | **✕** | **✕** | **✓** | **NR** | **NR** | **✓** | **✓** |
| Joseph (2017) | Are Predictive Equations for Estimating Resting Energy Expenditure Accurate in Asian Indian Male Weightlifters? | HERE | **✕** | **✓** | **NR** | **✓** | **?** |  | **✓** | **✓** | **✕** | **✕** | **✕** |
| Koehler (2016) | Low resting metabolic rate in exercise-associated amenorrhea is not due to a reduced proportion of highly active metabolic tissue compartments | HERE | **✓** | **✓** | **✓** | **✓** | **✓** |  | **✓** | **NR** | **NR** | **✓** | **✓** |
| Langan-Evans (2020) | The Psychological and Physiological Consequences of Low Energy Availability in a Male Combat Sport Athlete | HERE | **NR** | **✓** | **✓** | **NR** | **NR** | **NR** | **✓** | **?** | **NR** | **NR** | **NR** |
| Mackay (2019) | The Validity of Resting Metabolic Rate-Prediction Equations and Reliability of Measured RMR in Female Athletes | HERE | **✓** | **✓** | **✓** | **NR** | **✕** | **✕** | **✓** | **NR** | **NR** | **✕** | **✕** |
| MacKenzie-Shalders (2020) | Change in resting metabolic rate in rugby athletes during a rugby preseason – do increases in skeletal muscle mass increase athlete energy expenditure? | HERE | **✓** | **✓** | **NR** | **NR** | **✕** | **✕** | **✓** | **?** | **?** | **✕** | **✕** |
| Marra (2021) | Resting energy expenditure in elite athletes: development of new predictive equations based on anthropometric variables and bioelectrical impedance analysis derived phase angle | HERE | **✓** | **✓** | **✓** | **✓** | **✕** | **✕** | **✓** | **NR** | **✓** | **✓** | **?** |
| Marques (2021) | Basal metabolic rate for high-performance female karate athletes | HERE | **NR** | **NR** | **NR** | **NR** | **NR** | **NR** | **✓** | **✕** | **✓** | **✕** | **✕** |
| Melin (2015) | Energy availability and the female athlete triad in elite endurance athletes | HERE | **✓** | **✓** | **NR** | **✓** | **✕** | **✕** | **✓** | **NR** | **NR** | **✓** | **✕** |
| Morehan (2016) | The Assessment of Total Energy Expenditure During a 14-Day In-Season Period of Professional Rugby League Players Using the Doubly Labelled Water Method | HERE | **?** | **NR** | **NR** | **NR** | **✕** | **✕** | **✓** | **NR** | **✓** | **NR** | **NR** |
| Moss (2021) | Assessment of energy availability and associated risk factors in professional female soccer players | HERE | **✕** | **✓** | **✓** | **NR** | **✕** | **✕** | **✓** | **✕** | **✓** | **✓** | **✕** |
| Nicols (2020) | Accuracy of resting metabolic rate prediction equations among healthy adults in Trinidad and Tobago. | HERE | **✓** | **✕** | **NR** | **NR** | **✓** |  | **NR** | **✕** | **NR** | **✕** | **✕** |
| O’Neill (2022) | Resting Metabolic Rate in Female Rugby Players: Differences in Measured Versus Predicted Values | HERE | **✓** | **✓** | **NR** | **NR** | **✕** | **✕** | **✓** | **✕** | **✓** | **✓** | **✓** |
| Sjodin (1996) | The influence of physical activity on BMR | HERE | **✓** | **✓** | **✓** | **✓** | **✓** |  | **✓** | **NR** | **✓** | **✓** | **✕** |
| Staal (2018) | Low RMR(ratio) as a Surrogate Marker for Energy Deficiency, the Choice of Predictive Equation Vital for Correctly Identifying Male and Female Ballet Dancers at Risk. | HERE | **✓** | **✓** | **✓** | **NR** | **✕** | **✕** | **NR** | **NR** | **NR** | **✓** | **✕** |
| Stenqvist (2020) | Impact of a 4-Week Intensified Endurance Training Intervention on Markers of Relative Energy Deficiency in Sport (RED-S) and Performance Among Well-Trained Male Cyclists | HERE | **NR** | **NR** | **NR** | **NR** | **✕** | **✕** | **NR** | **NR** | **NR** | **✓** | **✓** |
| Strock (2020) A | Indices of Resting Metabolic Rate Accurately Reflect Energy Deficiency in Exercising Women | HERE | **✓** | **✓** | **✓** | **✓** | **✓** |  | **✓** | **NR** | **NR** | **NR** | **✓** |
| Strock (2020) B | Characterizing the resting metabolic rate ratio in ovulatory exercising women over 12 months | HERE | **✓** | **✓** | **✓** | **✓** | **✓** |  | **✓** | **NR** | **NR** | **?** | **✓** |
| Ten-Haff (2014) | Resting Energy Expenditure Prediction in Recreational Athletes of 18–35 Years: Confirmation of Cunningham Equation and an Improved Weight-Based Alternative | HERE | **✕** | **✕** | **NR** | **NR** | **✓** |  | **✓** | **NR** | **NR** | **✓** | **?** |
| Thompson (1993) | Resting Metabolic Rate and Thermic Effect of a Meal in Low- and Adequate-Energy Intake Male Endurance Athletes | HERE | **✕** | **✓** | **NR** | **NR** | **✓** |  | **NR** | **NR** | **NR** | **✕** | **✕** |
| Tinsley (2019) | Resting metabolic rate in muscular physique athletes: validity of existing methods and development of new prediction equations | HERE | **✕** | **✓** | **✓** | **NR** | **✓** |  | **✓** | **NR** | **✓** | **✓** | **✓** |
| Watson (2019) | Determining a Resting Metabolic Rate Prediction Equation for Collegiate Female Athletes | HERE | **✕** | **✓** | **✓** | **✓** | **NR** | **NR** | **✓** | **✕** | **✓** | **✓** | **✓** |
| Wong (2012) | Predicting basal metabolic rates in Malaysian adult elite athletes | HERE | **✕** | **✓** | **✓** | **NR** | **✓** |  | **✓** | **✕** | **✓** | **✓** | **✓** |

| **Key** | | | |
| --- | --- | --- | --- |
| Yes = | **✓** | Unclear = | **?** |
| No = | **✕** | Not Reported = | **NR** |
